# Artemisinin–(Iso)quinoline Hybrids by C−H Activation and Click Chemistry: Combating Multidrug‐Resistant Malaria

**DOI:** 10.1002/anie.201907224

**Published:** 2019-08-08

**Authors:** Aysun Çapcı, Mélanie M. Lorion, Hui Wang, Nina Simon, Maria Leidenberger, Mariana C. Borges Silva, Diogo R. M. Moreira, Yongping Zhu, Yuqing Meng, Jia Yun Chen, Yew Mun Lee, Oliver Friedrich, Barbara Kappes, Jigang Wang, Lutz Ackermann, Svetlana B. Tsogoeva

**Affiliations:** ^1^ Organic Chemistry Chair I and Interdisciplinary Center for Molecular Materials (ICMM) Friedrich-Alexander University of Erlangen-Nürnberg Nikolaus-Fiebiger-Straße 10 91054 Erlangen Germany; ^2^ Institut für Organische und Biomolekulare Chemie Georg-August-Universität Göttingen Tammannstraße 2 37077 Göttingen Germany; ^3^ Institute of Medical Biotechnology Friedrich-Alexander University of Erlangen-Nürnberg Paul-Gordon-Straße 3 91052 Erlangen Germany; ^4^ Fiocruz, Instituto Gonçalo Moniz, 40296-710 Salvador, BA Brazil; ^5^ Artemisinin Research Center, and Institute of Chinese Materia Medica China Academy of Chinese Medical Sciences Beijing 100700 China; ^6^ Department of Biological Sciences National University of Singapore 117600 Singapore Singapore; ^7^ Shenzhen People's Hospital Shenzhen 518020 China; ^8^ German Center for Cardiovascular Research (DZHK) Germany

**Keywords:** antimalarial agents, artemisinin, drug resistance, hybridization, proteomics

## Abstract

A substantial challenge worldwide is emergent drug resistance in malaria parasites against approved drugs, such as chloroquine (CQ). To address these unsolved CQ resistance issues, only rare examples of artemisinin (ART)‐based hybrids have been reported. Moreover, protein targets of such hybrids have not been identified yet, and the reason for the superior efficacy of these hybrids is still not known. Herein, we report the synthesis of novel ART–isoquinoline and ART–quinoline hybrids showing highly improved potencies against CQ‐resistant and multidrug‐resistant *P. falciparum* strains (EC_50_ (Dd2) down to 1.0 nm; EC_50_ (K1) down to 0.78 nm) compared to CQ (EC_50_ (Dd2)=165.3 nm; EC_50_ (K1)=302.8 nm) and strongly suppressing parasitemia in experimental malaria. These new compounds are easily accessible by step‐economic C−H activation and copper(I)‐catalyzed azide–alkyne cycloaddition (CuAAC) click reactions. Through chemical proteomics, putatively hybrid‐binding protein targets of the ART‐quinolines were successfully identified in addition to known targets of quinoline and artemisinin alone, suggesting that the hybrids act through multiple modes of action to overcome resistance.

## Graphical abstract

**ARTful hybrids**: Artemisinin (ART)‐based hybrids that are highly potent against CQ/drug‐sensitive and multidrug‐resistant parasites and of strong efficacy in experimental malaria were synthesized. New putatively hybrid‐binding proteins, in addition to known target proteins of quinoline and artemisinin, were identified by chemical proteomics, suggesting that the hybrids act through multiple modes of action to overcome resistance.

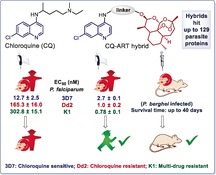

## Introduction

**Figure 1:**
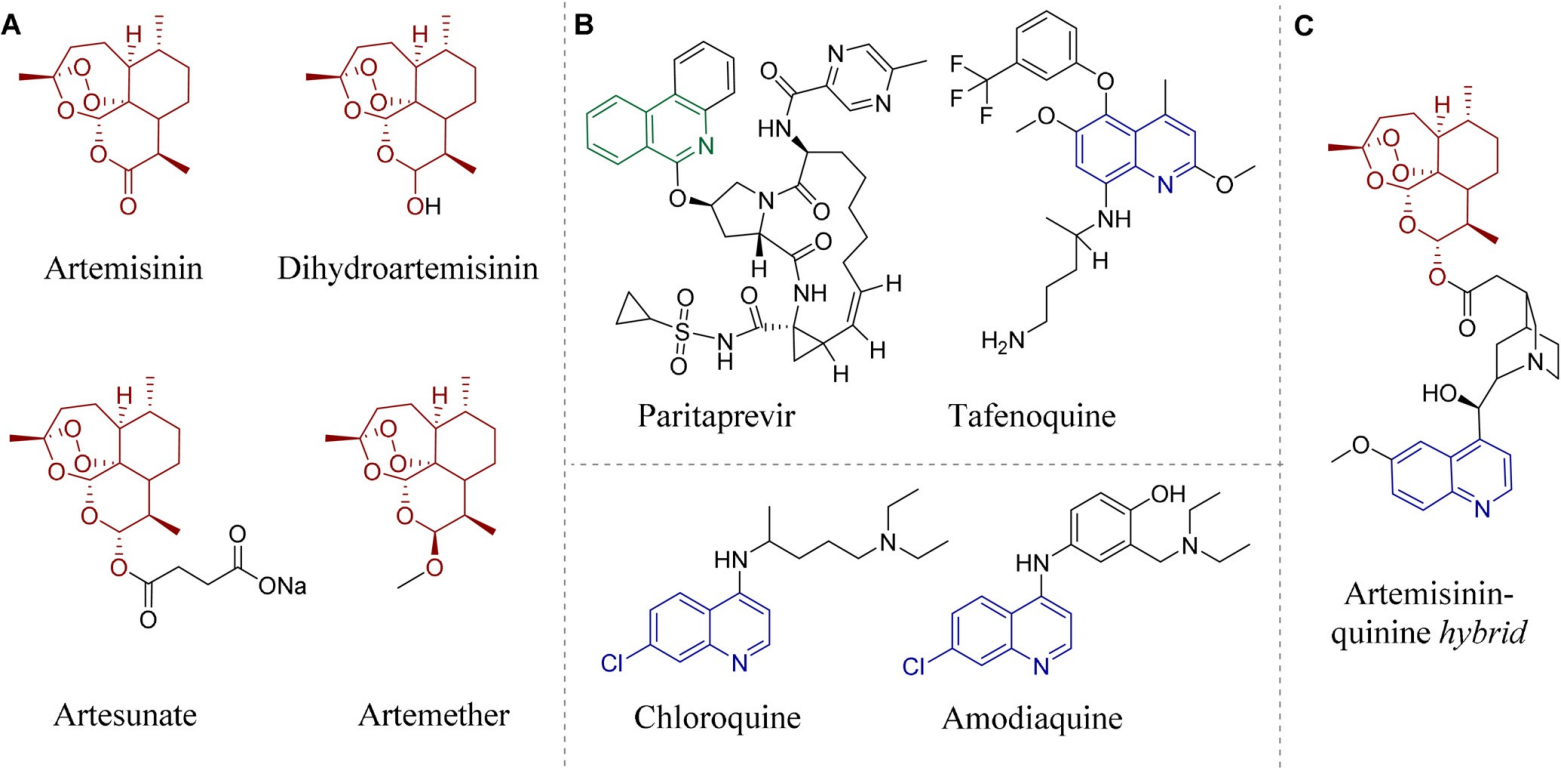
Selected artemisinin derivatives and pharmaceutically relevant isoquinolines/quinolines. A) Artemisinin (naturally occurring), dihydroartemisinin, artesunate, and artemether (semisynthetic derivatives). B) Clinically applied drugs with a quinoline or isoquinoline core: paritaprevir, tafenoquine, chloroquine, and amodiaquine. C) First example of an artemisinin–quinine hybrid.9a

Every year malaria parasites cause approximately 200 million infections in humans and almost half a million deaths according to the World Health Organization.^1^ *Plasmodium falciparum* (*P. falciparum*), representing the most pathogenic species of this eukaryotic genus, is mostly responsible for lethal courses of infection. Currently, artemisinin (ART; Figure 1 A) and quinoline derivatives (e.g., chloroquine (CQ); Figure 1 B) are the two main classes of antimalarial drugs.^2^ ART is an enantiopure sesquiterpene lactone, which was first extracted from the Chinese medicinal plant *Artemisia annua L*. (sweet wormwood) in 1972 by Youyou Tu (Nobel Prize 2015).^3^ Isoquinolines and quinolines are further privileged scaffolds, used clinically as antimalarial and antiviral drugs, for example, through the different commercial drugs amodiaquine, paritaprevir, and tafenoquine (Figure 1 B). Inspired by these traditional and pharmacologically well‐established compounds, many derivatives of artemisinin and quinoline have been designed, synthesized, and biologically investigated.^4^

The emergence of parasite resistance against approved drugs has been on the rise over the last decades, and medical concerns have thus increased dramatically. Two resistant strains Dd2 and K1 carry mutations and a number of copies for genes^5^ encoding for multidrug resistance transporters, resulting in resistances against currently available antimalarial drugs. Notably, Dd2 is resistant to CQ, mefloquine, and the antifolates sulfadoxine, pyrimethamine, and cycloguanil, whereas K1 is resistant to CQ and antifolates.^5^ In addition, they show different mutations, leading to an amino acid exchange of a target enzyme resulting in resistance against antifolates (sulfadoxine, pyrimethamine, cycloguanil). Compared with chloroquine/drug‐sensitive strains (3D7), the fitness and thus the reproduction rate of chloroquine/multidrug‐resistant (Dd2 and K1) parasites is slightly impaired by resistance‐mediating polymorphisms in putative drug transporters and/or in target enzymes,^6^ even without drug pressure.

CQ‐resistant *P. falciparum* has been reported from almost all malaria‐endemic countries. Moreover, the therapeutic achievements based on the natural product ART and its semi‐synthetic derivatives (dihydroartemisinin, artesunate, artemether; Figure 1 A) are also threatened by emerging drug resistance.^7^ Therefore, the WHO discourages mono‐therapy, which primarily promotes further development of resistance to ART in endemic areas, and recommends the use of artemisinin‐based combination therapies (ACTs) to reduce the risk of drug resistance. ACTs use ART or its derivatives together with one or more other antimalarial drugs that act through different mechanisms.^8^

Recently, structural fusions of artemisinins and quinolines, namely hybrid molecules (see Figure 1 C for an example), have been described as being even more efficient against different biological threats, such as malaria parasites and cancer cells.^9^ Hybridization appears as a powerful concept^10^ to increase the activity or pharmacological efficacy of known drugs or the bioactive constituents of the hybrid molecule^11^ and to potentially overcome drug resistance,^12^, ^13^ as hybrids can be less susceptible to drug resistance.

Whereas some examples of hybrid molecules of quinoline and ART exist, neither systematic SAR (structure–activity relationship) studies with different linkers nor identifications of their protein targets are known yet, while ART–isoquinoline hybrids have not even been reported thus far. To fill these gaps, we herein present the structural design, synthesis, and assessment of the in vitro antimalarial activities of 17 new ART–isoquinoline and ART–quinoline hybrid compounds able to combat multidrug‐resistant malaria. The antimalarial activities of the resulting hybrids were tested on the *P. falciparum* drug‐sensitive strain 3D7 and the two multidrug‐resistant strains Dd2 and K1.

The applied new isoquinoline precursors were accessible by versatile cobalt(III)‐catalyzed step‐economic C−H activation methods, while the linkers in the hybrids were generated using CuAAC (copper‐catalyzed azide–alkyne cycloaddition) click reactions and a novel rearrangement of the in situ formed tertiary amides to secondary amides. The SAR study revealed important aspects of the linker group, which led us to identify even more potent antimalarial hybrid compounds able to overcome drug resistance. One reason for the increased activity of the hybrids versus their individual constituents could be the simultaneous cellular uptake of both pharmacophores owing to their covalent linking, which is not possible for combination therapy. Besides, a possible synergistic effect of the applied pharmacophores might partially explain the pronounced bioactivity of the hybrids.

We further experimentally demonstrated the efficacy of selected hybrid compounds against malaria in *P. berghei*‐infected mice. Importantly, the proteomic characteristics of the hit hybrid compounds were assessed to identify putative targets in the *Plasmodium* proteome. We have shown that the hybridization of two individual pharmacophores into a new bioactive compound has high potential in addressing resistance issues because hybrids can simultaneously target proteins of both pharmacophores (artemisinin and quinoline), as well as new hybrid‐binding proteins. Overall, our findings are highly relevant towards the future development of effective multi‐action drugs against resistant malaria.

## Results and Discussion

### Chemistry

**Figure 2:**
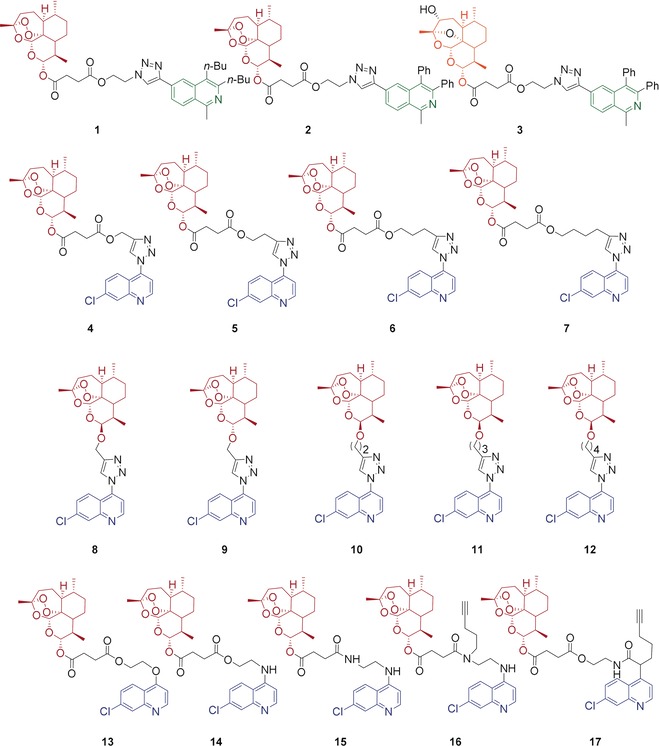
Novel hybrids **1**–**17** applied for activity examination against *P. falciparum* 3D7, Dd2, and K1 strains. Red, blue, and green indicate the parent pharmacophores of the molecules. The black moieties represent the linker groups of the molecules. The 3‐hydroxy‐desoxydihydroartemisinin unit of hybrid **3** is shown in orange.

The synthesis of the new hybrids **1**–**17** (Figure 2) is discussed in two parts according to the chemical reactions that were applied to form the corresponding linker: 1) hybrid synthesis by Cu^I^‐catalyzed click reactions; and 2) hybrid synthesis by esterification, amide bond formation, and a novel rearrangement of the in situ formed tertiary amide into a secondary amide.

#### Hybrid Synthesis by Copper(I)‐Catalyzed Click Reactions

**Scheme 1:**
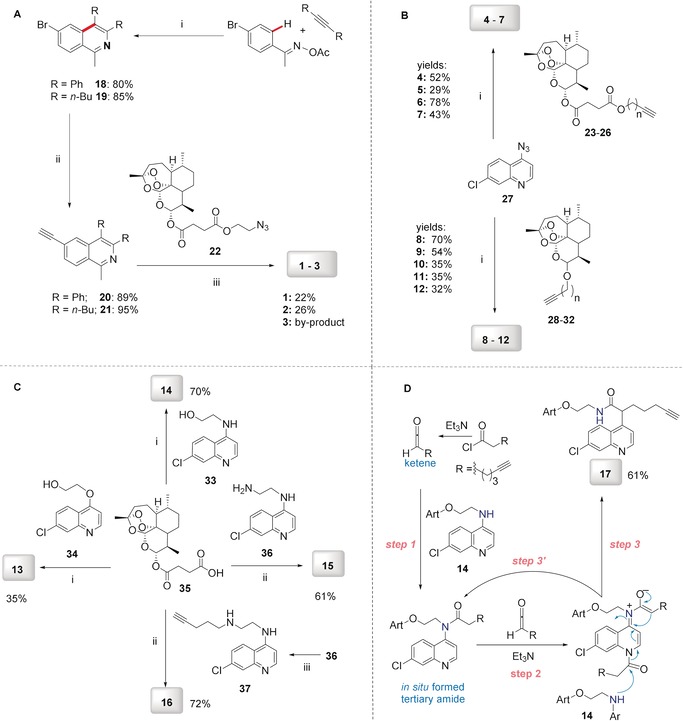
A) Synthesis of isoquinoline–artesunic acid hybrids **1**–**3**. B) Synthesis of 7‐chloroquinoline–artesunic acid hybrids **4**–**7** and 7‐chloroquinoline–artemisinin hybrids **8**–**12**. C) Synthesis of 7‐chloroquinoline–artesunic acid hybrids **13**–**16**. D) Post‐modification of hybrid **14** to hybrid **17** in a novel domino process: in situ tertiary amide formation/intramolecular rearrangement reaction. Reagents and Conditions: A) (i) [Cp*Co(CO)I_2_] (10 mol %), AgSbF_6_ (20 mol %), NaOAc (20 mol %), 1,2‐dichloroethane, 120 °C, 1 h; (ii) 1) PdCl_2_(PPh_3_)_2_ (1.0 mol %), trimethylsilylacetylene, triethylamine, 50 °C, 2 h, 2) K_2_CO_3_, methanol, 25 °C, 4 h; (iii) CuSO_4_⋅5 H_2_O, sodium ascorbate, CH_2_Cl_2_/H_2_O, overnight. B) (i) CuSO_4_⋅5 H_2_O, sodium ascorbate, CH_2_Cl_2_/H_2_O, rt, overnight. **23**: *n*=1; **24**: *n*=2; **25**: *n*=3; **26**: *n*=4; **28**: *n*=1 (C‐10β); **29**: *n*=1 (C‐10α); **30**: *n*=2 (C‐10β); **31**: *n*=3 (C‐10β); **32**: *n*=4 (C‐10β). C) (i) DCC, DMAP, CH_2_Cl_2_, 0 °C→rt, overnight, N_2_; (ii) EDCI, DMAP, CH_2_Cl_2_, 0 °C→rt, overnight, N_2_; (iii) K_2_CO_3_, acetonitrile, 130 °C, overnight. D) In CH_2_Cl_2_, 0 °C→rt, 18 h, N_2_.

We first planned to investigate the variation of bioactivity when the linker bears a triazole entity. The CuAAC click reaction has become one of the most important chemical reactions in the field of medicinal chemistry with its highly preferable features. The resulting products feature a five‐membered triazole ring as the linker group, formed by 1,3‐dipolar cycloaddition (click) reaction. This five‐membered ring is a building block very often employed in valuable pharmaceuticals.^14^ As illustrated in Schemes 1 A and 1 B, we thus obtained hybrids **1**–**12**, bearing a triazole linker.

The artemisinin–isoquinoline hybrid compounds **1**–**3** were prepared (Scheme 1 A) through a CuAAC click reaction of new artesunic acid based azide **22** with the isoquinoline‐derived alkynes **20** and **21** in the presence of CuSO_4_⋅5 H_2_O and sodium ascorbate. The corresponding isoquinoline precursors **20** and **21** were synthesized by facile C−H cobaltation of *O*‐acetyl oximes with internal alkynes,^15^ followed by Sonogashira coupling of the obtained 6‐bromo‐isoquinolines **18** and **19** with trimethylsilylacetylene (Scheme 1 A and the Supporting Information). We were also able to isolate compound **3**, which is a side product of the formation of hybrid **2**. We found that the artemisinin moiety is hydroxylated at the C3 position to a desoxydihydroartemisinin derivative in the presence of copper(I) species, as reported previously.^16^ This 3‐hydroxy‐desoxydihydroartemisinin derivative **3** was also subjected to bioactivity examination.

The required new artesunic acid alkyne derivatives **23**–**26** (Scheme 1 B) were prepared by esterification reaction using EDCI (1‐ethyl‐3‐(3‐dimethylaminopropyl)carbodiimide), DCC (*N*,*N′*‐dicyclohexylcarbodiimide), and DMAP (4‐dimethylaminopyridine) in CH_2_Cl_2_ at room temperature, starting from artesunic acid (**35**), and 2‐propyn‐1‐ol, 3‐butyn‐1‐ol, 4‐pentyn‐1‐ol, and 5‐hexyn‐1‐ol, respectively (see the Supporting Information). Using the same set of alcohols, etherification reactions were performed with dihydroartemisinin to obtain precursors **28**–**32**. The subsequent 1,3‐dipolar cycloaddition reactions of artesunic acid based and dihydroartemisinin‐based alkyne derivatives **23**–**32** with 4‐azido‐7‐chloroquinoline (**27**) towards the corresponding hybrids **4**–**12** are presented in Scheme 1 B.

#### Hybrid Synthesis by Esterification, Amidation, and a Novel Rearrangement

We next enlarged the scope of the hybrids by diversifying the linker to examine the SAR for further medicinal chemistry insight. The new hybrid molecules **13**–**16** were synthesized from artesunic acid **35** with the corresponding 7‐chloroquinoline derivatives **33**, **34**, **36**, and **37** (Scheme 1 C and the Supporting Information). While the quinoline‐derived precursors **33**, **34**, and **36** were prepared based on previous reports,^17^ compound **37** was synthesized for the first time by a nucleophilic aliphatic substitution reaction of 5‐chloropentyne with quinoline‐based amine **36** (Scheme 1 C and the Supporting Information). The hybrid compounds **13** and **14** were obtained by esterification of artesunic acid **35** with the 7‐chloroquinoline‐derived primary alcohols **34** and **33**, using the coupling reagents DCC and DMAP in CH_2_Cl_2_ under nitrogen atmosphere_._ Primary and secondary amine derivatives of 7‐chloroquinoline (**36** and **37**) were used for amide coupling reactions in the presence of EDCI and DMAP in CH_2_Cl_2_ to furnish hybrids **15** and **16** (Scheme 1 C).

The alkyne‐tagged hybrid **16** was synthesized to identify its target proteins in *P. falciparum* by proteomics. To obtain another alkyne‐tagged hybrid compound for proteomics studies, we applied hybrid **14** for further modification through acylation of the secondary amine with hept‐6‐ynoyl chloride (Scheme 1 D). This experiment did not result in the expected tertiary amide product. Instead, we observed the product of a novel metal‐free intramolecular rearrangement of the in situ formed tertiary amide into the secondary amide hybrid **17**, which we used for proteomics studies. A possible mechanism of this novel multistep domino reaction is shown in Scheme 1 D. Triethylamine promotes dehydrohalogenation of chloro acetyl chloride to generate the corresponding ketene,^18^ which subsequently reacts with **14** to a tertiary amide (step 1), which undergoes a second acylation reaction (step 2). The in situ formed C1‐ammonium enolate intermediate undergoes an intramolecular C−C bond formation/C−N bond cleavage sequence (step 3) towards the final product **17**. Secondary amine **14** facilitates the rearomatization process via its acylation to a tertiary amide (step 3′).

### Antimalarial Activity

Remarkably, all investigated artemisinin‐derived hybrid compounds in this study (Figure 2) exhibited potent antimalarial activity in the nanomolar to picomolar range against chloroquine/drug‐sensitive (3D7) and chloroquine/multidrug‐resistant (Dd2 and K1) parasite strains (Table 1).

**Table 1:** EC_50_ values for hybrids **1**–**17**, hybrid precursors **33**–**37**, **18**, chloroquine (CQ), artemisinin (ART), and artesunic acid tested against *P. falciparum* parasite strains 3D7, Dd2, and K1.

Compound		EC_50_ [nm]±S.E.M.		
		3D7	Dd2	K1
**1**	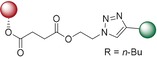	9.2±0.7	10±0.8	4.2±0.4
**2**	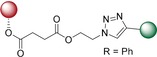	5.3±0.2	4.8±0.1	3.3±0.7
**3** ^[a]^	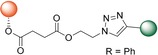	646.6±94.4	444.0±107.5	255.5±68.6
**4**	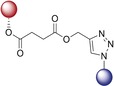	5.3±1.0	4.2±0.3	1.8±0.2
**5**	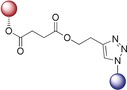	8.0±1.3	4.9±0.3	2.6±0.2
**6**	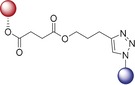	5.4±0.6	4.6±0.2	2.4±0.1
**7**	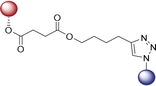	6.2±0.4	4.4±0.4	2.5±0.4
**8**	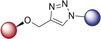	9.0±1.5	5.9±0.6	4.3±0.7
**9** ^[b]^	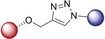	27.5±2.9	10.6±1.3	5.9±1.4
**10**	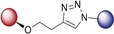	8.8±1.1	4.5±0.3	2.0±0.2
**11**	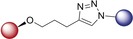	9.4±1.1	5.1±0.2	3.8±0.4
**12**	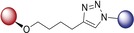	8.4±1.6	6.1±0.2	3.7±0.5
**13**	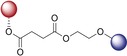	14.8±0.4	10.6±0.4	5.5±0.4
**14**	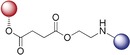	4.5±0.5	2.3±0.6	1.7±0.3
**15**	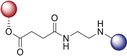	2.7±0.1	1.0±0.2	0.78±0.1
**16**	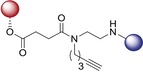	3.5±0.3	1.6±0.3	1.3±0.3
**17**	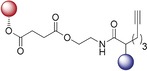	6.0±0.4	4.8±0.9	2.2±0.2
**18**	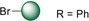	16 400	18 400	22 100
**27**	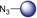	1980	1890	2860
**33**	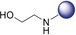	760	1600	2400
**36**	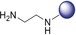	17.4±0.6	483.3±44.0	512.3±49.7
**37**	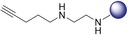	13.3±0.7	41.5±4.9	53.8±7.5
CQ	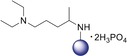	12.7±2.5	165.3±16	302.8±15.1
ART	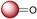	26.8±2.4	11.3±1.8	5.4±0.5
artesunic acid (**35**)	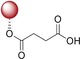	14.4±2.0	10.9±0.7	5.2±0.8

[a] Because of the limited available quantity, biological replicates were only done twice for the Dd2 and K1 strain. [b] Precipitates: The compound had to be heated at 55 °C for 15 min to dissolve, which resulted in an extreme variation in the EC_50_ values especially in the case of hybrid **11**. EC_50_=half‐maximal effective concentration; S.E.M.=standard error of the mean.

While quinolines are well‐known for their antimalarial activity, much less is known about the antimalarial activity of isoquinolines and their structural determinants. In the following, the novel ART–isoquinolines hybrids **1**–**3** were initially investigated. The EC_50_ (half‐maximal effective concentration) values of hybrids **1** and **2** against 3D7, Dd2, and K1 strains of *P*. *falciparum* are 9.2, 10.0, and 4.2 nm and 5.3, 4.8, and 3.3 nm, respectively, indicating the potent activity of these novel hybrids compared to parent compounds artesunic acid **35** and isoquinoline precursor **18**. Furthermore, we observed that the antimalarial activity of hybrid **3** is 100 times lower than that of hybrid **2**. The drop in activity of 3‐hydroxy‐desoxydihydroartemisinin derivative **3** underlines the importance of the 1,2,4‐trioxane fragment for high activity.

Next, we examined the influence of the linker length on the activity of the artesunic acid–quinoline hybrid compounds **4**–**7** (Figure 2), where the linker length is elongated from one to four carbon atoms. However, among these hybrids **4**–**7**, the elongation of the linker did not change the antimalarial activity of the compounds in a substantial manner (Table 1). These hybrids are highly potent antimalarial compounds, with EC_50_ values in the nanomolar range, and the shortest linker allowed us to achieve the highest activity observed against all *P. falciparum* strains used in this study. CQ gave an EC_50_ value of 12.7 nm against 3D7, whereas the hybrids showed EC_50_ values of 5.3, 8.0, 5.4, and 6.2 nm (short to long carbon chain linkers), being more active than CQ (Table 1). In the case of resistant strains, hybrids **4**–**7** are even more potent, showing 34 to 39 times higher potency against Dd2 (4.2–4.9 nm), and 116 to 168 times higher activity against K1 (1.8–2.6 nm) than CQ. Moreover, triazole‐linked artesunic acid–7‐chloroquinoline hybrids are highly potent against resistant *P. falciparum* strains. All hybrids were able to overcome CQ drug resistance and multidrug resistance; most importantly they also outperformed the reference drug artemisinin. Remarkably, the hybrids are more active than their parent compound artesunic acid (**35**) and 4‐azido‐7‐chloroquinoline (**27**; 14.4/10.9/5.2 nm and 1982/2523/2939 nm, respectively) in all cases (Table 1). Thus hybridization can be used to access more potent bioactive compounds than their parent precursors for this set of hybrids **4**–**7**.

Similar to our previous results (see Table 1, hybrids **4**–**7**), ART–quinoline hybrids **8**–**12** showed great activity against malaria parasites ranging from 2.0 nm to 27.5 nm, revealing that these hybrids are also more active against CQ/multidrug‐resistant parasites than against CQ/drug‐sensitive parasites. Hybrids **8**, **10**, **11**, and **12** showed almost the same potency when they were tested against three different strains. The hybrids exhibited EC_50_ values of 9.0 nm, 8.8 nm, 9.4 nm, and 8.4 nm against 3D7; 5.9 nm, 4.5 nm, 5.1 nm, and 6.1 nm against Dd2; and 4.3 nm, 2.0 nm, 3.8 nm, and 3.7 nm against K1 strains, respectively (Table 1). Interestingly, we again did not observe a correlation between activity and linker length. Hybrids **8** and **9** are β‐ and α‐anomers because of the stereochemistry at the C10 position of the artemisinin unit. As previously reported for α‐ and β‐arteethers,^19^ we have also observed that the β‐isomer **8** is more active than the α‐isomer **9**. Nevertheless, with EC_50_ values of 27.5 nm, 10.6 nm, and 5.9 nm, the α‐isomer of this hybrid is still highly active against *P. falciparum* strains 3D7, Dd2, and K1.

Next, we tested the new 7‐chloroquinoline–artesunic acid hybrids **13**–**15**, containing ester and amide linkers, against *P. falciparum* parasites. Hybrid **14** is more active than hybrid **13**. Replacing the H‐bond acceptor oxygen atom at the C4 position of the 7‐chloroquinoline unit of hybrid **13** with an H‐bond donor amine group resulted in the formation of 4‐aminoquinoline chemical space (hybrid **14**).

As expected, this chemical modification substantially increased the potency against all *P. falciparum* strains, which is explained through the formation of the 4‐aminoquinoline, a known antimalarial core structure. The activities increased, as seen from decreases in the EC_50_ values from 14.8 to 4.5 nm for 3D7, from 10.6 to 2.3 nm for Dd2, and from 5.5 to 1.7 nm for K1 strains (Table 1). Based on these results, we assumed that the composition of the linker affects the activity. Furthermore, we decided to replace the second oxygen atom by a nitrogen atom. This designed compound **15** gave EC_50_ values of 2.7 nm, 1.0 nm, and 780 pm against 3D7, Dd2, and K1 strains, respectively (Table 1). Through this systematic replacement of heteroatoms, we examined requirements on the linker for high antimalarial activity, which led to EC_50_ values as low as 780 pm against the K1 strain with hybrid **15**. These results are consistent with those obtained with previous hybrids (**13** and **14**) when comparing the activities of the individual constituents of each hybrid against chloroquine/drug‐sensitive (3D7) and chloroquine/multidrug‐resistant (Dd2 and K1) parasites, as higher efficiencies were always observed against resistant parasites. It is impressive that the generated compounds are able to counteract all resistances presented by the Dd2 and K1 strains. On the basis of the observed SAR, we hypothesized that these artesunic acid–7‐chloroquinoline based hybrids are more potent when they bear a nitrogen atom on the linker. Moreover, they show excellent antimalarial activity.

Next, compounds **16** and **17** were evaluated as alkyne‐tagged hybrids. They were designed for proteomics experiments, which require an alkyne unit to tag the molecule to a biotin affinity tag through a click reaction. The binding proteins of these hybrids were investigated to understand the mechanism of action of the most active antimalarial hybrid. To the best of our knowledge, none of the artemisinin–quinoline hybrids have been previously investigated based on their target profiling. Hybrid **16** gave EC_50_ values of 3.5, 1.6, and 1.3 nm against 3D7, Dd2, and K1 strains, respectively (Table 1). As an analogue, our hybrid **16** can be compared with hybrid **15**, and it can be implied that the hydrogen atom on the secondary amide improves antimalarial activity. In contrast, hybrid **16** is a tertiary amide and does not provide an N−H bond, unlike hybrid **15**, and it is thus less active. When the amide nitrogen atom is occupied by an alkyne chain, the activity is lower as it is the case for hybrid **14**, which features an ester bond and a H‐bond acceptor. This finding might also be a confirmation that the H‐bond donor improves the activity. As expected, when we tested the antimalarial activity of the post‐modified hybrid **17**, the activity dropped compared to its pre‐modified version, that is, hybrid **14**. The decrease in activity is possible due to depletion of the 4‐aminoquinoline core, which is essential for heme binding.^20^ This initial set of compounds has shown that artemisinin and 7‐chloroquinoline are a promising pair to improve the activity against malaria parasites, and that the composition of the linker is a structural determinant for modulating activity.

### Parasitemia Suppression in Experimental Malaria

We evaluated the suppressive activity of the hybrids in *P. berghei*‐infected mice over four consecutive days of treatment.^21^ Compared with the vehicle group, mice treated subcutaneously with 105 μmol kg^−1^ (40 mg kg^−1^) of artesunate (ARE) showed a >99 % reduction in blood parasitemia, and this dose also conferred 100 % animal survival (Table 2, entry 2). Based on these results, we chose a dose of 105 μmol kg^−1^ given by subcutaneous injection as a starting point for testing the hybrid compounds. To gain insight into the SARs of our compounds, we selected the artesunic acid–7‐chloroquinoline based hybrid **14**, containing an ester linker group (the thermal, hydrolytic, and enzymatic stability of **14** is high; see the Supporting Information), as its potency is similar to that of alkyne‐tagged hybrid **16**, prepared for target identification experiments. In addition, the artemisinin–7‐chloroquinoline based hybrid **12**, containing a triazole linker, was selected. The use of hybrids **12** and **14** enabled us to investigate the importance of the linker group for antimalarial efficacy (Figures S1–S3 in the Supporting Information).

**Table 2:** Summary of the activities of hybrid **14** and artesunate against blood‐stage *P. berghei*‐infected mice.

Entry	Compound	Dose [μmol/kg body weight]	Parasitemia inhibition [%]^[a]^	Median survival [days]	Number of alive animals (%)^[b]^
subcutaneous administration (s.c.)					
1	vehicle	–	–	20	0/10 (0)
2	ARE	105	>99	>41	5/5 (100)
3	hybrid **14**	105	>99	>41	5/5 (100)
4	ARE	35	–	–	0/8 (0)^[c]^
5	hybrid **14**	35	95.2±4.9	>41	5/5 (80)^[d]^
					
oral administration by gavage (*P. Os*.)					
6	hybrid **14**	105	78.6±10^[e]^	35^[e]^	6/10 (60)^[e]^

[a] Values are mean±S.D. and were taken from 10 dpi. [b] Until 41 dpi. [c] Value taken from Ref. 21. [d] One of five mice were blood smear positive for parasites 41 dpi. [e] Median of two independent experiments, where error is S.E.M. dpi=days post‐infection. ARE=Artesunate. S.D.=standard deviation. S.E.M.=standard error of the mean.

At a dose of 105 μmol kg^−1^ (65 mg kg^−1^), hybrid **14** reduced parasitemia by >99 %, while at a dose of 35 μmol kg^−1^, it reduced parasitemia by 95.2±4.9 % (Table 2, entries 3 and 5). As a result of the strong parasitemia suppression, all mice treated with 105 μmol kg^−1^ of hybrid **14** remained free of parasites for up to 41 dpi (days post‐infection), and they were considered cured. When hybrid **14** was given at a dose of 35 μmol kg^−1^ (s.c.), 80 % of mice remained free of parasites up to 41 dpi, which is a cure rate higher than for artesunate treatment at the same dosage and with the experimental model.^21^ In contrast to hybrid compound **14**, hybrid **12** featuring a triazole linker did not reduce parasitemia when administered at a dose of 105 μmol kg^−1^ (Figure S2).

Having ascertained the efficacy of hybrid **14** upon subcutaneous administration, we tested whether this compound is able to inhibit parasitemia upon oral administration (Table 2, entry 6). Compound **14** displayed strong antiparasitic activity, reducing parasitemia by 78.6±10 % and increasing the median animal survival time compared to the vehicle group. From two independent experiments, 60 % of mice remained free of parasites in the group treated orally with hybrid **14** (Table 2, entry 6). In contrast, infected mice orally treated with ARE at a dose of 105 μmol kg^−1^ were not cured. In fact, ARE is curative only when administered subcutaneously^22^ or in combination with mefloquine. The fact that hybrid **14** inhibited parasitemia and provided protection from mortality upon oral uptake indicates that this compound has oral efficacy, which is a highly desired property for a new antimalarial drug candidate.

ART and ARE are known to have a fast onset of antimalarial action, which causes a rapid decrease in parasitemia, but they also have a short half‐life.^23^ Unlike ARE, hybrid **14** presented a more long‐lasting action. This was inferred when instead of the standard treatment for four consecutive days, mice were treated at intercalated days at 3 and 48 h post‐infection (Figure S4 and Table S1). At day 10 post‐infection, both hybrid **14** and artesunate treatment suppressed parasitemia; however, at day 12 post‐infection, parasitemia had increased rapidly (i.e., recrudescence of parasitemia) in four out of five mice, and at day 16 post‐infection, all mice treated with ARE showed parasite recrudescence. In contrast, only two of five mice treated with hybrid **14** presented detectable parasites at day 12 post‐infection, showing that treatment with the hybrid compound is more efficient in delaying the recrudescence of parasites compared with ARE treatment.

### Curative Potential in Experimental Malaria

**Figure 3:**
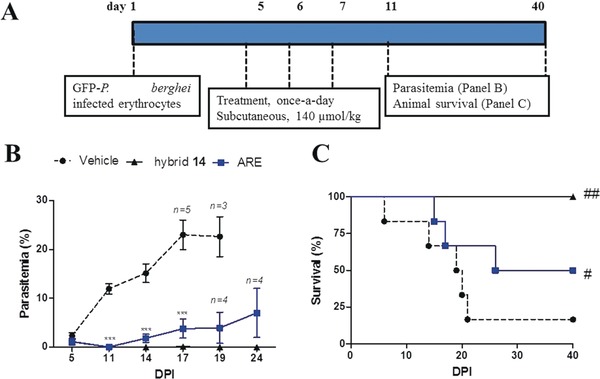
Experimental design of the curative Thompson test (A), follow‐up of parasitemia (B), and animal survival (C) in *P. berghei*‐infected mice. Parasitemia and animal survival were measured using an *n=*6 group, unless indicated otherwise. Error bars indicate S.D. ***p<0.01 (two‐way ANOVA) versus vehicle group. ^#^ *p*<0.01, ^##^ *p*<0.005 (Log‐rank, Mantel‐Cox test) versus vehicle. dpi=days post‐infection. S.D.=standard deviation.

The fact that hybrid **14** presented impressive parasitemia suppression led us to determine its curative potential (Thompson test). As expected, hybrid **14** cured 100 % of mice, while artesunate only cured 33 % of mice when administered at the same dose (Figure 3 and Table S2). Based on the cure ratio, we estimated that hybrid **14** is threefold more effective than ARE.

Apart from piperaquine, the required dose of antimalarials to cure mice in the curative model of Thompson is at the very least two times higher than the required dose to protect mice from mortality in the suppressive model of Peters.^24^ Strikingly, we observed that the sum of dosages of the hybrid compound employed in the suppressive test was enough to provide cure. This puts hybrid compound **14** in an efficacy range that is only comparable to those of piperaquine and ACTs.

### The Underlying Mechanism of Potency Enhancement

**Figure 4:**
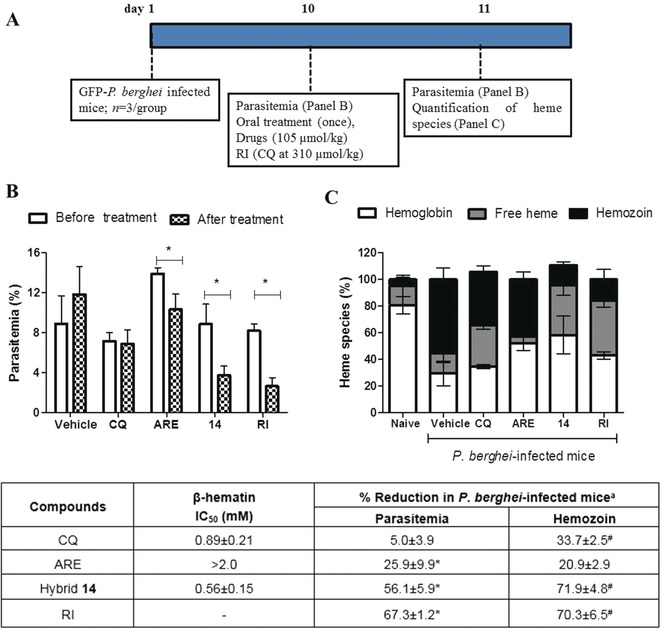
Experimental design (A), parasitemia (B), heme species (C), and summary of results (Table) from *P. berghei*‐infected mice within 24 h of oral treatment. In (B), parasitemia was determined by GFP signal using flow cytometry. In (C), heme species were quantified from the peripheral blood after mouse euthanasia. The results of two independent experiments with *n=*3 groups for each experiment are shown. Error bars are means and 95 % CI (95 % confidence interval). IC_50_ and % reduction values are mean±S.D. In the table, β‐hematin formation was determined 24 h after drug incubation. [a] Values relative to the infected untreated group (vehicle). *p<0.05 (one‐way ANOVA and Newman‐Keuls multiple comparison test) for before versus after treatment. ^#^ *p*<0.05 in comparison to vehicle. CQ=Chloroquine, ARE=Artesunate. RI=Reference inhibitor (CQ at 310 μmol kg^−1^).

To study the origin of potency enhancement, we analyzed the potential of hybrid **14** in impairing malarial pigment formation in in vitro and in vivo models. ARE lacks inhibitory properties in the in vitro β‐hematin formation while hybrid **14** inhibited β‐hematin formation with a potency as high as that of chloroquine (Figure 4 and Table S3). Assuming that only the 7‐chloroquinoline part of hybrid compound **14** is responsible for the β‐hematin inhibitory properties,20b, 20c the observation that compound **14** is as potent as chloroquine is explained by the fact that the 7‐chloroquinoline moiety in the hybrid compound can adopt different conformations and orientations, in part greatly influenced by the linker group. While this kind of potency enhancement against β‐hematin formation has been observed for quinoline derivatives,20c it has not been demonstrated in experimental malaria. Here, we introduce the notion that the potency enhancement of hybrid compound **14** against β‐hematin correlates well with its efficacy in impairing parasite hemozoin in experimental malaria. This was confirmed in a designed experiment with *P. berghei*‐infected mice by simultaneously monitoring parasitemia and heme species, including the cellular hemozoin (Figure 4).

These experiments revealed that 24 h after administration of a single oral dose (105 μmol kg^−1^), compound **14** had reduced parasitemia by 56.1 %. In addition to an impressive suppression of parasitemia, the hybrid was effective in impairing hemozoin formation by 71.9 % in comparison to untreated mice. At the same dose, ARE was only half as effective as compound **14** in reducing parasitemia but did not significantly reduce hemozoin. In contrast, CQ did not reduce parasitemia but attenuated hemozoin formation, albeit twice less than compound **14**. Concomitant to a decrease in the hemozoin level, both CQ and compound **14** increased the levels of free heme, while ARE did not, clearly showing that CQ and compound **14** reduce parasite viability by interrupting the biosynthesis of hemozoin. In terms of efficacy, compound **14** behaved similarly to the group receiving a reference inhibitor of hemozoin (CQ‐treated mice at a high dose of 100 mg kg^−1^, 310 μmol kg^−1^). Our combination of in vitro and in vivo experiments, using pharmacologically relevant drug doses, in addition to a critical quantification of free heme and hemozoin levels, allowed us to recognize that hemozoin inhibition is due to a direct effect of hybrid drug treatment rather than a consequence of parasitemia reduction. This also supports the notion that the efficacy of hybrid **14** in *P. berghei*‐infected mice is not only due to the presence of the artemisinin moiety, but also highly dependent on the quinoline.

### Chemical Proteomics Profiling of the Interactive Protein Targets of Hybrids 16 and 17 in *P. falciparum*

**Figure 5:**
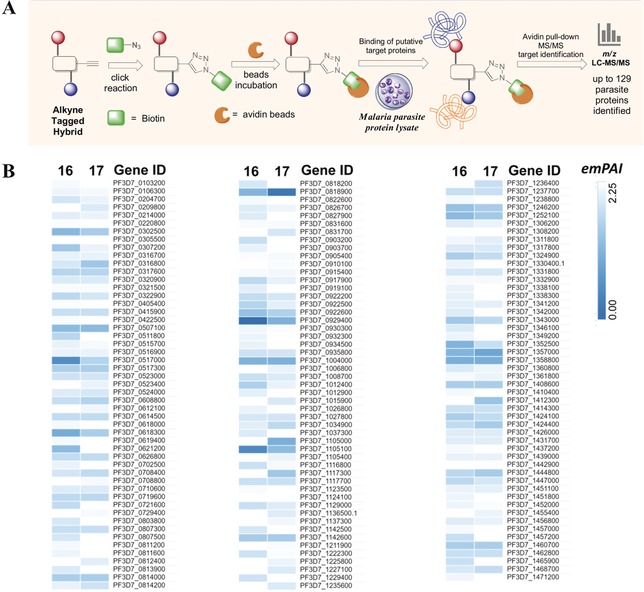
Target Identification. A) The alkyne‐tagged hybrids were reacted with biotin azide to generate the affinity probes for interactive target enrichment. Affinity pull‐down coupled mass spectrometry identification was carried out to characterize the hybrids targets. B) Heat map representation of the relative abundance of individual targets of hybrids **16** and **17**. The emPAI scores of individual proteins were used to generate the heat map with morpheus software. Complete datasets of the drug targets are shown in Table S5.

With the alkyne‐tagged artemisinin–chloroquinoline hybrids **16** and **17** in hand, we linked the alkyne‐tagged analogues to biotin azide through click chemistry (Figure 5 A). Then, the biotin‐bearing analogues were incubated with avidin beads to prepare the drug affinity beads. The enriched and purified *P*. *falciparum* parasite protein lysates were incubated with the drug‐bearing beads for 4 h. The drug binding targets were affinity‐purified by avidin beads and identified by tandem mass spectrometry. A total of 129 and 107 parasite proteins were identified as direct targets of hybrids **16** and **17** (see Table S5), while pull‐down of the dimethyl sulfoxide treated control beads did not identify any parasite proteins. The exponentially modified protein abundance index (emPAI) was used to provide a semi‐quantification of protein abundance.^25^

Heat map analysis (Figure 5 B) showed that the target profiles of hybrids **16** and **17** are generally similar to each other as the hybrids possess a similar artemisinin moiety. Consistent with the higher antimalarial potency, the target labeling potential of hybrid **16** is also slightly higher than that of hybrid **17**. Along with the target identification we found a number of proteins, such as heat shock protein 70, high‐molecular‐weight rhoptry protein 2, and 40S ribosomal protein S15, that were strongly enriched by both probes. Interestingly, we found that both artemisinin hybrid compounds **16** and **17** can interact with PfATP6, a well‐known artemisinin target.

**Figure 6:**
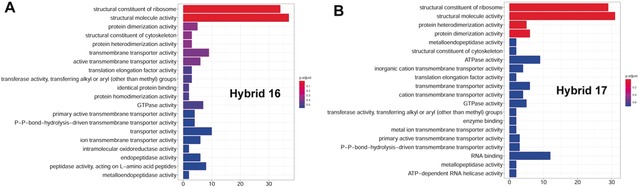
Gene ontology (GO) analysis of interactive proteins for A) hybrid **16** and B) hybrid **17**.

**Figure 7:**
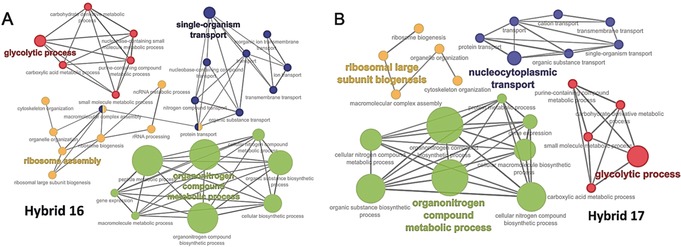
Hybrid **16** (A) and hybrid **17** (B) targets are involved in multiple biological processes essential for parasite survival.

Gene ontology pathway analysis was performed to map out the critical pathways of the drug targets involved in the antimalarial targets of the hybrid compounds (Figure 6). A number of metabolic processes are the most significant pathways affected by the two hybrids **16** and **17**, including glycolytic and organonitrogen compound metabolic processes (Figure 7).

The findings of the proteomic analysis support the notion that hybrid compounds **16** and **17** affect multiple pathways classically recognized for artemisinin action (such as the PfATP6 enzyme) and for quinolines (40S ribosomal protein machinery), which are important targets for development and survival during blood stage *P. falciparum* growth.^26^ These findings have important future implications for the development of efficient drug candidates based on ART‐derived hybrid compounds.

## Conclusion

The burden of malaria, especially its most fatal species *P. falciparum*, and the emergence of resistances to known antimalarial drugs (e.g., CQ and ART) are two of the biggest concerns for global infectious disease control. Our work provides access by, among others, environmentally benign C−H activation to novel ART‐based hybrid compounds, which show outstanding activities against the drug‐sensitive 3D7 wild‐type strain and against two multidrug‐resistant strains (Dd2, K1) of *P. falciparum* parasites, with EC_50_ values ranging from 780 pm to 27.5 nm. The hybrids are significantly more potent towards resistant parasite strains. Thus these compounds illustrate the high potential of the hybridization concept as an alternative drug discovery approach for efficient treatment and to overcome drug resistance. The potency enhancement of hybrids in comparison to their corresponding parent compounds can be explained through the simultaneous cellular uptake of both pharmacophores covalently bound via a linker, and by possible synergistic effects. These concepts are supported by the observation that one of the most potent hybrid compounds against *P. falciparum*, the ART–quinoline hybrid **14**, demonstrated superior efficacy in comparison to the antimalarial drug artesunate in *P. berghei*‐infected mice, being in its efficacy only comparable to ACTs. The outstanding efficacy of hybrid **14** might be due to the combination of the pharmacological features of quinolines (potent hemozoin inhibition) and artemisinins (fast parasite killing).

Finally, using a chemical proteomics approach, we identified a comprehensive set of *P. falciparum* parasite proteins as direct targets of the selected highly active hybrids **16** and **17**. The results demonstrate that the hybrid compounds affect targets important for development and survival during blood‐stage *P. falciparum* development, such as the PfATP6 enzyme (responsible for artemisinin action) and the 40S ribosomal protein machinery (classically recognized for quinoline action).

In general, these findings should provide a valuable basis for further molecular investigations of putative target proteins of these artemisinin‐based compounds. This is an important implication for antimalarial drug design, in particular for hybrid drugs that can simultaneously target protein synthesis (as observed by proteomics) and hemozoin (Hz) formation (as observed by β‐hematin assay) as well as surmount multidrug resistance (inferred by EC_50_ values). Our results should also encourage further mechanistic studies and be a stepping stone towards overcoming multidrug resistance.

## Conflict of interest

The authors declare no conflict of interest.

## Supporting information

As a service to our authors and readers, this journal provides supporting information supplied by the authors. Such materials are peer reviewed and may be re‐organized for online delivery, but are not copy‐edited or typeset. Technical support issues arising from supporting information (other than missing files) should be addressed to the authors.

Supplementary
