# Supplementary material for: Artemisinin–(Iso)quinoline Hybrids by C−H Activation and Click Chemistry: Combating Multidrug‐Resistant Malaria
Source: Angew Chem Int Ed Engl. 2019 Aug 8;58(37):13066–79. doi: 10.1002/anie.201907224 (PMC6899722; doi:10.1002/anie.201907224)
Supplement: Supplementary file 1 — Supplementary [file ANIE-58-13066-s001.pdf]

Angewandte Chemie
Eine Zeitschrift der Gesellschaft Deutscher Chemiker

Supporting Information

# Artemisinin–(Iso)quinoline Hybrids by C−H Activation and Click Chemistry: Combating Multidrug-Resistant Malaria

*Aysun Çapcı, Mélanie M. Lorion, Hui Wang, Nina Simon, Maria Leidenberger, Mariana C. Borges Silva, Diogo R. M. Moreira, Yongping Zhu, Yuqing Meng, Jia Yun Chen, Yew Mun Lee, Oliver Friedrich, Barbara Kappes, Jigang Wang,* Lutz Ackermann,* and Svetlana B. Tsogoeva**

anie_201907224_sm_miscellaneous_information.pdf

# Table of Contents

**1.** General information

**2.** Synthesis and characterization of compounds

**3.** Stability experiments

**4.** Biological methods

**5.** $^{1}H$ and $^{13}C$ NMR spectra and mass spectra of the compounds

**6.** NMR spectra of the compounds after stability tests

**7.** Filtering PAINS elements among hybrid compounds **1 - 17**

**8.** References

## 1. General information

All reactions were performed in distilled and dried or HPLC grade solvents under $N_2$. The reagents supplied from commercial sources were used without further purification. TLC chromatography was performed on precoated aluminium silica gel SIL G/UV254 plates (Macherey-Nagel & Co.) and the detection occurred via fluorescence quenching, development in a molybdato phosphate solution (10% in EtOH). The compounds were purified via column chromatography and the hybrid compounds were reprecipitated from $CH_2Cl_2$ in *n*-hexane to obtain a pure compound for elemental analysis and further biological tests. All compounds were dried in high-vacuum ($10^{-3}$ mbar). $^{1}H$ NMR and $^{13}C$ NMR spectra were recorded at room temperature on a Bruker Avance or JEOL JNM GX 400 spectrometer operating at 300, 400 or 600 MHz, 75, 100 or 125 MHz. All chemical shifts are given in the ppm-scale and refer to the nondeuterized proportion of the solvent. ESI, APPI and MALDI mass spectra were recorded on a Bruker Daltonik maXis 4G or Bruker Daltonik micrOTOF II focus. Elemental analysis (C, H, N) were carried out with an Euro EA 3000 (EuroVector) machine and an Elementar vario MICRO cube machine and calculated values confirm a purity of > 95% for the all biologically tested compounds. Artesunic acid and dihydroartemisinin were purchased from ABCR (Karlsruhe, Germany) and TCI (Deutschland GmbH). 4,7-dichloroquinoline was purchased from Sigma Aldrich (Germany). Experimental details and the spectra of the hybrids and their precursors can be found following in the supporting information.

## 2. Synthesis and characterization of compounds

**The general synthesis route of compounds 18 and 19 via Cobalt−Catalyzed C−H/N−O Functionalization:**

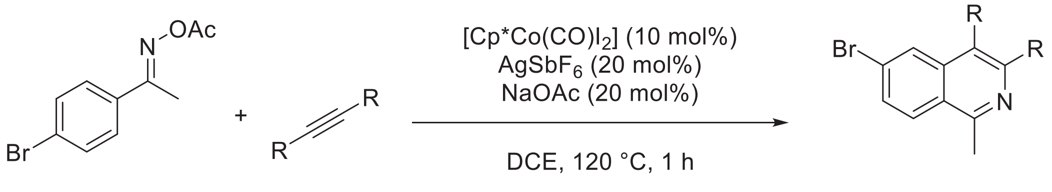


**Scheme S1.** General presentation of precursor synthesis.

A suspension of *O*-acetyl oxime (0.50 mmol, 1.00 equiv), alkyne (0.75 mmol, 1.50 equiv), $Cp^*CoI_2(CO)$ (0.05 mmol, 10 mol %), $AgSbF_6$ (0.1 mmol, 20 mol %) and NaOAc (0.1 mmol, 20 mol %) in DCE (0.25 M) was stirred at 120 °C for 1 h under air. After cooling to ambient temperature, the solvent was evaporated in vacuo and the remaining residue was purified by column chromatography on silica gel to afford the desired products **18**/**19**.[1]

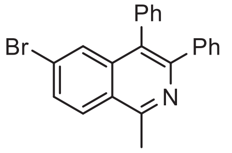


**Compound 18:** The general procedure for compound **18** was followed using (*E*)-1-(4-bromophenyl) ethanone *O*-acetyl oxime (128 mg, 0.50 mmol) and diphenylacetylene (134 mg, 0.75 mmol). Purification by column chromatography on silica gel (*n*-hexane/EtOAc: 30/1 → 20/1 → 10/1) yielded compound **18** (150 mg, 80%) as a slightly yellow solid.

$^1$H NMR (300 MHz, $CDCl_3$) $\delta$: 8.08 (dd, $J$ = 8.9, 0.5 Hz, 1H), 7.83 (dd, $J$ = 2.0, 0.5 Hz, 1H), 7.69 (dd, $J$ = 8.9, 2.0 Hz, 1H), 7.39 – 7.34 (m, 5H), 7.24 – 7.18 (m, 5H), 3.07 (s, 3H). $^{13}$C NMR (125 MHz, $CDCl_3$) $\delta$: 157.8, 150.6, 140.6, 137.4, 136.8, 131.3, 130.2, 130.0, 128.4, 128.4, 128.3, 127.7, 127.5, 127.3, 127.2, 125.0, 124.6, 22.7. MS (ESI) *m/z* (relative intensity) 376 (100) $[M+H]^+$ ($^{81}$Br), 374 (100) $[M+H^+]$ ($^{79}$Br). HRMS (ESI+) *m/z* calculated for $C_{22}H_{17}BrN$ $[M+H]^+$ ($^{79}$Br): 374.0539, found: 374.0534. Elemental analysis; calculated for $C_{22}H_{16}BrN$: C: 70.60 H: 4.31 N: 3.74 found: C: 70.17 H: 4.35 N: 3.77.

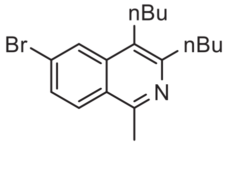


**Compound 19:** The general procedure for compound **19** was followed using (*E*)-1-(4-bromophenyl) ethanone *O*-acetyl oxime (128 mg, 0.50 mmol) and dec-5-yne (104 mg, 0.75 mmol). Purification by column chromatography on silica gel (*n*-hexane/EtOAc: 40/1 → 20/1) yielded compound **19** (142 mg, 85%) as a yellow solid. M.p. = 48−50 °C.

$^1$H NMR (600 MHz, $CDCl_3$) $\delta$: 8.07 (d, $J$ = 1.9 Hz, 1H), 7.91 (d, $J$ = 8.9 Hz, 1H), 7.57 (dd, $J$ = 8.9, 1.9 Hz, 1H), 2.92 – 2.87 (m, 4H), 2.86 (s, 3H), 1.72 – 1.67 (m, 2H), 1.60 – 1.53 (m, 2H), 1.52 – 1.48 (m, 2H), 1.47 – 1.43 (m, 2H), 0.99 (t, $J$ = 7.3 Hz, 3H), 0.95 (t, $J$ = 7.3 Hz, 3H). $^{13}$C NMR (125 MHz, $CDCl_3$) $\delta$: 155.5, 153.1, 136.7, 128.6, 127.8, 125.9, 125.3, 124.4, 124.3, 35.3, 33.1, 32.7, 27.4, 23.2, 23.1, 22.4, 14., 14.0. MS (ESI) *m/z* (relative intensity) 336 (100) $[M+H]^+$ ($^{81}$Br), 334 (100) $[M+H]^+$ ($^{79}$Br). HRMS (ESI+) *m/z* calculated for $C_{18}H_{25}BrN$ $[M+H]^+$ ($^{79}$Br): 334.1165, found: 334.1160.

**The general synthesis route of compounds 20 and 21:** A suspension of **18** or **19** (1.0 equiv.), trimethylsilylacetylene (1.9 equiv.), $PdCl_2(PPh_3)_2$ (1.0 mol %), CuI (5.0 mol %) in $NEt_3$ (0.1 M) was stirred at 50 °C for 2 h under $N_2$. After cooling to ambient temperature, the solvent was evaporated *in vacuo*. The residue was dissolved in EtOAc, filtered through a plug of celite and concentrated *in vacuo*. The remaining solid was dissolved in MeOH (0.1 M). $K_2CO_3$ (2.0 equiv.) was added and the resulting suspension was stirred at 25 °C for 1 h. The solvent was evaporated *in vacuo* and the remaining residue was purified by column chromatography on silica gel to afford the desired products **20** or **21**.

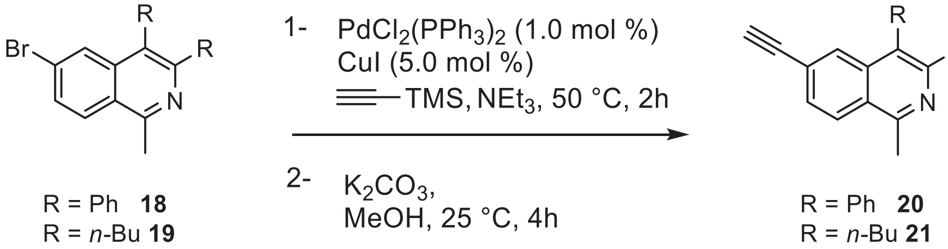


**Scheme S2.** General presentation of precursor synthesis.

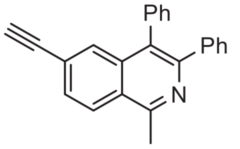


**Compound 20:** 6-Bromo-1-methyl-3,4-diphenylisoquinoline (**18**) (580 mg, 1.55 mmol). Purification by column chromatography on silica gel (*n*-hexane/EtOAc: 10/1) yielded compound **20** (440 mg, 89%) as a pale-yellow solid. M.p. = 200−202 °C.

$^{1}$H NMR (300 MHz, $CDCl_3$) $\delta$: 8.13 (dd, $J$ = 8.6, 0.7 Hz, 1H), 7.81 (dd, $J$ = 1.6, 0.7 Hz, 1H), 7.61 (dd, $J$ = 8.6, 1.6 Hz, 1H), 7.38 – 7.30 (m, 5H), 7.22 – 7.15 (m, 5H), 3.15 (s, 1H), 3.05 (s, 3H). $^{13}$C NMR (125 MHz, $CDCl_3$) $\delta$: 157.5, 150.2, 140.6, 136.9, 135.6, 131.2, 130.3, 130.1, 129.1, 128.7, 128.2, 127.5, 127.2, 127.0, 125.6, 125.3, 123.6, 83.4, 79.3, 22.8. MS (ESI) *m/z* (relative intensity) 320 (100) $[M+H]^+$. HRMS (ESI+) *m/z* calculated for $C_{24}H_{18}N$ $[M+H]^+$: 320.1434, found: 320.1431.

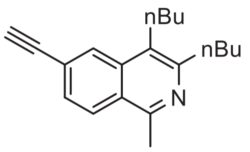


**Compound 21:** 6-Bromo-3,4-dibutyl-1-methylisoquinoline (**19**) (457 mg, 1.30 mmol). Purification by column chromatography on silica gel (*n*-hexane/EtOAc: 10/1) yielded compound **21** (345 mg, 95%) as a pale-yellow solid. M.p. = 84−86 °C.

$^{1}$H NMR (500 MHz, $CDCl_3$) $\delta$: 8.09 (d, $J$ = 1.5 Hz, 1H), 7.99 (d, $J$ = 8.6 Hz, 1H), 7.51 (dd, $J$ = 8.6, 1.5 Hz, 1H), 3.21 (s, 1H), 2.96 – 2.89 (m, 4H), 2.86 (s, 3H), 1.72 – 1.67 (m, 2H), 1.60 – 1.55 (m, 2H), 1.52 – 1.41 (m, 4H), 0.99 (t, $J$ = 7.3 Hz, 3H), 0.95 (t, $J$ = 7.3 Hz, 3H). $^{13}$C NMR (125 MHz, $CDCl_3$) $\delta$: 155.5, 152.8, 134.9, 127.9, 127.8, 126.2, 125.9, 125.2, 123.0, 83.8, 78.7, 35.2, 33.1, 32.8, 27.3, 23.2, 23.1, 22.3, 14.1, 14.0. MS (ESI) *m/z* (relative intensity) 280 (100) $[M+H]^+$. HRMS (ESI+) *m/z* calculated for $C_{20}H_{26}N$ $[M+H]^+$: 280.2060, found: 280.2055.

**The general synthesis route of hybrid 1-3**: A solution of compound **20** or **21** (1.0 equiv.), compound **22** (1.2 equiv.), $CuSO_4$ (5.0 mol %) and sodium (L)-ascorbate (10 mol %) in a 1/1 mixture of $CH_2Cl_2/H_2O$ was stirred under nitrogen at 25 °C for 16 h. The solvent was evaporated *in vacuo* and the remaining residue was purified by column chromatography on silica gel and HPLC yielded **1** and **2**.

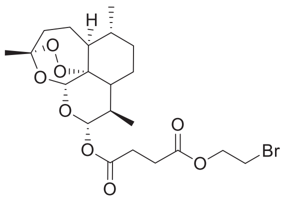

**Compound 22**: First step: At 0 °C, 2-bromoethanol (0.43 mL, 6 mmol, 1.0 equiv.) was added dropwise to a solution of artesunic acid (**35**) (3.0 g, 7.8 mmol, 1.3 equiv.), DCC (1.6g, 7.8 mmol, 1.3 equiv.) and DMAP (360 mg, 3 mmol, 50 mol %) in $CH_2Cl_2$ (20 mL). The reaction mixture was stirred at 25 °C for 16 h and extracted with $CH_2Cl_2$. Purification by column chromatography (EtOAc/*n*-hexane: 4/1) afforded the product (2.54 g, 86%) as a white solid. M.p. = 84−86 °C.

$^1$H NMR (300 MHz, $CDCl_3$) $\delta$: 5.79 (d, $J$ = 9.9 Hz, 1H), 5.42 (s, 1H), 4.39 (t, $J$ = 6.2 Hz, 2H), 3.50 (t, $J$ = 6.2 Hz, 2H), 2.76 – 2.64 (m, 4H), 2.61 – 2.52 (m, 1H), 2.42 – 2.31 (m 1H), 2.07 – 1.97 (m, 1H), 1.94 – 1.83 (m, 1H), 1.81 – 1.67 (m, 2H), 1.65 – 1.57 (m, 1H), 1.55 – 1.45 (m, 1H), 1.43 (s, 3H), 1.40 – 1.25 (m, 3H), 1.08 – 0.99 (m, 1H), 0.95 (d, $J$ = 5.9 Hz, 3H), 0.85 (d, $J$ = 7.0 Hz, 3H). $^{13}$C NMR (125 MHz, $CDCl_3$) $\delta$: 171.6, 170.9, 104.7, 92.4, 91.4, 80.2, 63.4, 51.4, 45.4, 37.2, 36.1, 33.9, 31.9, 29.1, 28.8, 28.5, 26.1, 24.9, 21.8, 20.3, 12.6.

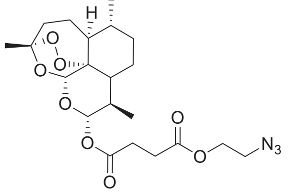

Second step: $NaN_3$ (1.0 g, 15.5 mmol, 3.0 equiv.) was added portion wise to a solution of the product which was obtained in the first step (2.54 g, 5.18 mmol, 1.0 equiv.), and NaI (39 mg, 0.26 mmol, 5.0 mol %) in DMF (20 mL). The reaction mixture was stirred at 60°C for 16 h and extracted with $CH_2Cl_2$. The organic layer was dried over $Na_2SO_4$ and evaporated *in vacuo* to afford product **22** (2.28 g, 97%) as a yellow oil.

$^1$H NMR (300 MHz, $CDCl_3$) $\delta$: 5.80 (d, $J$ = 9.9 Hz, 1H), 5.43 (s, 1H), 4.28 – 4.24 (m, 2H), 3.50 – 3.47 (m, 2H), 2.81 – 2.67 (m, 4H), 2.66 – 2.55 (m, 1H), 2.43 – 2.22 (m, 1H), 2.15 – 1.99 (m, 2H), 1.94 – 1.86 (m, 1H), 1.81 – 1.70 (m, 2H), 1.68 – 1.59 (m, 1H), 1.56 – 1.48 (m, 1H), 1.45 (s, 3H), 1.43 – 1.26 (m, 2H), 1.13 – 1.03 (m, 1H), 0.96 (d, $J$ = 5.9 Hz, 3H), 0.87 (d, $J$ = 7.1 Hz, 3H). $^{13}$C NMR (125 MHz, $CDCl_3$) $\delta$: 171.6, 170.9, 104.4, 92.1, 91.4, 80.1, 63.1, 51.5, 49.7, 49.2, 37.3, 36.2, 34.1, 31.9, 29.1, 28.8, 26.0, 24.6, 22.0, 20.2, 12.1. MS (ESI) *m/z* (relative intensity): 929 (66) [2M+Na$^+$], 476 (100) [M+Na]$^+$. HRMS (ESI+) *m/z* calculated for $C_{21}H_{31}N_3O_8Na$ [M+Na$^+$]: 476.2003, found: 476.1993.

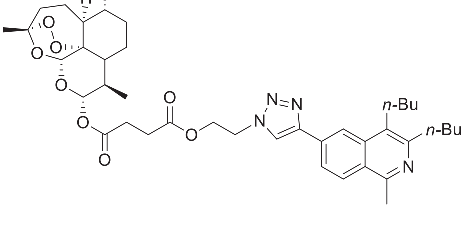

**Hybrid 1:** The general procedure hybrid **1** was followed using **21** (139 mg, 0.50 mmol). Purification by column chromatography on silica gel (*n*-hexane/EtOAc: 3/2) and HPLC yielded hybrid **1** (80 mg, 22%) as a slightly yellow oil.

$^1$H NMR (300 MHz, $CDCl_3$) $\delta$: 8.59 (d, $J$ = 1.5 Hz, 1H), 8.26 (s, 1H), 8.14 (d, $J$ = 8.7 Hz, 1H), 8.03 (dd, $J$ = 8.7, 1.5 Hz, 1H), 5.67 (d, $J$ = 9.9 Hz, 1H), 5.16 (s, 1H), 4.83 – 4.70 (m, 2H), 4.62 – 4.54 (m, 2H), 3.18 – 3.04 (m, 2H), 2.98 – 2.90 (m, 5H), 2.77 – 2.67 (m, 4H), 2.57 – 2.48 (m, 1H), 2.39 – 2.27 (m, 1H), 2.03 – 1.92 (m, 1H), 1.87 – 1.62 (m, 6H), 1.61 – 1.43 (m, 7H), 1.35 (s, 3H), 1.23 – 1.14 (m, 1H), 1.05 – 1.00 (m, 6H), 0.91 – 0.81 (m, 6H), 0.74 (d, $J$ = 7.1 Hz, 3H). $^{13}$C NMR (75 MHz, $CDCl_3$) $\delta$: 171.3, 171.2, 155.3, 152.6, 147.5, 135.8, 131.5, 126.8, 125.5, 123.5, 122.2, 120.0, 104.4, 92.4, 91.4, 80.0, 63.2, 51.4, 49.1, 45.0, 37.0, 36.2, 35.4, 35.3, 34.0, 33.3, 32.9, 31.6, 29.5, 29.2, 29.1, 27.3, 25.8,

24.5, 23.2, 22.3, 21.7, 20.0, 14.1, 14.0, 11.9. MS (ESI) *m/z* (relative intensity) 755 (18) $[M+Na]^+$, 733 (100) $[M+H]^+$. HRMS (ESI+) *m/z* calculated for $C_{41}H_{57}N_4O_8$ $[M+H]^+$: 733.4171, found: 733.4164. Elemental analysis; calculated for $C_{41}H_{56}N_4O_8$: C: 67.19 H: 7.70 N: 7.64 found: C: 66.85 H: 7.55 N: 7.43.

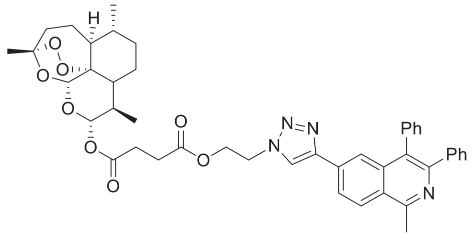


**Hybrid 2:** The general procedure hybrid **2** was followed using **20** (154 mg, 0.50 mmol). Purification by column chromatography on silica gel (*n*-hexane/EtOAc: 3/2) and HPLC yielded hybrid **2** (100 mg, 26%) as a slightly yellow oil.

$^1$H NMR (300 MHz, $CDCl_3$) $\delta$: 8.28 (d, *J* = 9.1 Hz, 1H), 8.17 (dd, *J* = 9.1, 1.7 Hz, 1H), 8.17 (d, *J* = 1.7 Hz, 1H), 8.02 (s, 1H), 7.38 – 7.29 (m, 6H), 7.24 – 7.15 (m, 4H), 5.68 (d, *J* = 9.9 Hz, 1H), 5.28 (s, 1H), 4.68 – 4.65 (m, 2H), 4.56 – 4.48 (m, 2H), 3.09 (s, 3H), 2.70 – 2.63 (m, 4H), 2.59 – 2.47 (m, 1H), 2.39 – 2.28 (m, 1H), 2.04 – 1.95 (m, 1H), 1.90 – 1.81 (m, 1H), 1.72 – 1.52 (m, 3H), 1.46 – 1.38 (m, 1H), 1.34 (s, 3H), 1.28 – 1.03 (m, 4H), 0.91 (d, *J* = 5.9 Hz, 3H), 0.78 (d, *J* = 7.1 Hz, 3H). $^{13}$C NMR (75 MHz, $CDCl_3$) $\delta$: 171.3, 171.1, 157.5, 150.2, 147.2, 141.0, 137.3, 136.3, 132.1, 131.5, 130.3, 129.4, 128.4, 127.6, 127.2, 126.9, 126.3, 125.7, 124.8, 122.5, 122.2, 104.4, 92.4, 91.4, 80.2, 62.9, 51.4, 49.1, 45.1, 37.2, 36.2, 34.0, 31.7, 29.2, 28.9, 25.8, 24.5, 22.7, 21.9, 20.2, 12.0. MS (ESI) *m/z* (relative intensity) 795 (8) $[M+Na]^+$, 773 (100) $[M+H]^+$. HRMS (ESI+) *m/z* calculated for $C_{45}H_{49}N_4O_8$ $[M+H]^+$: 773.3545, found: 773.3544. Elemental analysis; calculated for $C_{45}H_{48}N_4O_8$: C: 69.93 H: 6.26 N: 7.25 found: C: 70.17 H: 6.31 N: 7.18.

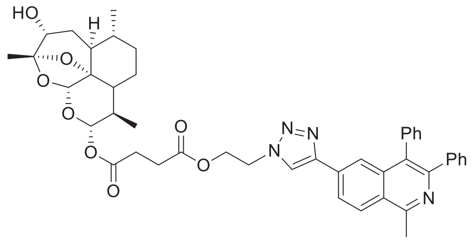


**Hybrid 3:** Hybrid **3** was observed as by-product of hybrid **2**.

$^1$H NMR (300 MHz, $CDCl_3$) $\delta$: 8.28 (d, *J* = 8.7 Hz, 1H), 8.17 (dd, *J* = 8.7, 1.7 Hz, 1H), 8.10 (d, *J* = 1.7 Hz, 1H), 8.05 (s, 1H), 7.90 (s, 1H), 7.42 – 7.32 (m, 5H), 7.28 – 7.17 (m, 5H), 6.94 (d, *J* = 5.1 Hz, 1H), 4.69 – 4.62 (m, 2H), 4.56 – 4.48 (m, 2H), 3.09 (s, 3H), 2.71 – 2.56 (m, 4H), 2.54 – 2.47 (m, 1H), 2.39 – 2.28 (m, 2H), 2.23 – 2.16 (m, 1H), 2.10 (s, 3H), 2.07 – 1.96 (m, 2H), 1.90 – 1.70 (m, 3H), 1.59 – 1.42 (m, 4H), 1.06 (d, *J* = 5.9 Hz, 3H), 1.03 (d, *J* = 7.1 Hz, 3H). $^{13}$C NMR (75 MHz, $CDCl_3$) $\delta$: 211.9, 209.1, 171.4, 170.6, 159.3, 157.6, 150.2, 147.4, 140.9, 137.4, 136.3, 131.9, 131.5, 130.2, 129.3, 128.3, 127.6, 127.2, 126.9, 126.5, 125.7, 124.6, 122.5, 121.5, 91.7, 62.9, 57.0, 51.8, 49.1, 41.1, 40.7, 36.5, 34.6, 31.0, 29.9, 28.8, 28.5, 22.7, 20.5, 20.0, 11.5. MS (ESI) *m/z* (relative intensity) 795 (7) $[M+Na^+]$, 773 (100) $[M+H]^+$. HRMS (ESI+) *m/z* calculated for $C_{45}H_{49}N_4O_8$ $[M+H^+]$: 773.3545, found: 773.3544. Elemental analysis; calculated for $C_{45}H_{48}N_4O_8$: C: 69.93 H: 6.26 N: 7.25 found: C: 70.27 H: 6.49 N: 7.27.

**Synthesis and characterization of 7-chloroquinoline derivatives and related hybrids:**

All 7-chloroquinoline derivatives (**27**, **33**, **34**, and **36**), except compound **37**, were prepared according to literature procedure.[2,3] Spectral data of these known 7-chloroquinoline derivatives match literature values.

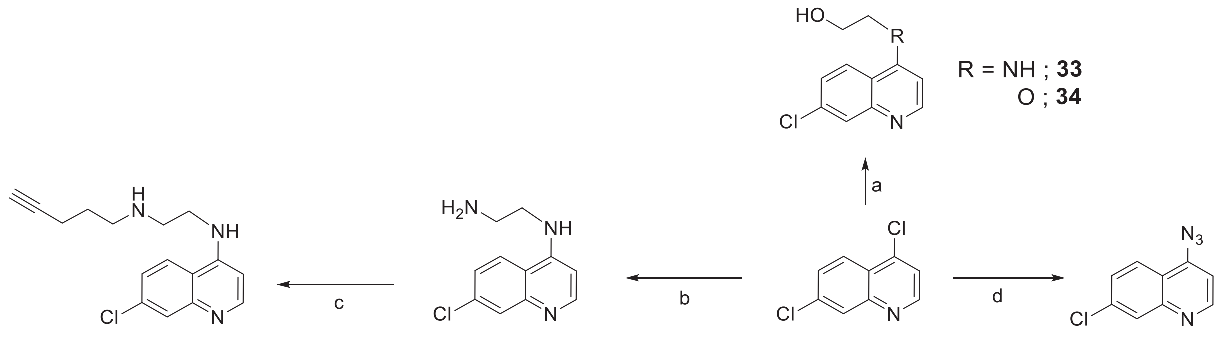


**37** **36** 4,7-dicholoroquinoline **27**

**Scheme S3.** General presentation of precursor synthesis. a) **34**; Ethanediol, *t*-BuOK/*t*-BuOH, 80 ºC, 18h, **33**; Ethanolamine, $Et_3N$, 120 ºC, 2h b) **36**; 1,2-Diaminoethane, 80 ºC-135 ºC c) **37**; 5-chloropent-1-yne, $K_2CO_3$, 130 ºC d) **37**; $NaN_3$, DMF, 100ºC, 3h.

**Elemental Analysis of 7-chloroquinoline derivatives:**

**Compound 33**: Elemental analysis; calculated for $C_{11}H_{11}ClN_2O$ C: 59.33 H: 4.98 N: 12.58 Found: C: 58.86 H: 4.93 N: 12.57.

**Compound 36**: Elemental analysis; calculated for $C_{11}H_{12}ClN_3$ C: 59.60 H: 5.46 N: 18.96 Found: C: 59.57 H: 5.40 N: 18.74.

**Compound 37**: Elemental analysis; calculated for $C_{16}H_{18}ClN_3$ C: 66.78 H: 6.30 N: 14.60 Found: C: 66.47 H: 6.31 N: 14.37.

**Compound 27**: Elemental analysis; calculated for $C_9H_5ClN_4$ C: 52.83 H: 2.46 N: 27.38 Found: C: 52.85 H: 2.62 N: 27.40.

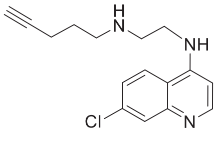


**Compound 37:** Compound **36** was dissolved in acetonitrile, 5-chloropent-1-yne and $K_2CO_3$ were added. The reaction mixture was refluxed at 130 ºC for overnight. After filtration, the solvent and the crude product was purified via column chromatography ($SiO_2$, $CH_2Cl_2$/MeOH 8:2) 18%, white powder.

$^1$H NMR (300 MHz, $CDCl_3$) $\delta$: 5.51 (m, 2H), 5.73 (t, $J$ = 2.6 Hz, 1H), 6.09 (td, $J$ = 6.9, 2.7 Hz, 2H), 6.61 (t, $J$ = 7.0 Hz, 2H), 6.84 (t, $J$ = 7.0 Hz, 2H), 7.15 (t, $J$ = 7.0 Hz, 2H), 10.10 (d, $J$ = 5.5 Hz, 1H), 11.09 (dd, $J$ = 8.9, 2.2 Hz, 1H), 11.59 (d, $J$ = 8.9 Hz, 1H), 11.65 (d, $J$ = 2.1 Hz, 1H), 12.20 (d, $J$ = 5.5 Hz, 1H). $^{13}$C NMR (75 MHz, $CDCl_3$) $\delta$: 16.36, 28.52, 42.01, 47.47, 48.00, 69.07, 83.80, 99.26, 117.46, 121.58, 125.47, 128.53, 135.10, 148.94, 150.15, 151.91 ppm. HRMS (ESI+) $m/z$ calculated for $C_{16}H_{19}ClN_3$ $[M+H]^+$: 288.1262; found: 288.1264. Elemental analysis; calculated for $C_{16}H_{18}ClN_3$ C: 66.78 H: 6.30 N: 14.60 Found: C: 66.47 H: 6.31 N: 14.37.

**The general synthesis route of compounds 23-26.** To a solution of artesunic acid (**35**), EDCI and DMAP in $CH_2Cl_2$ at 0 ºC, alcohol derivatives were added. The reaction mixture was warm up to room temperature and stirred 18-20 h, was then quenched with water. The mixture was extracted with $CH_2Cl_2$, dried over $Na_2SO_4$, and concentrated *in vacuo*. The crude compound was purified by column chromatography ($SiO_2$, Hexane/EtOAc) to give a colorless oil.

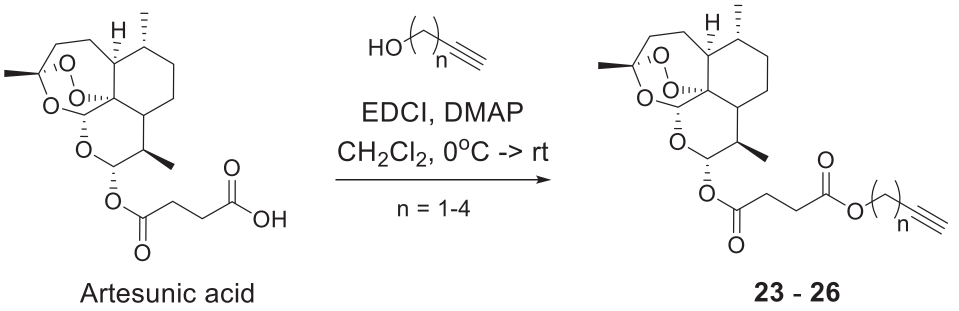


**Scheme S4.** General presentation of ester-linked artesunic acid- alkyne synthesis.

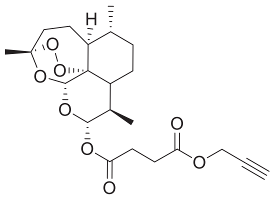

**Compound 23:** Artesunic acid (150 mg, 0.39 mmol, 1 equiv.), propargyl alcohol (45.45 µL, 0.78 mmol, 2 equiv.), $CH_2Cl_2$ (9 mL), EDCI (150 mg, 0.96 mmol, 1 equiv.), *N*,*N*-Diisopropylethylamine (15µl, 0.2 $10^{-3}$ mmol). Column chromatography conditions: Hexane/EtOAc 7/3, $R_f$: 0.62. Yield: 97%.

$^1$H NMR (300 MHz, $CDCl_3$) $\delta$: 0.80 (d, $J$ = 7.1 Hz, 3H), 0.91 (d, $J$ = 5.8 Hz, 3H), 0.99 (m, 1H), 1.16 – 1.36 (m, 4H), 1.38 (s, 3H), 1.44 – 1.76 (m, 4H), 1.82 – 1.89 (m, 1H), 1.97 – 2.02 (m, 1H), 2.28 – 2.38 (m, 1H), 2.44 – 2.45 (m, 1H), 2.48 – 2.58 (m, 1H), 2.60 – 2.74 (m, 4H), 4.59 – 4.71 (m, 2H), 5.39 (s, 1H), 5.77 (d, $J$ = 9.8, 1H) ppm. $^{13}$C NMR (75 MHz, $CDCl_3$) $\delta$: 12.13, 20.30, 22.06, 24.65, 26.02, 28.72, 29.11, 31.86, 34.15, 36.28, 37.34, 45.30, 51.61, 52.30, 75.14, 77.54, 80.17, 91.57, 92.31, 104.54, 171.00, 171.41 ppm.

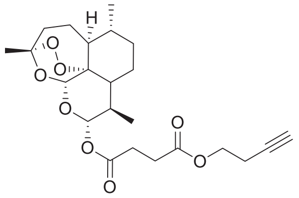

**Compound 24:** Artesunic acid (150 mg, 0.39 mmol, 1 equiv.), 3-*butyn*-1-ol (120 µL, 0.78 mmol, 55 mg, 4 equiv.), $CH_2Cl_2$ (6 mL), EDCI (150 mg, 0.96 mmol, 2.5 equiv.), DMAP (48 mg, 0.39 mmol, 1 equiv.). Column chromatography conditions: Hexane/EtOAc 7/3, $R_f$: 0.56. Yield: 88%.

$^1$H NMR (300 MHz, $CDCl_3$) $\delta$: 0.81 (d, $J$ = 7.1 Hz, 3H), 0.92 (d, $J$ = 5.8 Hz, 3H), 0.98 – 1.02 (m, 1H), 1.26 – 1.32 (m, 3H), 1.38 (s, 3H), 1.44 – 1.89 (m, 4H), 1.89 – 1.95 (m, 1H), 1.97 – 2.00 (m, 2H), 2.33 (td, $J$ = 13.9, 3.9 Hz, 1H), 2.46 – 2.72 (m, 7H), 4.15 (t, $J$ = 6.9, 2H), 5.39 (s, 1H), 5.75 (d, $J$ = 9.8, 1H) ppm; $^{13}$C NMR (75 MHz, $CDCl_3$) $\delta$: 12.08, 14.24, 18.94, 21.08, 22.02, 24.62, 25.98, 28.82, 29.19, 31.83, 34.12, 36.25, 37.29, 45.26, 51.59, 62.39, 70.08, 80.13, 91.52, 92.20, 104.47, 171.06, 171.84 ppm. HRMS (ESI+) *m/z* calculated for $C_{23}H_{32}NaO_8$ $[M+Na]^+$: 459.19891; found: 459.19894.

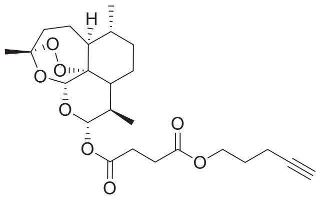

**Compound 25:** Artesunic acid (150 mg, 0.39 mmol, 1 equiv.), 4-*pentyn*-1-ol (144 µL, 1.56 mmol, 131 mg, 4 equiv.), $CH_2Cl_2$ (6 mL), EDCI (150 mg, 0.96 mmol, 2.5 equiv.), DMAP (48 mg, 0.39 mmol, 1 equiv.). Column chromatography conditions: Hexane/EtOAc 7/3, $R_f$: 0.62. Yield: 92%.

$^1$H NMR (300 MHz, $CDCl_3$) $\delta$: 0.81 (d, $J$ = 7.1 Hz, 3H), 0.92 (d, $J$ = 5.8 Hz, 3H), 1.00 (d, $J$ = 9.6 Hz, 1H), 1.18 – 1.25 (m, 2H), 1.27 – 1.33 (m, 2H), 1.40 (s, 3H), 1.55 – 1.87 (m, 6H), 1.93 – 1.95 (t, 1H), 2.01 (s, 1H), 2.23 – 2.47 (m, 3H), 2.47 – 2.73 (m, 5H), 4.14 – 4.19 (t, 2H), 5.40 (s, 1H), 5.74 – 5.77 (d, $J$ = 9.9, 1H) ppm. $^{13}$C NMR (75 MHz, $CDCl_3$) $\delta$: 12.09, 15.21, 20.26, 22.03, 24.62, 25.99, 27.51, 28.88, 29.23, 31.83, 34.12, 36.25, 37.30, 45.27, 51.59, 63.28, 69.10, 80.14, 83.04, 91.53, 92.19, 104.48, 171.13, 172.04 ppm. HRMS (ESI+) *m/z* calculated for $C_{24}H_{34}NaO_8$ [M+Na]$^+$: 473.2148; found: 473.2146.

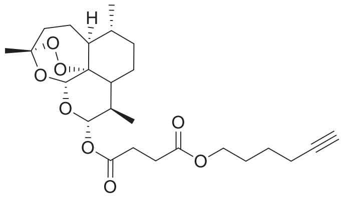

**Compound 26:** Artesunic acid (150 mg, 0.39 mmol, 1 equiv.), 5-hexyn-1-ol (87 µL, 0.78 mmol, 77 mg, 2 equiv.), $CH_2Cl_2$ (6 mL), EDCI (150 mg, 0.96 mmol, 2.5 equiv.), DMAP (48 mg, 0.39 mmol, 1 equiv.). Column chromatography conditions: Hexane/EtOAc 7/3, $R_f$: 0.73. Yield: 99%.

$^1$H NMR (300 MHz, $CDCl_3$) $\delta$: 0.77 (d, $J$ = 7.1 Hz, 3H), 0.88 (d, $J$ = 5.8 Hz, 3H), 0.95 – 0.99 (m, 1H), 1.17 – 1.28 (m, 3H), 1.34 (s, 3H), 1.46 – 1.72 (m, 8H), 1.78 – 1.97 (m, 3H), 2.14 (td, $J$ = 6.9, 2.6 Hz, 2H), 2.28 (td, $J$ = 14.1, 3.7 Hz, 1H) 2.41 – 2.67 (m, 5H), 4.03 (t, $J$ = 6.4 Hz, 2H), 5.35 (s, 1H), 5.70 (d, $J$ = 9.8, 1H) ppm. $^{13}$C NMR (75 MHz, $CDCl_3$) $\delta$: 171.94, 170.97, 104.28, 92.01, 91.34, 83.72, 79.96, 68.78, 64.06, 51.43, 45.11, 37.12, 36.10, 33.98, 31.68, 29.08, 28.75, 27.46, 25.82, 24.73, 24.48, 21.86, 20.13, 17.90, 11.94.ppm. HRMS (ESI+) *m/z* calculated for $C_{25}H_{36}NaO_8$ [M+H]$^+$: 487.2303; found: 487.2302.

**The general synthesis route of hybrids 4-7:** The alkyne derived artesunic acid (160 mg, 0.379 mmol, 1 equiv.) and 4-azido-7-chloroquinoline (**27**) (155 mg, 0.758 mmol, 2 equiv.) were dissolved in $CH_2Cl_2$ (7.5 mL). The solution of $CuSO_4 \cdot 5H_2O$ (19 mg, 0.0758 mmol, 20 mol %) and L-ascorbic acid (30 mg, 0.152 mmol, 40 mol %) in $H_2O$ (7.5 mL) was then added to the reaction mixture. After stirring overnight, the crude was extracted with $CH_2Cl_2$, dried over $Na_2SO_4$, and concentrated *in vacuo*. The product was isolated via column chromatography ($SiO_2$, Hexane/EtOAc: 1/1).

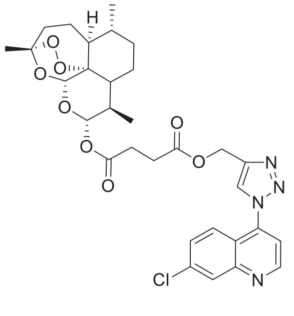

**Hybrid 4:** Compound **23** (160 mg, 0.379 mmol,1 equiv.), 4-azido-7-chloroquinoline (**27**) (155 mg, 0.758 mmol, 2 equiv.), $CH_2Cl_2$ (7.5 mL), $CuSO_4 \cdot 5H_2O$ (19 mg, 0.0758, 20 mol %), L-ascorbic acid (30 mg, 0.152 mmol, 40 mol %), $H_2O$ (7.5 mL). Yield: 52%.

$^1$H NMR (300 MHz, $CDCl_3$) $\delta$: 0.78 (d, $J$ = 7.1 Hz, 3H), 0.92 (d, $J$ = 5.4 Hz, 3H), 1.12 – 1.16 (m, 4H), 1.29 (s, 3H), 1.49 – 1.67 (m, 4H), 1.80 – 1.94 (m, 2H), 2.23 – 2.33 (m, 1H), 2.43 – 2.55 (m, 1H), 2.68 – 2.77 (m, 4H), 5.04 (s, 1H), 5.35 – 5.48 (m, 2H), 5.62 (d, $J$ = 9.9 Hz, 1H), 7.53 (d, $J$ = 4.6 Hz, 1H), 7.60 (dd, $J$ = 9.1 Hz, 1H), 8.00 (d, $J$ = 9.1, 1H), 8.19 (s, 1H), 8.22 (d, $J$ = 2.1 Hz, 1H), 9.05 (d, $J$ = 4.6 Hz, 1H) ppm. $^{13}$C NMR (75 MHz, $CDCl_3$) $\delta$: 12.14, 20.25, 22.00, 24.57, 25.91,

29.00, 29.25, 31.77, 34.10, 36.20, 37.32, 45.15, 51.49, 57.99, 80.07, 91.41, 92.39, 104.46, 116.34, 120.75, 124.96, 125.82, 128.98, 129.51, 136.99, 141.16, 144.01, 150.20, 151.51, 171.33, 171.93 ppm. HRMS (ESI+) *m/z* calculated for $C_{31}H_{35}ClN_4NaO_8$ [M+Na]$^+$: 649.2049; found: 649.2036. Elemental analysis; calculated for: $C_{31}H_{35}ClN_4O_8$: C: 59.38 H: 5.63 N: 8.93 found: C: 59.77 H: 5.80 N: 9.04.

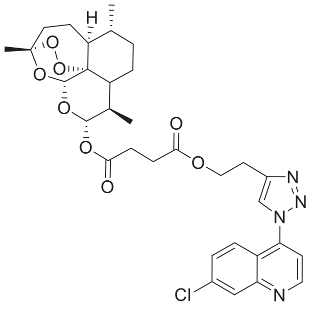

**Hybrid 5:** Compound **24** (140 mg, 0.321 mmol, 1 equiv.), 4-azido-7-chloroquinoline (**27**) (132 mg, 0.641 mmol, 2 equiv.), $CH_2Cl_2$ (5 mL), $CuSO_4{\cdot}5H_2O$ (16 mg, 0.0642 mmol, 20 mol %), L-ascorbic acid (25.5 mg, 0.128 mmol, 40 mol %), $H_2O$ (5 mL). Yield: 29%.

$^1$H NMR (300 MHz, CDCl$_3$) $\delta$: 0.77 (d, *J* = 7.1 Hz, 3H), 0.92 (d, *J* = 5.2 Hz, 3H), 1.14 – 1.23 (m, 4H), 1.30 (s, 3H), 1.49 – 1.96 (m, 4H), 1.79 – 1.96 (m, 2H), 2.23 – 2.34 (m, 1H), 2.39 – 2.51 (m, 1H), 2.62 – 2.70 (m, 4H), 3.23 (t, *J* = 6.1 Hz, 2H), 4.39 – 4.56 (m, 2H), 5.14 (s, 1H), 5.60 (d, *J* = 9.9 Hz, 1H), 7.57 (d, *J* = 4.6 Hz, 1H), 7.61 (d, *J* = 2.2 Hz, 1H), 8.08 (d, *J* = 3.6 Hz, 1H), 8.11 (s, 1H), 8.20 (d, *J* = 2.2 Hz, 1H), 9.04 (d, *J* = 4.7 Hz, 1H) ppm. $^{13}$C NMR (75 MHz, CDCl$_3$) $\delta$: 12.08, 20.25, 21.99, 24.54, 25.47, 25.91, 29.05, 29.20, 31.76, 34.05, 36.22, 37.28, 45.14, 51.50, 63.40, 80.09, 91.44, 92.34, 104.46, 116.11, 120.75, 124.22, 125.22, 128.90, 129.34, 136.83, 141.26, 145.08, 150.24, 151.54, 171.31, 171.84 ppm. HRMS (ESI+) *m/z* calculated for $C_{32}H_{37}ClN_4NaO_8$ [M+Na]$^+$: 663.2207; found: 663.2192. Elemental analysis; calculated for $C_{32}H_{37}ClN_4O_8$: C: 59.95 H: 5.82 N: 8.74 found: C: 60.14 H: 6.19 N: 8.21.

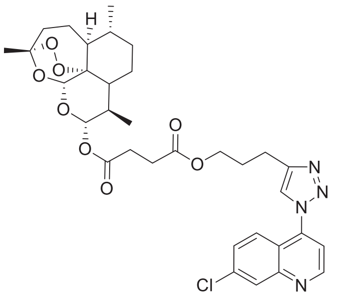

**Hybrid 6:** Compound **25** (75 mg, 0.166 mmol, 1 equiv.), 4-azido-7-chloroquinoline (**27**) (68 mg, 0.33 mmol, 2 equiv.), $CH_2Cl_2$ (3 mL), $CuSO_4{\cdot}5H_2O$ (8.3 mg, 0.033 mmol, 20 mol %), L-ascorbic acid (7 mg, 0.066 mmol, 40 mol %), $H_2O$ (3 mL). Yield: 78%.

$^1$H NMR (300 MHz, CDCl$_3$) $\delta$: 0.78 (d, *J* = 7.1 Hz, 3H), 0.91 (d, *J* = 5.1 Hz, 3H), 1.18 – 1.26 (m, 5H), 1.33 (s, 3H), 1.49 – 1.57 (m, 1H), 1.62 – 1.71 (m, 2H), 1.79 – 1.96 (m, 2H), 2.09 – 2.18 (m, 2H), 2.33 – 2.34 (m, 1H), 2.42 – 2.54 (m, 1H), 2.60 – 2.70 (m, 4H), 2.93 (t, *J* = 7.4 Hz, 2H), 4.18 (t, *J* = 6.2 Hz, 2H), 5.24 (s, 1H), 5.68 (d, *J* = 9.8 Hz, 1H), 7.49 (d, *J* = 4.6 Hz, 1H), 7.57 (dd, *J* = 9.1 Hz, 2.1 Hz, 1H), 7.91(s, 1H), 8.02 (d, *J* = 9.1 Hz, 1H), 8.19 (d, *J* = 2.1 Hz, 1H), 9.02 (d, *J* = 4.7 Hz, 1H) ppm. $^{13}$C NMR (75 MHz, CDCl$_3$) $\delta$: 12.09, 20.23, 21.92, 21.98, 24.55, 25.92, 28.06, 28.99, 29.23, 31.79, 34.05, 36.20, 37.27, 45.17, 51.50, 63.48, 80.08, 91.45, 92.26, 104.44, 116.00, 120.69, 123.32, 123.38, 124.99, 128.95, 129.31, 136.80, 141.22, 147.57, 150.21, 171.26, 172.10 ppm. HRMS (ESI+) *m/z* calculated for $C_{33}H_{39}ClN_4NaO_8$ [M+Na]$^+$: 677.2359; found: 677.2349. Elemental analysis; calculated for $C_{33}H_{39}ClN_4O_8$: C: 60.50 H: 6.00 N: 8.55 found: C: 60.57 H: 6.32 N: 8.18.

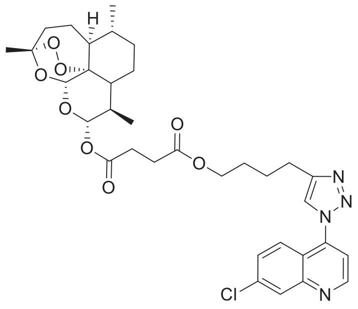

**Hybrid 7:** Compound **26** (175 mg, 0.377 mmol, 1 equiv.), 4-azido-7-chloroquinoline (**27**) (154 mg, 0.753 mmol, 2 equiv.), $CH_2Cl_2$ (6 mL), $CuSO_4{\cdot}5H_2O$ (19 mg, 0.0754 mmol, 20 mol %), L-ascorbic acid (30 mg, 0.15 mmol, 40 mol %), $H_2O$ (6 mL). Yield: 43%. $^1H$ NMR (300 MHz, $CDCl_3$) $\delta$: 0.77 (d, $J$ = 7.1 Hz, 3H), 0.87 (d, $J$ = 5.0 Hz, 3H), 1.09 – 1.24 (m, 5H), 1.33 (s, 3H), 1.49 – 1.82 (m, 9H), 2.22 – 2.32 (m, 1H), 2.43 – 2.50 (m, 1H), 2.59 – 2.66 (m, 4H), 2.86 (t, $J$ = 7.2 Hz, 2H), 4.12 (s, 2H), 5.26 (s, 1H), 5.68 (d, $J$ = 9.8 Hz, 1H), 7.47 (d, $J$ = 4.7 Hz, 1H), 7.54 (d, $J$ = 9.0 Hz, 1.8 Hz, 1H), 7.84 (s, 1H), 8.02 (d, $J$ = 9.1 Hz, 1H), 8.16 (s, 1H), 8.99 (d, $J$ = 4.7 Hz, 1H) ppm. $^{13}C$ NMR (75 MHz, $CDCl_3$) $\delta$: 12.13, 21.91, 25.11, 25.64, 28.16, 28.91, 29.22, 31.77, 36.16, 37.20, 45.15, 51.41, 64.28, 80.04, 91.41, 92.17, 104.39, 115.77, 115.90, 120.61, 122.71, 122.78, 124.84, 125.05, 128.71, 128.98, 129.26, 136.73, 141.14, 148.52, 150.16, 151.43, 171.12, 172.07 ppm. HRMS (ESI+) *m/z* calculated for $C_{34}H_{42}ClN_4O_8$ $[M+H]^+$: 669.2681; found: 669.2686. Elemental analysis; calculated for $C_{34}H_{41}ClN_4O_8$: C: 61.03 H: 6.18 N: 8.37 found: C: 60.75 H: 6.45 N: 8.00.

**The general synthesis route of compounds 28-32:** DHA was dissolved in dry $CH_2Cl_2$:MeCN (1:1) under $N_2$, subsequently the catalyst $H_3[P(W_3O_{10})_4]{\cdot}xH_2O$ (phosphotungstic acid hydrate) was added. After stirring the reaction mixture for 5 min., alcohol derivative was added. The reaction mixture was stirred 1.5-2 h and screened by thin layer chromatography to observe the consumption of DHA. When DHA was consumed, the solvent evaporated under reduced pressure and crude product was purified via column chromatography ($SiO_2$, Hexane/EtOAc). In all reactions, alpha beta isomeric mixture of the products observed, however the isomers successfully separated via column chromatography.

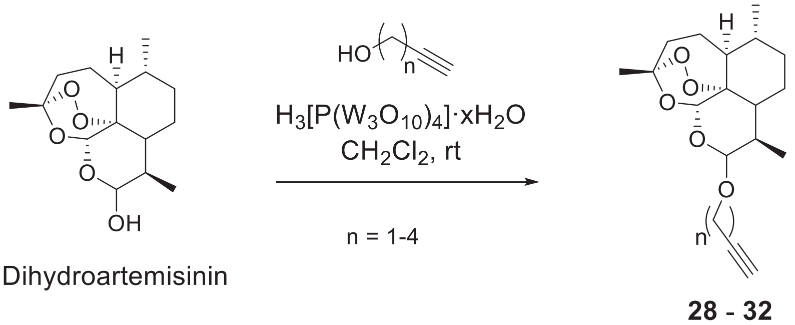


Dihydroartemisinin

**28 - 32**

**Scheme S5.** General presentation of precursor synthesis.

**Compound 28 (C-10β isomer):** DHA (250 mg, 0.88 mmol, 1 equiv.), $H_3[P(W_3O_{10})_4]{\cdot}xH_2O$ (127 mg, 0.044 mmol, 0.05 equiv.) propargyl alcohol (77 mg, 1.1 mmol, 1.25 equiv.). Yield: 86%.

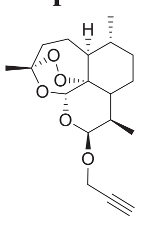

$^1H$ NMR (300 MHz, $CDCl_3$) $\delta$: 0.88 – 0.89 (m, 1H), 0.94 (d, $J$ = 12.9 Hz, 6H), 1.21 – 1.37 (m, 2H), 1.44 (s, 3H), 1.46 – 1.55 (m, 3H), 1.60 – 1.68 (m, 1H), 1.73 – 1.81 (m, 2H), 1.84 – 1.93 (m, 1H), 2.00 – 2.07 (m, 1H), 2.32 – 2.43 (m, 2H), 2.65 – 2.70 (m, 1H), 4.31 (d, $J$ = 2.7 Hz, 2H), 4.98 (d, $J$ = 3.5 Hz, 1H), 5.41 (s, 1H) ppm. $^{13}C$ NMR (75 MHz, $CDCl_3$) $\delta$: 104.24, 100.72, 88.16, 81.14, 79.88, 74.08, 55.06, 52.66, 44.46,

37.52, 36.52, 34.73, 30.70, 26.24, 24.79, 24.55, 20.44, 12.90 ppm. HRMS (ESI+) *m/z* calculated for $C_{18}H_{26}NaO_5$ $[M+Na]^+$: 345.1678; found: 345.1677.

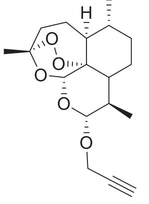

**Compound 29 (C-10α isomer):** The product was obtained as side product of compound **28**.

$^1$H NMR (300 MHz, $CDCl_3$) $\delta$: 0.88 (d, *J* = 7.2 Hz, 3H), 0.94 (d, *J* = 6.1 Hz, 3H), 0.96 – 1.03 (m, 1H), 1.20 – 1.35 (m, 3H), 1.41 (s, 3H), 1.44 – 1.57 (m, 2H), 1.65 – 1.79 (m, 2H), 1.83 – 1.89 (m, 1H), 1.97 – 2.02 (m, 1H), 2.31 – 2.44 (m, 3H), 4.33 – 4.44 (m, 2H), 4.66 (d, *J* = 9.3 Hz ,1H), 5.32 (s, 1H) ppm. $^{13}$C NMR (75 MHz, $CDCl_3$) $\delta$: 104.37, 97.62, 91.35, 80.44, 79.77, 74.29, 55.10, 51.74, 45.54, 37.44, 36.42, 34.34, 32.43, 26.08, 24.80, 22.31, 20.32, 12.48 ppm. HRMS (ESI+) *m/z* calculated for $C_{18}H_{26}NaO_5$ $[M+Na]^+$: 345.1672; found: 345.1680.

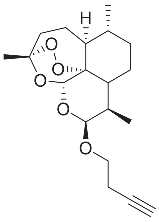

**Compound 30 (C-10β isomer):** DHA (250 mg, 0.88 mmol, 1 equiv.), $H_3[P(W_3O_{10})_4]\cdot xH_2O$ (127 mg, 0.044 mmol, 0.05 equiv.), butyn-1-ol (77 mg, 1.1 mmol, 83 µM, 1.25 equiv.). Yield: 64%.

$^1$H NMR (300 MHz, $CDCl_3$) $\delta$: 0.79 – 0.84 (m, 1H), 0.87 (d, *J* = 7.3 Hz, 3H), 0.90 (d, *J* = 6.2 Hz, 3H), 1.13 – 1.35 (m, 2H), 1.39 (s, 3H), 1.42 – 1.87 (m, 6H), 1.91 (t, *J* = 2.6 Hz, 1H), 1.95 – 2.02 (m, 1H), 2.32 (ddd, *J* = 14.5, 13.5, 4.0 Hz,1H), 2.42 (td, *J* = 6.6, 2.6 Hz, 2H), 2.53 – 2.63 (m, 1H), 3.51 (dt, *J* = 9.6, 6.3 Hz, 1H), 3.88 (dt, *J* = 9.6, 6.8 Hz, 1H), 4.78 (d, *J* = 3.3 Hz, 1H), 5.42 (s, 1H) ppm. $^{13}$C NMR (75 MHz, $CDCl_3$) $\delta$: 13.01, 20.08, 20.44, 24.34, 24.73, 26.21, 30.91, 34.73, 36.46, 37.44, 44.44, 52.62, 66.25, 69.13, 81.13, 81.78, 88.02, 101.92, 104.07 ppm. HRMS (ESI+) *m/z* calculated for $C_{19}H_{28}NaO_5$ $[M+Na]^+$: 359.1829; found: 359.1827.

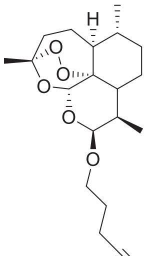

**Compound 31 (C-10β isomer):** DHA (250 mg, 0.88 mmol, 1 equiv.), $H_3[P(W_3O_{10})_4]\cdot xH_2O$ (127 mg, 0.044 mmol, 0.05 equiv.), pentyn-1-ol (92.5 mg, 1.1 mmol, 102 µM, 1.25 equiv.). Yield: 98%.

$^1$H NMR (300 MHz, $CDCl_3$) $\delta$: 0.77 (m, 1H), 0.83 (d, *J* = 7.4 Hz, 3H), 0.88 (d, *J* = 6.2 Hz, 3H), 1.08 – 1.29 (m, 2H), 1.35 (s, 3H), 1.38 – 1.99 (m, 10H), 2.20 (td, *J* = 7.1, 2.7 Hz, 2H), 2.23 – 2.34 (m, 1H), 2.49 – 2.60 (m, 1H), 3.38 (dt, *J* = 9.7, 5.9 Hz, 1H), 3.88 (dt, *J* = 9.8, 6.1 Hz, 1H), 4.71 (d, *J* = 4.6 Hz, 1H), 5.33 (s, 1H) ppm. $^{13}$C NMR (75 MHz, $CDCl_3$) $\delta$: 12.96, 15.37, 20.34, 24.44, 24.65, 26.15, 28.47, 30.87, 34.61, 36.39, 37.38, 44.39, 52.54, 66.55, 68.73, 80.99, 83.60, 87.80, 101.97, 103.96 ppm. HRMS (ESI+) *m/z* calculated for $C_{20}H_{30}NaO_5$ $[M+Na]^+$: 373.1987; found: 373.1985.

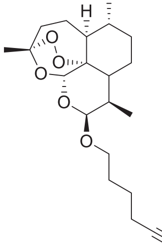

**Compound 32 (C-10β isomer):** DHA 250 mg, 0.88 mmol, 1 equiv.), $H_3[P(W_3O_{10})_4]\cdot xH_2O$ (127 mg, 0.044 mmol, 0.05 equiv.), hexyn-1-ol (108 mg, 1.1 mmol, 122.7µM 1.25 equiv.). Yield: 77%.

$^1$H NMR (300 MHz, $CDCl_3$) $\delta$: 0.85 (d, *J* = 7.1 Hz, 3H), 0.92 (d, *J* = 6.0 Hz, 3H), 0.93 – 1.02 (m, 1H), 1.17 – 1.35 (m, 3H), 1.41 (s, 3H), 1.43 – 1.88 (m, 9H), 1.91 (t, *J* = 2.6 Hz, 1H), 1.95 – 2.03 (dt, 1H), 2.19 (td, *J* = 6.9, 2.6 Hz, 2H), 2.29 – 2.44 (m, 2H), 3.40 (dt, *J* = 9.8, 6.5 Hz, 1H), 3.96 (dt, *J* = 9.8, 5.8 Hz, 1H), 4.39 (d,

$J$ = 3.6 Hz, 1H), 5.30 (s, 1H) ppm. HRMS (ESI+) *m/z* calculated for $C_{21}H_{32}NaO_5$ $[M+Na]^+$: 387.2142; found: 387.2149.

**The general synthesis route of hybrids 8-12:** The alkyne derived dihydroartemisinin (1 equiv.) and 4-azido-7-chloroquinoline (**27**) (2 equiv.) were dissolved in $CH_2Cl_2$. The solution of $CuSO_4 \cdot 5H_2O$ (20 mol %) and L-ascorbic acid (40 mol %) in $H_2O$ was then added to the reaction mixture. After stirring overnight, the crude was extracted with $CH_2Cl_2$, dried over $Na_2SO_4$, and concentrated *in vacuo*. The product was isolated via column chromatography ($SiO_2$, Hexane/EtOAc).

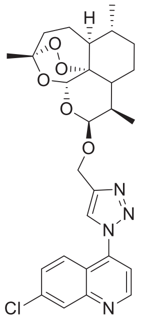

**Hybrid 8:** Compound **28** (100 mg, 0.31 mmol, 1 equiv.), 4-azido-7-chloroquinoline (**27**) (63.4 mg, 0.31 mmol, 1 equiv.), $CH_2Cl_2$ (1 mL), $CuSO_4 \cdot 5H_2O$ (3.87 mg, 0.0155 mmol, 5 mol %), L-ascorbic acid (6.14 mg, 0.031 mmol, 10 mol %), $H_2O$ (1 mL). ($SiO_2$, Hexane/EtOAc 1:1, $R_f$: 0.26) Yield: 70%.

$^1$H NMR (300 MHz, $CDCl_3$) $\delta$: 0.94 (d, $J$ = 5.5 Hz, 6H), 1.08 – 1.17 (m, 1H), 1.44 (s, 3H), 1.51-2.12 (m, 9H), 2.34 – 2.54 (m, 1H), 2.70 (s, 1H), 4.85 – 5.09 (m, 3H), 5.44 (s, 1H), 7.52 (d, $J$ = 4.6 Hz, 1H), 7.60 (dd, $J$ = 9.2, 2.2 Hz, 1H), 7.99 (s, 1H), 8.03 (s, 1H), 8.25 (s, 1H), 9.06 (d, $J$ = 4.7 Hz, 1H) ppm. $^{13}$C NMR (75 MHz, $CDCl_3$) $\delta$: 12.53, 19.82, 24.02, 24.22, 25.67, 30.40, 34.10, 35.94, 36.95, 43.89, 52.04, 61.26, 80.57, 87.55, 101.76, 103.72, 115.53, 120.19, 123.76, 124.19, 128.51, 128.94, 136.46, 140.57, 145.67, 149.75, 150.86 ppm. HRMS (ESI+) *m/z* calculated for $C_{27}H_{32}ClN_4O_5$ $[M+H]^+$: 527.2056; found: 527.2061. Elemental analysis; calculated for $C_{27}H_{31}ClN_4O_5$: C: 61.53 H: 5.93 N: 10.63 found: C: 61.26 H: 5.44 N: 10.35.

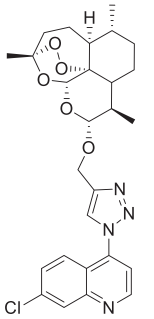

**Hybrid 9:** Compound **29** (80 mg, 0.25 mmol, 1 equiv.), 4-azido-7-chloroquinoline (**27**) (65 mg, 0.32 mmol, 1 equiv.), $CH_2Cl_2$ (1 mL), $CuSO_4 \cdot 5H_2O$ (1.25 mg, 0.05 mmol, 2 mol %), L-ascorbic acid (4.4 mg, 0.025 mmol, 10 mol %), $H_2O$ (1 ml). ($SiO_2$, Hexane/EtOAc 7:3, $R_f$: 0.12) Yield: 54%.

$^1$H NMR (300 MHz, $CDCl_3$) $\delta$: 0.89 (d, $J$ = 7.1 Hz, 3H), 0.93 (d, $J$ = 5.8 Hz, 3H), 0.99 – 1.05 (m, 1H), 1.27 – 1.31 (m, 2H), 1.33 (s, 3H), 1.38 – 1.98 (m, 7H), 2.29 – 2.40 (dd, $J$ = 31.9 Hz, 1H), 2.42 – 2.52 (m, 1H), 4.68 (d, $J$ = 9.3 Hz, 1H), 5.04 (dd, $J$ = 65.5, 12.8 Hz, 2H), 5.38 (s, 1H), 7.49 (d, $J$ = 4.6 Hz, 1H), 7.57 (d, $J$ = 9.1 Hz, 1H), 8.00 (d, $J$ = 9.1 Hz, 1H), 8.12 (s, 1H), 8.20 (d, $J$ = 2.1 Hz, 1H), 9.02 (d, $J$ = 4.6 Hz, 1H) ppm. $^{13}$C NMR (125 MHz, $CDCl_3$) $\delta$: 151.54, 150.37, 146.93, 141.24, 137.02, 129.56, 129.11, 124.92, 124.88, 120.78, 116.14, 104.48, 100.41, 91.45, 80.53, 62.68, 51.74, 45.46, 37.53, 36.41, 34.33, 32.77, 26.13, 24.84, 22.34, 20.40, 12.75 ppm. HRMS (ESI+) *m/z* calculated for $C_{27}H_{31}ClN_4NaO_5$ $[M+Na]^+$: 549.1875; found: 549.1879. Elemental analysis; calculated for $C_{27}H_{31}ClN_4O_5$: C: 61.53 H: 5.93 N: 10.63 found: C: 61.39 H: 5.89 N: 10.48.

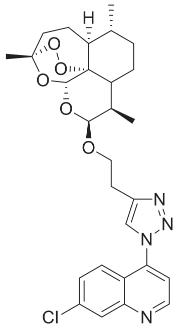

**Hybrid 10:** Compound **30** (111 mg, 0.38 mmol, 1 equiv.), 4-azido-7-chloroquinoline (**27**) (118 mg, 0.575 mmol, 1.5 equiv.), $CH_2Cl_2$ (7 mL), $CuSO_4 \cdot 5H_2O$ (19 mg, 0.076 mmol, 20 mol %), L-ascorbic acid (30 mg, 0.152 mmol, 40 mol %), $H_2O$ (7 mL). ($SiO_2$, Hexane/EtOAc 1:1, $R_f$: 0.24) Yield: 35%.

$^1$H NMR (300 MHz, $CDCl_3$) $\delta$: 0.84 (d, $J$ = 5.8 Hz, 3H), 0.86 (d, $J$ = 7.4 Hz, 3H), 0.90 – 0.92 (m, 1H), 1.21 – 1.14 (m, 2H) 1.40 (s, 3H), 1.48 – 1.98 (m, 7H), 2.33 (td, $J$ = 14.1, 3.9 Hz, 1H), 2.59 – 2.66 (m, 1H), 3.11 – 3.23 (m, 2H), 3.81 (dt, $J$ = 9.8, 6.3 Hz, 1H), 4.22 (dt, $J$ = 9.7, 6.4 Hz, 1H), 4.85 (d, $J$ = 3.4 Hz, 1H), 5.29 (s, 1H), 7.46 (d, $J$ = 4.6 Hz, 1H), 7.57 (dd, $J$ = 9.1, 2.0 Hz, 1H), 7.85 (s, 1H), 8.01 (d, $J$ = 9.1 Hz, 1H), 8.23 (d, $J$ = 2.0 Hz, 1H), 9.04 (d, $J$ = 4.6 Hz, 1H) ppm. $^{13}$C NMR (100 MHz, DMSO–$d_6$) $\delta$: 152.36, 149.55, 145.55, 140.40, 135.34, 128.84, 128.18, 125.70, 124.78, 119.99, 116.22, 103.25, 100.60, 86.90, 80.36, 66.31, 51.89, 43.68, 36.51, 35.96, 33.98, 30.44, 25.93, 25.61, 24.12, 23.77, 19.85, 12.69 ppm. HRMS (ESI+) *m/z* calculated for $C_{28}H_{34}ClN_4O_5$ $[M+H]^+$: 541.2212; found: 541.2225. Elemental analysis; calculated for $C_{28}H_{33}ClN_4O_5$: C: 62.16 H: 6.15 N: 10.36 found: C: 61.88 H: 6.03 N: 10.40.

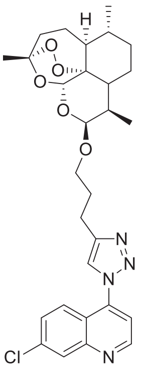

**Hybrid 11:** Compound **31** (180 mg, 0.514 mmol, 1 equiv.), 4-azido-7-chloroquinoline (**27**) (210 mg, 1.03 mmol, 2 equiv.), $CH_2Cl_2$ (6 mL), $CuSO_4 \cdot 5H_2O$ (13 mg, 0.0514 mmol, 10 mol %), L-ascorbic acid (20.4 mg, 0.103 mmol, 20 mol %), $H_2O$ (6 ml). ($SiO_2$, Hexane/EtOAc 1:1, $R_f$: 0.24) Yield: 35%.

$^1$H NMR (300 MHz, $CDCl_3$) $\delta$: 0.94 (d, $J$ = 3.4 Hz, 3H), 0.95 (d, $J$ = 2.3 Hz, 3H), 1.21 – 1.38 (m, 3H), 1.41 (s, 3H), 1.45 – 1.53 (m, 2H), 1.62 – 1.68 (m, 1H), 1.76 – 1.92 (m, 3H), 2.00 – 2.15 (m, 3H), 2.37 (td, $J$ = 13.6, 3.9 Hz, 1H), 2.62 – 2.69 (m, 1H), 2.97 (t, $J$ = 7.6 Hz, 2H), 3.52 (dt, $J$ = 9.9, 6.1 Hz, 1H), 3.96 (dt, $J$ = 9.8, 6.3 Hz, 1H), 4.83 (d, $J$ = 3.3 Hz, 1H), 5.43 (s, 1H), 7.50 (d, $J$ = 4.6 Hz, 1H), 7.61 (dd, $J$ = 9.1, 2.1 Hz, 1H), 7.81(s, 1H), 8.05 (d, $J$ = 9.1 Hz, 1H), 8.25 (d, $J$ = 2.0 Hz, 1H), 9.05 (d, $J$ = 4.7 Hz, 1H) ppm. $^{13}$C NMR (75 MHz, $CDCl_3$) $\delta$: 13.24, 20.51, 22.60, 24.70, 24.84, 26.34, 27.06, 29.37, 31.07, 34.77, 36.56, 37.66, 44.55, 52.69, 67.52, 81.24, 88.10, 102.28, 104.28, 115.96, 120.80, 122.82, 124.97, 129.09, 129.50, 137.00, 141.31, 150.36, 151.50 ppm. HRMS (ESI+) *m/z* calculated for $C_{29}H_{35}ClN_4NaO_5$ $[M+Na]^+$: 577.2188; found: 577.2175. Elemental analysis; calculated for $C_{29}H_{35}ClN_4O_5$: C: 62.75 H: 6.36 N: 10.09 found: C: 63.17 H: 6.45 N: 9.75.

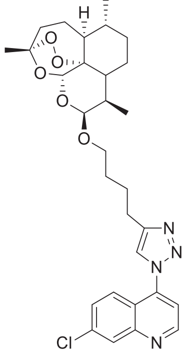

**Hybrid 12:** Compound **32** (133 mg, 0.365 mmol, 1 equiv.), 4-azido-7-chloroquinoline (**27**) (90 mg, 0.438 mmol, 2 equiv.), $CH_2Cl_2$ (6 mL), $CuSO_4 \cdot 5H_2O$ (3.6 mg, 0.015 mmol, 4 mol %), L-ascorbic acid (14.5 mg, 0.073 mmol, 20 mol %), $H_2O$ (6 mL). ($SiO_2$, Hexane/EtOAc 1:1, $R_f$: 0.24) Yield: 32%.

$^1$H NMR (300 MHz, $CDCl_3$) $\delta$: 0.84 (d, $J$ = 5.7 Hz, 3H), 0.86 (d, $J$ = 7.4 Hz, 3H), 0.90 – 0.92 (m, 1H), 1.13 – 1.20 (m, 3H), 1.40 (s, 3H), 1.43 – 2.01 (m, 8H), 2.33 (td, $J$ = 14.0, 3.9 Hz, 1H), 2.59 – 2.66 (m, 1H), 3.11 – 3.23 (m, 2H), 3.81 (dt, $J$ = 9.8, 6.3 Hz, 1H), 4.22 (dt, $J$ = 9.8, 6.4 Hz, 1H), 4.85 (d, $J$ = 3.5 Hz, 1H), 5.29 (s, 1H), 7.46 (d, $J$ = 4.6 Hz, 1H), 7.57 (dd, $J$ = 9.1, 2.2 Hz, 1H), 7.85 (s, 1H), 8.01

(d, $J$ = 9.1 Hz, 1H), 8.23 (d, $J$ = 2.1 Hz, 1H), 9.04 (d, $J$ = 4.6 Hz, 1H) ppm. $^{13}$C NMR (75 MHz, $CDCl_3$) $\delta$: 13.12, 20.39, 24.53, 24.69, 25.30, 26.08, 26.23, 29.31, 30.94, 34.64, 36.44, 37.49, 44.44, 52.55, 68.01, 81.11, 87.93, 102.04, 104.09, 115.84, 120.66, 122.52, 124.86, 128.94, 129.31, 136.81, 141.18, 148.90, 150.21, 151.42 ppm. HRMS (ESI+) *m/z* calculated for $C_{30}H_{37}ClN_4NaO_5$ $[M+Na]^+$: 591.2344; found: 591.2342. Elemental analysis; calculated for $C_{30}H_{37}ClN_4O_5$: C: 63.32 H: 6.55 N: 9.85 found: C: 63.57 H: 6.63 N: 9.50.

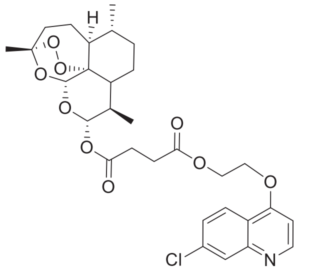

**Hybrid 13:** Artesunic acid (100 mg, 0.26 mmol, 1 equiv.) and 7-chloroquinoline derivative **34** (58 mg, 0.26 mmol, 1 equiv.) were dissolved in dry $CH_2Cl_2$ (0.52 mL) under $N_2$. DCC (59 mg, 0.286 mmol, 1.1 equiv.) and DMAP (127 mg, 1.04 mmol, 4 equiv.) were added at 0 °C to the solution. The reaction mixture was warmed up to room temperature and stirred overnight. After filtration of the precipitated urea, solvent was evaporated, and the hybrid **13** was purified via column chromatography ($SiO_2$, $CH_2Cl_2$/MeOH 95:5, $R_f$: 0.67), 35%, white crystalline.

$^1$H NMR (300 MHz, $CDCl_3$) $\delta$: 0.78 (d, $J$ = 7.1 Hz, 3H), 0.94 (d, $J$ = 5.8, 3H), 1.39 (s, 3H), 1.55 – 1.71 (m, 6H), 1.85 – 1.94 (m, 2H), 2.29 – 2.41 (m, 1H), 2.48 – 2.56 (m, 1H), 2.71 – 2.72 (m, 4H), 3.41 – 3.52 (m, 1H), 4.00 – 4.11 (m, 1H), 4.38 – 4.41 (m, 2H), 4.59 – 4.61 (m, 2H), 5.28 – 5.34 (d, $J$ =17.2, 1H), 5.69 – 5.72 (d, $J$ = 9.7, 1H), 6.73 – 6.75 (d, $J$ = 5.3, 1H), 7.46 – 7.50 (d, $J$ = 9.0 Hz, 1H), 8.06 (s, 1H), 8.14 – 8.17 (d, $J$ = 9.0, 1H), 8.72 – 8.74 (d, $J$ =5.2, 1H) ppm. $^{13}$C NMR (75 MHz, $CDCl_3$) $\delta$: 12.12, 20.33, 22.05, 24.69, 25.09, 26.07, 28.97, 29.26, 31.90, 34.09, 34.18, 36.33, 37.38, 45.29, 49.24, 51.66, 62.37, 66.61, 80.22, 91.61, 92.36, 101.05, 104.60, 123.80, 126.98, 136.19, 152.36, 161.46, 171.09, 172.09 ppm. MS (MALDI-TOF): m/z calculated for $C_{30}H_{36}ClNO_9$ $[M]^+$ = 590; found: 590. Elemental analysis; calculated for $C_{30}H_{36}ClNO_9$: C: 61.07 H: 6.15 N: 2.37 found: C: 61.26 H: 6.44 N: 2.35.

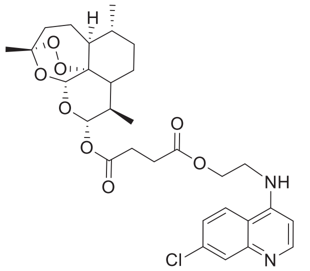

**Hybrid 14:** Artesunic acid (100 mg, 0.26 mmol, 1 equiv.) and 7-chloroquinoline derivative **33** (115.8 mg, 0.52 mmol, 2 equiv.) were dissolved in dry $CH_2Cl_2$ (7.5 mL) under $N_2$. DCC (59 mg, 0.286 mmol, 1.1 equiv.) and DMAP (63 mg, 0.52 mmol, 2 equiv.) were added at 0 °C to the solution. The reaction mixture was warmed up to room temperature and stirred overnight. After filtration of the precipitated urea, solvent was evaporated, and the hybrid **14** was purified via column chromatography ($SiO_2$, Hexane/EtOAc 2:8, $R_f$: 0.2), 70%, white crystalline.

$^1$H NMR (300 MHz, $CDCl_3$) $\delta$: 0.73 (d, $J$ = 7.1 Hz, 3H), 0.92 (d, $J$ = 5.8 Hz, 3H), 1.18 – 1.24 (m, 4H), 1.31 (s, 3H), 1.49 – 1.64 (m, 3H), 1.79 – 2.00 (m, 2H), 2.25 – 2.35 (m, 2H), 2.45 – 2.57 (m, 1H), 2.67 – 2.73 (m, 4H), 3.57 – 3.62 (m, 2H), 4.45 (t, $J$ = 5.1 Hz, 2H), 5.14 (s, 1H), 5.62 (d, $J$ = 9.9 Hz, 1H), 5.92 (brs, 1H), 6.37 (d, $J$ = 5.5 Hz, 1H), 7.41 (dd, $J$ = 2.2 Hz, 1H), 7.90 (d, $J$ = 9.0 Hz, 1H), 7.96 (d, $J$ = 2.1 Hz, 1H), 8.49 (d, $J$ = 5.5 Hz, 1H) ppm. $^{13}$C (75 MHz, $CDCl_3$) $\delta$: 11.43, 19.60, 21.25, 24.00, 25.25, 28.75, 28.94, 31.12, 33.50, 35.69, 36.66, 41.74, 44.55, 50.97, 61.78, 79.52, 90.96, 91.91, 98.35, 103.91, 116.98, 121.69, 124.76, 127.94, 134.46, 148.69, 149.24, 151.38, 171.03, 171.61 ppm. HRMS (ESI+) *m/z* calculated for $C_{30}H_{38}ClN_2O_8$

$[M+H]^+$: 589.2311; found: 589.2323. Elemental analysis; calculated for $C_{30}H_{37}ClN_2O_8$ C: 61.17 H: 6.33 N: 4.76 found: C: 60.75 H: 6.46 N: 4.50.

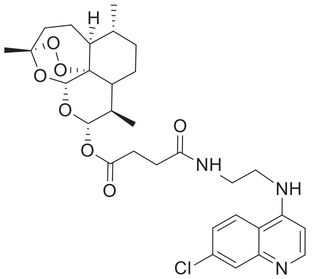

**Hybrid 15:** Artesunic acid (100 mg, 0.26 mmol, 1 equiv.) was dissolved in dry $CH_2Cl_2$. The coupling agent EDCI (40 mg, 0.26 mmol, 1 equiv.) which was used to activate the carboxyl of artesunic acid, was added at 0 ºC. DMAP (32 mg, 0.26 mmol, 1 equiv.) and 7-chloroquinoline derivative **36** (115 mg, 0.52 mmol, 2 equiv.) were added subsequently. The reaction mixture was warm up to room temperature and stirred overnight. The reaction was worked up with water (25 mL) and extracted with $CH_2Cl_2$ (25 mL x 3). The combined $CH_2Cl_2$ phase was evaporated and the hybrid **15** was isolated via column chromatography ($SiO_2$, $CH_2Cl_2$/MeOH 15:1, $R_f$: 0.2), 61%, white solid.

$^1$H NMR (300 MHz, $CDCl_3$) $\delta$: 0.73 (d, $J$ = 7.1 Hz, 3H), 0.85 – 0.97 (m, 5H), 1.34 (s, 3H), 1.49 – 1.56 (m, 1H), 1.62 – 1.67 (m, 2H), 1.80 – 1.87 (m, 1H), 1.93 – 1.99 (m, 1H), 2.22 – 2.34 (m, 1H), 2.43 – 2.65 (m, 6H), 2.75 – 2.79 (m, 2H), 3.39 – 3.76 (m, 4H), 4.96 (s, 1H), 5.59 (d, $J$ = 9.9 Hz, 1H), 6.30 (d, $J$ = 5.7 Hz, 1H), 6.90 (d, $J$ = 6.1 Hz, 2H), 7.39 (dd, $J$ = 8.9, 2.2 Hz, 1H), 7.93 (s, 1H), 7.96 (s, 1H), 8.42 (d, $J$ = 5.6 Hz, 1H) ppm. $^{13}$C NMR (75 MHz, $CDCl_3$) $\delta$: 11.41, 13.60, 19.59, 21.33, 22.15, 23.99, 25.31, 27.76, 30.22, 30.99, 31.08, 31.23, 33.48, 35.63, 36.65, 37.91, 42.61, 44.01, 44.55, 50.87, 79.47, 90.84, 92.04, 97.75, 103.93, 116.56, 122.18, 125.23, 171.58, 173.25 ppm. HRMS (ESI+) *m/z* calculated for $C_{30}H_{39}ClN_3O_7$ $[M+H]^+$: 588.2471; found: 588.2462. Elemental analysis; calculated for $C_{30}H_{38}ClN_3O_7$ C: 61.27 H: 6.51 N: 7.15 found: C: 61.40 H: 6.89 N: 6.86.

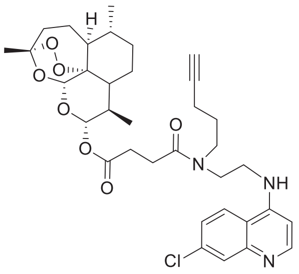

**Hybrid 16:** Artesunic acid (66 mg, 0.172 mmol, 1 equiv.) was dissolved in dry $CH_2Cl_2$. The coupling agent EDCI (40 mg, 0.26 mmol, 1 equiv.) which was used to activate the carboxylic acid of artesunic acid, was added at 0 ºC. DMAP (32 mg, 0.26 mmol, 1 equiv.) and 7-chloroquinoline derivative **37** (99 mg, 0.344 mmol, 2 equiv.) were added subsequently. The reaction mixture was warm up to room temperature and stirred overnight. The reaction was worked up with water (25 mL) and extracted with $CH_2Cl_2$ (25 mL x 3). The combined $CH_2Cl_2$ fraction was evaporated and the hybrid **16** was isolated via column chromatography ($SiO_2$, $CH_2Cl_2$/MeOH 10:2), 72%, white solid.

$^1$H NMR (300 MHz, $CDCl_3$) $\delta$: 8.43 (d, $J$ = 5.8 Hz, 1H), 8.06 (s, 1H), 7.79 (d, $J$ = 9.0 Hz, 1H), 7.52 (brs, H), 7.44 (dd, $J$ = 8.9, 2.2 Hz, 1H), 6.33 (d, $J$ = 5.8 Hz, 1H), 5.68 (d, $J$ = 9.8 Hz, 1H), 5.34 (s, 1H), 3.85 (t, $J$ = 5.3 Hz, 2H), 3.60 – 3.47 (m, 4H), 2.85 – 2.66 (m, 5H), 2.57 – 2.47 (m, 2H), 2.40 – 2.27 (m, 4H), 2.07 (t, $J$ = 2.6 Hz, 1H), 2.06 – 1.99 (m, 1H), 1.91 – 1.84 (m, 3H), 1.71 – 1.46 (m, 4H), 1.41 (s, 3H), 1.02 – 1.98 (m, 1H), 0.96 (d, $J$ = 5.8 Hz, 3H), 0.76 (d, $J$ = 7.1 Hz, 3H) ppm. $^{13}$C NMR (125 MHz, $CDCl_3$) $\delta$: 174.76, 171.57, 136.47, 126.40, 122.82, 116.80, 104.60, 97.85, 92.33, 91.60, 82.43, 80.25, 70.40, 51.67, 47.84, 47.41, 45.62, 45.27, 44.35, 37.39, 36.36, 34.15, 31.96, 31.73, 29.77, 27.97, 27.43, 26.10, 24.70, 22.80, 22.07, 20.34, 15.93, 14.27, 12.12 ppm. HRMS (ESI+) *m/z* calculated for $C_{35}H_{45}ClN_3O_7$ $[M+H]^+$: 654.2941; found:

654.2950. Elemental analysis; calculated for $C_{35}H_{44}ClN_3O_7$ C: 64.26 H: 6.78 N: 6.42 found: C: 63.99 H: 7.00 N: 6.05.

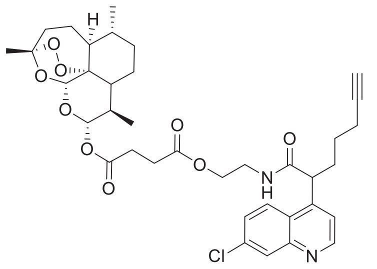

**Hybrid 17:** To a stirred solution of hybrid **14** (589.1 mg, 1.00 mmol) in $CH_2Cl_2$ (20 mL) *hept*-6-ynoyl chloride (289.2 mg, 2.00 equiv.) and $Et_3N$ (0.42 mL, 3.00 equiv.) was added at 0 °C. The reaction mixture was warmed to room temperature and stirred for a further 18 h. The reaction was poured into an aqueous $NaHCO_3$ solution and the aqueous layer was extracted with $CH_2Cl_2$. The combined organic layers were washed with brine, dried over $MgSO_4$ and concentrated. The crude residue was purified by column chromatography (*n*-hexane/EtOAc: 2/3) to afford the hybrid **17** as a white solid (425.3 mg, 61%).

$^1$H NMR (600 MHz, $CDCl_3$) (dr: 1.0/1.0) $\delta$ = 8.86 (d, *J* = 4.5 Hz, 1H), 8.80 – 8.79 (d, *J* = 9.2 Hz, 1H), 8.19 – 8.17 (d, *J* = 9.2 Hz, 1H), 8.08 – 8.09 (d, *J* = 2.2 Hz, 1H), 8.07–8.06 (d, *J* = 2.2 Hz, 2H), 7.53 – 7.51 (m, 2H), 7.50 – 7.48 (m, 2H), 6.66 (t, *J* = 5.8 Hz, 1H), 6.37 (t, *J* = 5.8 Hz, 1H), 5.46 (d, *J* = 9.9 Hz, 1H), 5.42 (d, *J* = 9.9 Hz, 1H), 5.00 (s, 1H), 4.55 (s, 1H), 4.38 (ddd, *J* = 11.0, 7.7, 3.1 Hz, 1H), 4.25 – 4.20 (m, 2H), 4.16 (dd, *J* = 8.5, 6.4 Hz, 1H), 4.11 – 4.01 (m, 3H), 3.50 – 3.36 (m, 3H), 3.26 – 3.21 (m, 1H), 2.69 – 2.64 (m, 1H), 2.55 –.41 (m, 7H), 2.36 – 2.18 (m, 9H), 2.06 – 2.00 (m, 3H), 1.92 (t, *J* = 2.6 Hz, 1H), 1.91 (t, *J* = 2.6 Hz, 1H), 1.88 – 1.60 (m, 10H), 1.58 – 1.42 (m, 3H), 1.28 – 1.15 (m, 7H), 1.08 (s, 3H), 1.07 – 0.97 (m, 2H), 0.95 – 0.84 (m, 10H), 0.74 (d, *J* = 7.1 Hz, 3H), 0.64 (d, *J* = 7.1 Hz, 3H). $^{13}$C NMR (125 MHz, $CDCl_3$) (dr: 1.0/1.0) $\delta$ = 171.6, 171.5, 171.4, 171.3, 171.0, 171.0, 151.4, 151.3, 148.9, 148.8, 147.1, 145.4, 134.9, 134.9, 129.2, 129.1, 127.5, 127.3, 125.5, 125.3, 124.8, 124.6, 120.0, 119.7, 104.3, 104.1, 92.3, 92.2, 91.3, 91.3, 83.9, 83.6, 79.9, 79.8, 69.0, 68.8, 62.8, 62.2, 51.4, 51.2 , 47.8, 46.7, 45.0, 44.8, 39.0, 38.9, 37.1, 37.0, 36.1, 36.1, 33.9, 33.9, 32.6, 31.8, 31.5, 31.3, 29.4, 29.4, 29.3, 29.2, 26.7, 26.6, 25.7, 25.4, 24.4, 24.2, 21.9, 21.9, 20.1, 20.0, 18.3, 18.1, 12.0, 11.9. MS (ESI) *m/z* (relative intensity) 697 (100) $[M+H]^+$. HRMS (ESI+) *m/z* calculated for $C_{37}H_{46}{}^{35}ClN_2O_9$ $[M+H]^+$: 697.2886, found: 697.2879. Elemental analysis; calculated for $C_{37}H_{45}ClN_2O_9$: C, 63.74; H, 6.51; N, 4.02; Found: C, 63.68; H, 6.62; N, 4.01.

## 3. Stability experiments

**Thermal stability:** The thermal stability test of hybrids **1**, **4**, **7**, **9**, **10**, **13**, and **14**, was performed in DMSO-d$_6$ at 60 ºC for 60 h, and their stability was confirmed via $^1$H and $^{13}$C NMR spectroscopy. Decomposition of the hybrids was not observed, showing that hybrids are highly thermally stabile. (See following pages, the selected NMR spectra). **Hydrolytic stability:** The hydrolytic stability of the hybrid **14** was observed in hydrochloric acid (nonenzymatic simulated gastric fluid, pH 1-2) and phosphate buffer (nonenzymatic simulated intestinal fluid, pH 6.8) for 84 h. The sample was extracted from solution with dichloromethane and measured via $^1$H NMR at 400 MHz. The results showed that the ester derivative (hybrid **14**) remained stable in simulated intestinal fluids for 84 h. In simulated gastric fluid the hybrid **14** hydrolyzes to alcohol and acid. The stability of hybrid **14** in the acetate buffer at pH 5 and 37 ºC for 24 h was also confirmed via $^1$H NMR that hybrid **14** remain stable. **Enzymatic stability:** Even though the

ester bonds known to be hydrolyze to acid and alcohol by carboxyl esterase, some of them can persist to esterase.[4,5] We tested the hydrolysis ratio of ester bond containing hybrid **14** in the presence of carboxyl esterase (PLE) for 24 h at room temperature according to published protocol.[6] The hybrid remained intact after 24 h according to $^1$H NMR and ESI-MS measurements. Additionally, stability of hybrid **14** (5 mg) was tested in 1 mL human serum (HS) and 1 mL fetal bovine serum (FBS) at 37 ºC for 24 h. The stability of the hybrid was confirmed via $^1$H NMR (see following pages).

## 4. Biological methods

### Cytotoxicity studies against *P. falciparum* 3D7 strains

***P. falciparum* culture.** *P. falciparum* 3D7, Dd2, and K1 parasites were cultured in type A-positive human erythrocytes at a hematocrit of 5% in RPMI 1640 supplemented with 25 mM HEPES, 2 mM l-glutamine, 24 mM $NaHCO_3$, 0.1 mM hypoxanthine, 25 µg/mL gentamycin and 0.5% albumax I. Cultures were incubated at 37 °C under controlled atmospheric conditions of 3% $O_2$, 5% $CO_2$, and 92% $N_2$ at 95% relative humidity.

***In vitro* antimalarial activity assay.** Cultures used in cell proliferation assays were synchronized by sorbitol treatment.[7] Concentrations to inhibit parasite growth by 50% ($EC_{50}$) were determined using the SYBR Green I malaria drug sensitivity assay.[8] 50 µl aliquots of a cell suspension containing ring stages at a parasitemia of 0.2% and a hematocrit of 2% were added to the wells of 96-well microtiter plates. Plates were incubated for 72 h in the presence of different drug concentrations. Subsequently, cells of each well were lysed with 100 µl lysis buffer (40 mM Tris, pH 7.5, 10 mM EDTA, 0.02% saponin, 0.08% Triton X-100) containing 8.3 µM SYBR green. Plates were incubated for 1 h in the dark at room temperature under constant mixing before fluorescence (excitation wavelength 485 nm; emission wavelength 520 nm) was determined using a microtiter plate fluorescence reader (Victor X4; Perkin Elmer). Drugs were serially diluted 1:3 with initial drug concentrations being 243 nM for artesunic acid and its derivates, 81 nM for dihydroartemisinin and and 243 nM for its derivates and 243 nM for chloroquine when the strain 3D7 was used and 729 nM when Dd2 and K1 strains were examined. For compounds **3**, **33**, **36**, and **18** the initial drug concentrations were 100 µM, when the $EC_{50}$ values were above 100 nM and 243 nM for compound **36** when 3D7 was used as strain. Except for hybrid **3**, each drug concentration was examined in triplicate and repeated at least three times. Uninfected erythrocytes (hematocrit 2%) and infected erythrocytes without drug served as controls and were investigated in parallel. Percent growth was calculated as described by Beez.[9] Data were analyzed using the SigmaPlot (version 12.0; Hill function, three parameters) and Sigma Stat programs.

### Efficacy of hybrid compounds in the experimental malaria

**Drugs and dilutions**: Artesunate was supplied by FarManguinhos (Rio de Janeiro, Brazil). All drugs were dissolved in propylene glycol (Merck Millipore, Darmstadt, Germany) and then diluted in sterile saline to a final concentration of 2:1 (v/v) of propylene glycol:saline.

**Parasite and animals:** *P. berghei* expressing green fluorescent protein (GFP)[10] was kindly supplied by Prof. Antoniana Ursine Krettli (Fiocruz Minas/Brazil). Male Swiss Webster mice (18–22 g) were housed at Instituto Gonçalo Moniz (Brazil), maintained in sterilized cages under a controlled environment, receiving a rodent balanced diet and water *ad libitum*. All experiments were carried out in accordance with the recommendations of Ethical Issues Guidelines and were approved by the Animal Ethics Committee of Instituto Gonçalo Moniz (protocol number 016/2017).

**Suppressive activity (Peters test):** Mice were infected by intraperitoneal injection of $1 \times 10^6$ GFP-*P. berghei*-infected erythrocytes per mouse and randomly divided into groups of *n*=5. Treatment was initiated 3 h post infection and given once a day for four consecutive days by subcutaneous injection of 100 µL or orally by gavage of a 200 µL volume. Untreated infected mice receiving propylene glycol /saline (2:1 v/v) were used as negative control (vehicle). At days 5-24 post-infection, two drops of blood collected from the tail were diluted in 200 µL of saline containing 5 U/mL of heparin sulphate. Parasitemia was determined in a flow cytometer (BD LSRFortessa) using at least 100`000 events and parasites were gated at FTIC channel. Animal survival was observed daily until day 41 post-infection. The % of parasitemia reduction was calculated as follow: [mean vehicle group − mean treated group/mean vehicle group] × 100. Experiments were independent repeated for treatment which inhibited > 70% the parasitemia.

**Curative activity (Thompson test):** Mice were infected by intraperitoneal injection of $1 \times 10^6$ GFP-*P. berghei*-infected erythrocytes per mouse. At 4 dpi, mice which reached parasitemia 2-3% were randomly divided into groups of *n* = 6 and treatment was initiated 5 dpi and given once a day for three consecutive days by subcutaneous injection of 100 µL. Untreated infected mice receiving propylene glycol /saline (2:1 v/v) were used as negative control (vehicle). At days 11-24 post-infection, two drops of blood collected from the tail were used for parasitemia determination and animal survival were determined as described above.

**Inhibition of β-hematin formation**: In a 96-well U-bottom microplate, 100 µL of a 0.5 mg/mL solution of heme dissolved in 0.2 NaOH solution was distributed. Compounds were dissolved in 20% DMSO in saline and a 50 µL of different concentrations of compounds at range of 2-0.5 mM was added in triplicate. After, 25 µL of acetic acid and 75 µL of sodium acetate buffer (3 M) were added and the plate incubated at 37ºC for 24 h. After cooling at room temperature, plate was centrifuged at 3300 g for 25 min, washed with water. Pellet was resuspended with 200 µL of DMSO, centrifuged and the remaining pellet was dissolved in 150 µL of 0.1 M NaOH solution and the plate was read for spectroscopic quantitation at 405 nm. Three independent experiments were performed, $IC_{50}$ values were calculated in Prism fitting into a non-linear regression equation.

**Hemozoin analyses:** Mice were infected by intraperitoneal injection of 2 × 106 GFP-*P. berghei*-infected erythrocytes per mouse. When mice reached a parasitemia of 8-10 %, they were randomly divided into groups of *n*=3 and received treatment once a day by oral gavage of 105 µmol/kg body weight of hybrid **14** and chloroquine (210 µmol/kg body weight). Untreated

infected mice receiving propylene glycol /saline (2:1 v/v) and untreated uninfected mice were used as controls (vehicles). Before and after 24 h, two drops of blood collected from the tail were used for parasitemia determination by flow cytometry as described above. After 24 h of treatment, mice received anesthesia, blood was gently aspirated in the brachial plexus using a heparin coated tip and transferred into heparinized vials on ice. The blood samples were centrifuged at 2500 rpm at 4 °C for 15 min. to remove plasma. White cells were removed by filtration into a cellulose column pad and the pellets resulting from red cells were stored at -80ºC until analysis. Total hemogloblin, free heme and hemozoin of each sample were quantified following a protocol described by Egan et al.[11]

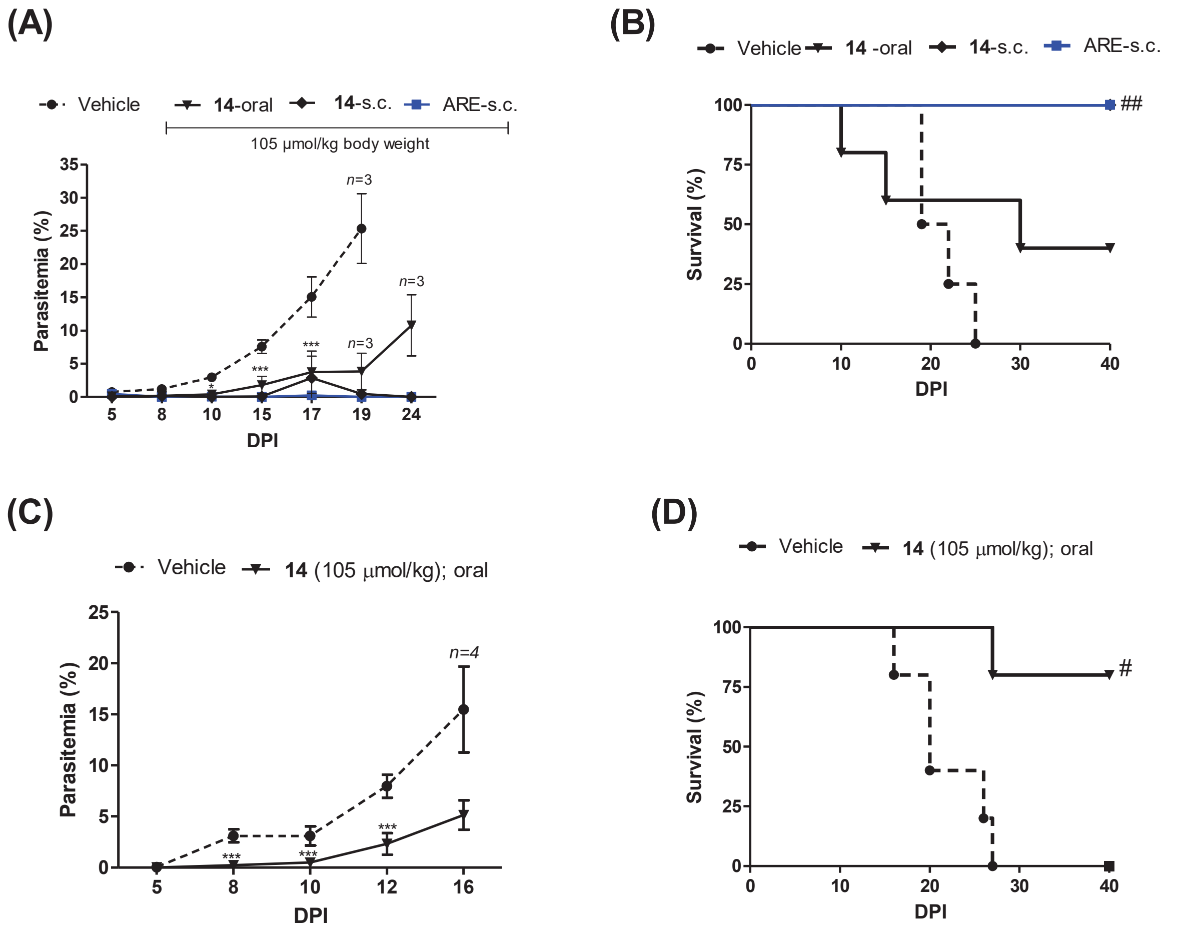


**Figure S1.** Parasitemia (A, C) and animal survival (B, D) of *P. berghei*-infected mice in the suppressive Peters test (treatment initiated 3 h post-infection). Compounds were given at dose of 105 µmol/kg and the administration route is indicated in the legend caption. Parasitemia and animal survival were measured using an *n*=5/group, unless indicated. Panels C and D are the results of independent experiment repetition for hybrid **14** given by oral administration. Error bars indicate standard deviation. *p<0.05, ***p<0.01 (two-way ANOVA) versus vehicle group. $^{\#}$p<0.01, $^{\#\#}$p<0.005 (Log-rank, Mantel-Cox test) versus vehicle. ARE = Artesunate, given subcutaneously. DPI = days post-infection.

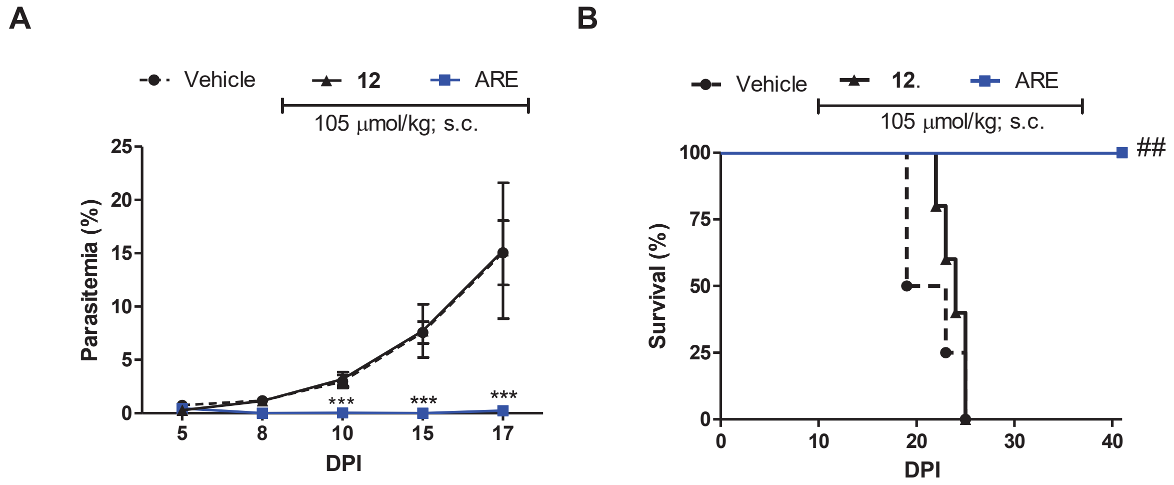


**Figure S2.** Parasitemia (A) and animal survival (B) of *P. berghei*-infected mice in the suppressive Peters test (treatment initiated 3 h post-infection). Hybrid **12** or ARE were given subcutaneously (s.c.) at dose of 105 μmol/kg. Parasitemia and animal survival were measured using an *n*=5/group. Error bars indicate standard deviation. *p<0.05, ***p<0.01 (two-way ANOVA) versus vehicle group. ##p<0.005 (Log-rank, Mantel-Cox test) versus vehicle. ARE = Artesunate, given subcutaneously. DPI = days post-infection.

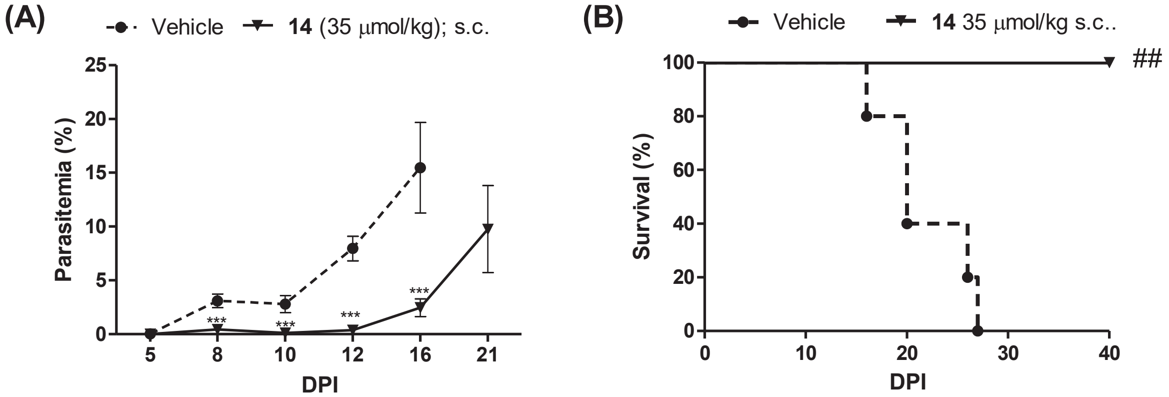


**Figure S3.** Parasitemia (A) and animal survival (B) of *P. berghei*-infected mice in the suppressive Peters test (treatment initiated 3 h post-infection). Compounds **14** were given subcutaneously (s.c.) at indicated dose. Parasitemia and animal survival were measured using an *n*=5/group. Error bars indicate standard deviation. *p<0.05, ***p<0.01 (two-way ANOVA) versus vehicle group. ##p<0.005 (Log-rank, Mantel-Cox test) versus vehicle. DPI = days post-infection.

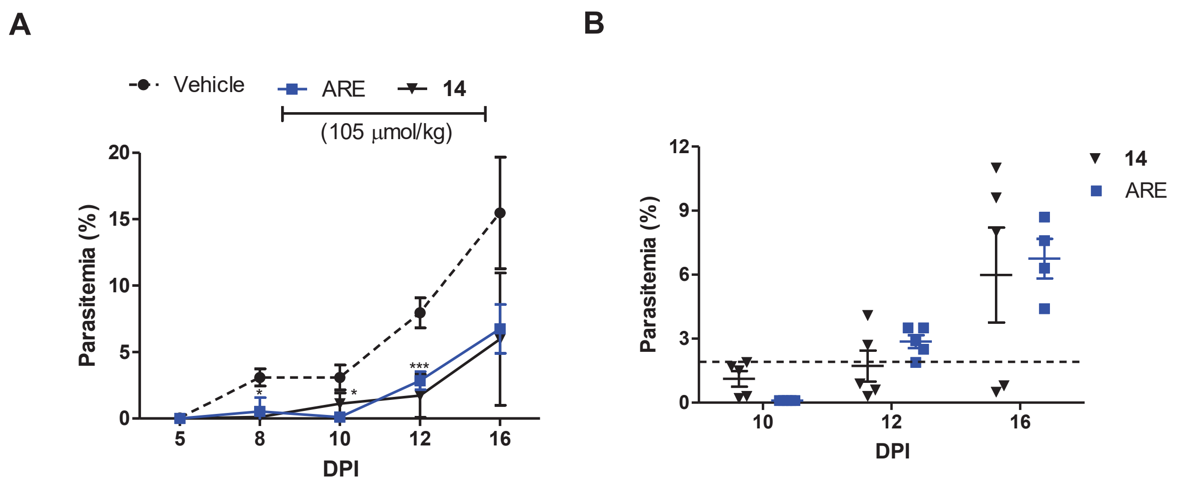


**Figure S4.** Curve of parasitemia (A), recrudescence of parasitemia (B) and summary of activities (Table S1) in *P. berghei*-infected mice treated with hybrid **14** or Artesunate (ARE) in intercalated regime at oral dose of 105 µmol/kg against *P. berghei*-infected mice.

**Table S1.** Raw values of Peters test.

| Compounds | % Parasitemia inhibition [a] | Median survival time (days) | Parasite recrudescence (%)[b] | |
|---|---|---|---|---|
| | | | Day 12 | Day 16 |
| Vehicle | -,- | 20 | - | - |
| ARE | 84.9±6.3 | 23 | 80 | 100 |
| Hybrid **14** | 74.0±4.1 | 30 | 40 | 60 |

[a] Values are mean ± standard deviation and were taken from 10 dpi. [b] Percentage of mice with detectable parasites (parasitemia > 2 %). Experiment using *n*=5/group. Oral administration by gavage after 3 and 48 h post-infection. Parasitemia was measured at indicated day using an *n*=5/group. Error bars indicate standard deviation. *p<0.05, ***p<0.01 (two-way ANOVA) versus vehicle group. In panel B, dashed line indicates 2 % parasitemia value cutoff.

**Table S2.** Row values of animal survival analysis of *P. berghei*-infected mice in the curative Thompson test (treatment initiated 5 dpi). Compounds were given subcutaneously at daily dose of 140 µmol/kg for three consecutive days. Animal survival were measured using an *n*=6/group.

| Compounds | Median survival time (days) | Number of animal alive (41 dpi) | Percentage of cured mice |
|---|---|---|---|
| Vehicle | 19 | 1/6* | 0 |
| ARE | 33 | 3/6** | 33.3 |
| **14** | >40 | 6/6 | 100 |

*Mouse presented blood smear positive for parasites (parasitemia of 20 %) in the 41 dpi. **One of three mice presented blood smear positive for parasites in the 41 dpi.

**Table S3.** Inhibition of heme polymerization into β-hematin, determined 24 h after drug incubation.

| Compounds | $IC_{50}$ ± standard deviation (mM) |
|---|---|
| Chloroquine diphosphate | 0.89±0.21 |
| Primaquine hydrochloride | >2.0 |
| Artesunate | >2.0 (23.4±5.4 % inhibition at 2.0 mM) |
| **14** | 0.56±0.15 |

**Parasite labelling for target identification.**

*P. falciparum* parasites (unsynchronized) were cultured with 1.25% haematocrit in 150 mm plate. Parasite were enriched and isolated as previously described.[12] Parasite proteins were extracted by suspending the pellets in 500 μL 0.16% SDS in PBS with protease inhibitors. Protein concentration of the lysates was quantified with BCA protein quantitation kit. About 500 μg of protein was treated with streptavidin beads attached by hybrid **16** and **17** for 4 h at room temperature to capture the target proteins. After washing with 0.1% SDS, and PBS, the beads were suspended in 100 µl triethylammonium bicarbonate (25 mM), then added 2 µl TCEP (100mM stock solution). The beads were heated at 65 °C for 1 h, subsequently1 µl MMTS (200mM stock solution) was added to react for 15 min at room temperature. Once reduction and alkylation performed, trypsin was added into the samples and incubated at 37 ºC overnight. The digested peptides were isolated from beads via a filter-spin column (GE Healthcare). Liquid chromatography–mass spectrometry/mass spectrometry method was described previously.[12]

**Filtering PAINS Elements among Hybrid Compounds 1 – 17 (Calculated)**

All seventeen hybrid compounds (**1 - 17**) were screened for pan assay interference compounds (PAINS) by FAF-Drugs4 filtering tool (server available at http://fafdrugs4.mti.univ-paris-diderot.fr).[13] All hybrids passed the PAINS filters (See following pages).

# 5. ¹H and ¹³C NMR spectra and mass spectra of the compounds

¹H NMR spectrum of **18** recorded on a Bruker Avance spectrometer (300 MHz, $CDCl_3$):

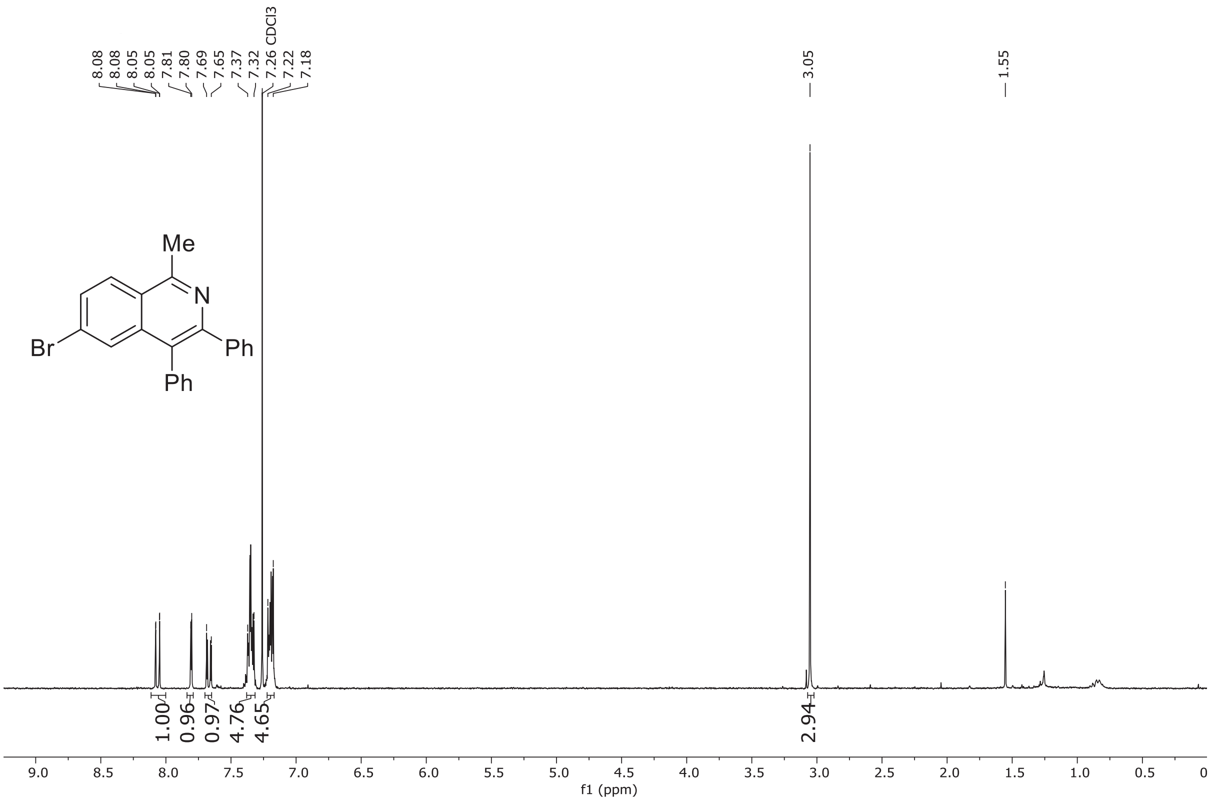


¹³C NMR spectrum of **18** recorded on a Bruker Avance spectrometer (75 MHz, $CDCl_3$):

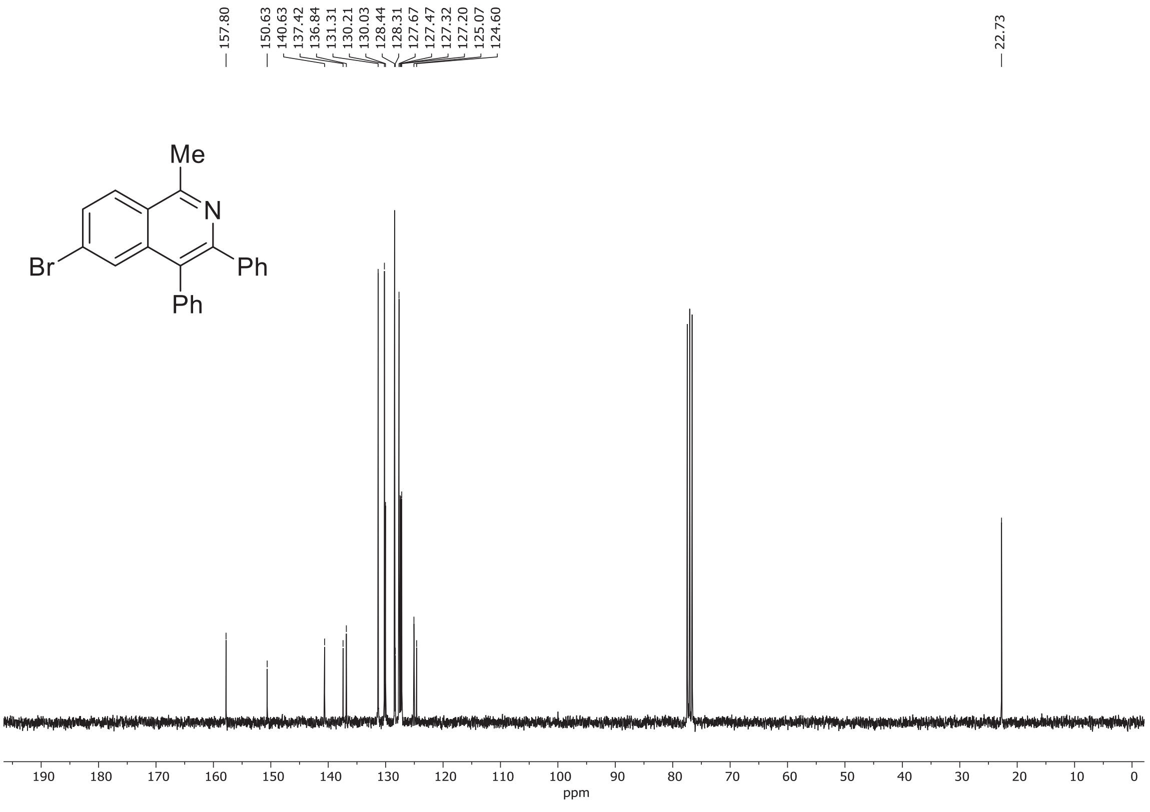

$^{1}$H NMR spectrum of **19** recorded on a Bruker Avance spectrometer (300 MHz, $CDCl_3$):

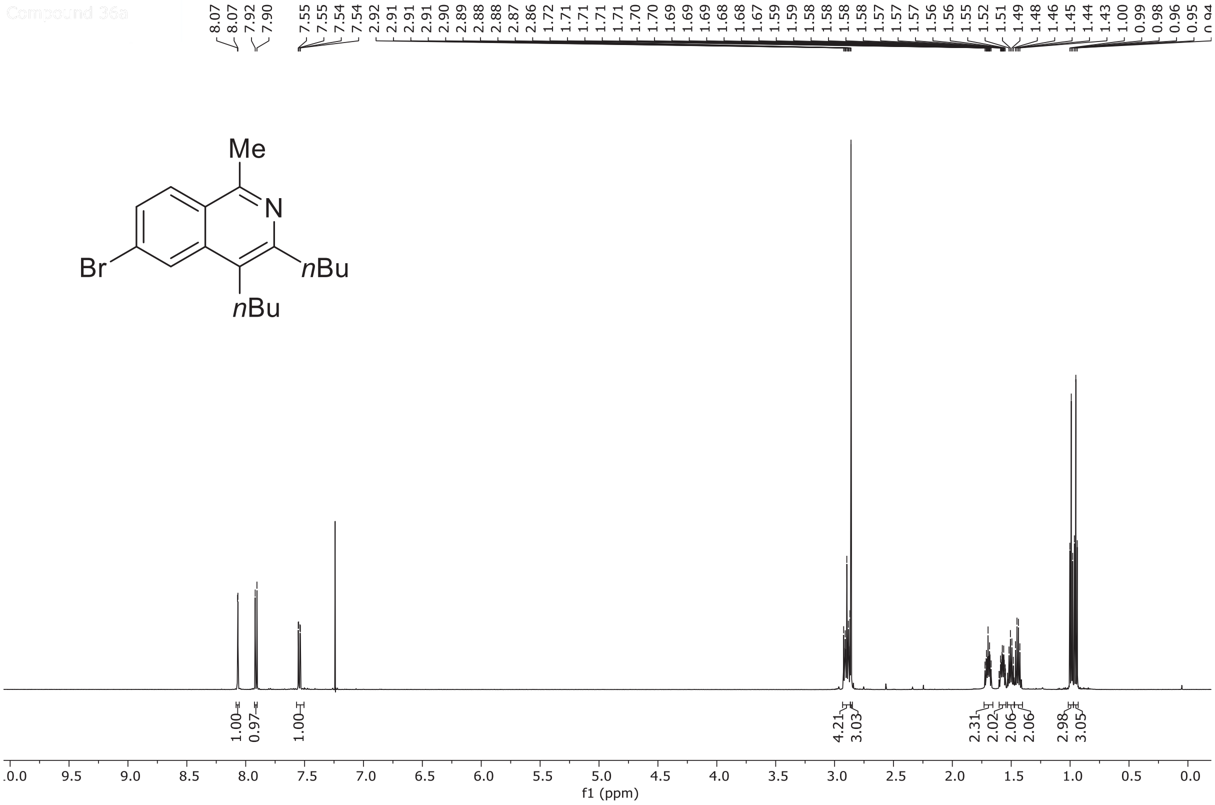


$^{1}$H NMR spectrum of **20** recorded on a Bruker Avance spectrometer (300 MHz, $CDCl_3$):

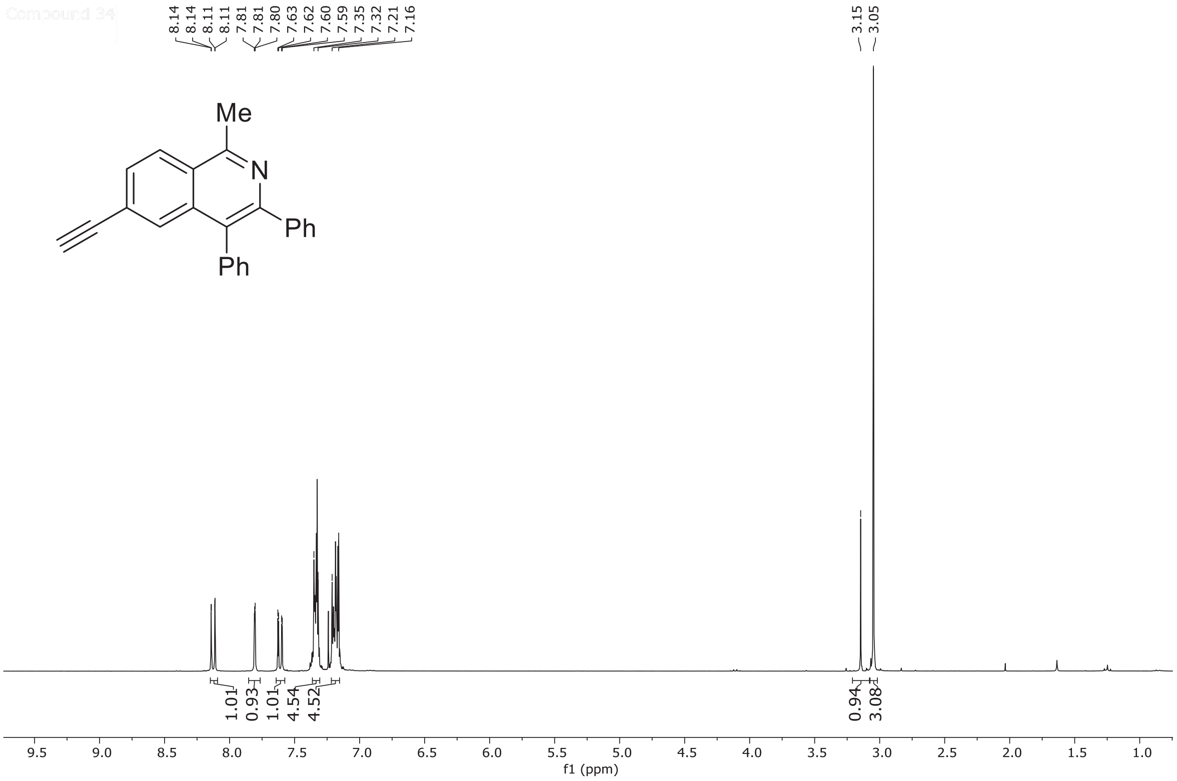

$^{13}C$ NMR spectrum of **20** recorded on a Bruker Avance spectrometer (75 MHz, $CDCl_3$):

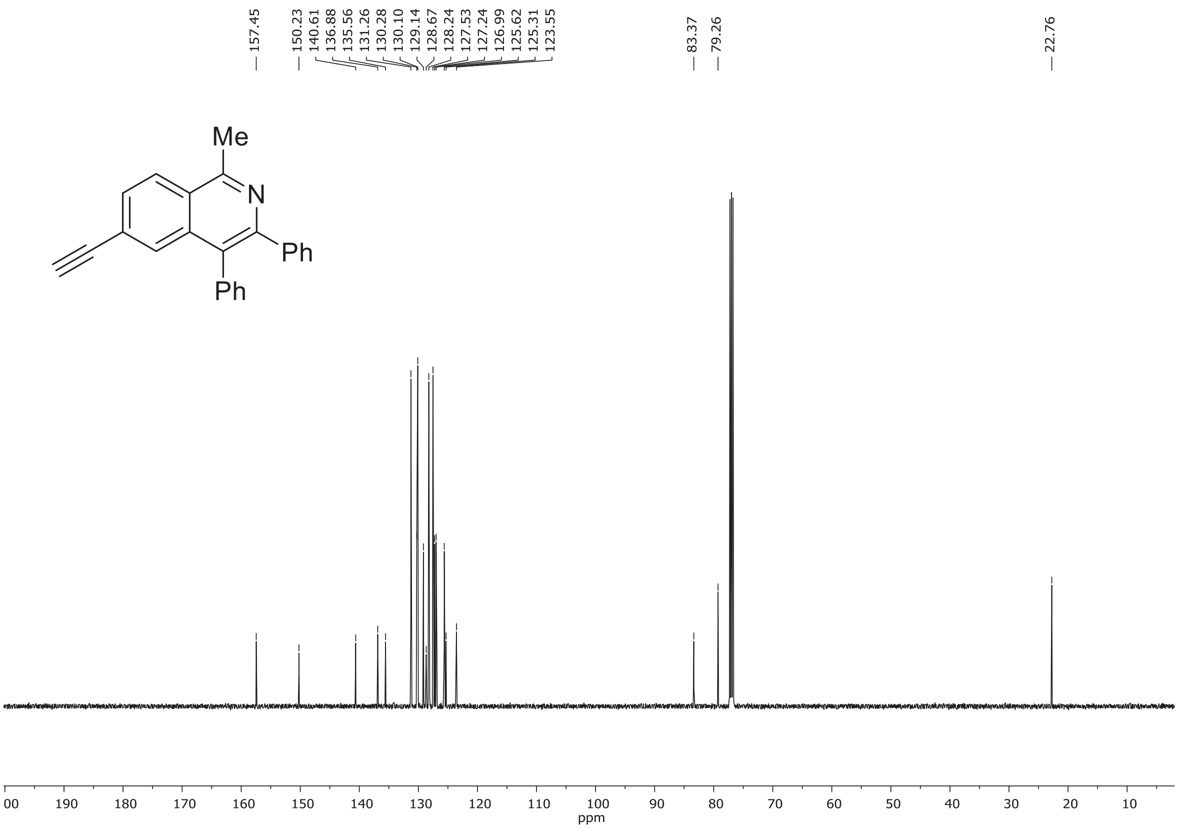


$^{1}H$ NMR spectrum of **21** recorded on a Bruker Avance spectrometer (300 MHz, $CDCl_3$):

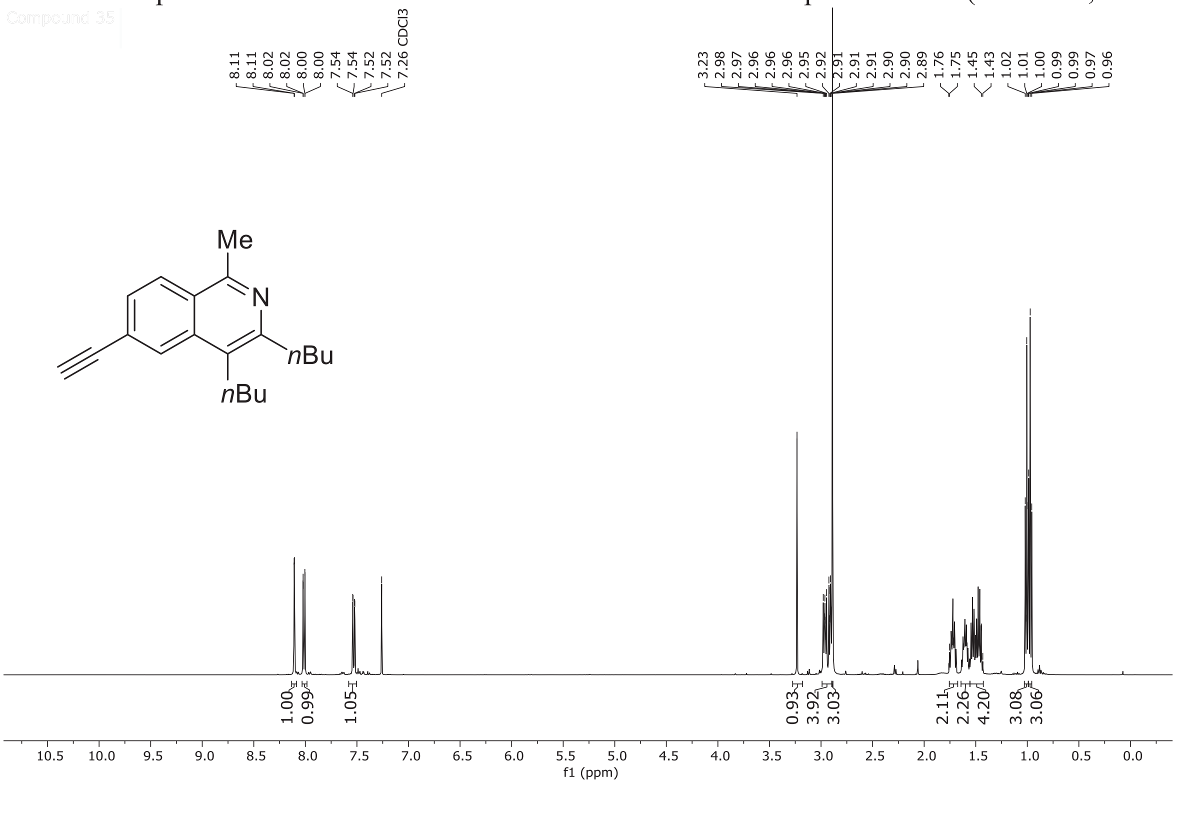

$^{13}C$ NMR spectrum of **21** recorded on a Bruker Avance spectrometer (75 MHz, $CDCl_3$):

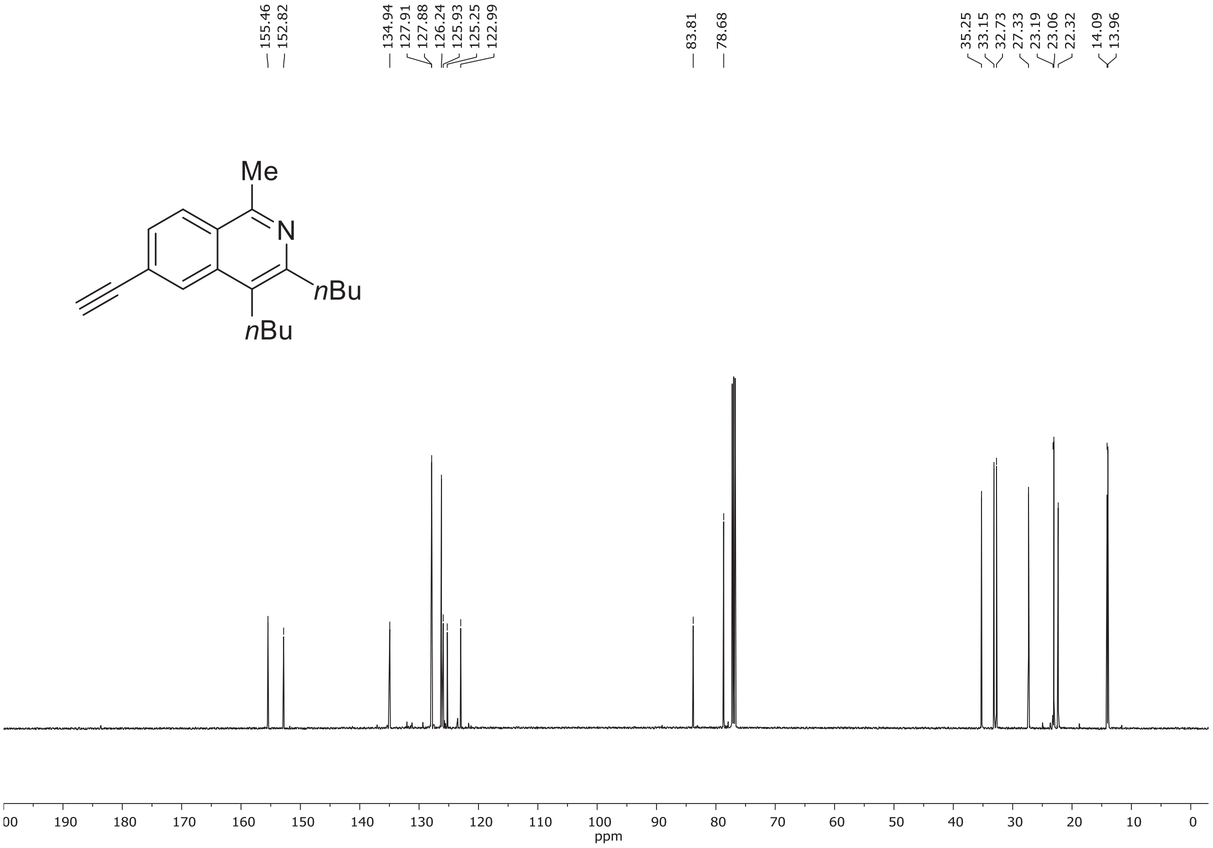


$^{1}H$ NMR spectrum of bromo intermediate recorded on a Bruker Avance spectrometer (300 MHz, $CDCl_3$):

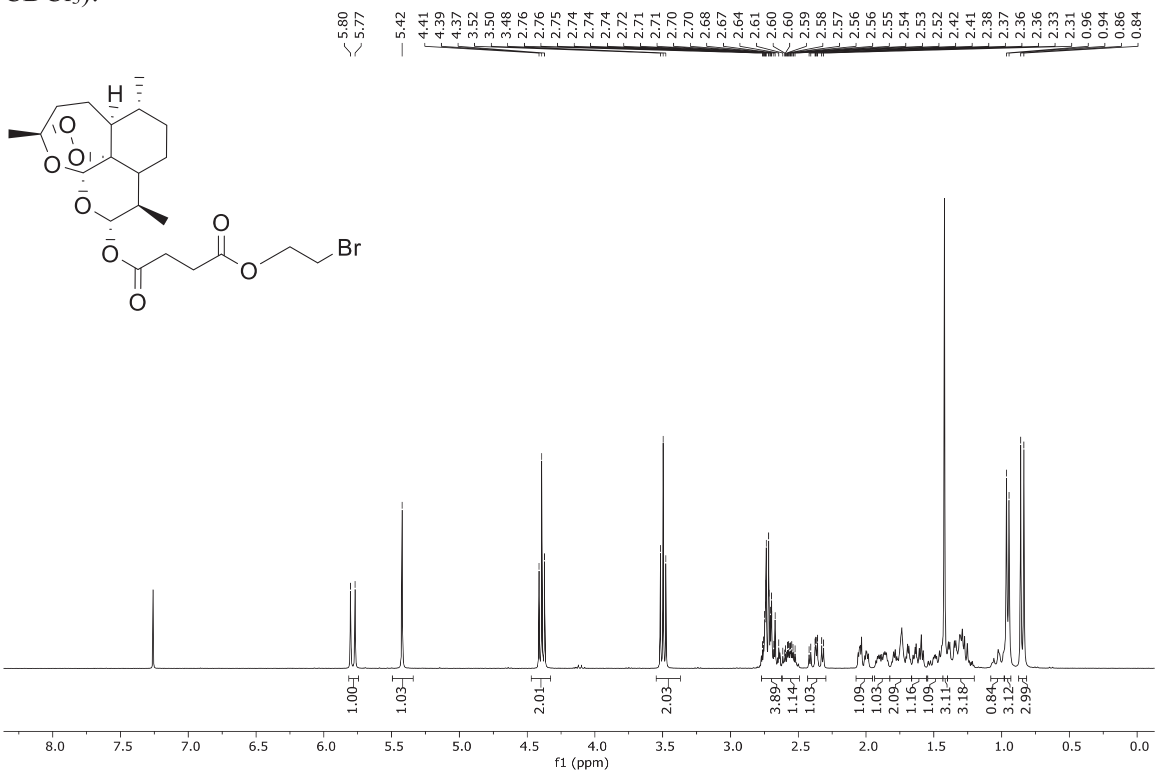

$^{1}$H NMR spectrum of **22** recorded on a Bruker Avance spectrometer (300 MHz, $CDCl_3$):

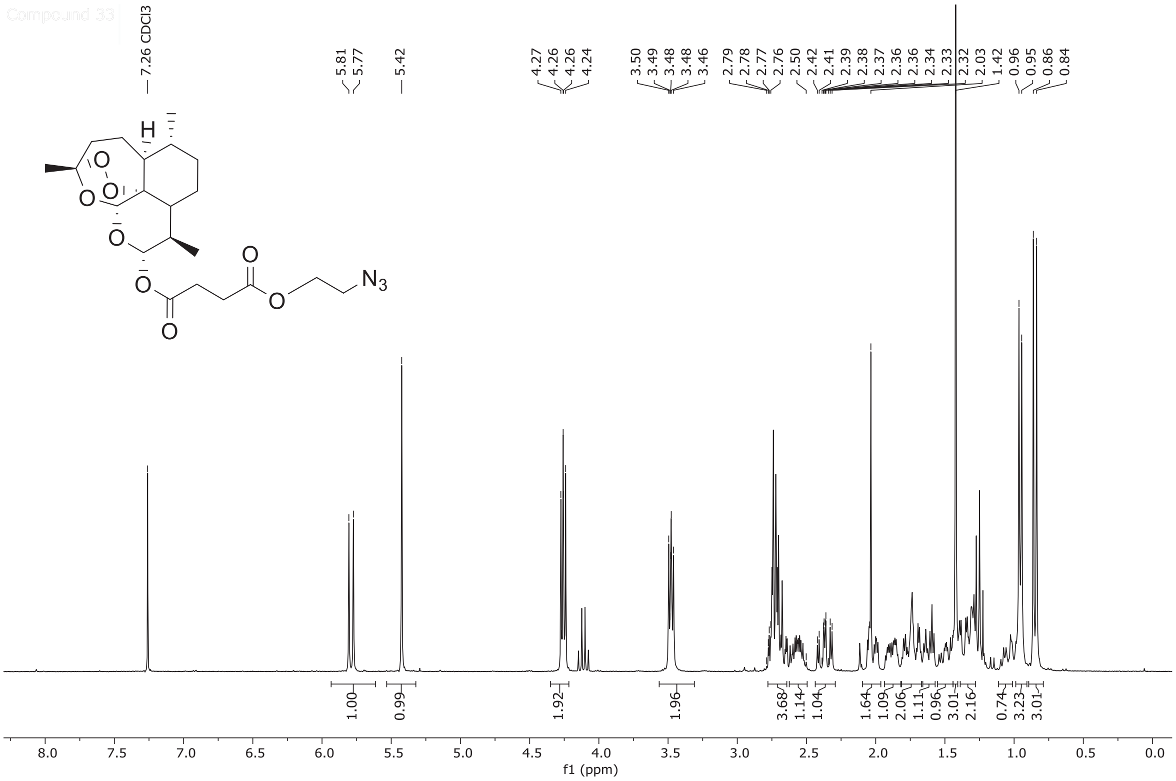


$^{13}$C NMR spectrum of bromo intermediate recorded on a Bruker Avance spectrometer (125 MHz, $CDCl_3$):

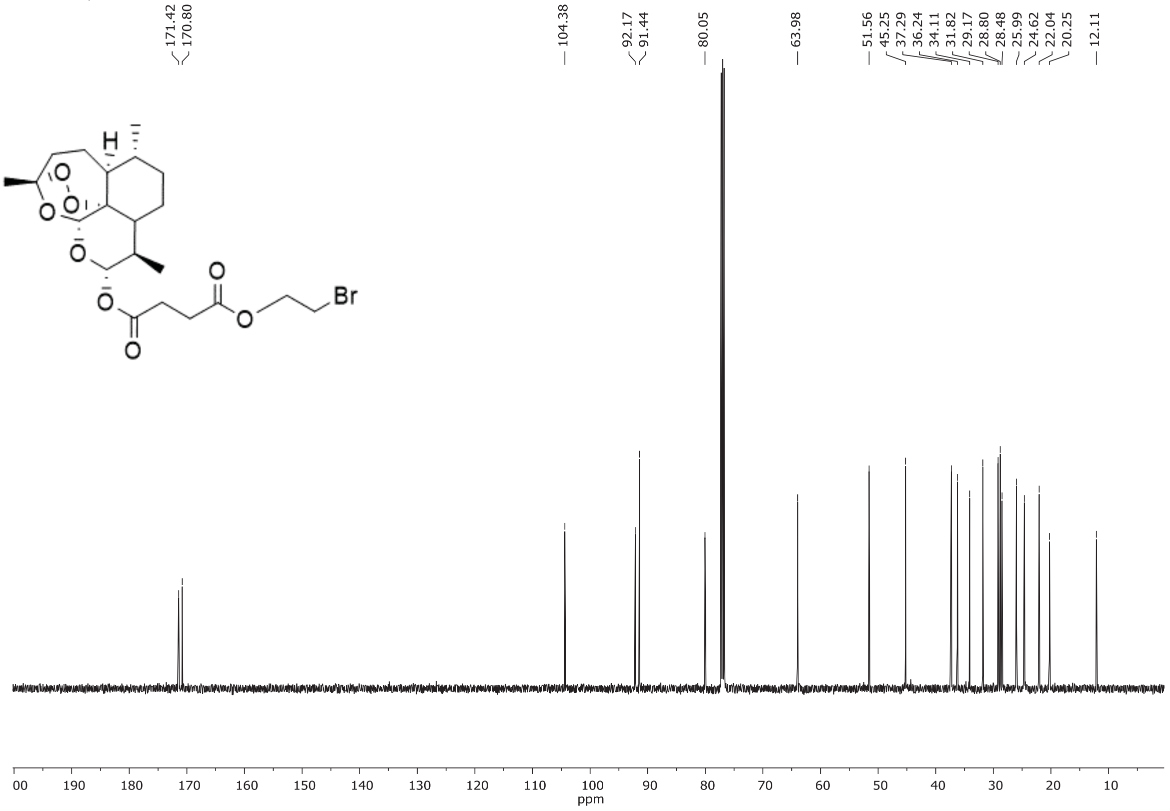

$^{13}C$ NMR spectrum of **22** recorded on a Bruker Avance spectrometer (125 MHz, $CDCl_3$):

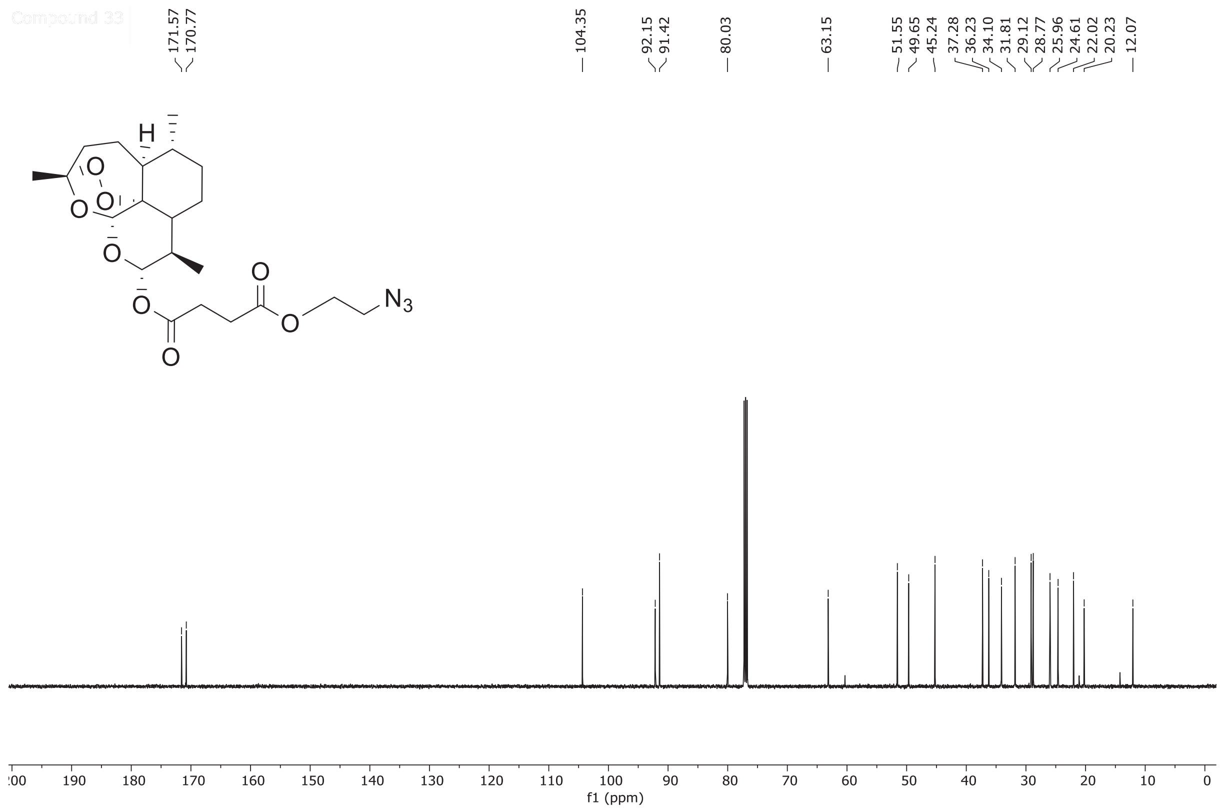


$^{1}H$ NMR spectrum of hybrid **1** recorded on a Bruker Avance spectrometer (300 MHz, $CDCl_3$):

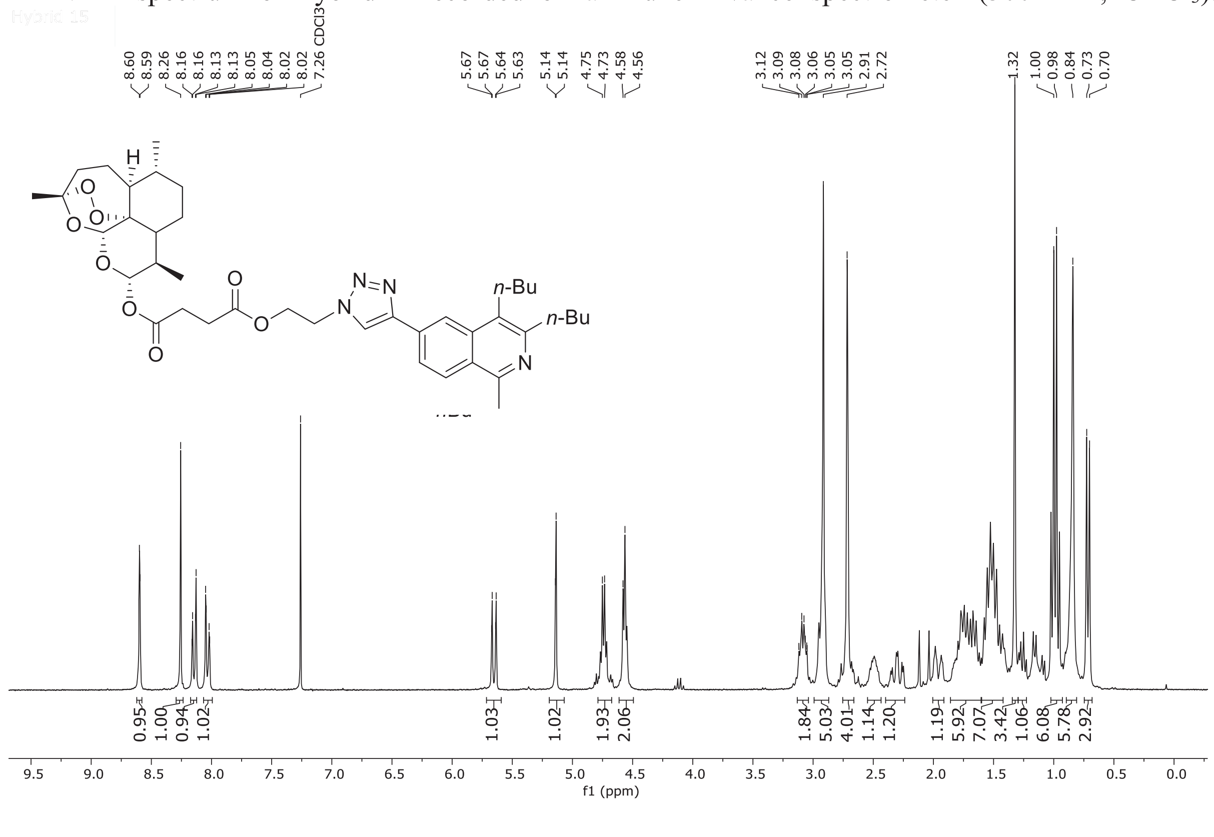

$^{13}C$ NMR spectrum of hybrid **1** recorded on a Bruker Avance spectrometer (75 MHz, $CDCl_3$):

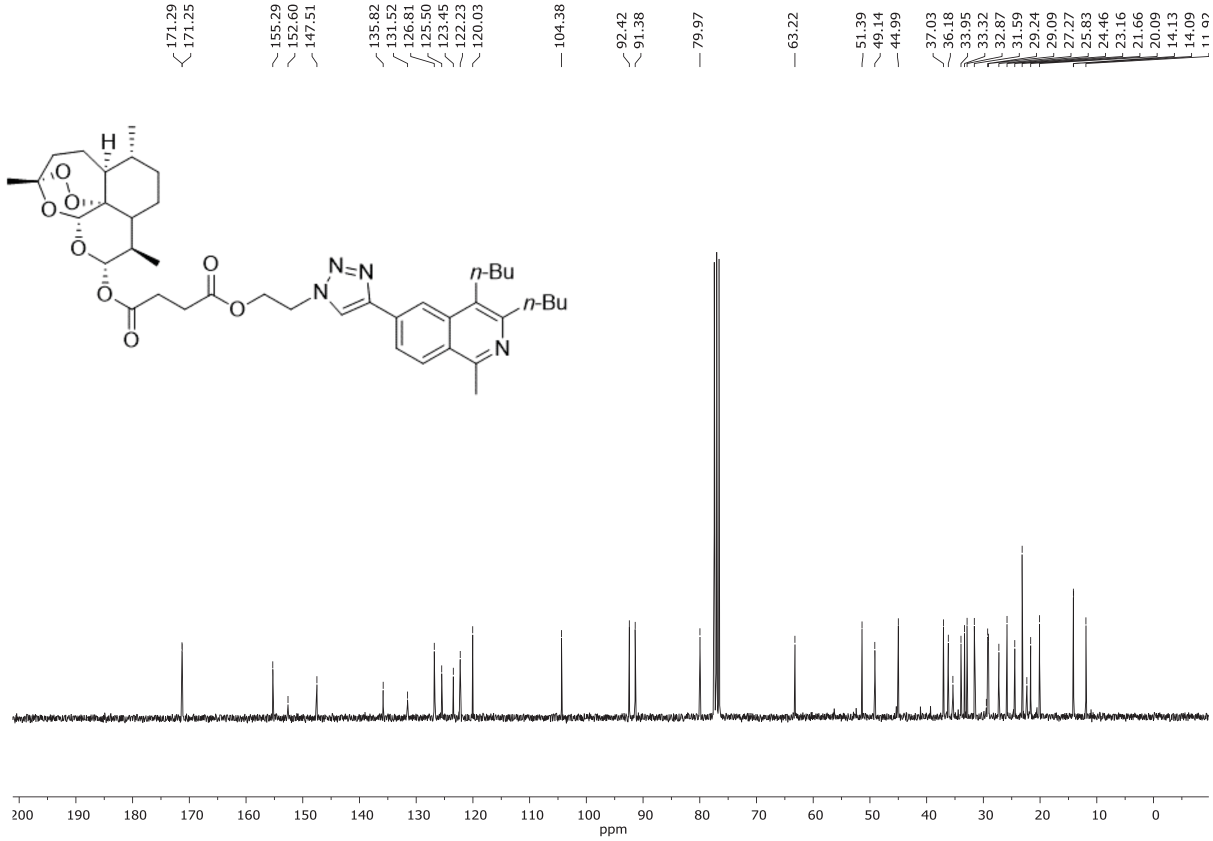


$^{1}H$ NMR spectrum of hybrid **2** recorded on a Bruker Avance spectrometer (300 MHz, $CDCl_3$):

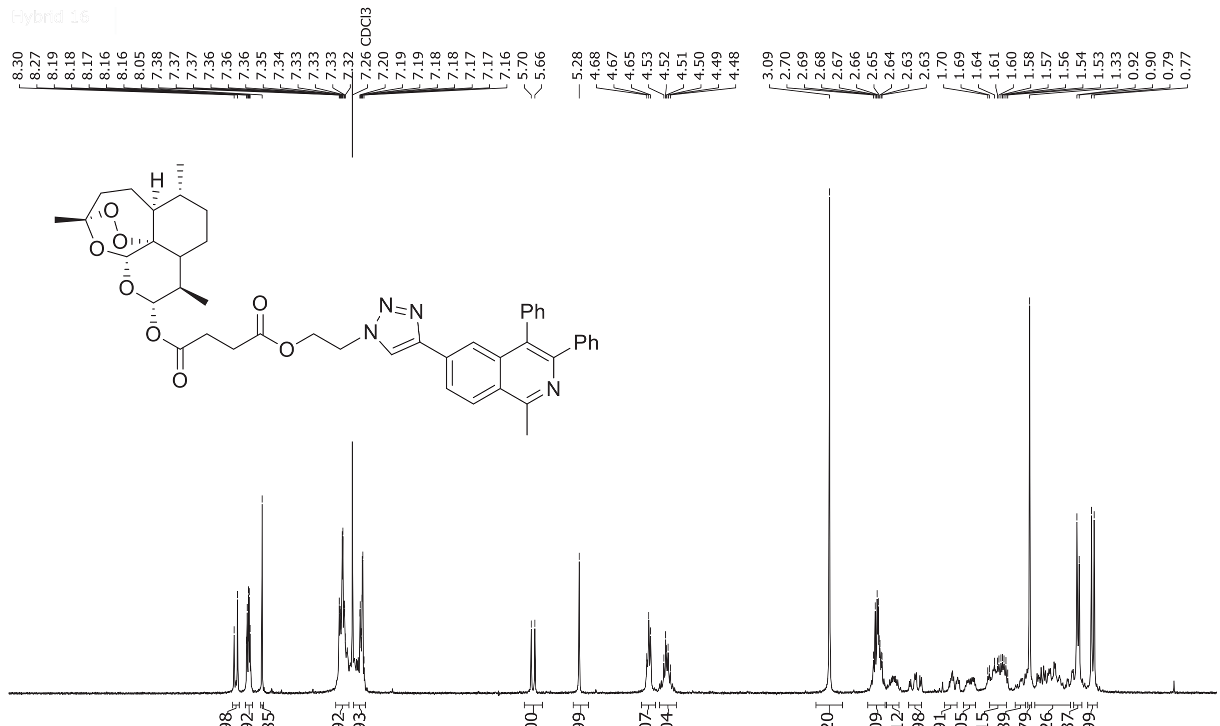

$^{13}$C NMR spectrum of hybrid **2** recorded on a Bruker Avance spectrometer (75 MHz, $CDCl_3$):

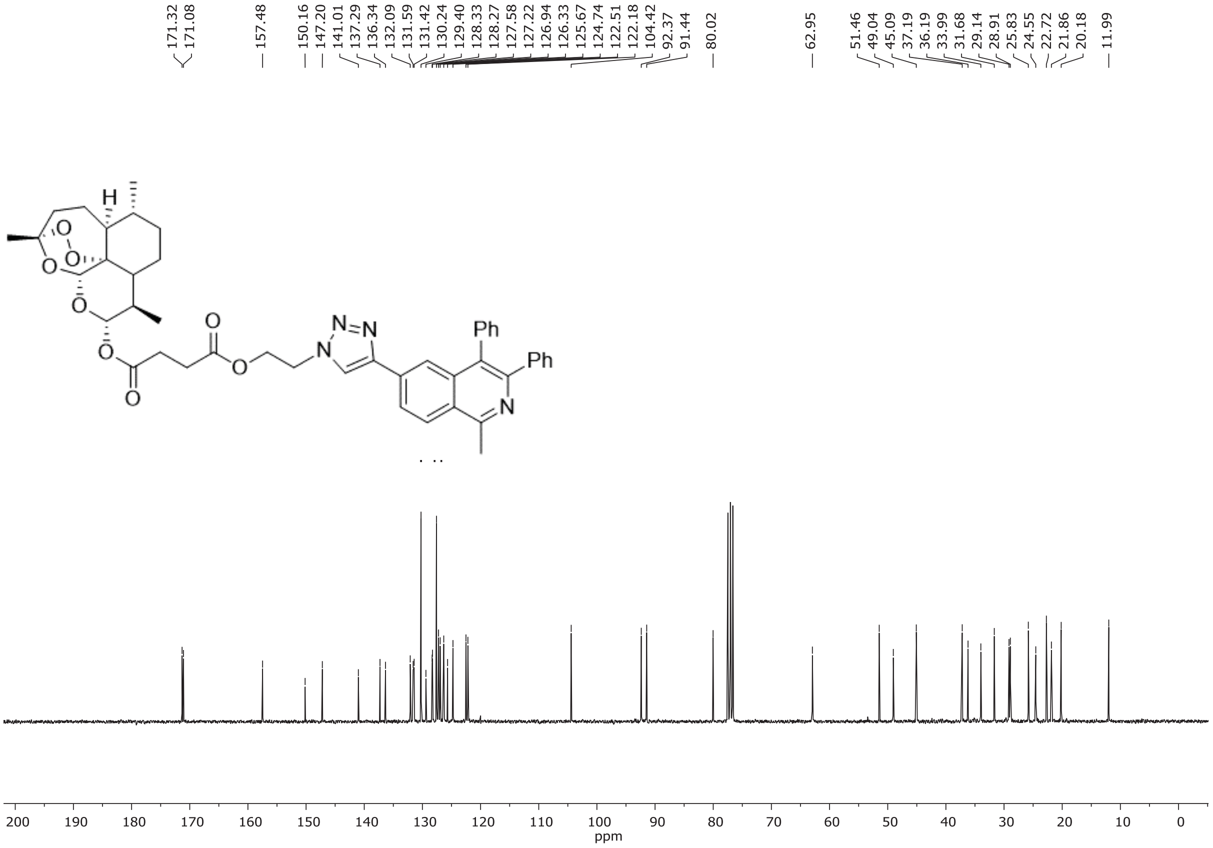


$^{1}$H NMR spectrum of hybrid **3** recorded on a Bruker Avance spectrometer (300 MHz, $CDCl_3$):

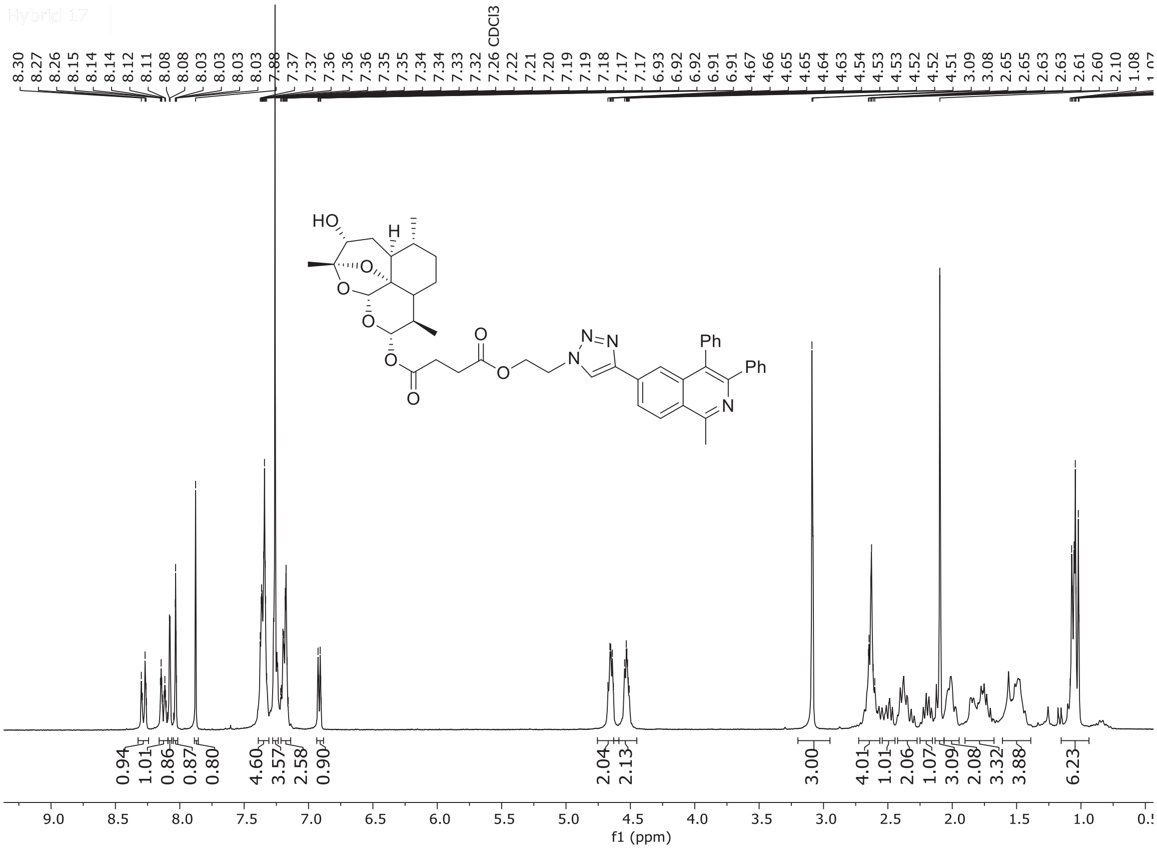

$^{13}C$ NMR spectrum of hybrid **3** recorded on a Bruker Avance spectrometer (75 MHz, $CDCl_3$):

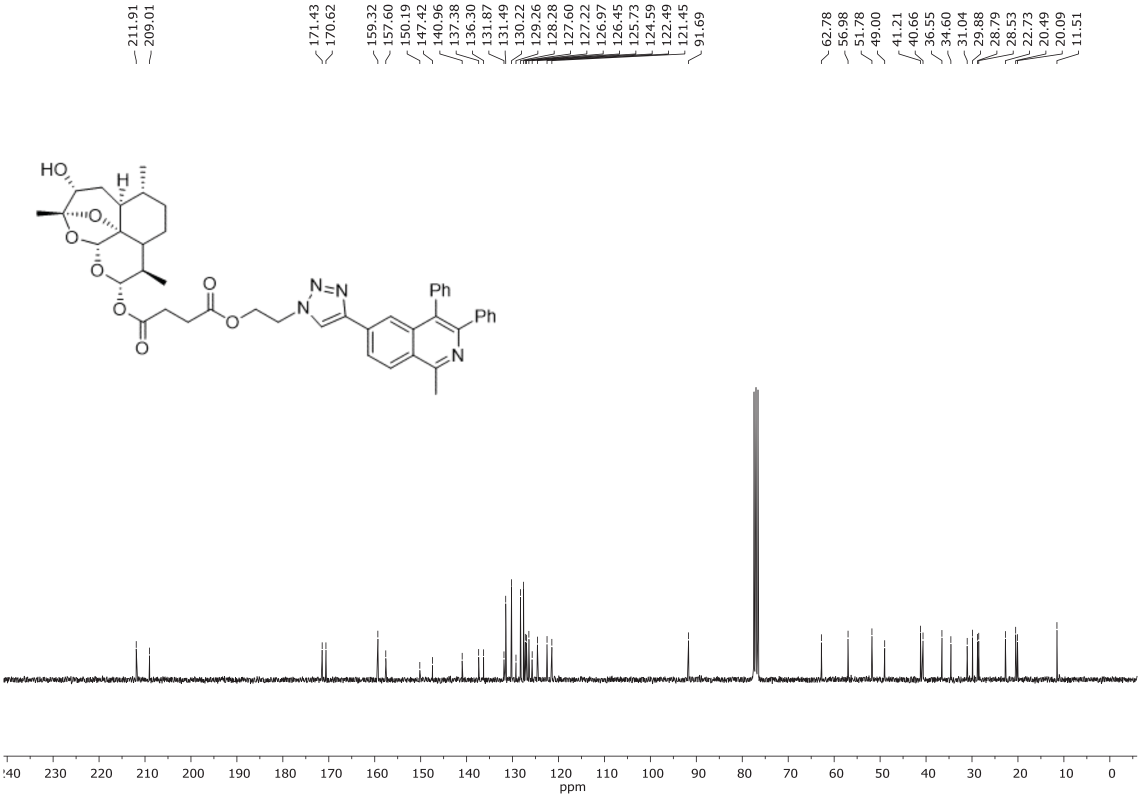


$^{1}H$ NMR spectrum of **4,7-dichloroquinoline** recorded on a Bruker Avance spectrometer (300 MHz, $CDCl_3$):

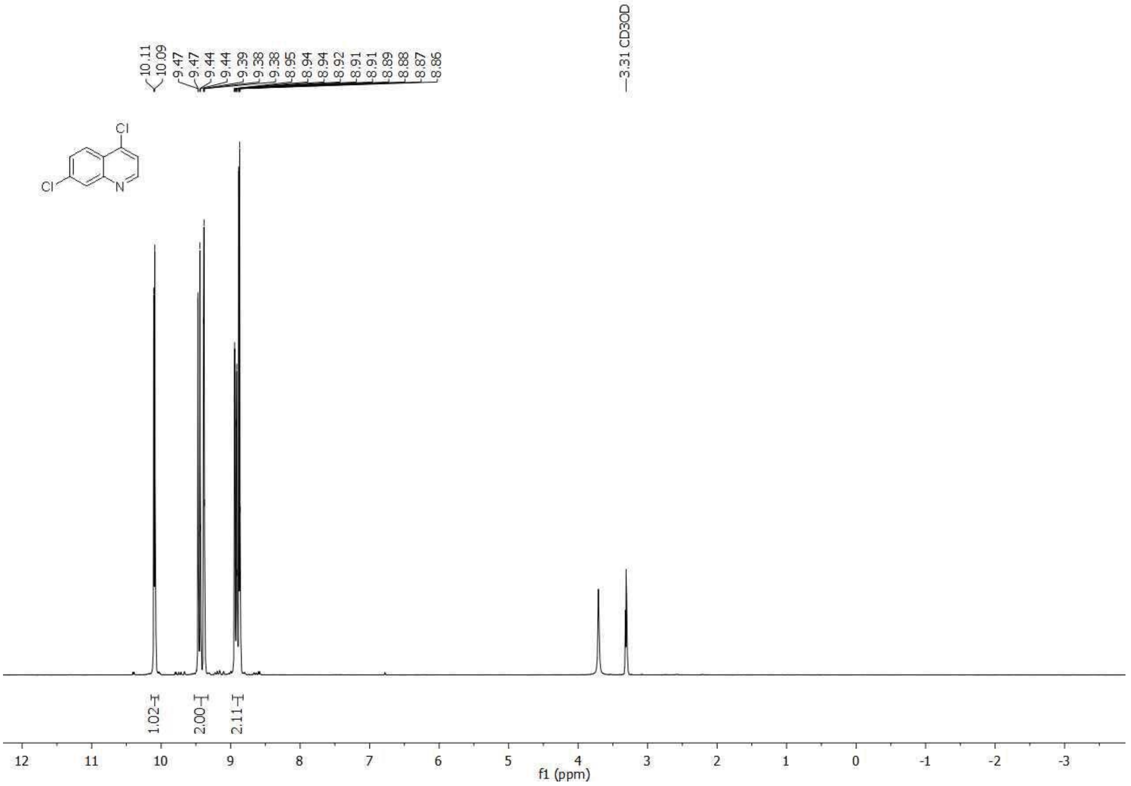

$^{13}C$ NMR spectrum of **4,7-dichloroquinoline** recorded on a Bruker Avance spectrometer (75 MHz, $CDCl_3$):

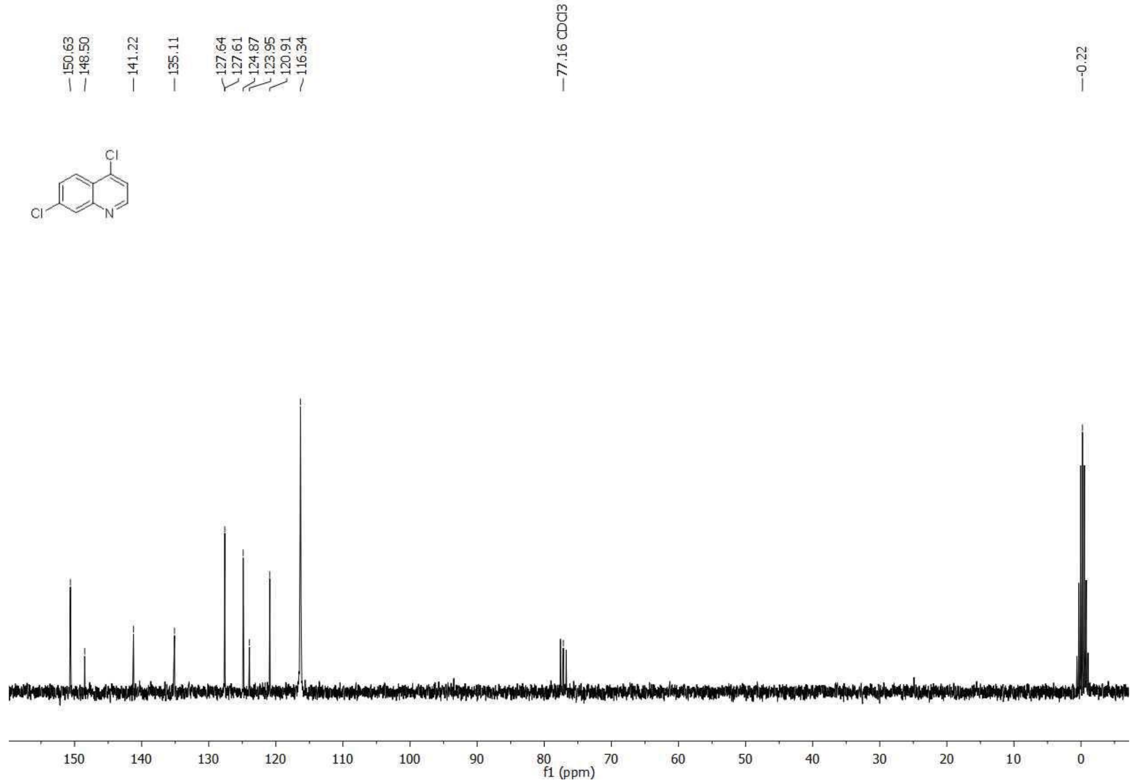


$^1H$ NMR spectrum of **33** recorded on a Bruker Avance spectrometer (300 MHz, $CDCl_3$):

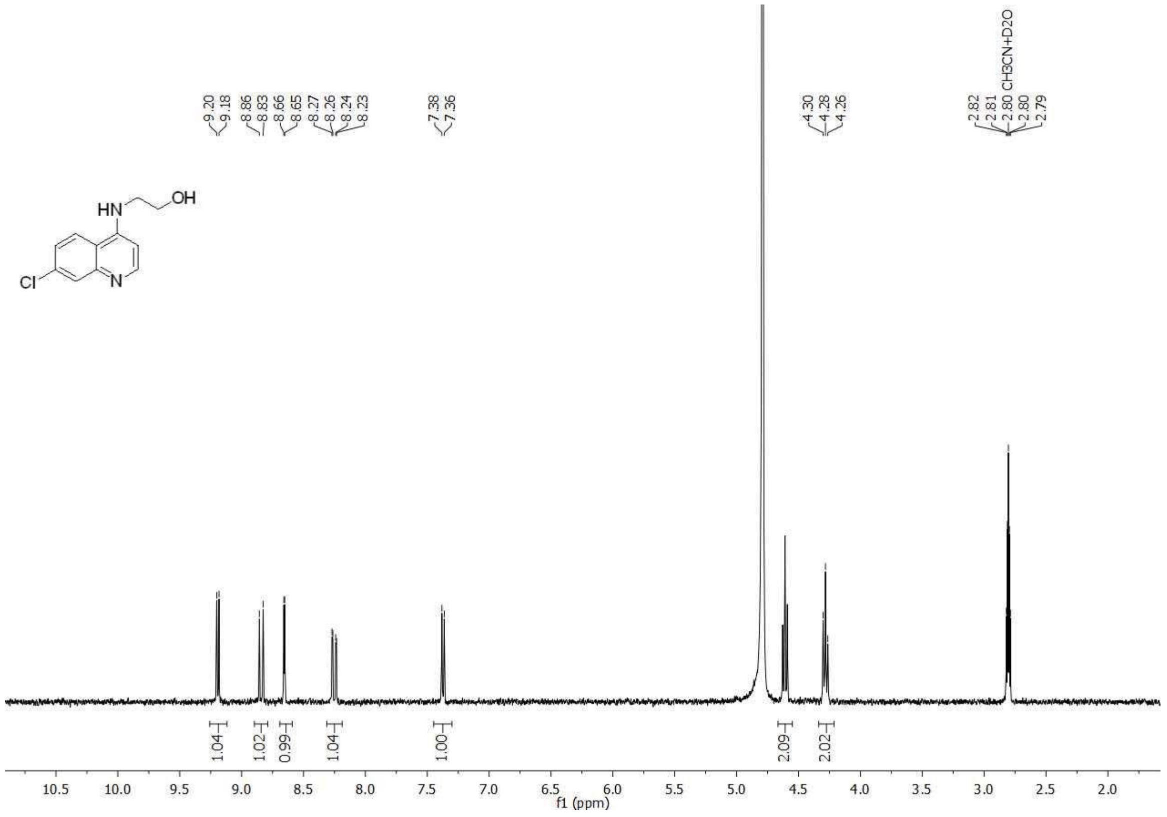

$^{13}C$ NMR spectrum of **33** recorded on a Bruker Avance spectrometer (75 MHz, $CDCl_3$):

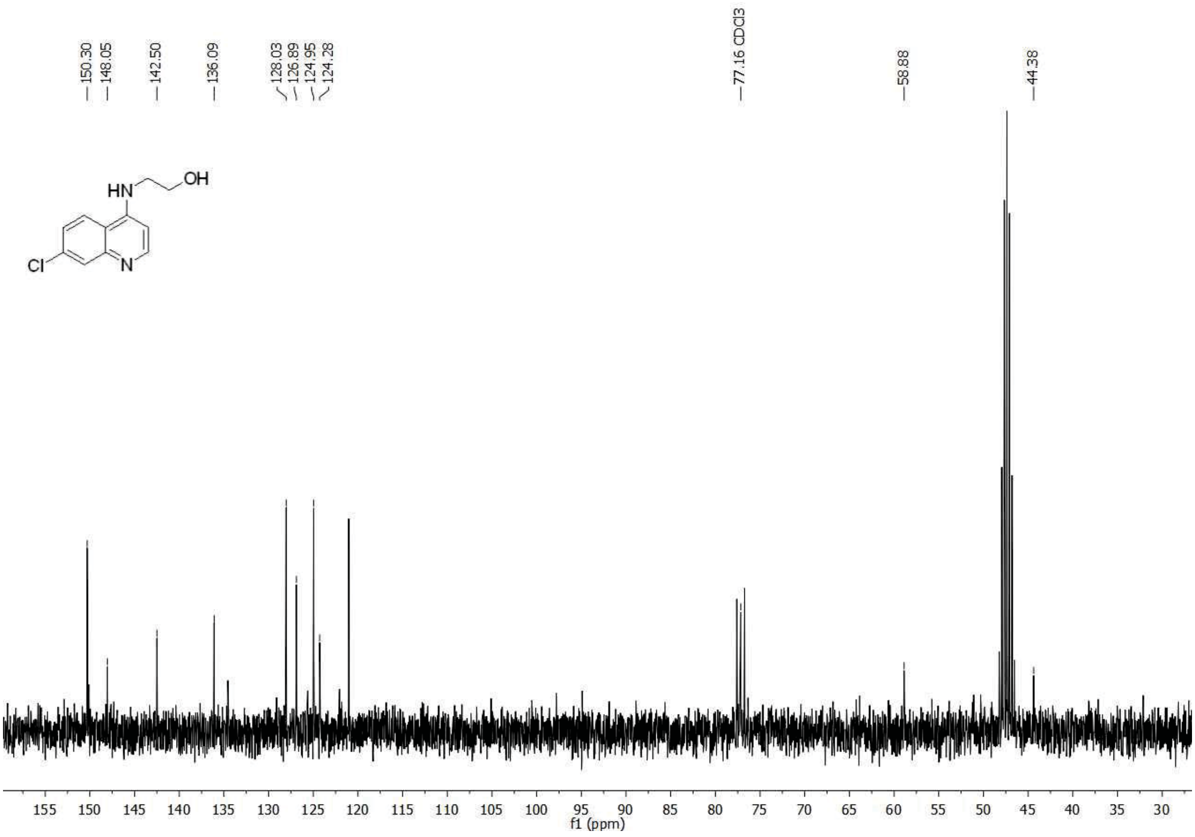


$^{1}H$ NMR spectrum of **36** recorded on a Bruker Avance spectrometer (300 MHz, $CDCl_3$):

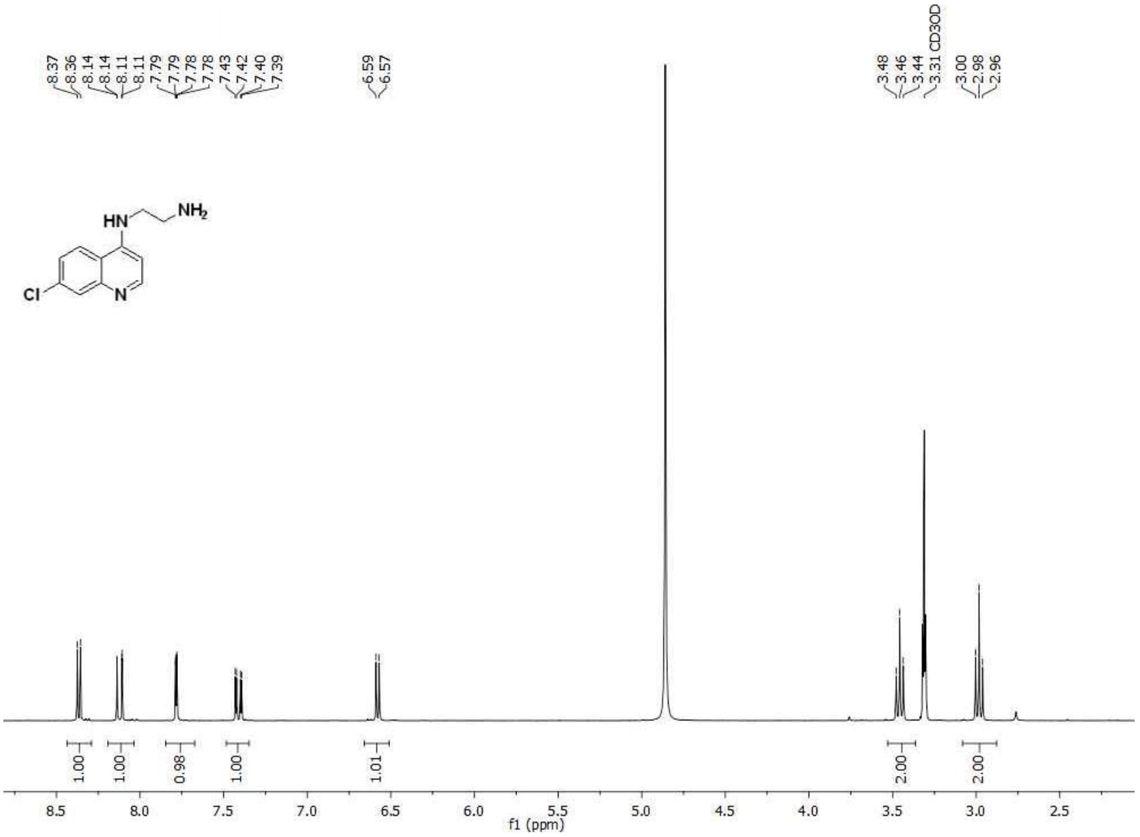

$^{13}C$ NMR spectrum of **36** recorded on a Bruker Avance spectrometer (75 MHz, $CDCl_3$):

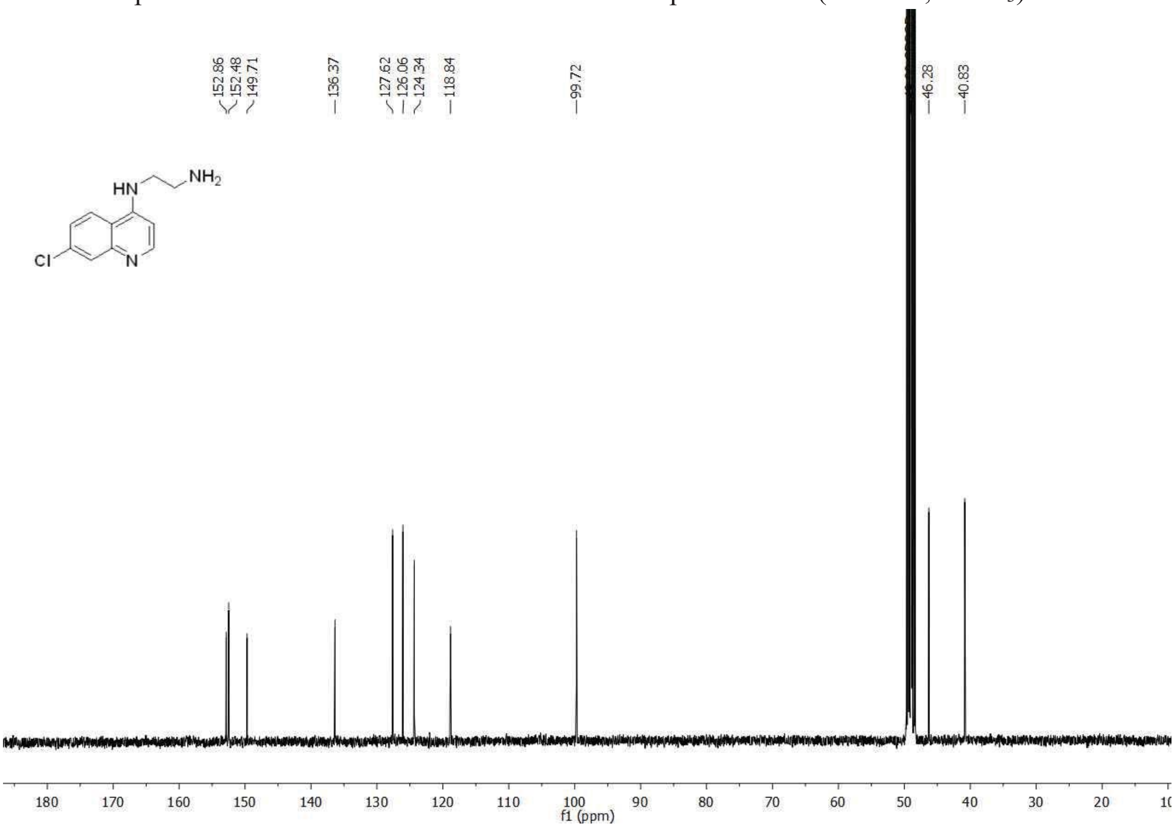


ESI mass spectrum of hybrid **36** recorded on a Bruker micrOTOF II focus TOF MS-spectrometer:

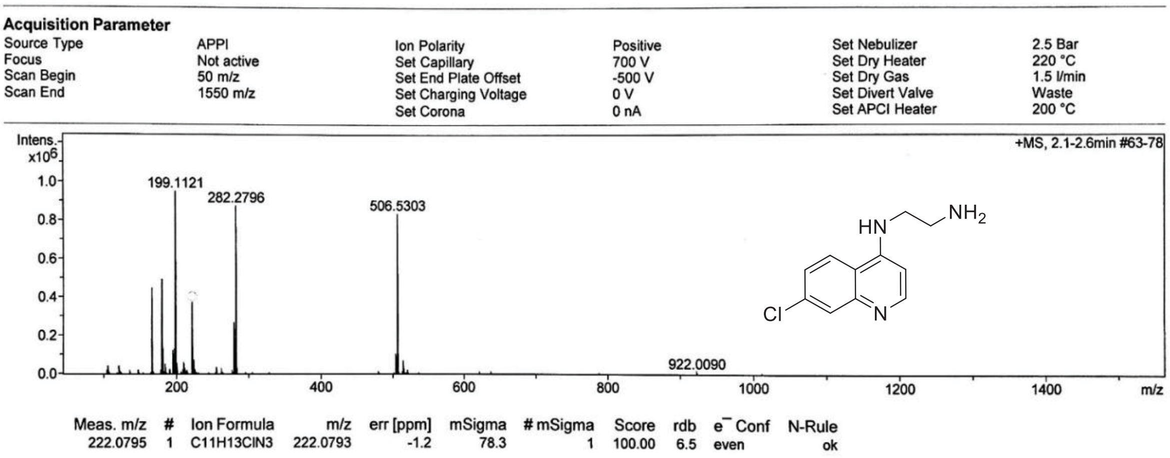

$^{1}$H NMR spectrum of **37** recorded on a Bruker Avance spectrometer (400 MHz, $CDCl_3$):

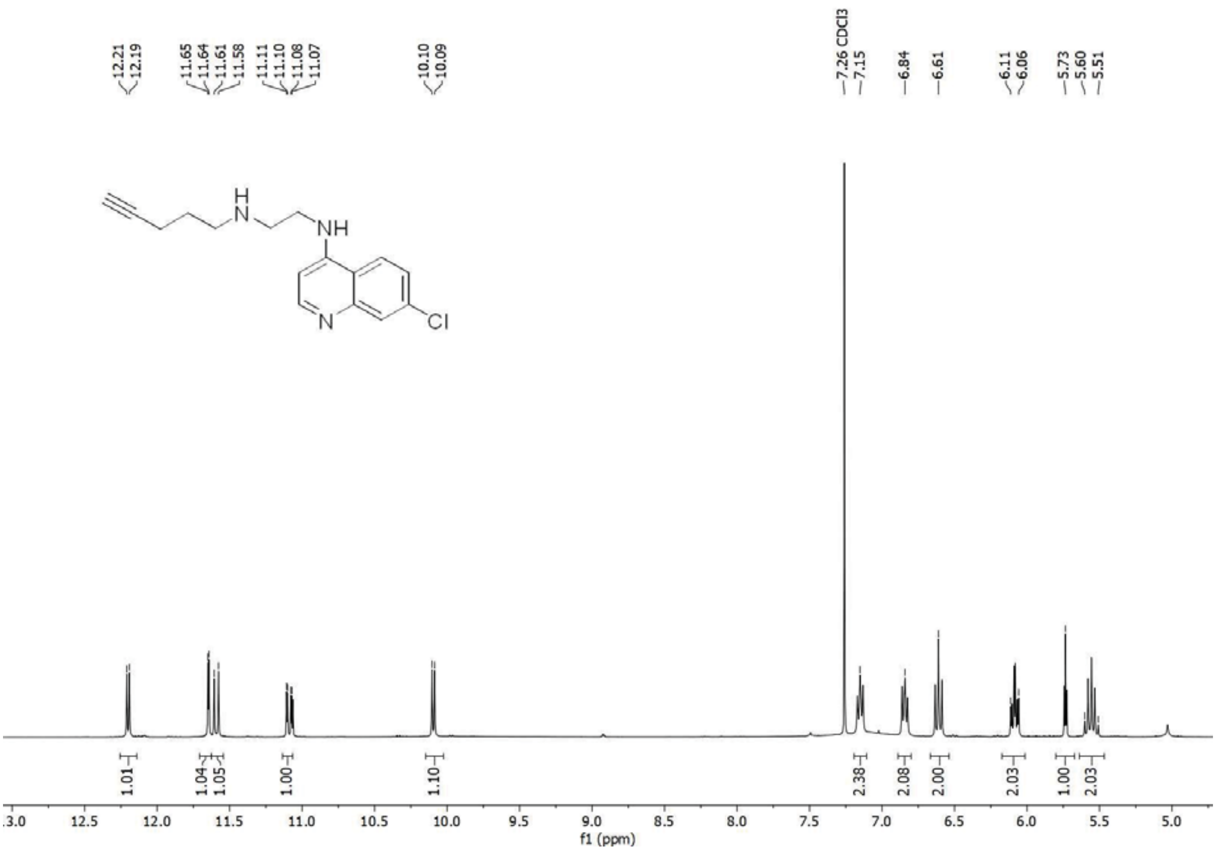


$^{13}$C NMR spectrum of **37** recorded on a Bruker Avance spectrometer (100 MHz, $CDCl_3$):

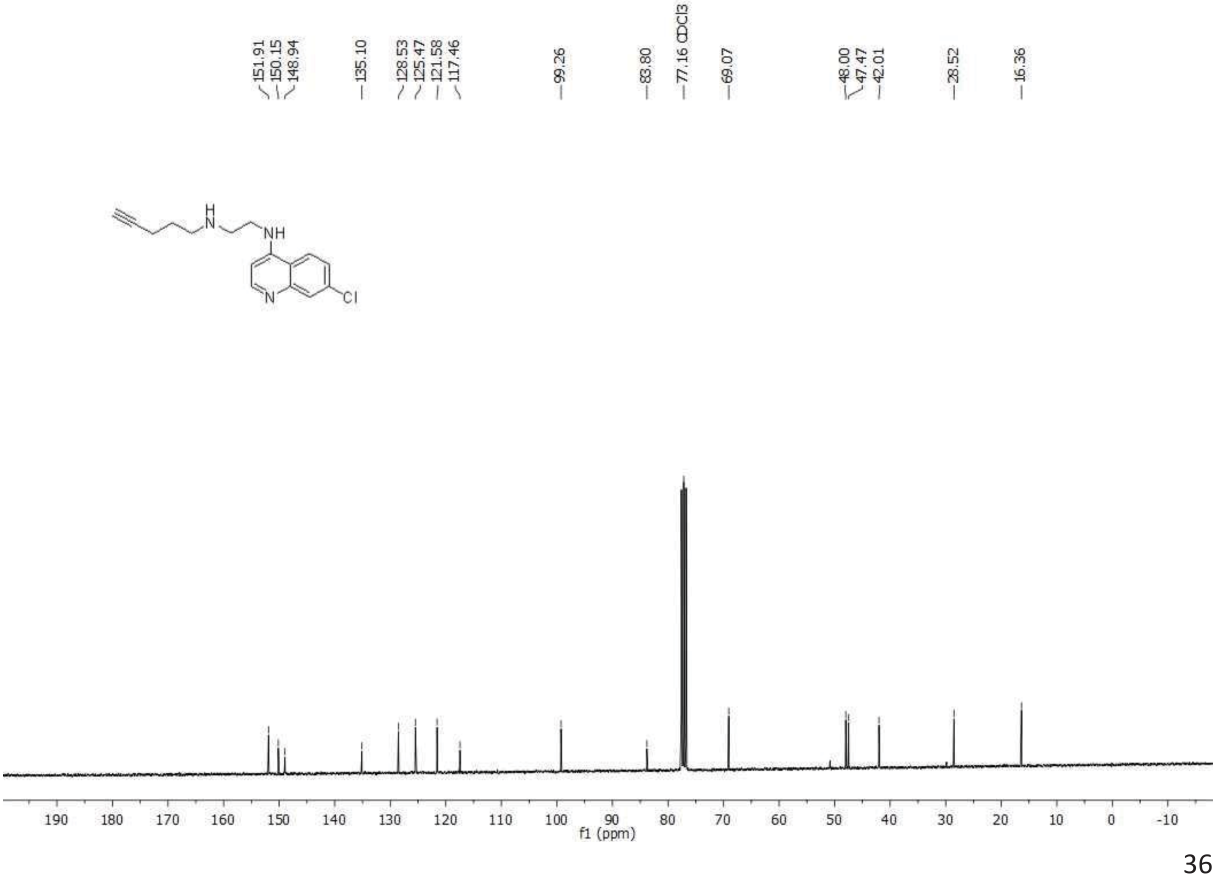

ESI mass spectrum of **37** recorded on a Bruker micrOTOF II focus TOF MS-spectrometer:

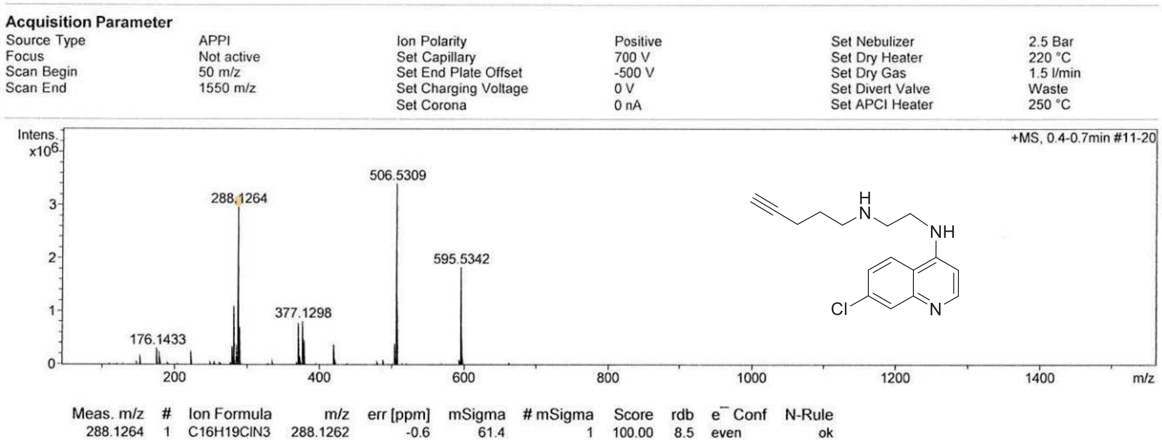


$^{1}$H NMR spectrum of **34** recorded on a Bruker Avance spectrometer (300 MHz, $CDCl_3$):

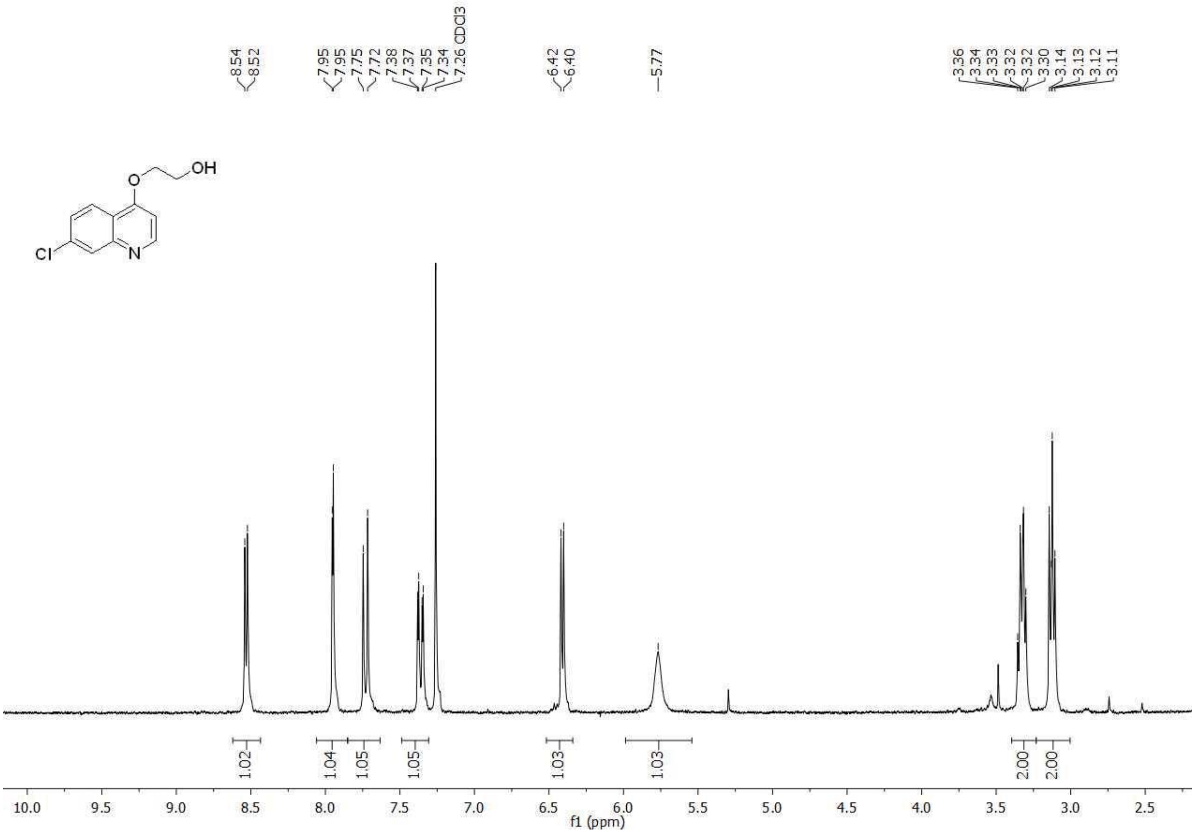

<sup></sup>$^{1}$H NMR spectrum of **27** recorded on a Bruker Avance spectrometer (300 MHz, $CDCl_3$):

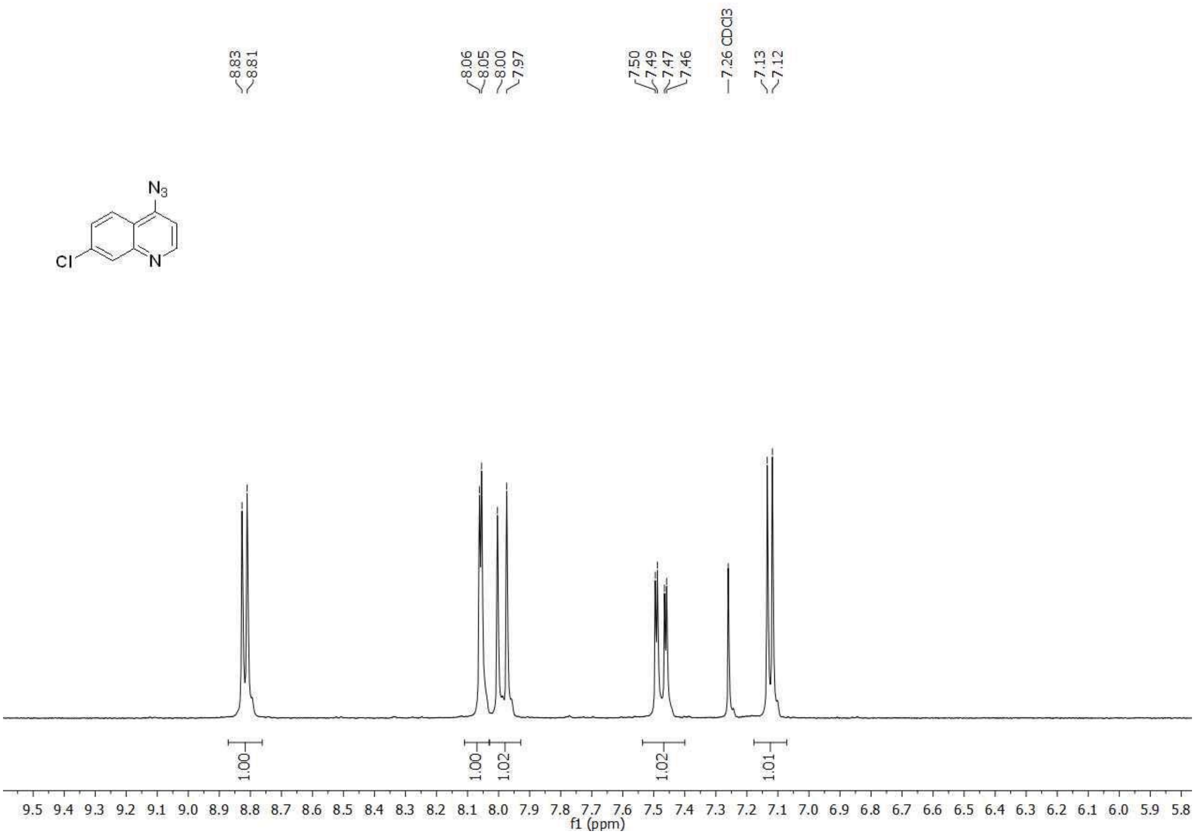


FTIR spectrum of **27** on a Agilent Resolution spectrometer

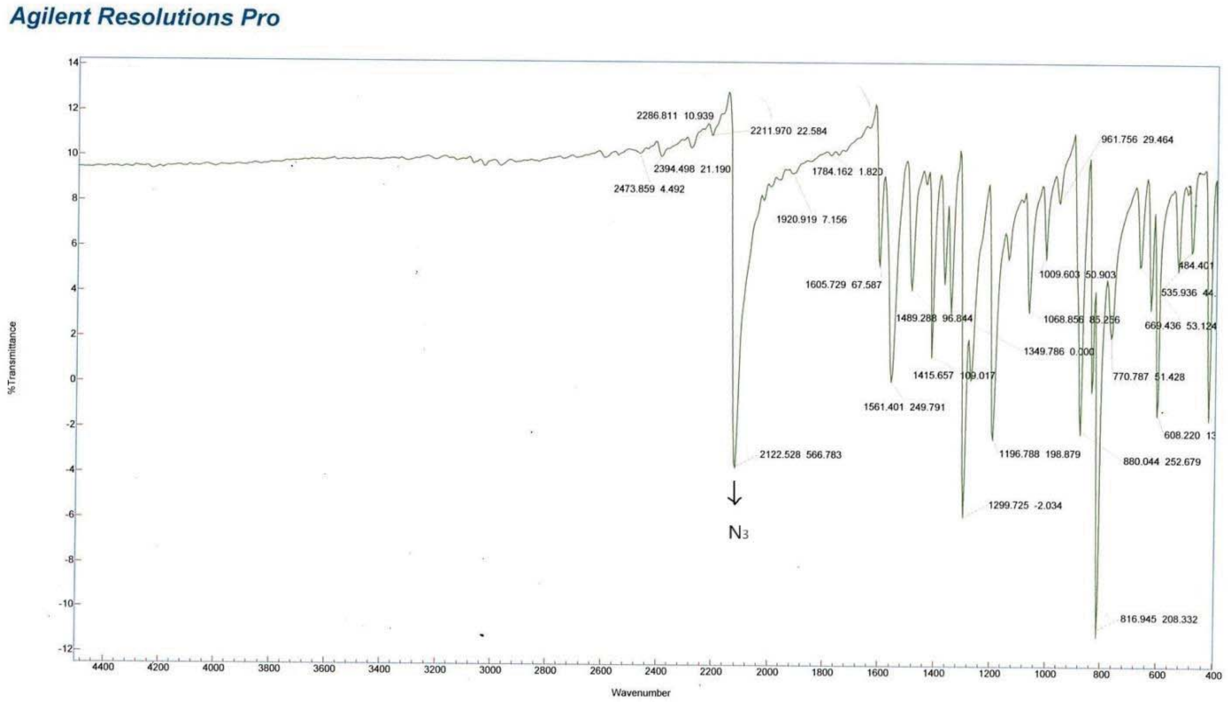

$^{1}H$ NMR spectrum of **23** recorded on a Bruker Avance spectrometer (300 MHz, $CDCl_3$):

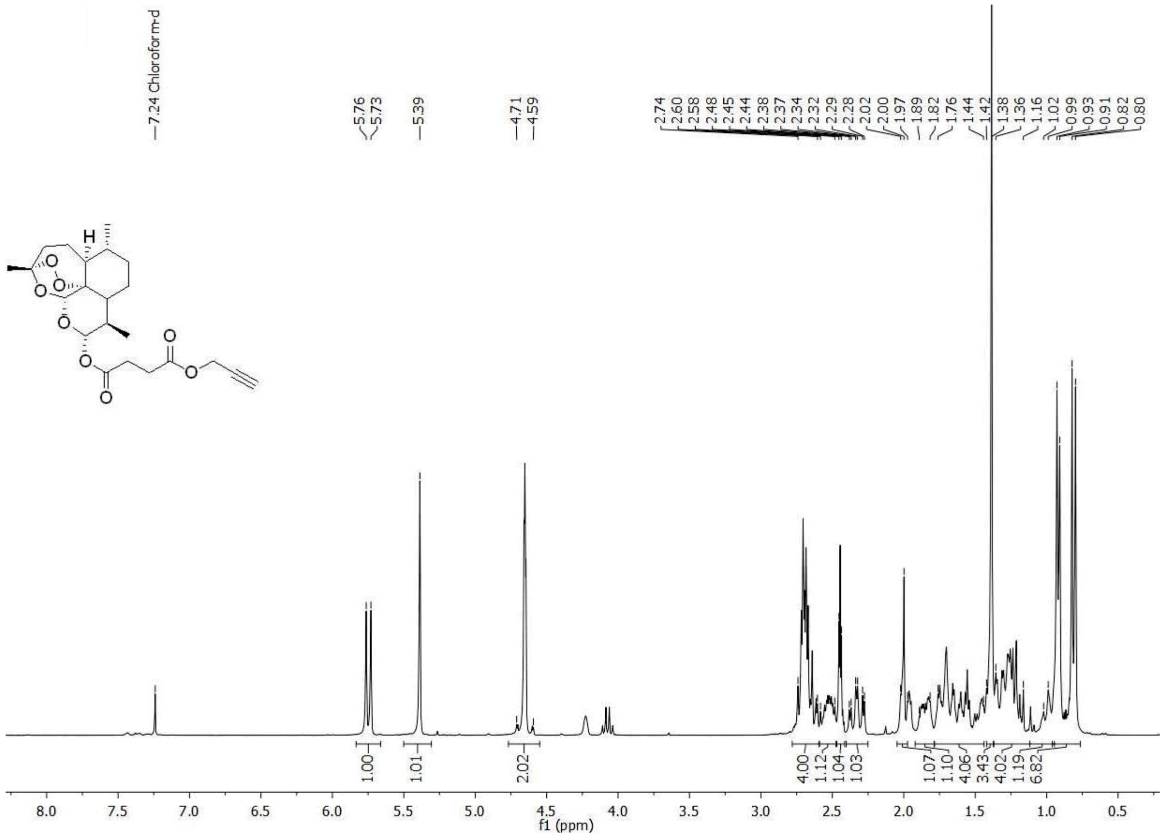

$^{13}C$ NMR spectrum of **23** recorded on a Bruker Avance spectrometer (100 MHz, $CDCl_3$):

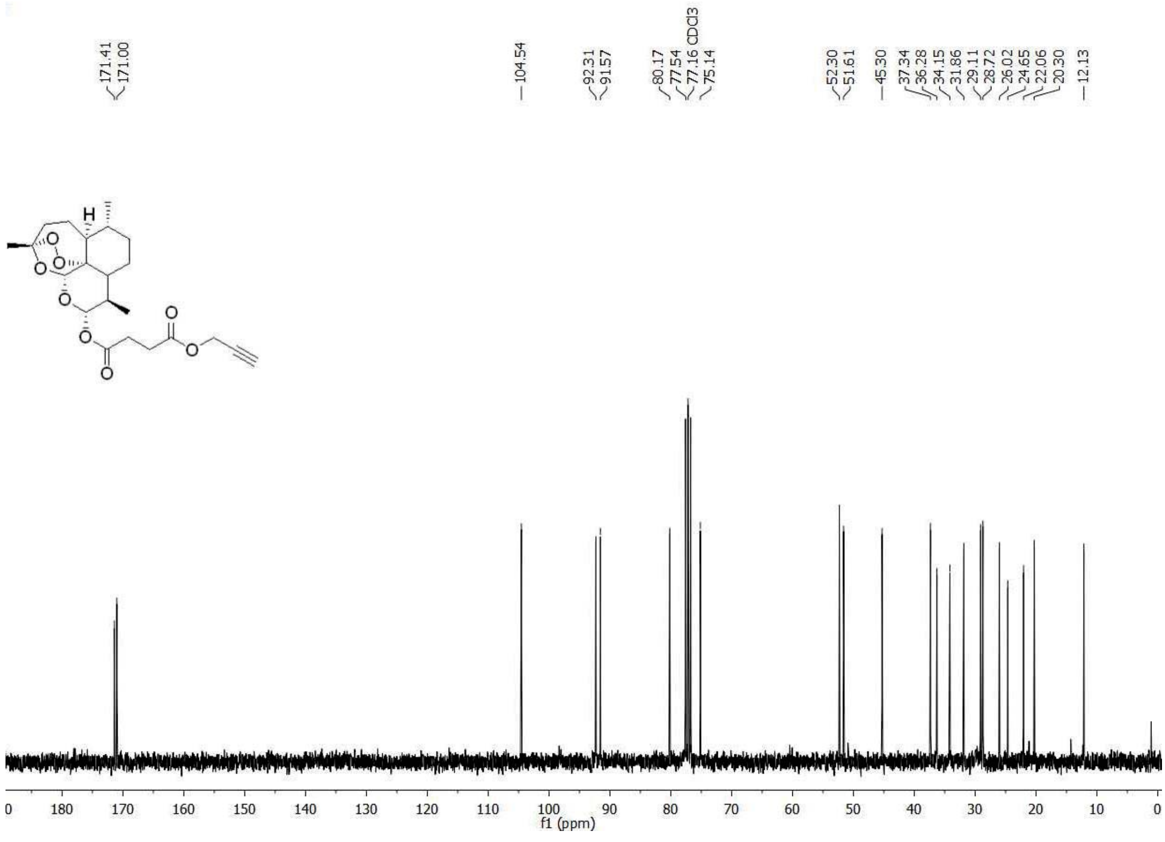

<sup></sup>

$^{1}$H NMR spectrum of **24** recorded on a Bruker Avance spectrometer (300 MHz, $CDCl_3$):

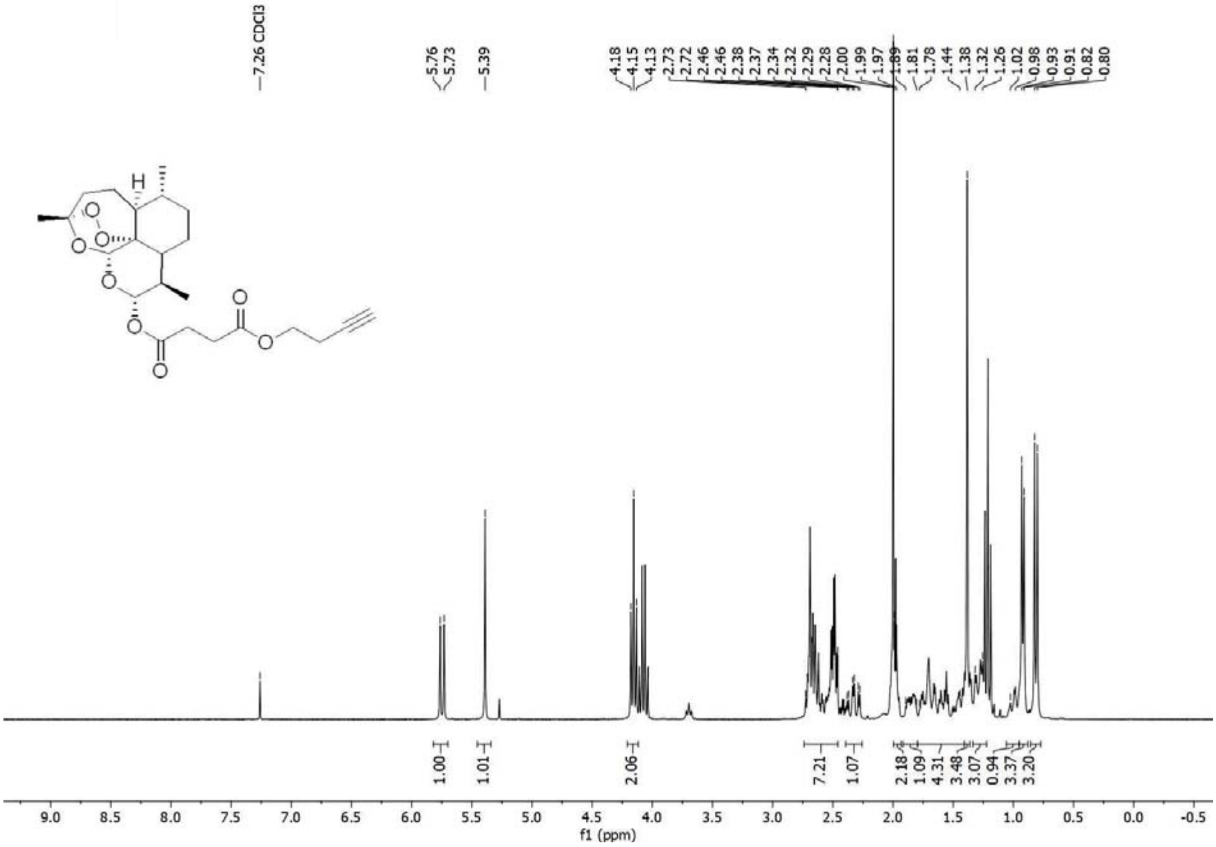

$^{13}$C NMR spectrum of **24** recorded on a Bruker Avance spectrometer (100 MHz, $CDCl_3$):

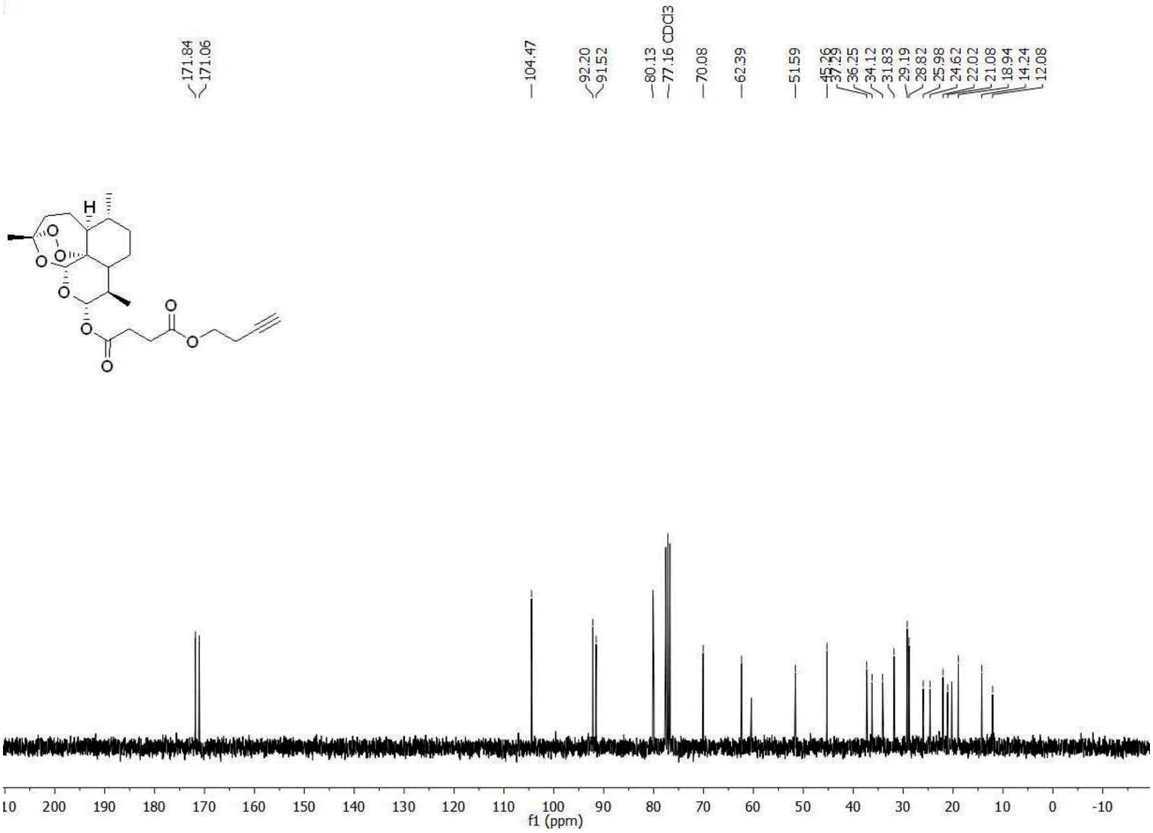

ESI mass spectrum of **24** recorded on a Bruker micrOTOF II focus TOF MS-spectrometer:

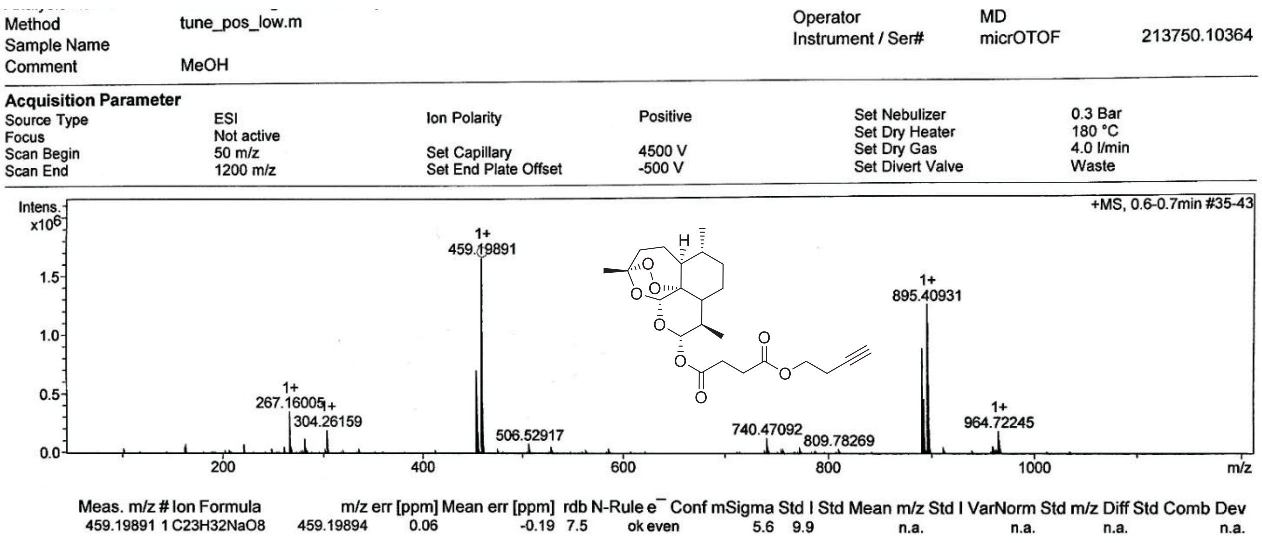


$^{1}$H NMR spectrum of **25** recorded on a Bruker Avance spectrometer (300 MHz, $CDCl_3$):

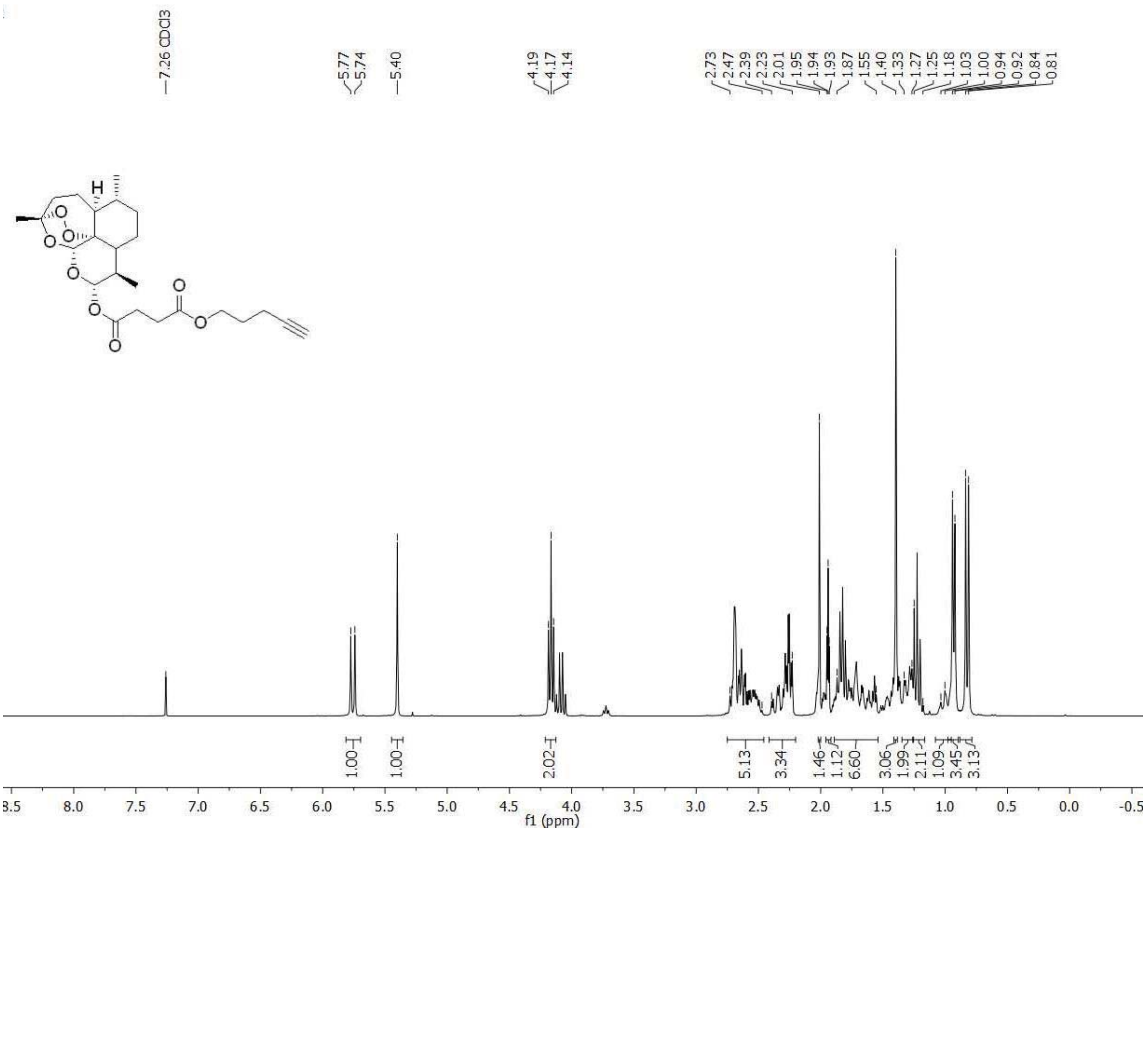

$^{13}C$ NMR spectrum of **25** recorded on a Bruker Avance spectrometer (100 MHz, $CDCl_3$):

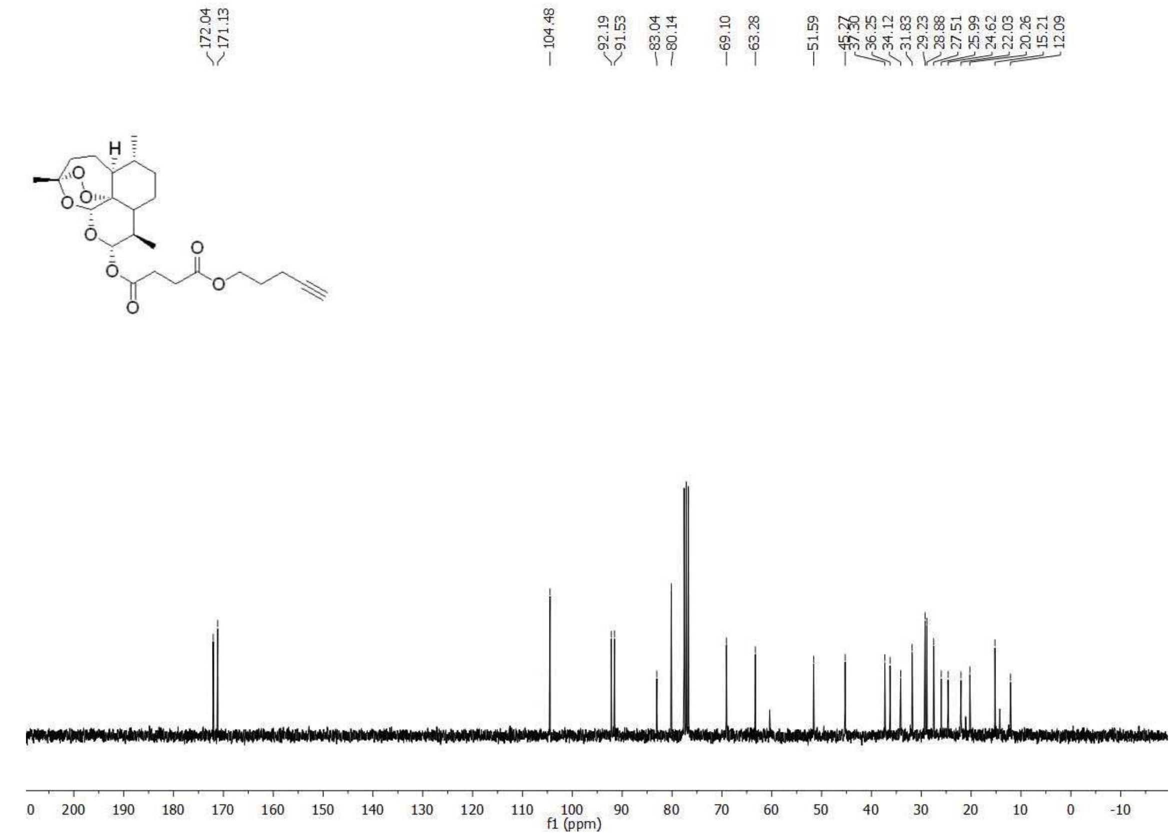


ESI mass spectrum of **25** recorded on a Bruker micrOTOF II focus TOF MS-spectrometer:

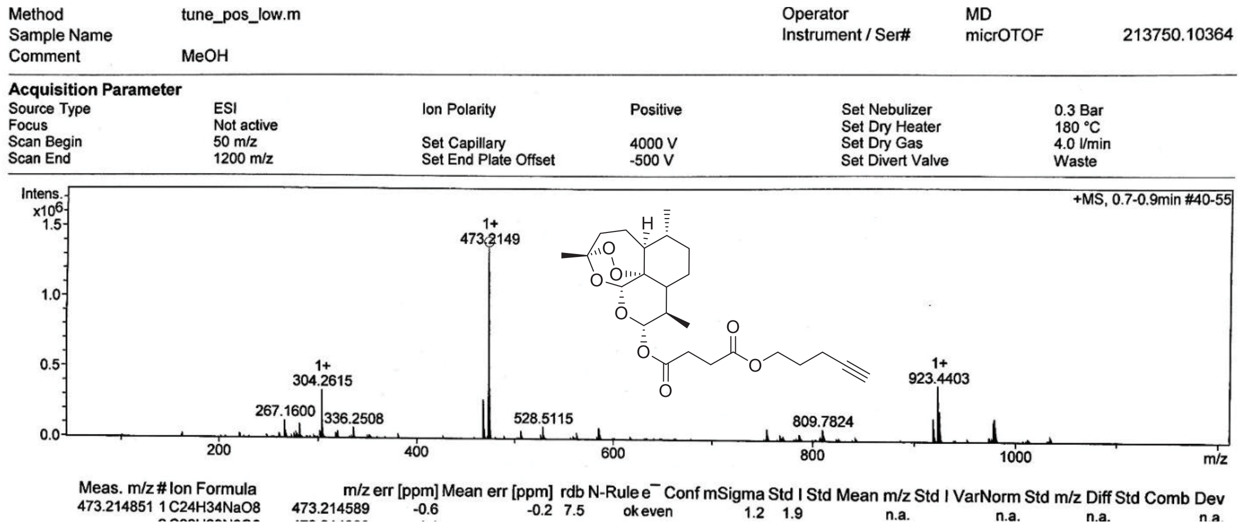

$^{1}$H NMR spectrum of **26** recorded on a Bruker Avance spectrometer (300 MHz, $CDCl_3$):

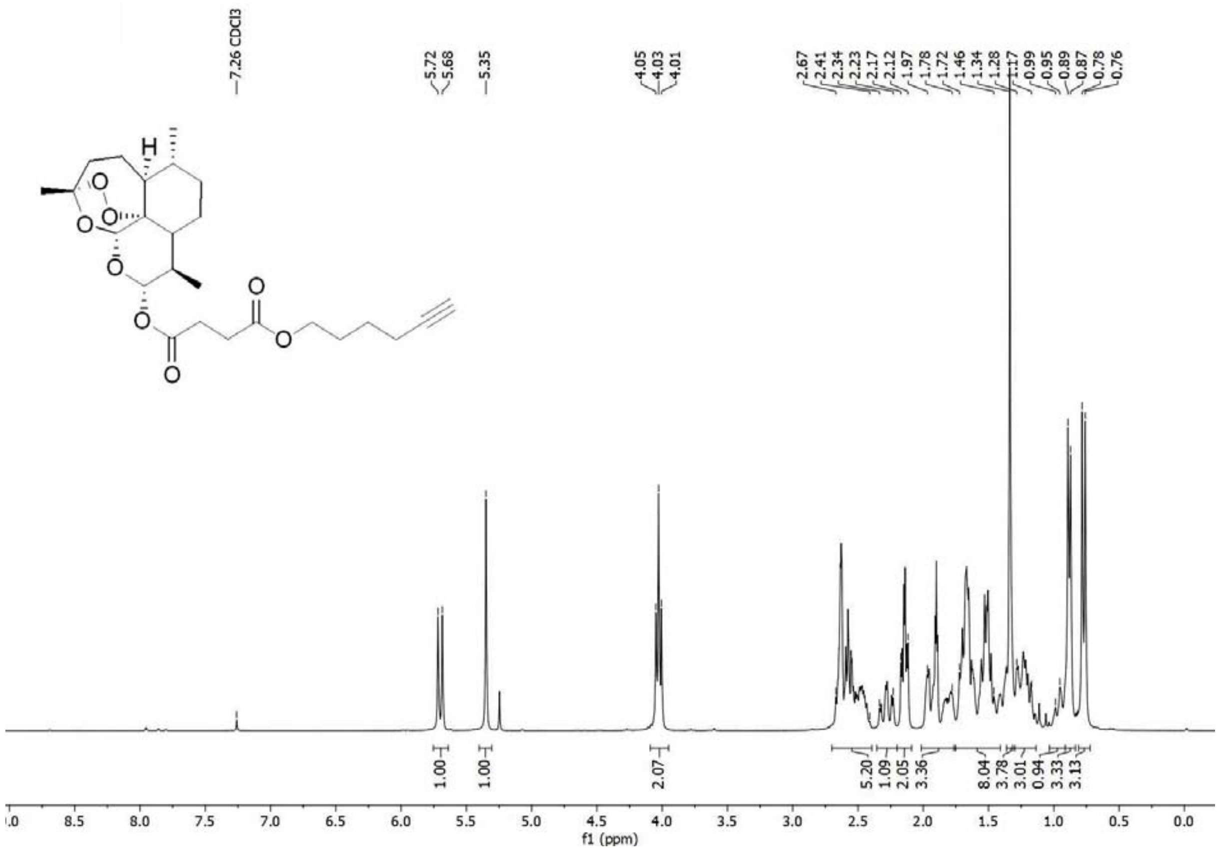


$^{13}$C NMR spectrum of **26** recorded on a Bruker Avance spectrometer (100 MHz, $CDCl_3$):

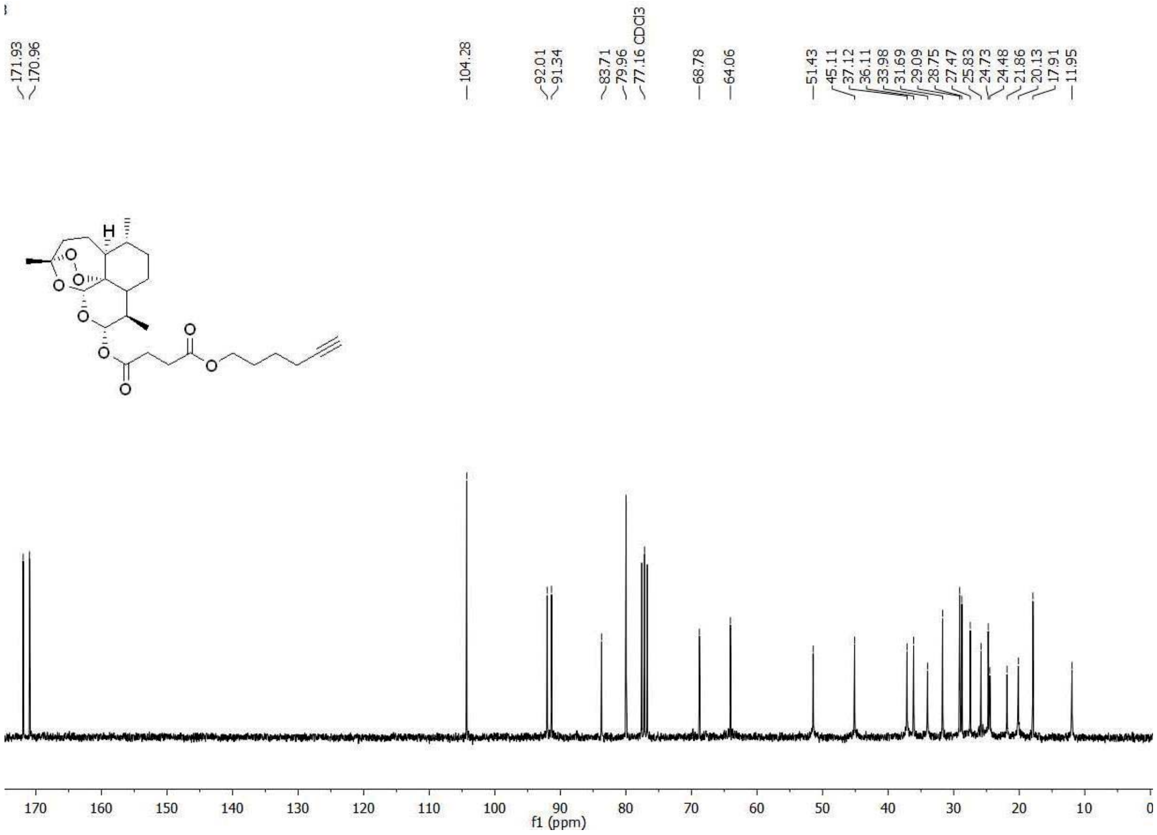

ESI mass spectrum of **26** recorded on a Bruker micrOTOF II focus TOF MS-spectrometer:

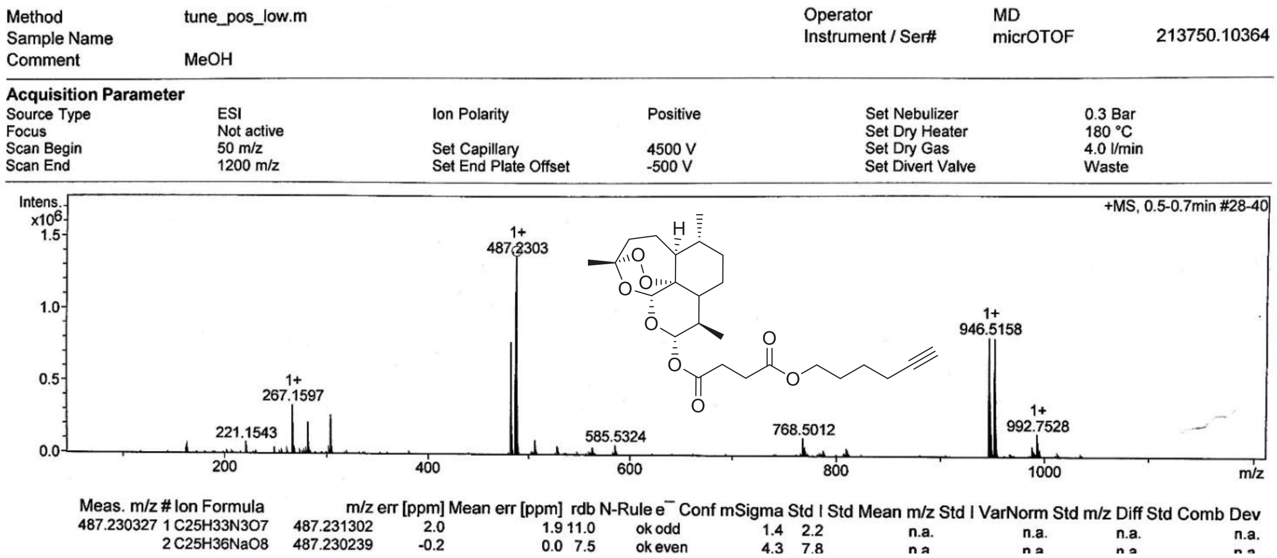


$^{1}$H NMR spectrum of hybrid **4** recorded on a Bruker Avance spectrometer (300 MHz, $CDCl_3$):

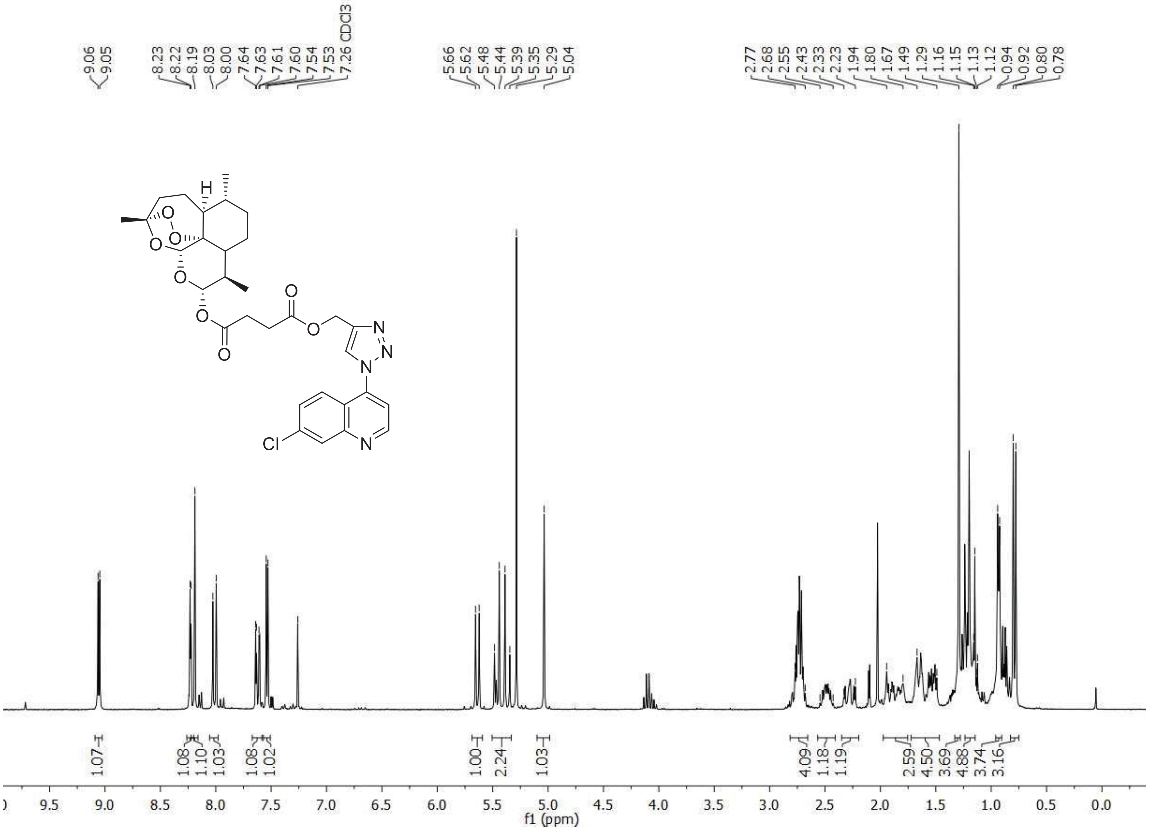

$^{13}C$ NMR spectrum of hybrid **4** recorded on a Bruker Avance spectrometer (100 MHz, $CDCl_3$):

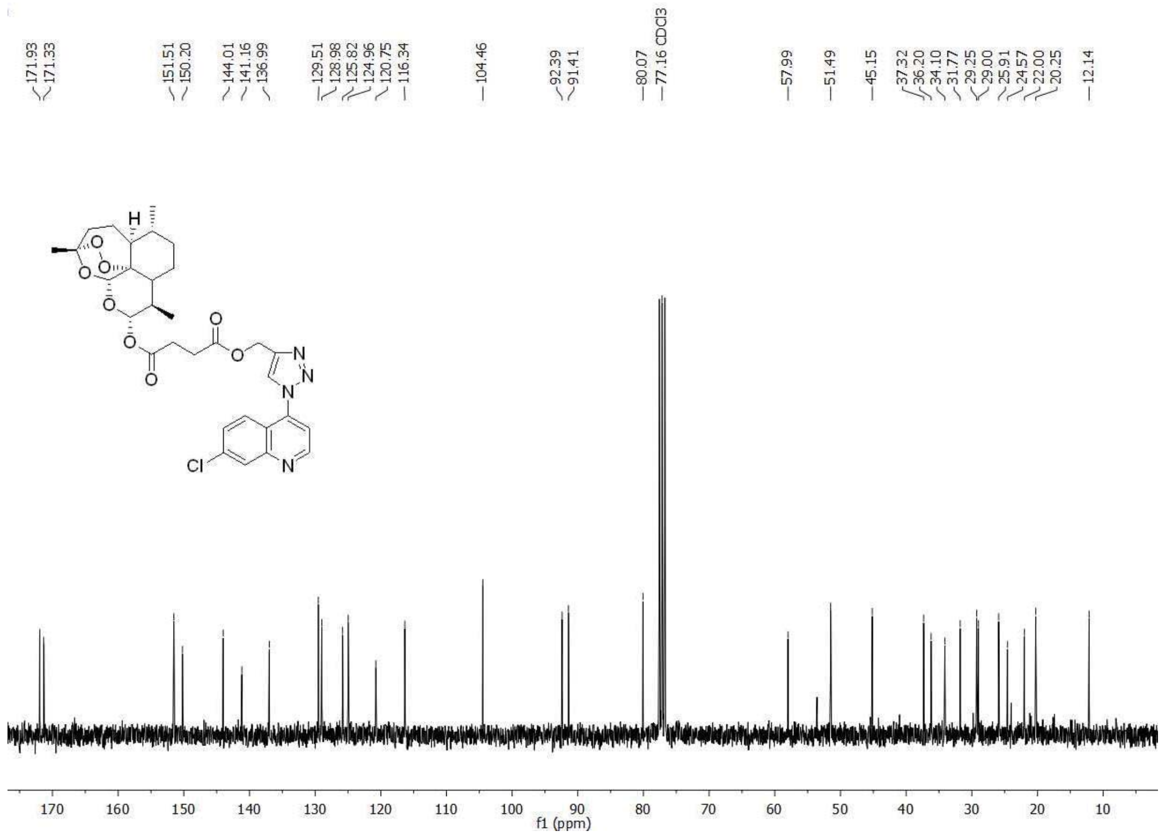


ESI mass spectrum of hybrid **4** recorded on a Bruker micrOTOF II focus TOF MS spectrometer:

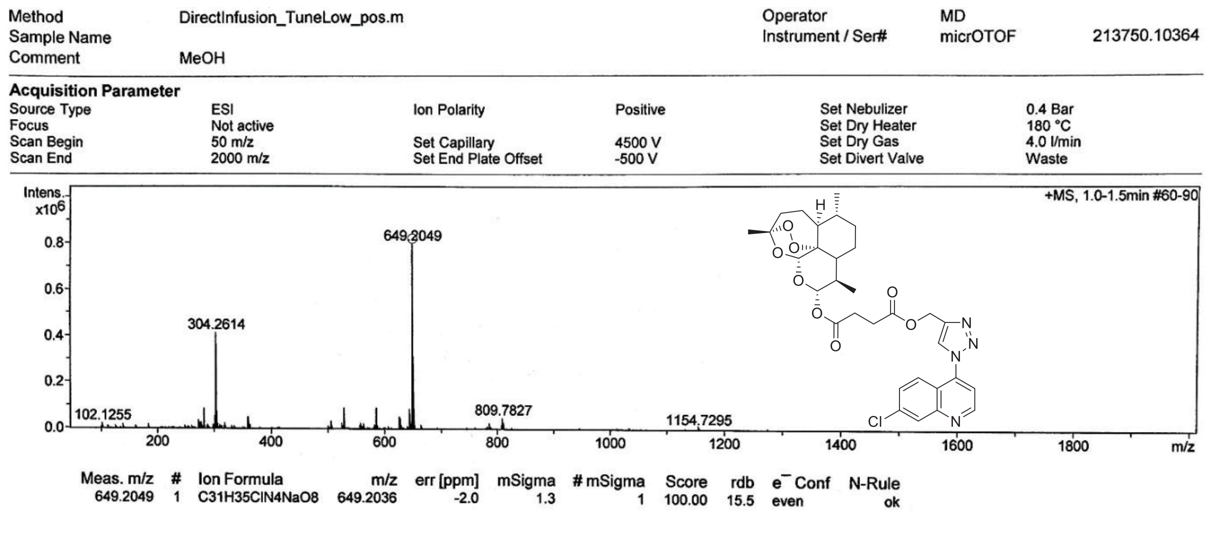

$^{1}$H NMR spectrum of hybrid **5** recorded on a Bruker Avance spectrometer (300 MHz, $CDCl_3$):

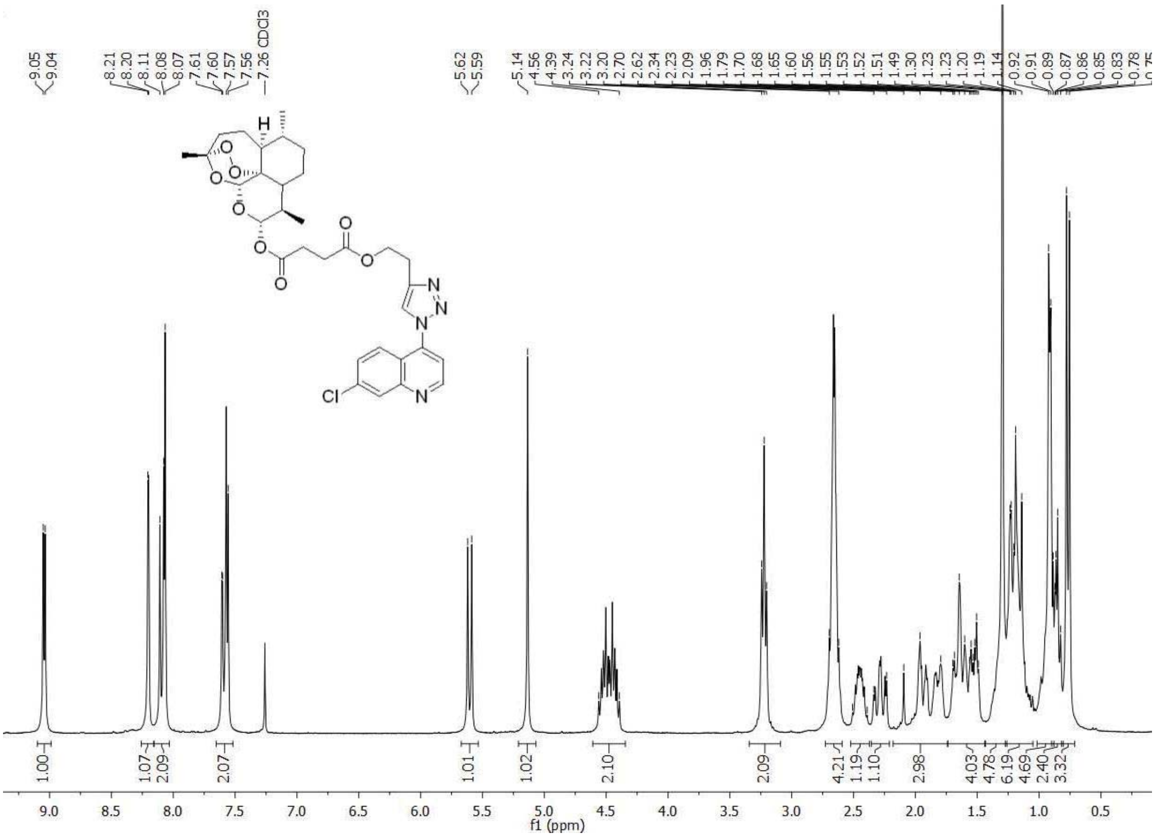

$^{13}$C NMR spectrum of hybrid **5** recorded on a Bruker Avance spectrometer (100 MHz, $CDCl_3$):

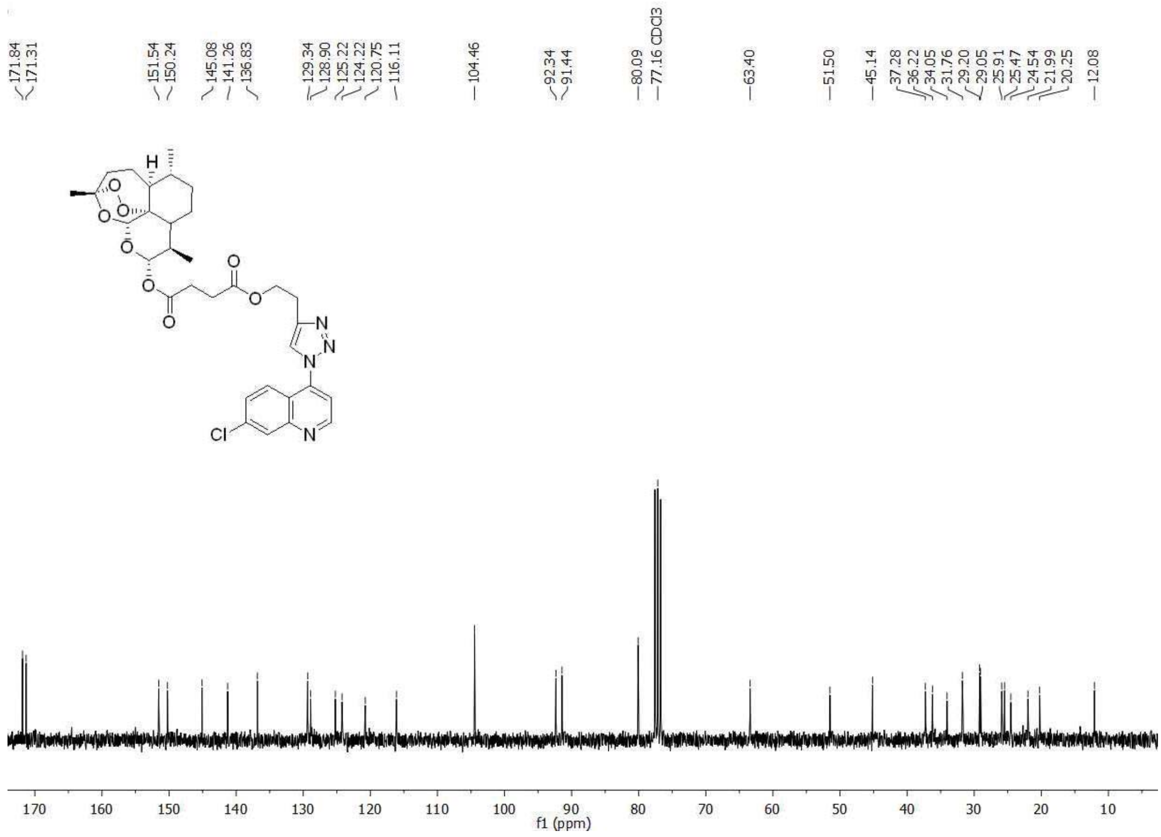

ESI mass spectrum of hybrid **5** recorded on a Bruker micrOTOF II focus TOF MS-spectrometer:

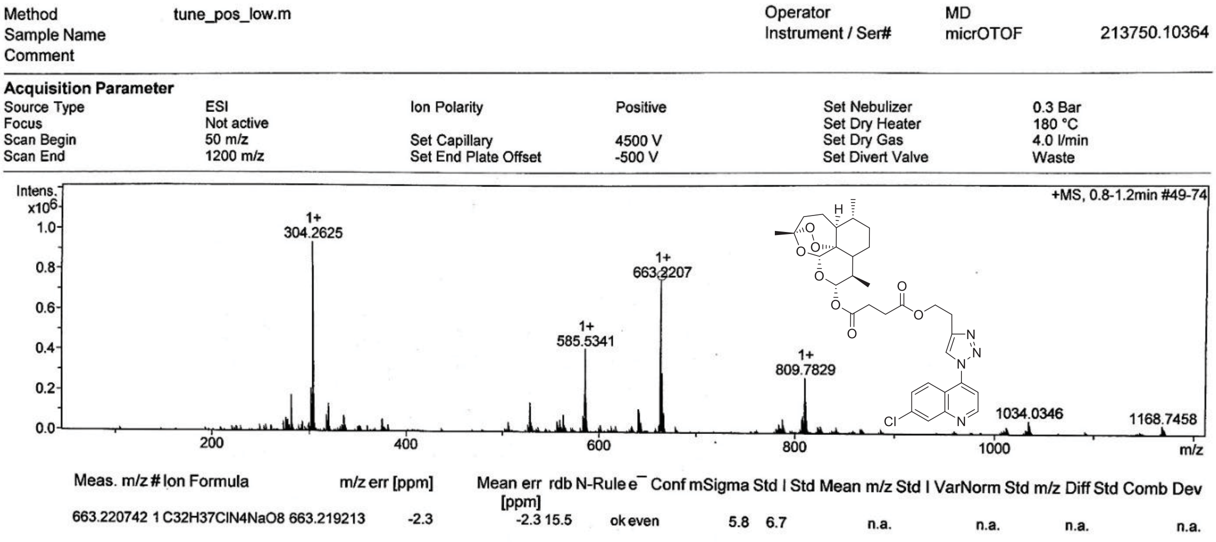


$^{1}$H NMR spectrum of hybrid **6** recorded on a Bruker Avance spectrometer (300 MHz, $CDCl_3$):

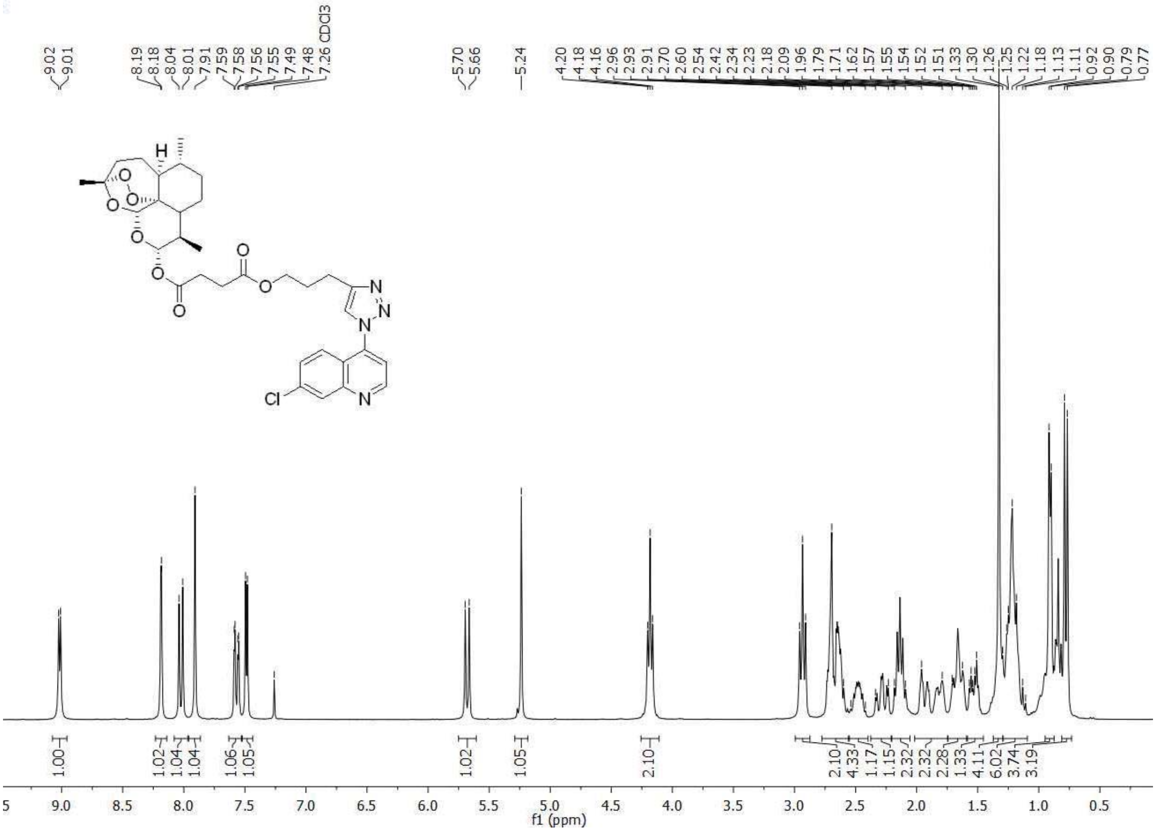

$^{13}C$ NMR spectrum of hybrid **6** recorded on a Bruker Avance spectrometer (100 MHz, $CDCl_3$):

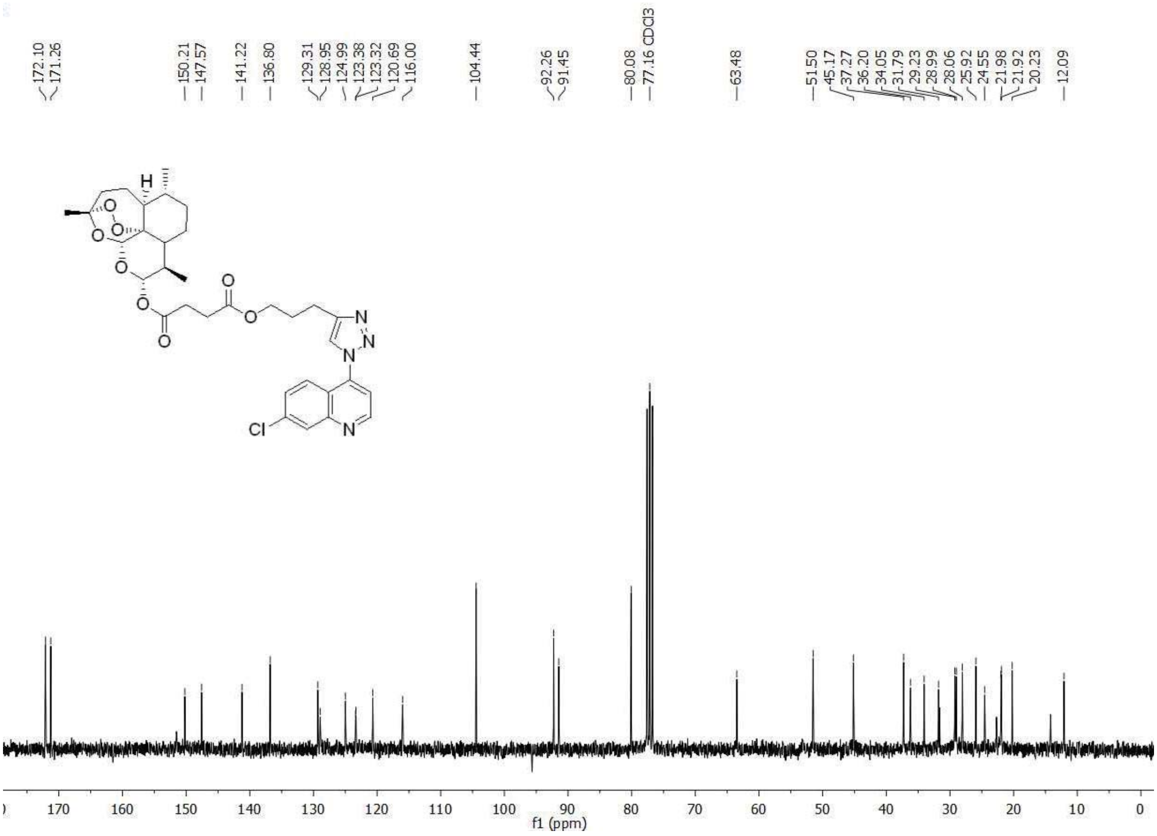


ESI mass spectrum of hybrid **6** recorded on a Bruker micrOTOF II focus TOF MS-spectrometer:

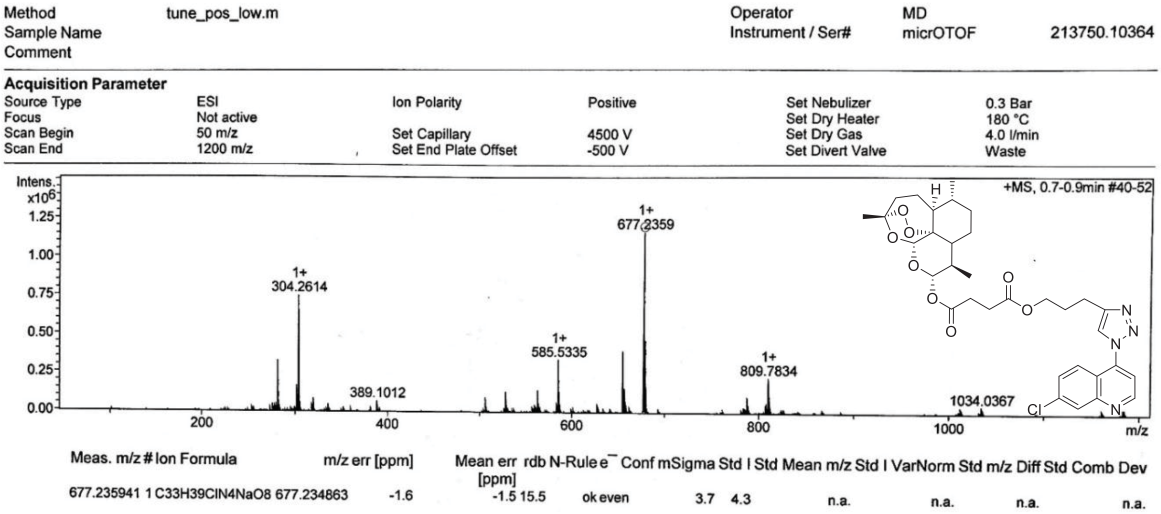

$^{1}$H NMR spectrum of hybrid **7** recorded on a Bruker Avance spectrometer (300 MHz, $CDCl_3$):

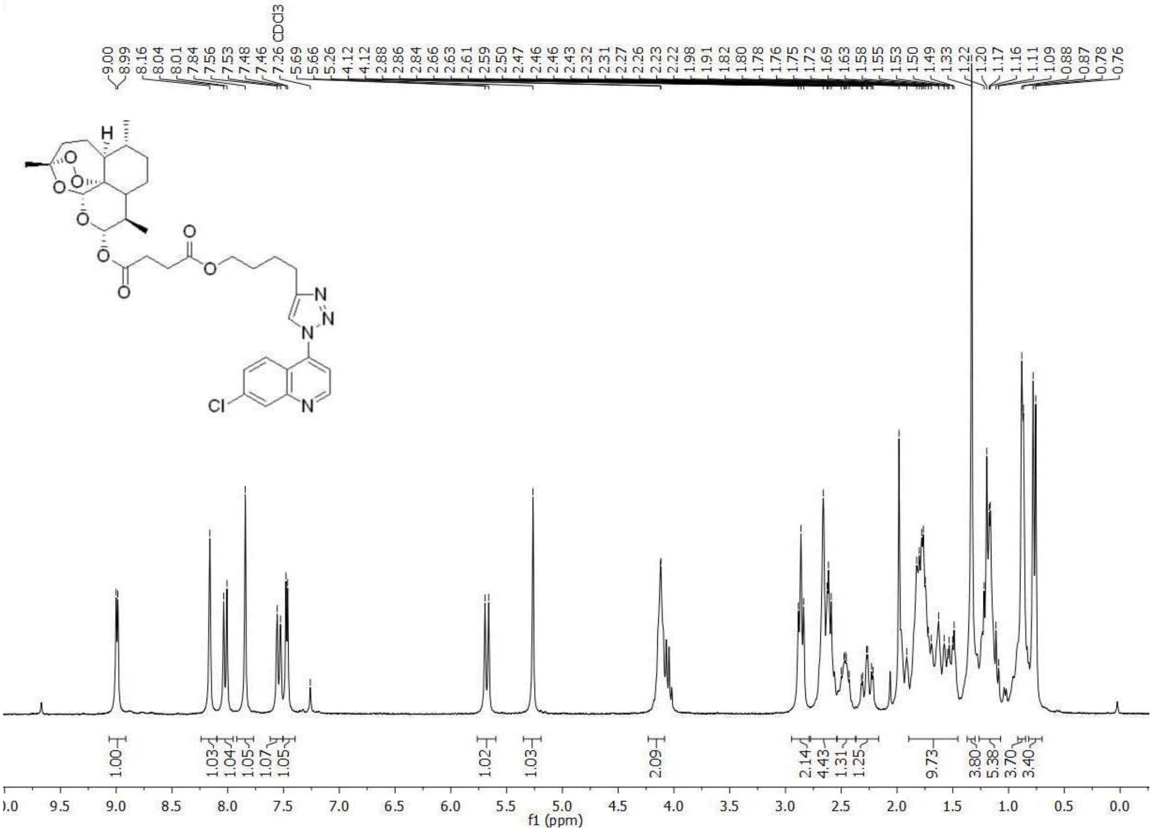


$^{13}$C NMR spectrum of hybrid **7** recorded on a Bruker Avance spectrometer (100 MHz, $CDCl_3$):

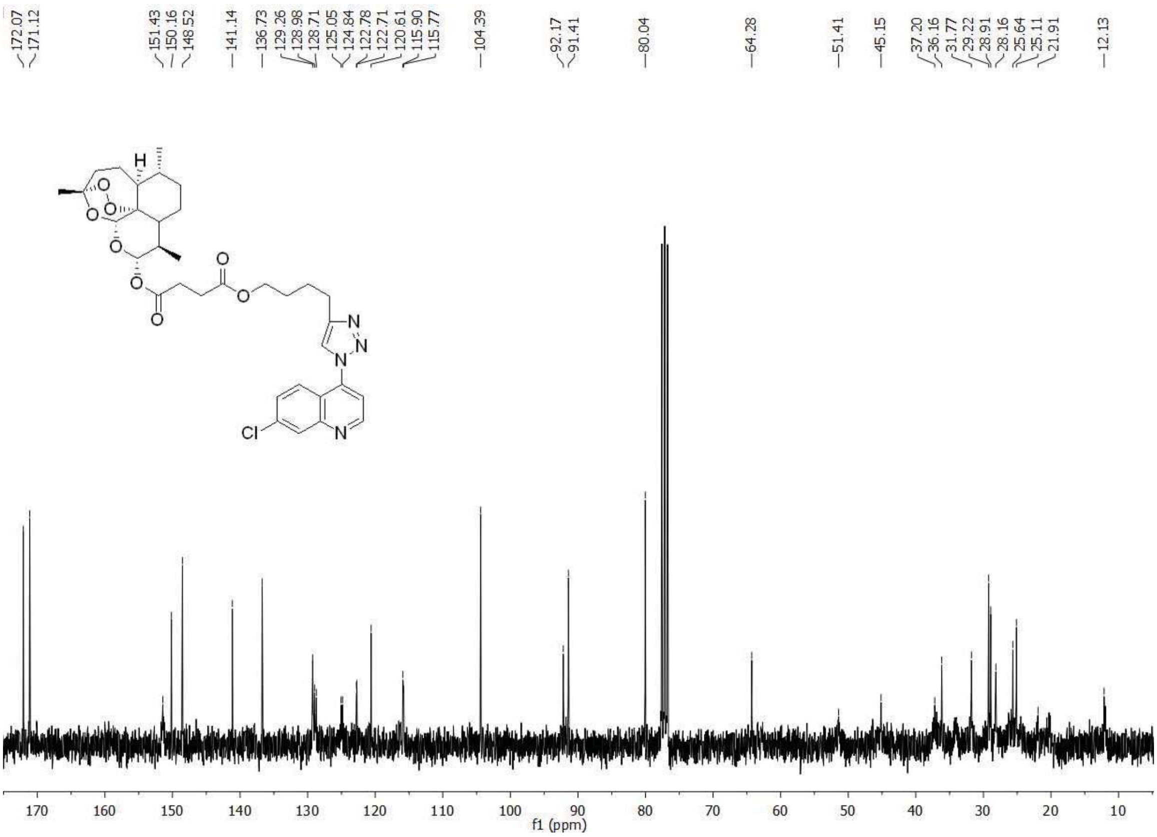

ESI mass spectrum of hybrid **7** recorded on a Bruker micrOTOF II focus TOF MS-spectrometer:

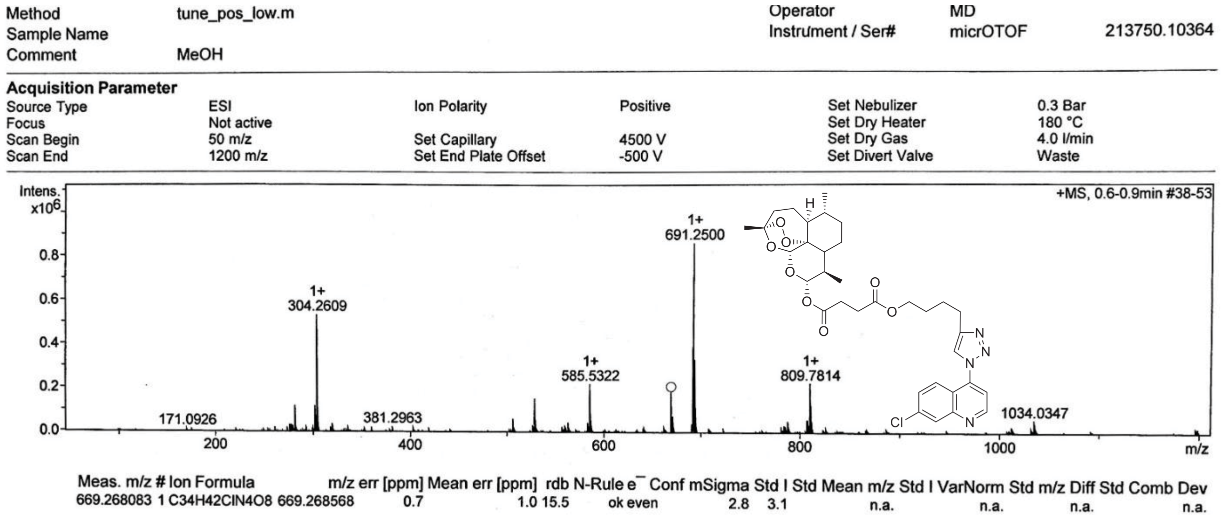


$^{1}$H NMR spectrum of **28** recorded on a Bruker Avance spectrometer (300 MHz, $CDCl_3$):

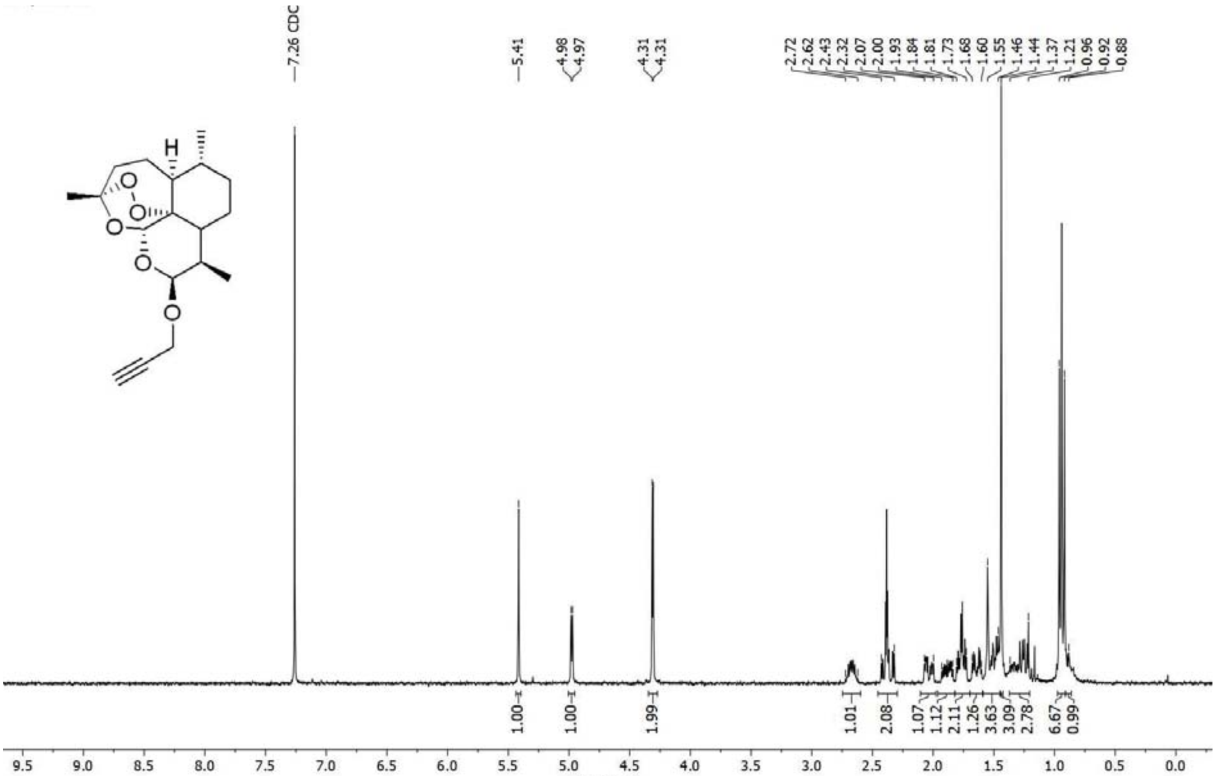

$^{13}C$ NMR spectrum of **28** recorded on a Bruker Avance spectrometer (75 MHz, $CDCl_3$):

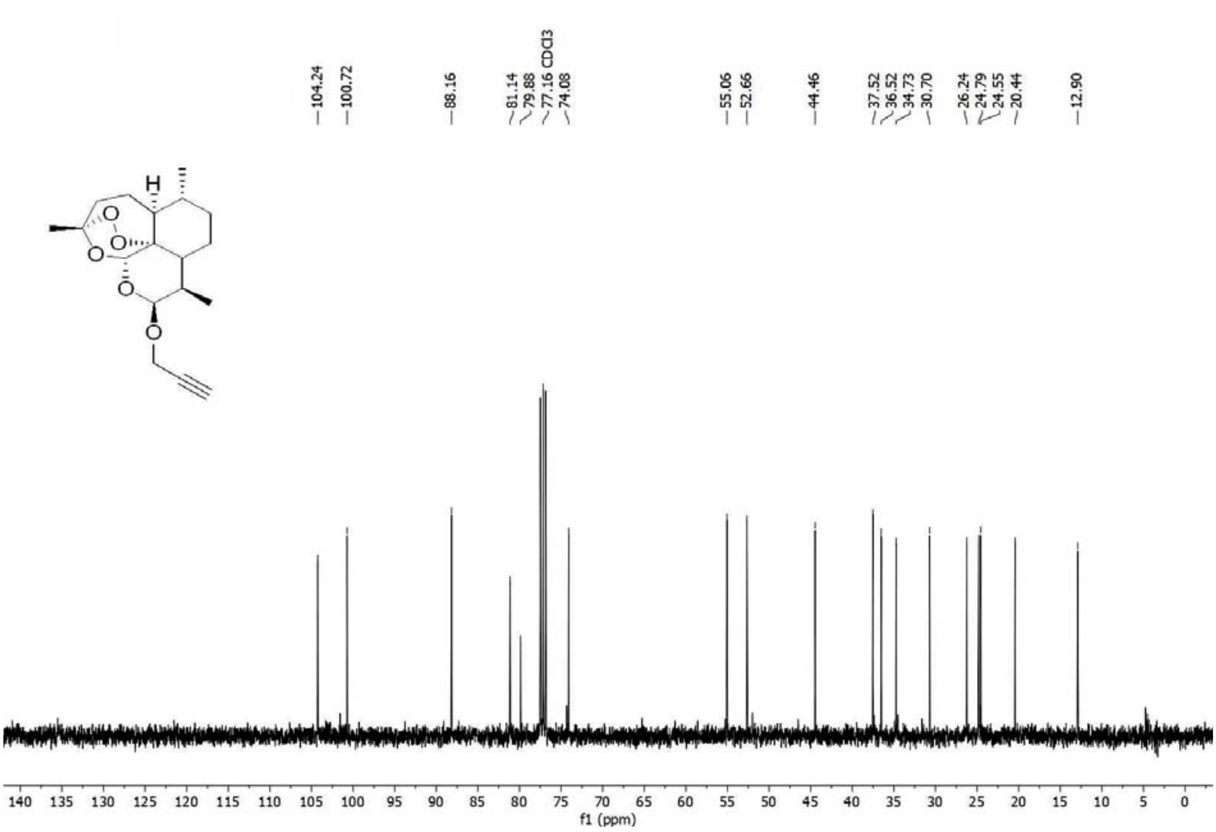

$^{1}H$ NMR spectrum of **29** recorded on a Bruker Avance spectrometer (300 MHz, $CDCl_3$):

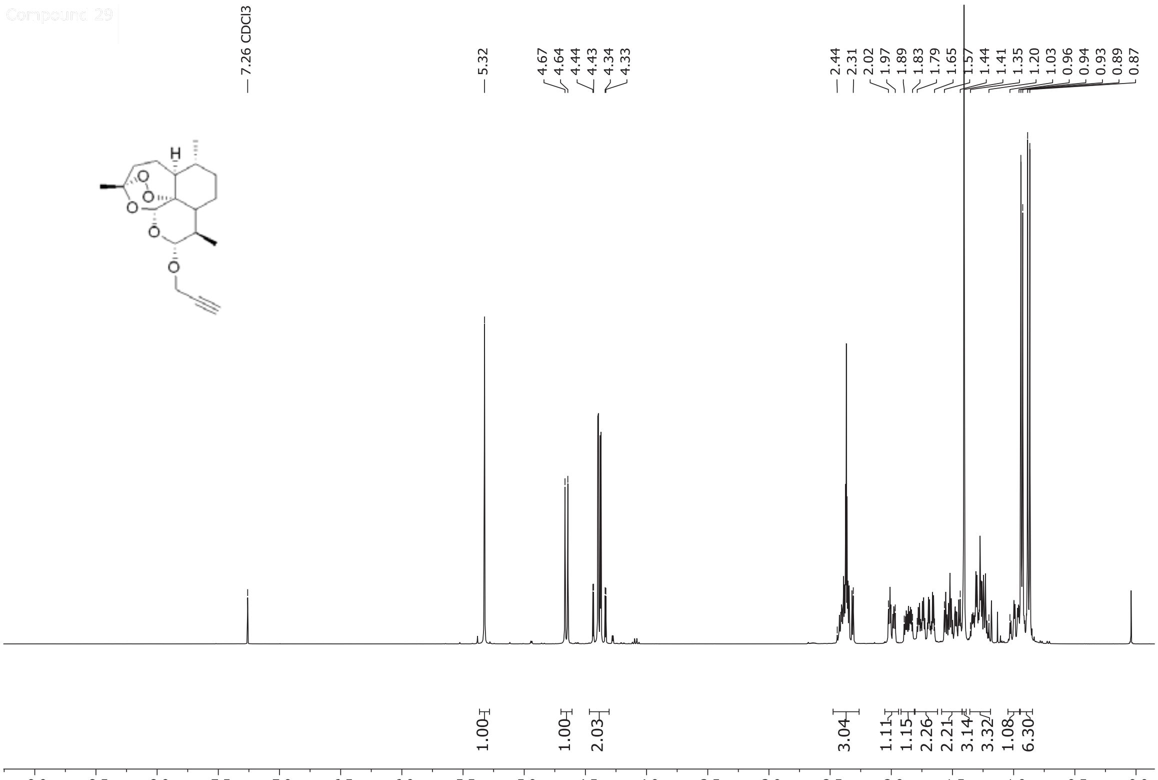

$^{13}C$ NMR spectrum of **29** recorded on a Bruker Avance spectrometer (75 MHz, $CDCl_3$):

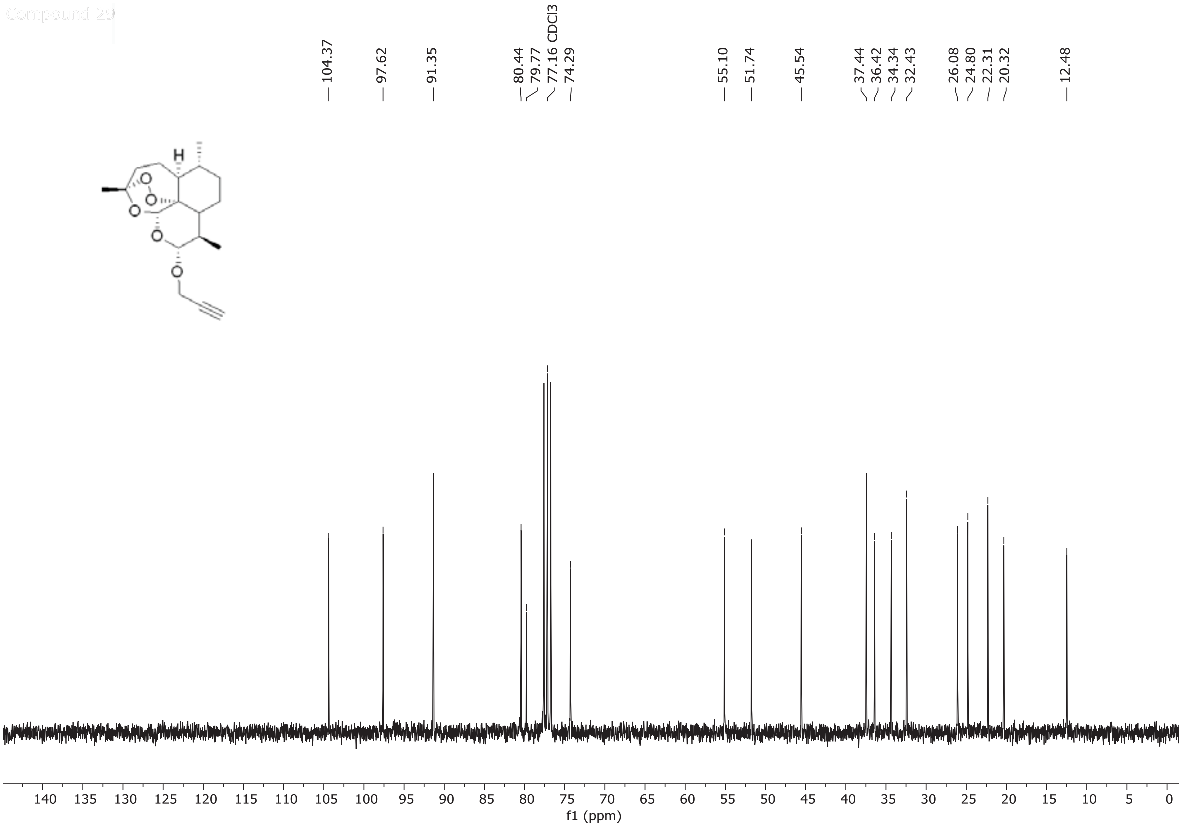


ESI mass spectrum of Compound **29** recorded on a Bruker micrOTOF II focus TOF MS-spectrometer:

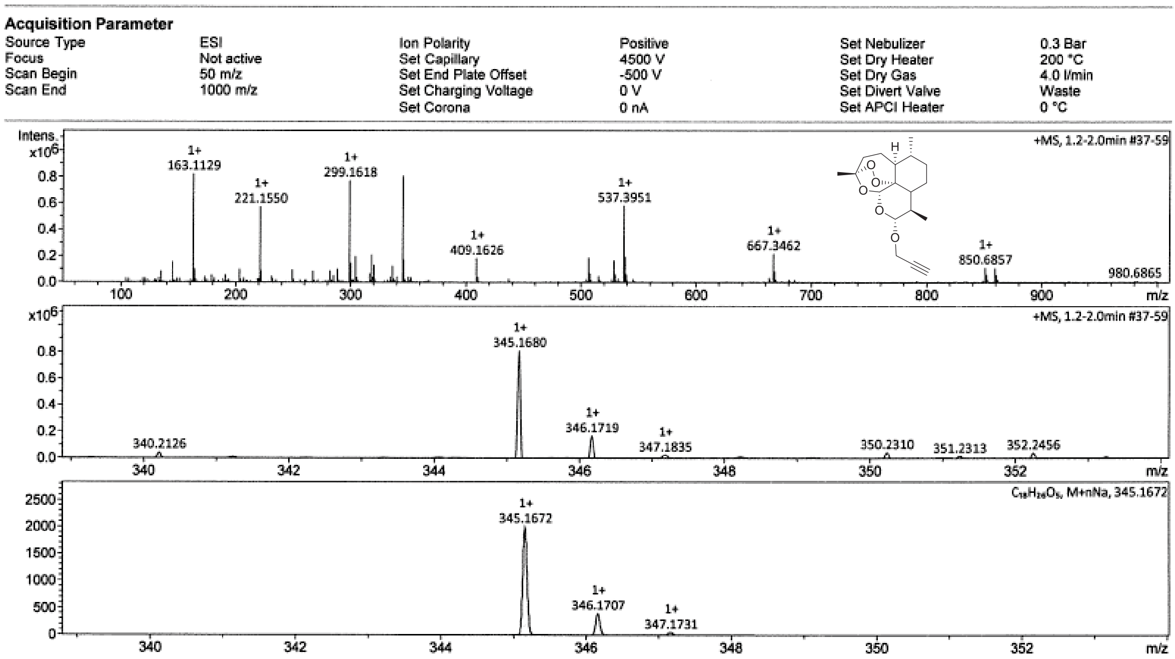

$^{1}$H NMR spectrum of **30** recorded on a Bruker Avance spectrometer (300 MHz, $CDCl_3$):

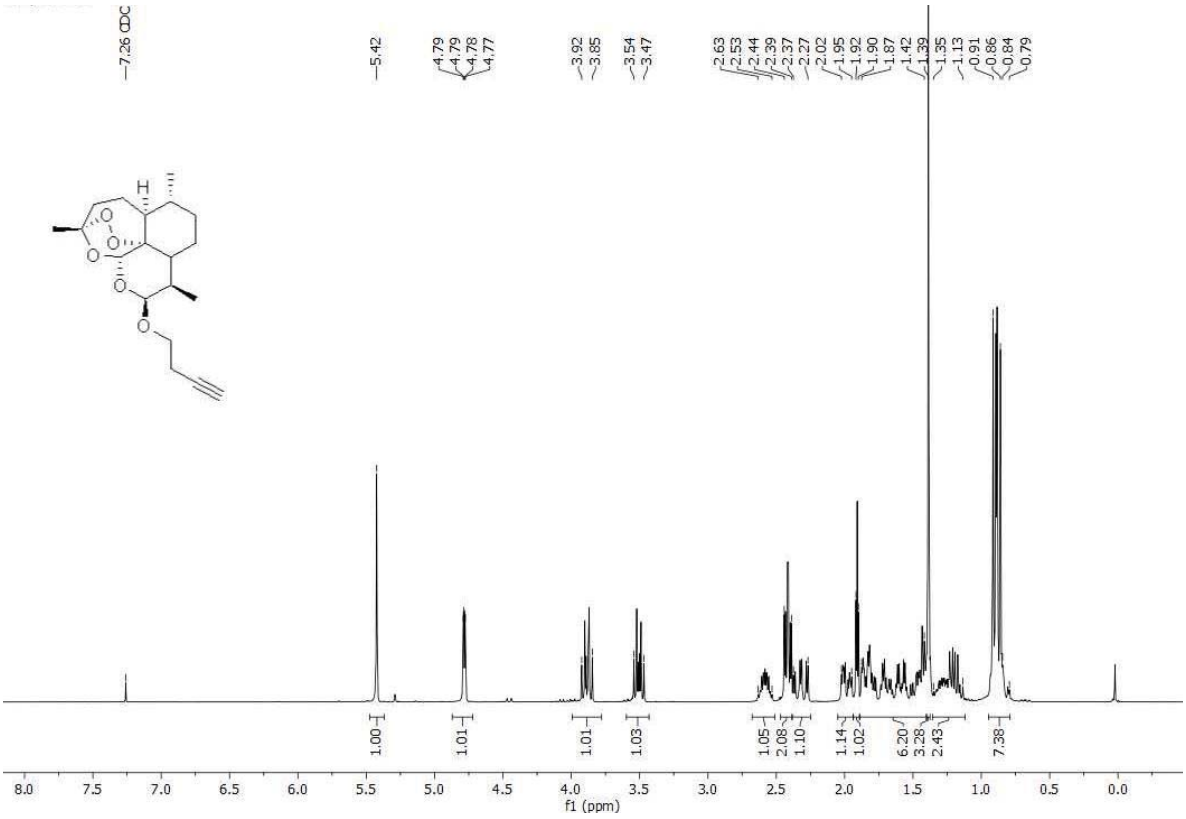


$^{13}$C NMR spectrum of **30** recorded on a Bruker Avance spectrometer (75 MHz, $CDCl_3$):

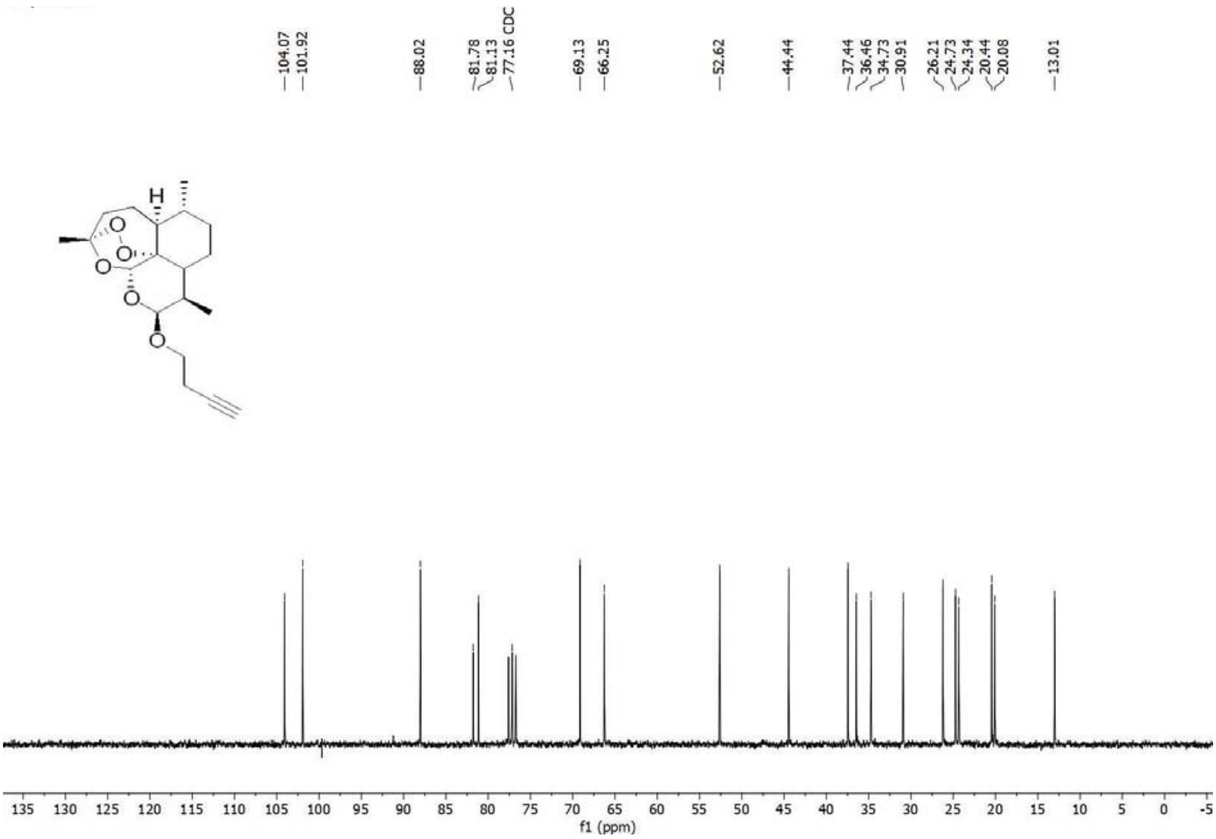

ESI mass spectrum of Compound **30** recorded on a Bruker micrOTOF II focus TOF MS-spectrometer:

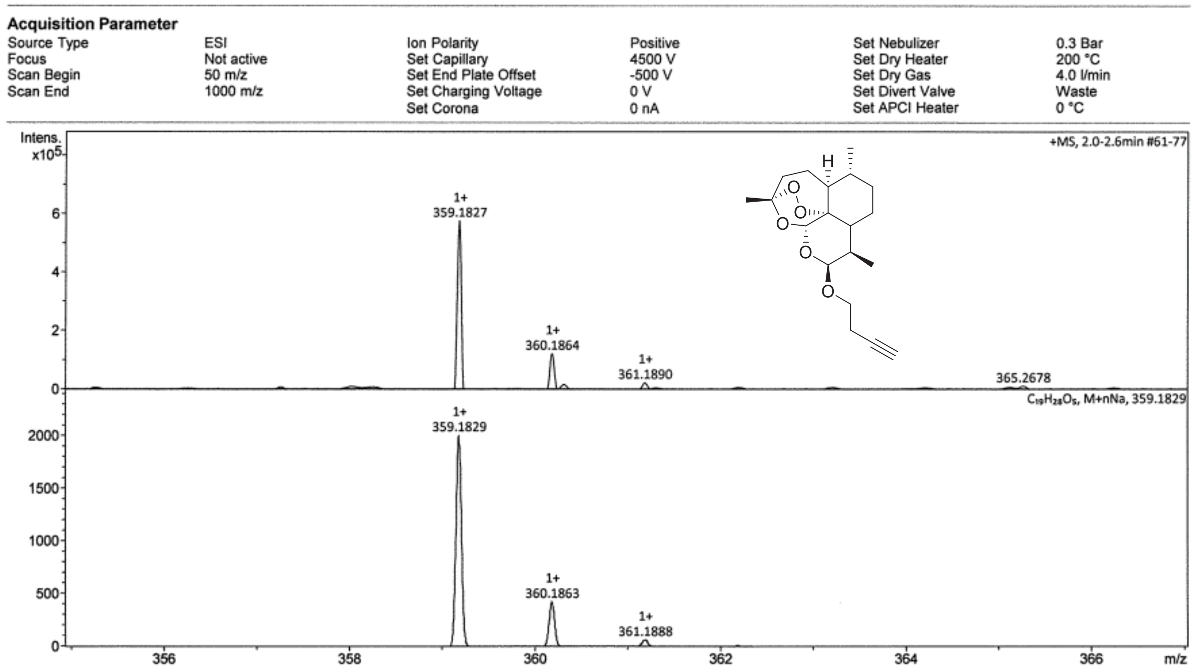


$^{1}H$ NMR spectrum of **31** recorded on a Bruker Avance spectrometer (300 MHz, $CDCl_3$):

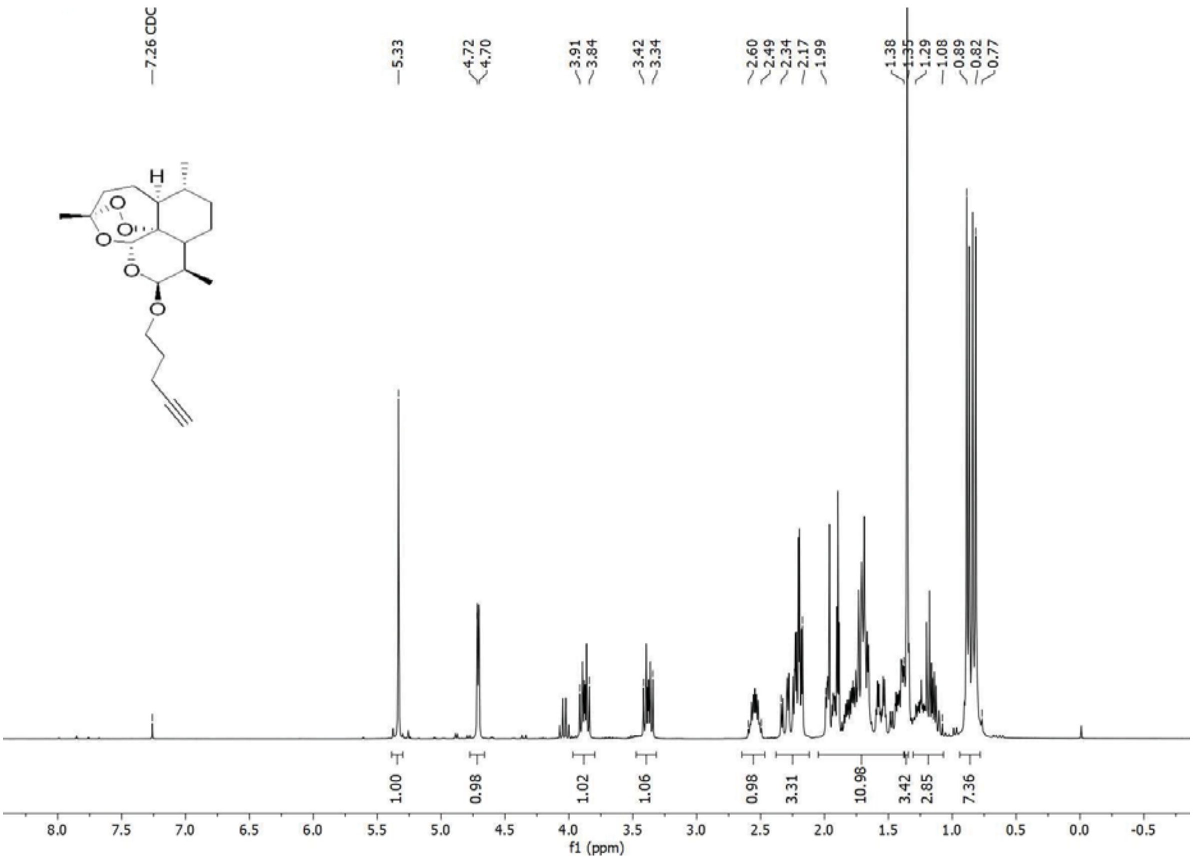

$^{13}C$ NMR spectrum of **31** recorded on a Bruker Avance spectrometer (75 MHz, $CDCl_3$):

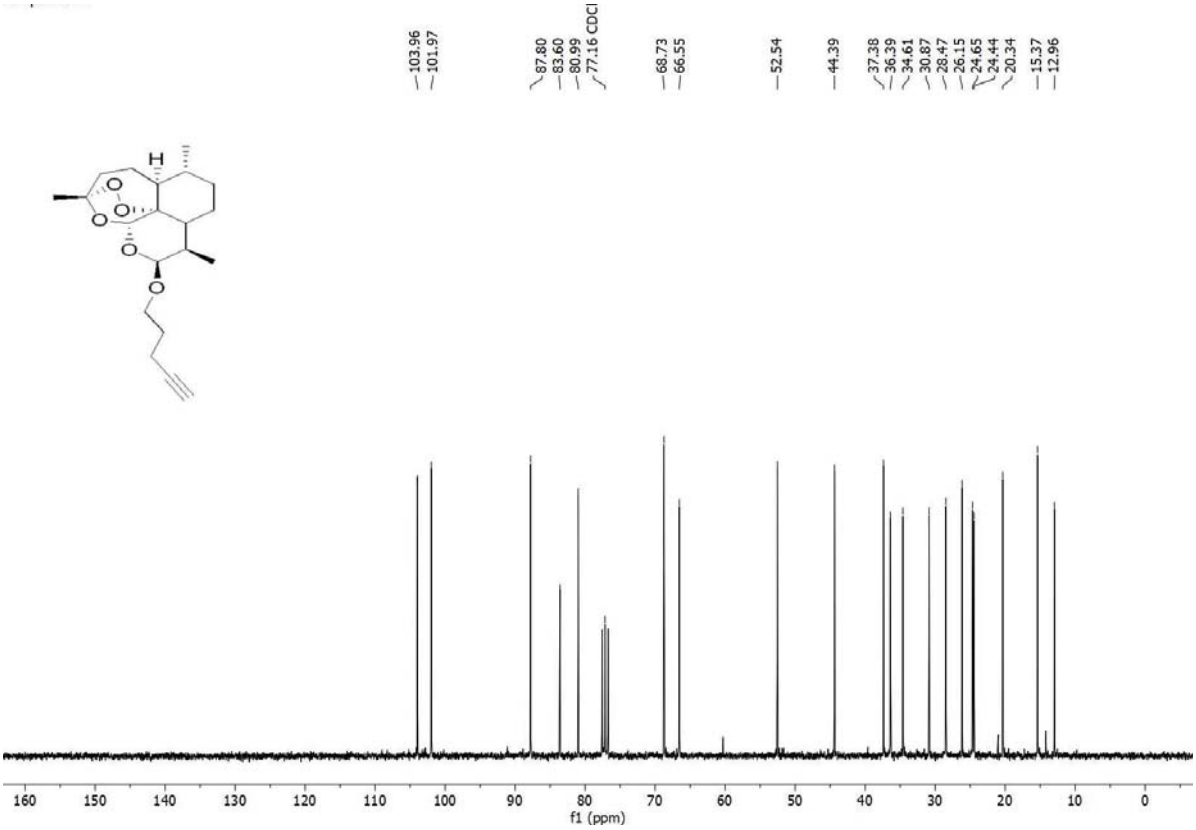


ESI mass spectrum of Compound **31** recorded on a Bruker micrOTOF II focus TOF MS-spectrometer:

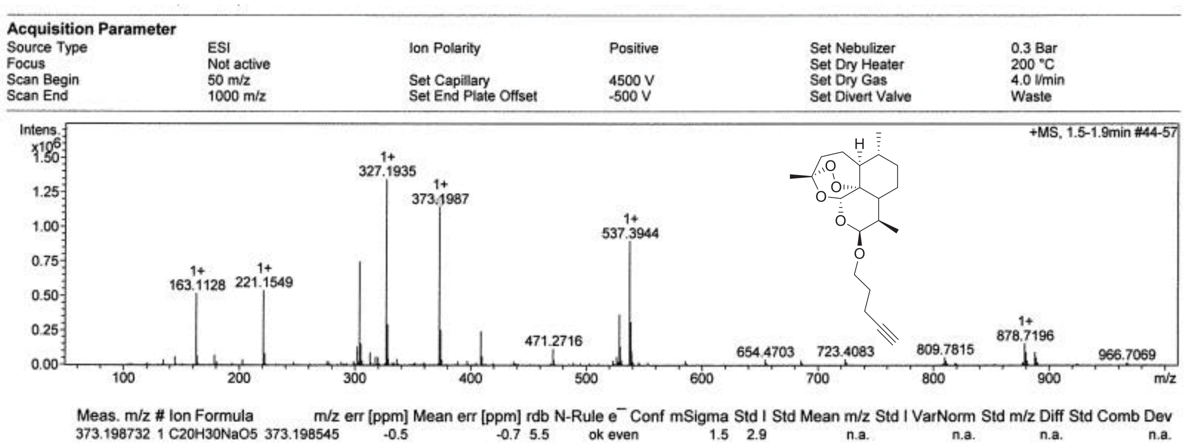

$^{1}$H NMR spectrum of **32** recorded on a Bruker Avance spectrometer (300 MHz, $CDCl_3$):

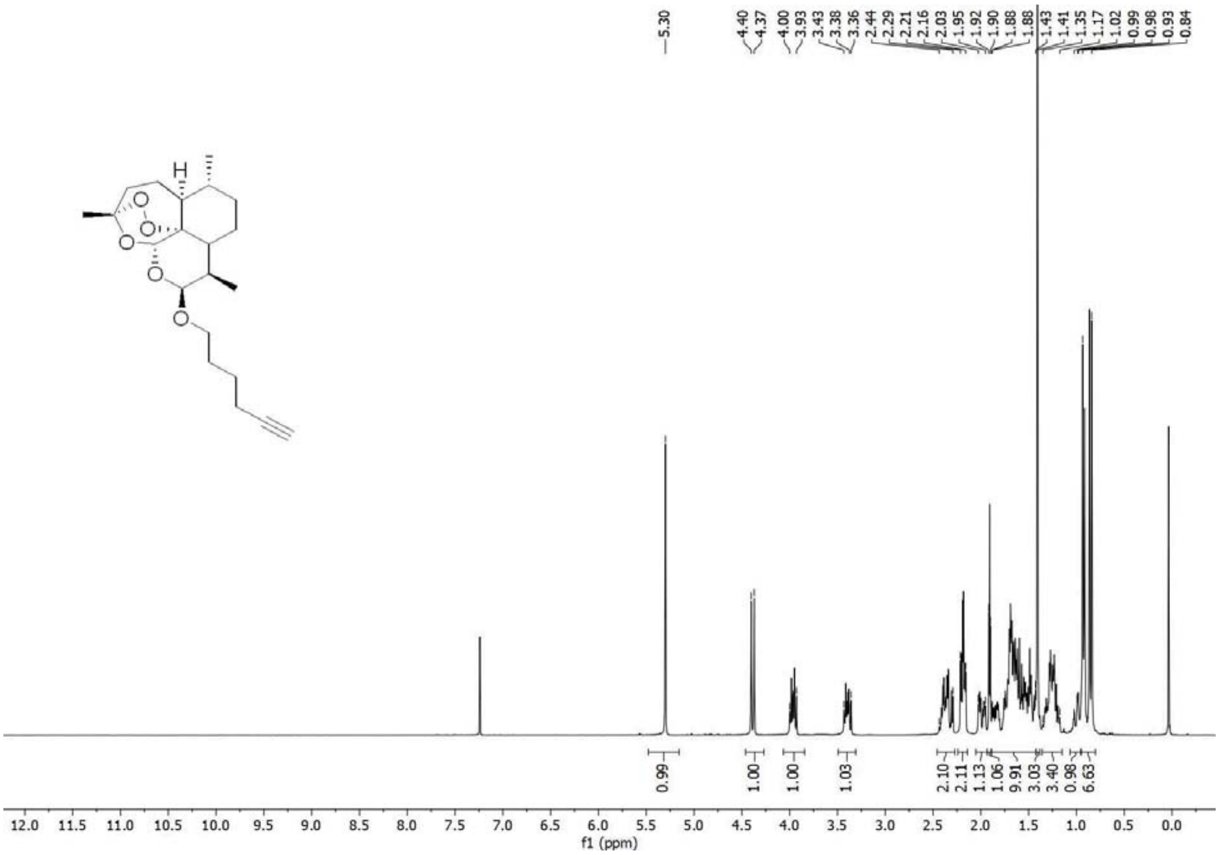


ESI mass spectrum of Compound **32** recorded on a Bruker micrOTOF II focus TOF MS-spectrometer:

**Acquisition Parameter**

| | | | | | |
|---|---|---|---|---|---|
| Source Type | ESI | Ion Polarity | Positive | Set Nebulizer | 0.3 Bar |
| Focus | Not active | | | Set Dry Heater | 180 °C |
| Scan Begin | 50 m/z | Set Capillary | 3800 V | Set Dry Gas | 4.0 l/min |
| Scan End | 700 m/z | Set End Plate Offset | -500 V | Set Divert Valve | Waste |

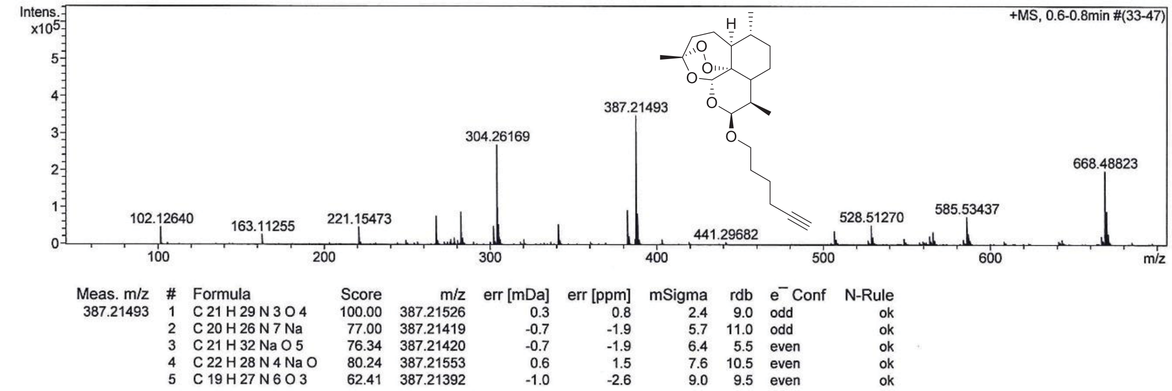


| Meas. m/z | # | Formula | Score | m/z | err [mDa] | err [ppm] | mSigma | rdb | e⁻ Conf | N-Rule |
|---|---|---|---|---|---|---|---|---|---|---|
| 387.21493 | 1 | C 21 H 29 N 3 O 4 | 100.00 | 387.21526 | 0.3 | 0.8 | 2.4 | 9.0 | odd | ok |
| | 2 | C 20 H 26 N 7 Na | 77.00 | 387.21419 | -0.7 | -1.9 | 5.7 | 11.0 | odd | ok |
| | 3 | C 21 H 32 Na O 5 | 76.34 | 387.21420 | -0.7 | -1.9 | 6.4 | 5.5 | even | ok |
| | 4 | C 22 H 28 N 4 Na O | 80.24 | 387.21553 | 0.6 | 1.5 | 7.6 | 10.5 | even | ok |
| | 5 | C 19 H 27 N 6 O 3 | 62.41 | 387.21392 | -1.0 | -2.6 | 9.0 | 9.5 | even | ok |

$^{1}H$ NMR spectrum of hybrid **8** recorded on a Bruker Avance spectrometer (300 MHz, $CDCl_3$):

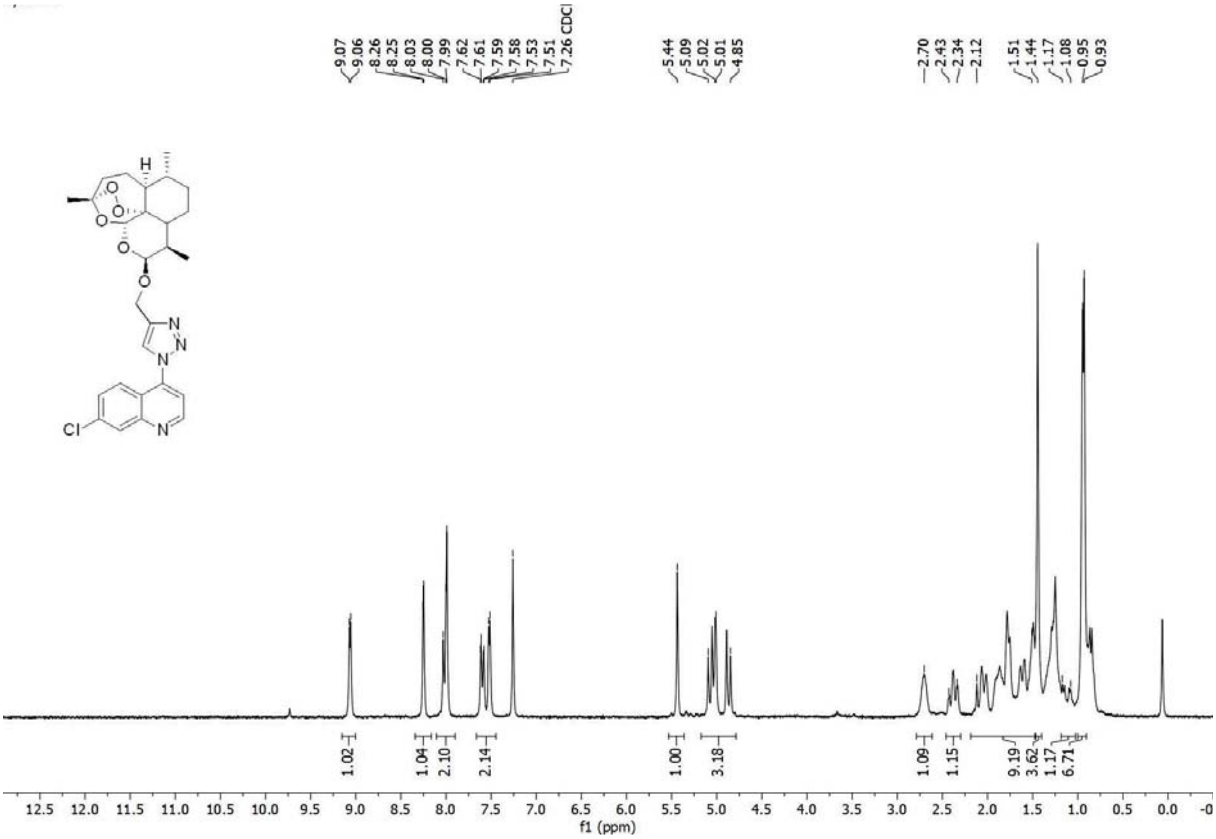


$^{13}C$ NMR spectrum of hybrid **8** recorded on a Bruker Avance spectrometer (75 MHz, $CDCl_3$):

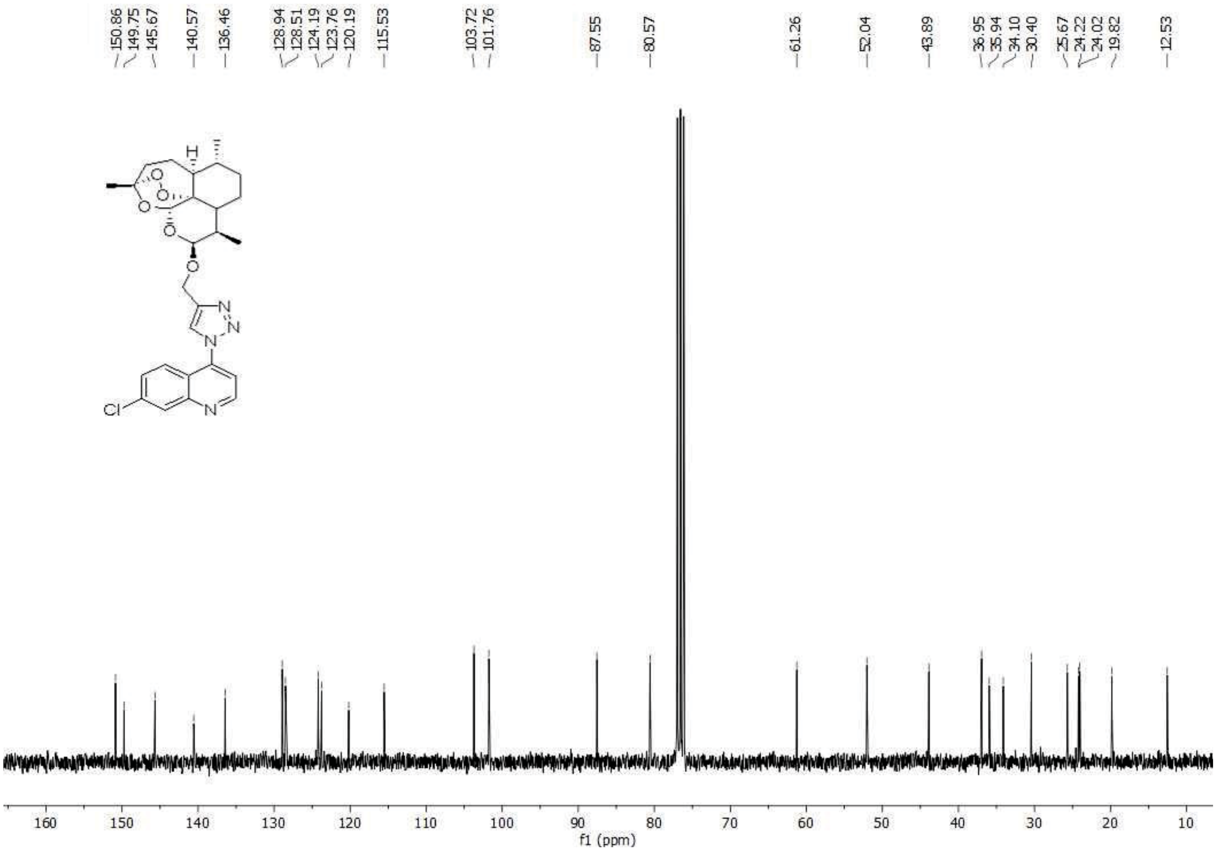

ESI mass spectrum of hybrid **8** recorded on a Bruker micrOTOF II focus TOF MS-spectrometer:

**Acquisition Parameter**

| | | | | | |
|---|---|---|---|---|---|
| Source Type | ESI | Ion Polarity | Positive | Set Nebulizer | 0.3 Bar |
| Focus | Not active | | | Set Dry Heater | 180 °C |
| Scan Begin | 50 m/z | Set Capillary | 4500 V | Set Dry Gas | 4.0 l/min |
| Scan End | 700 m/z | Set End Plate Offset | -500 V | Set Divert Valve | Waste |

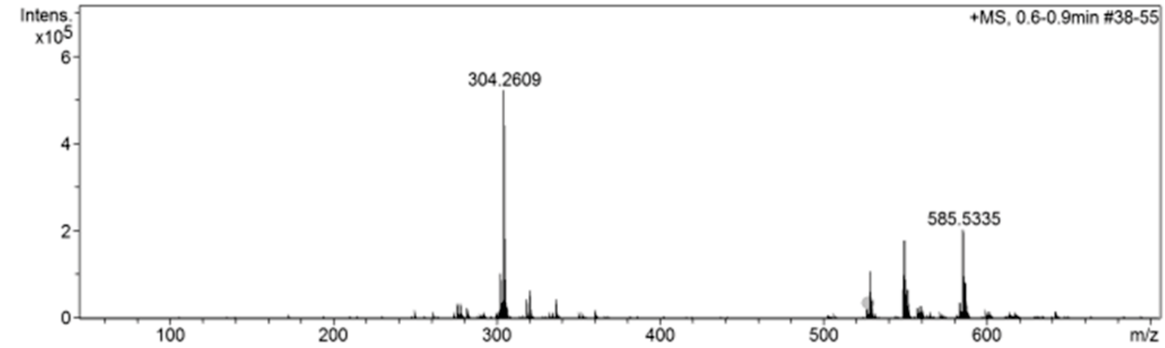


| Meas. m/z | # | Ion Formula | m/z | err [ppm] | mSigma | # mSigma | Score | rdb | e⁻ Conf | N-Rule |
|---|---|---|---|---|---|---|---|---|---|---|
| 527.2061 | 1 | C27H32ClN4O5 | 527.2056 | -1.0 | 23.0 | 1 | 100.00 | 13.5 | even | ok |

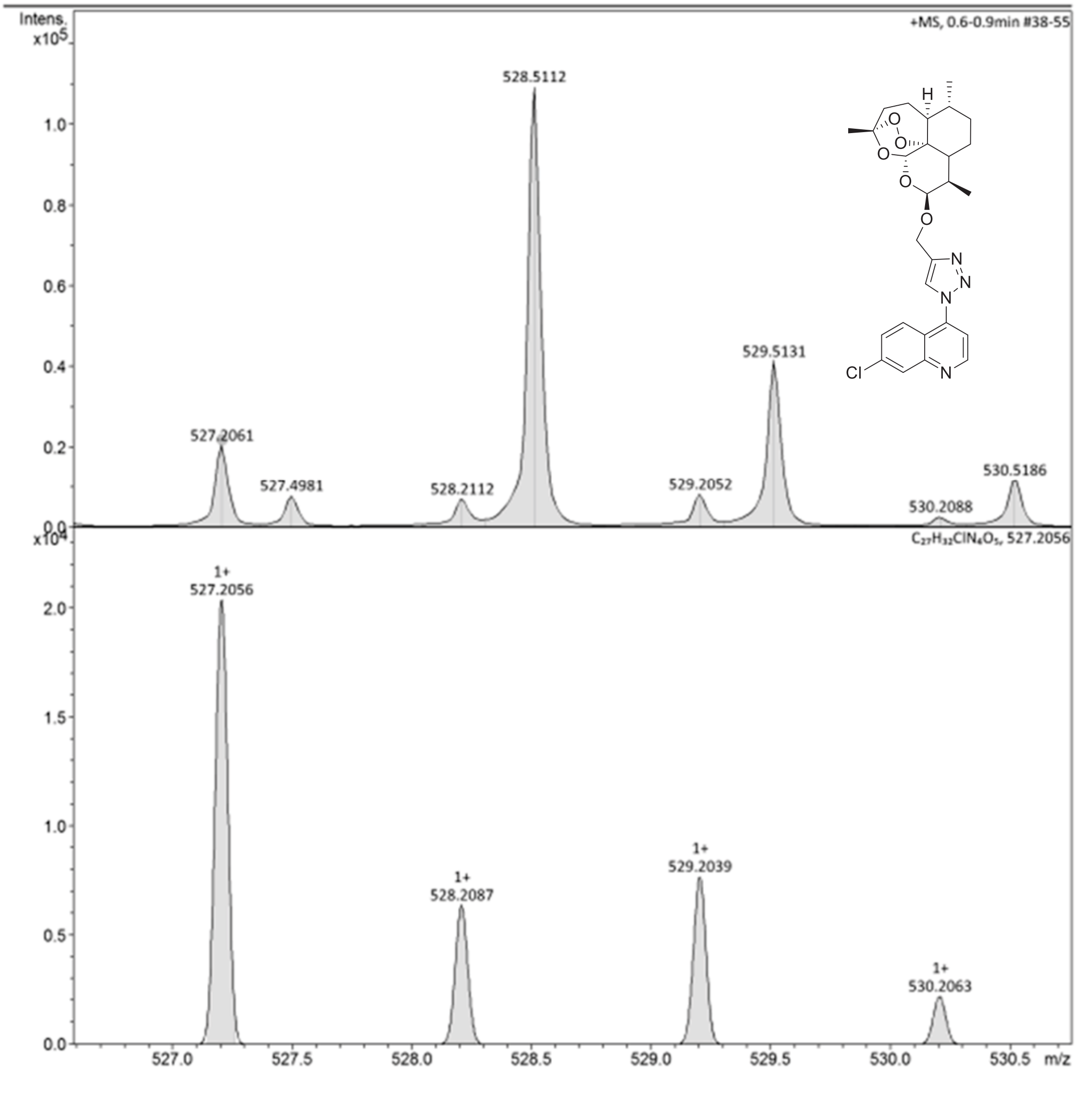

¹H NMR spectrum of hybrid **9** recorded on a Bruker Avance spectrometer (300 MHz, $CDCl_3$):

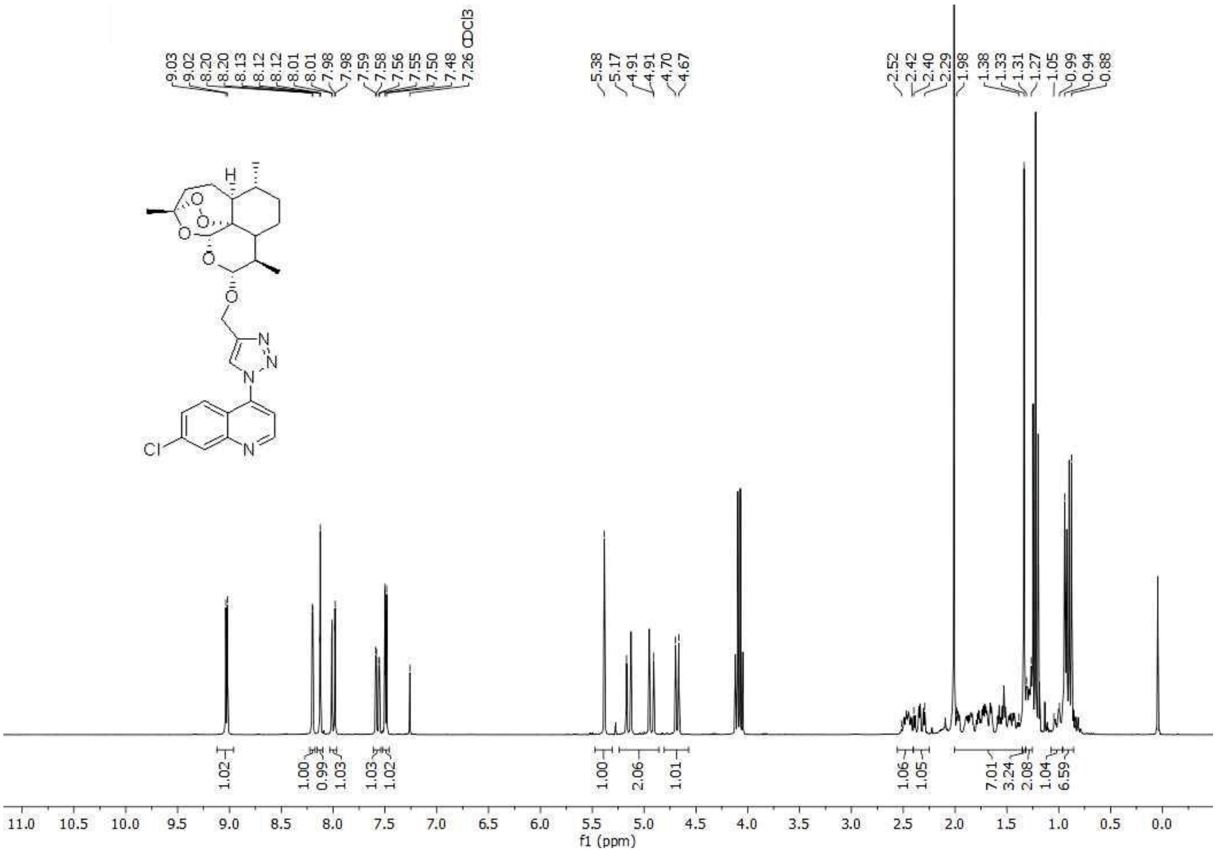


¹³C NMR spectrum of hybrid **9** recorded on a Bruker Avance spectrometer (75 MHz, $CDCl_3$):

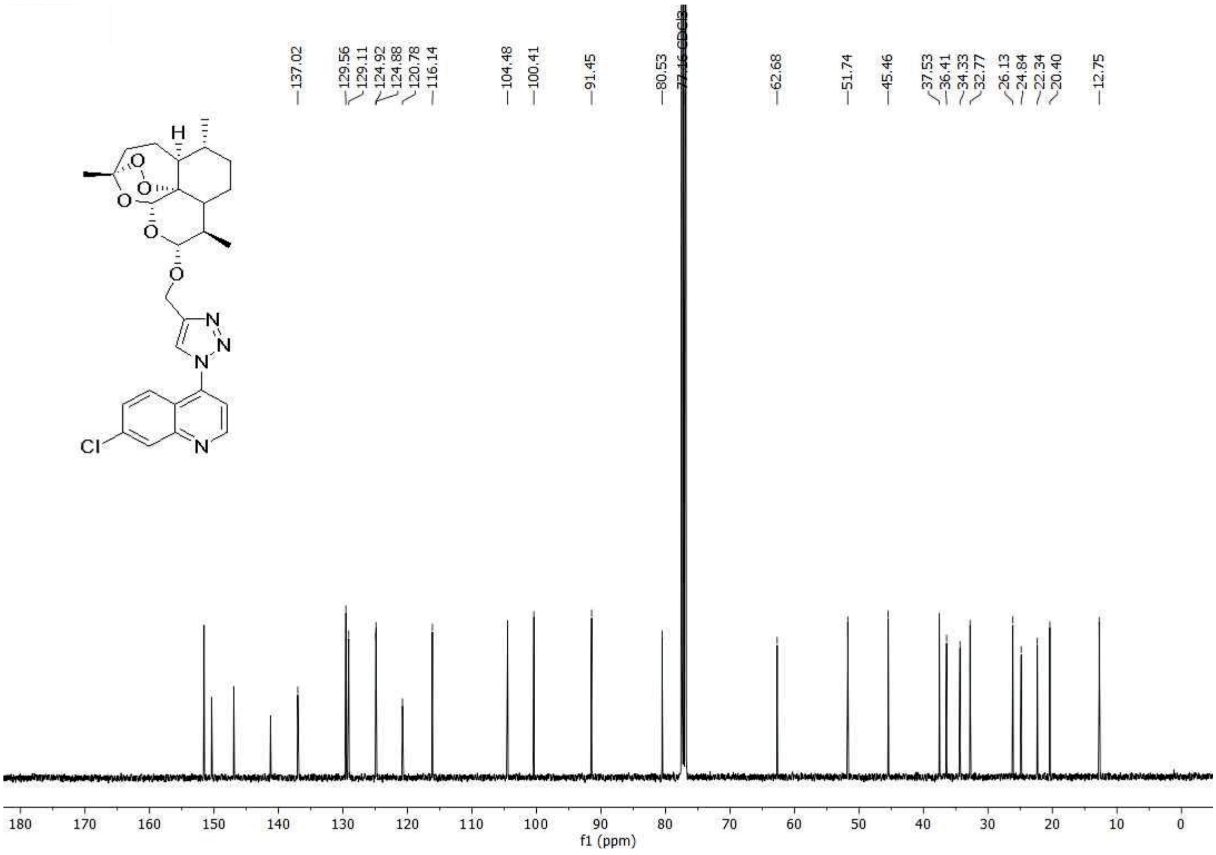

ESI mass spectrum of hybrid **9** recorded on a Bruker micrOTOF II focus TOF MS-spectrometer:

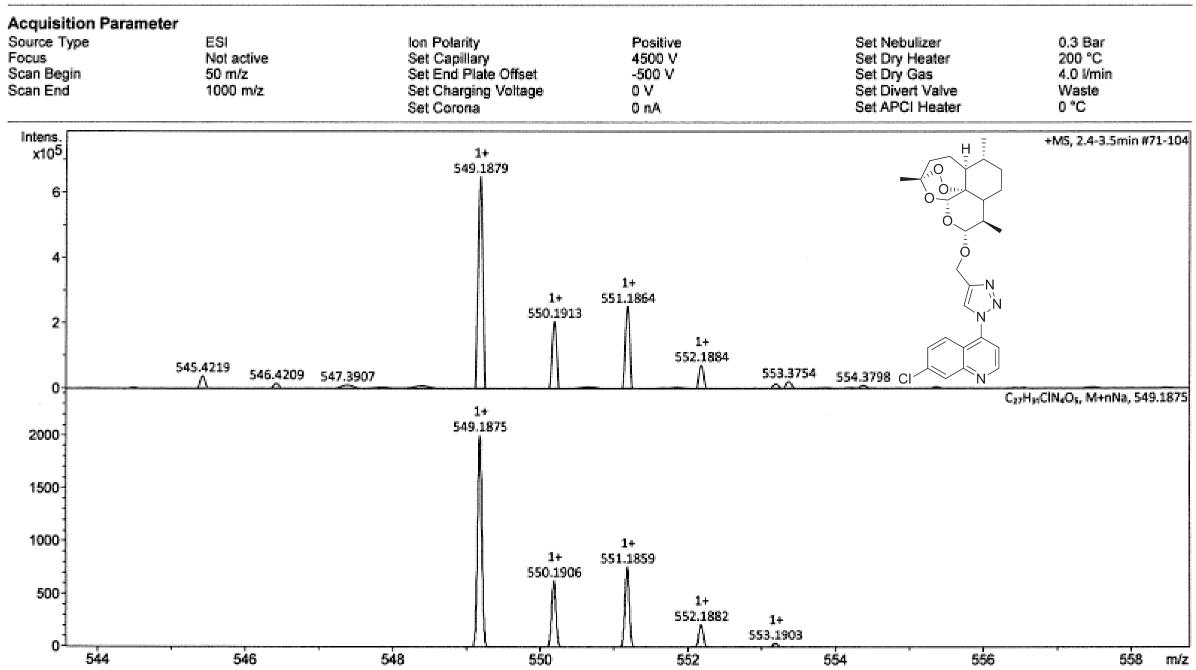


$^{1}$H NMR spectrum of hybrid **10** recorded on a Bruker Avance spectrometer (300 MHz, $CDCl_3$):

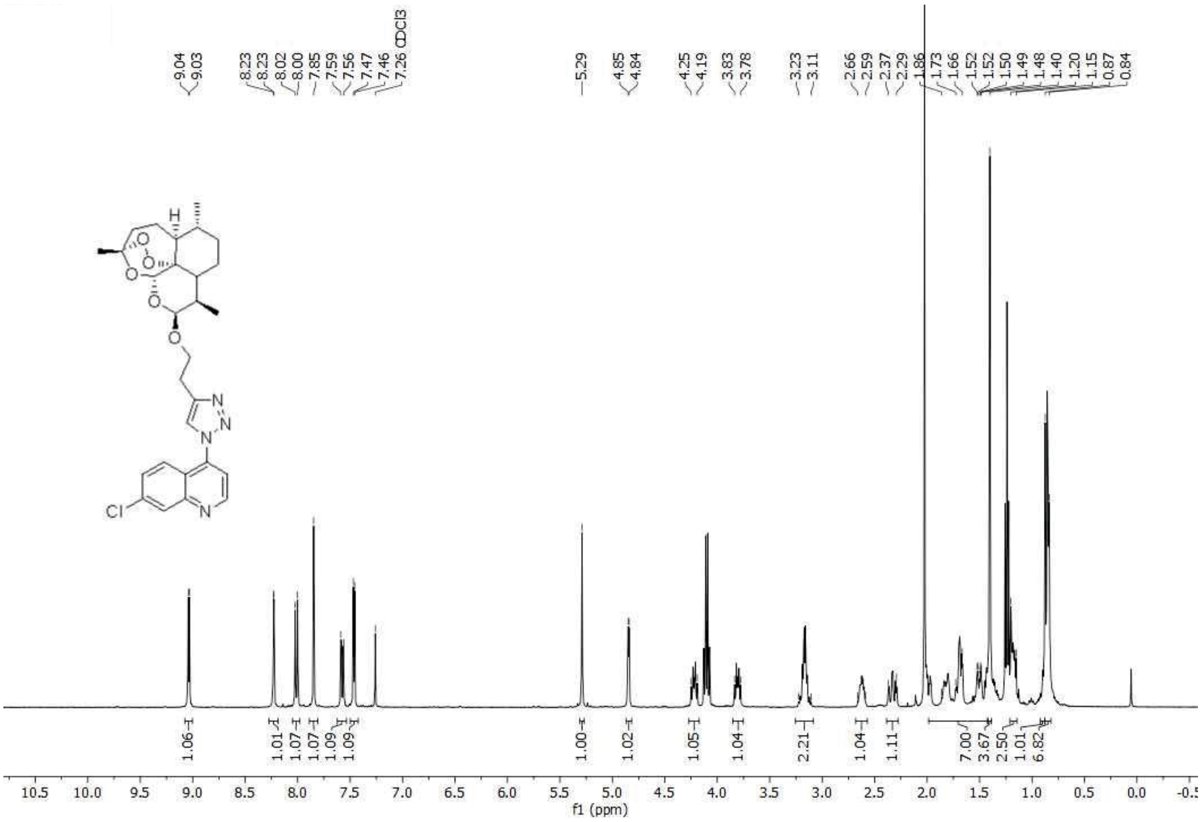

$^{13}C$ NMR spectrum of hybrid **10** recorded on a Bruker Avance spectrometer (100 MHz, DMSO-d$_6$):

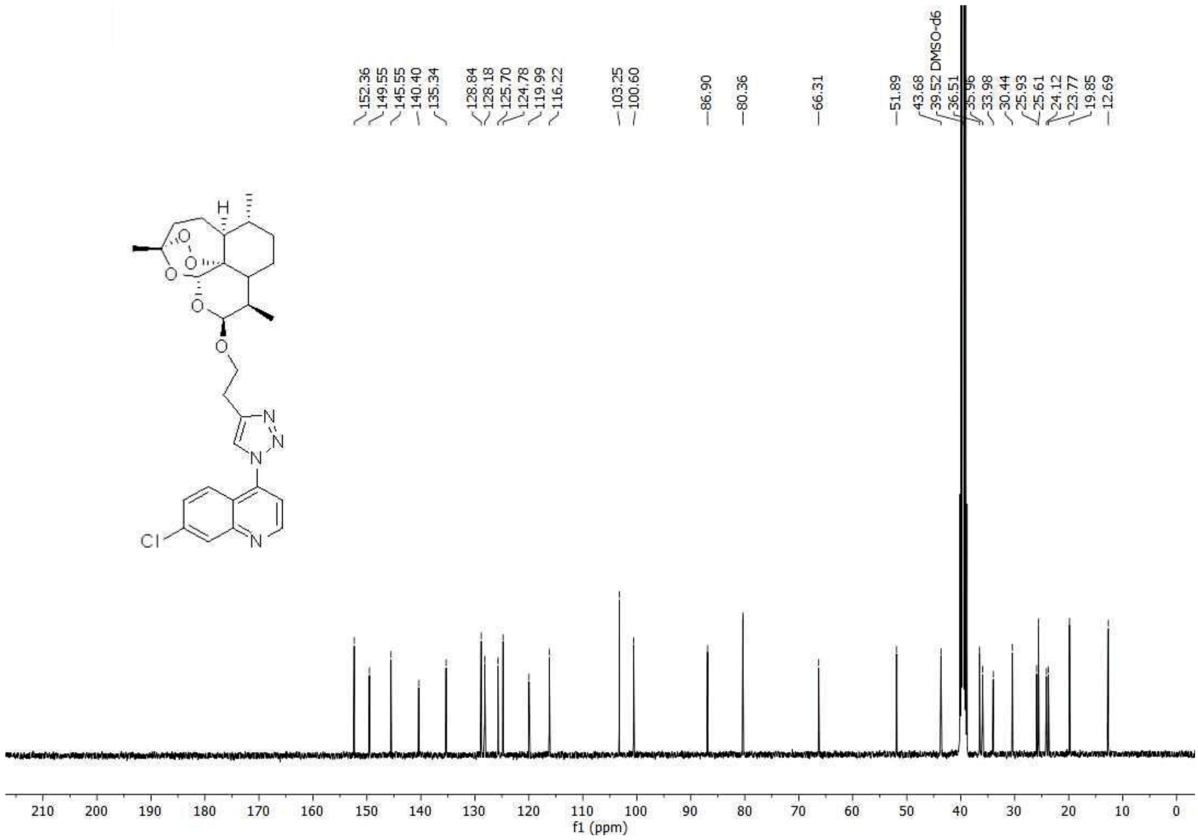


ESI mass spectrum of hybrid **10** recorded on a Bruker micrOTOF II focus TOF MS-spectrometer:

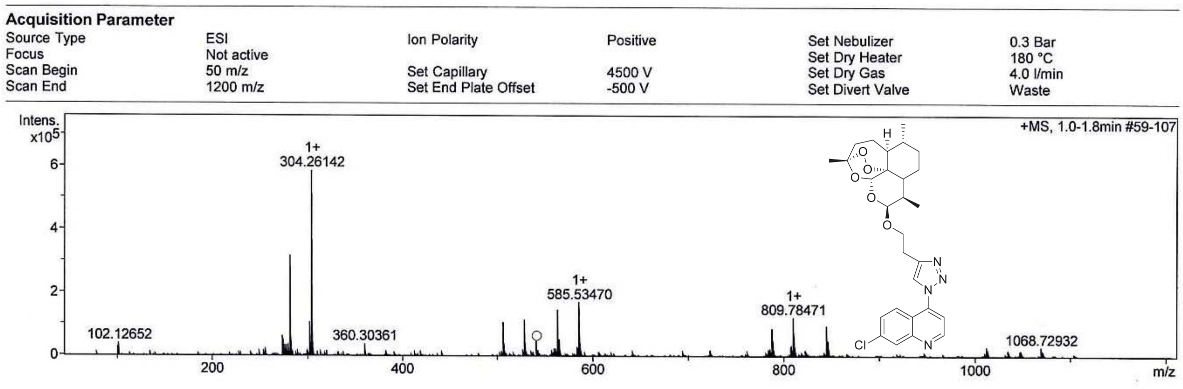


| Meas. m/z | # | Ion Formula | m/z | err [ppm] | Mean err [ppm] | rdb | N-Rule | e⁻ Conf | mSigma | Std I | Std Mean m/z | Std I VarNorm | Std m/z Diff | Std Comb Dev |
|---|---|---|---|---|---|---|---|---|---|---|---|---|---|---|
| 541.22257 | 1 | C28H34ClN4O5 | 541.22122 | -2.49 | 976.54 | 13.5 | ok | even | 9.9 | 13.0 | n.a. | n.a. | n.a. | n.a. |

$^{1}$H NMR spectrum of hybrid **11** recorded on a Bruker Avance spectrometer (300 MHz, $CDCl_3$):

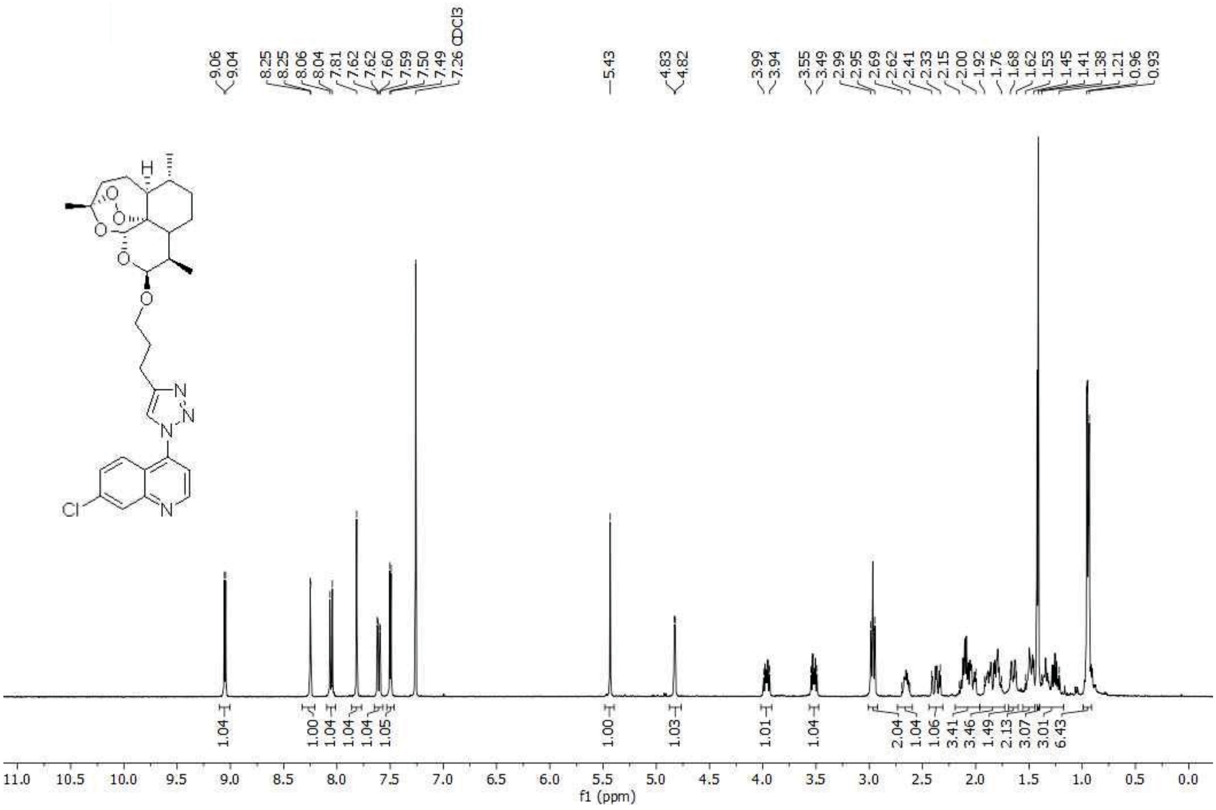


$^{13}$C NMR spectrum of hybrid **11** recorded on a Bruker Avance spectrometer (75 MHz, $CDCl_3$):

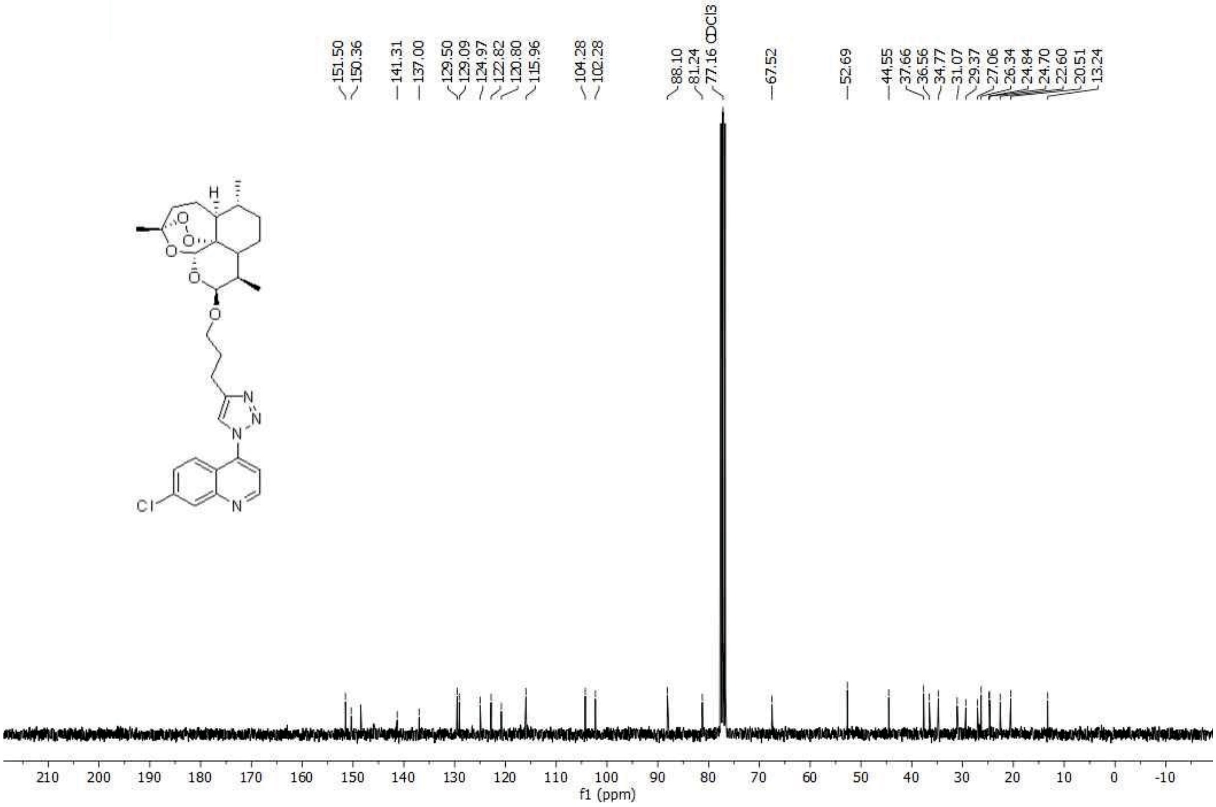

ESI mass spectrum of hybrid **11** recorded on a Bruker micrOTOF II focus TOF MS-spectrometer:

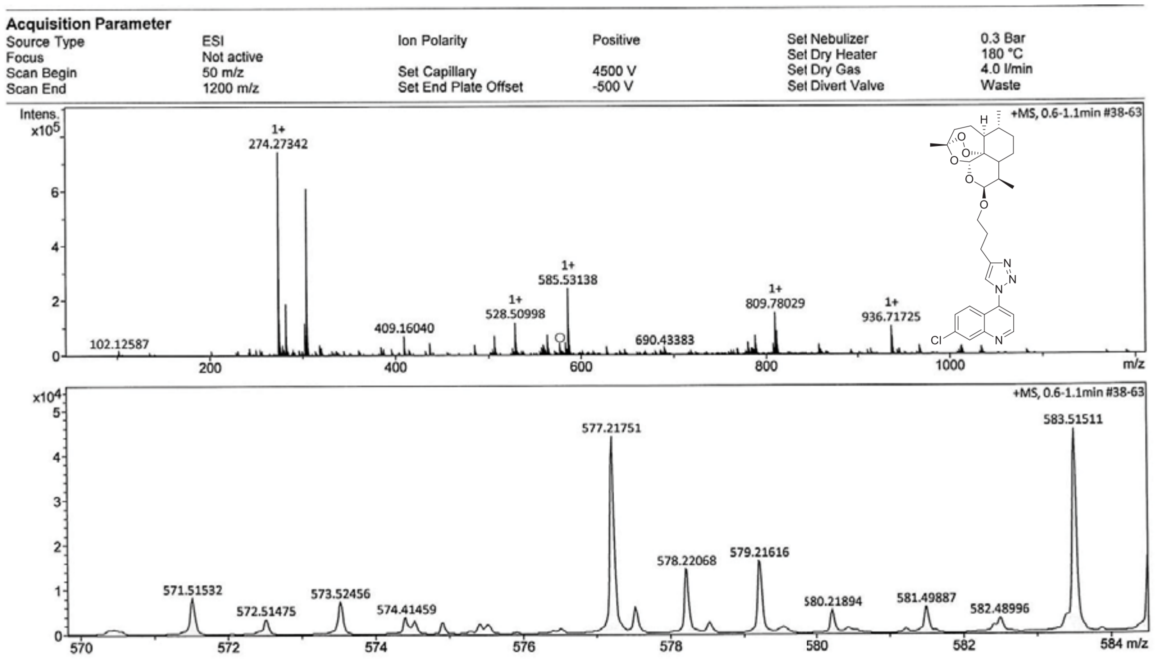


| Meas. m/z | # | Ion Formula | m/z | err [ppm] | Mean err [ppm] | rdb | N-Rule | e⁻ Conf | mSigma | Std I | Std Mean m/z | Std I VarNorm | Std m/z Diff | Std Comb Dev |
|---|---|---|---|---|---|---|---|---|---|---|---|---|---|---|
| 577.21751 | 1 | C29H35ClN4NaO5 | 577.21882 | 2.26 | 918.00 | 13.5 | ok | even | 12.3 | 17.2 | n.a. | n.a. | n.a. | n.a. |

$^1$H NMR spectrum of hybrid **12** recorded on a Bruker Avance spectrometer (300 MHz, $CDCl_3$):

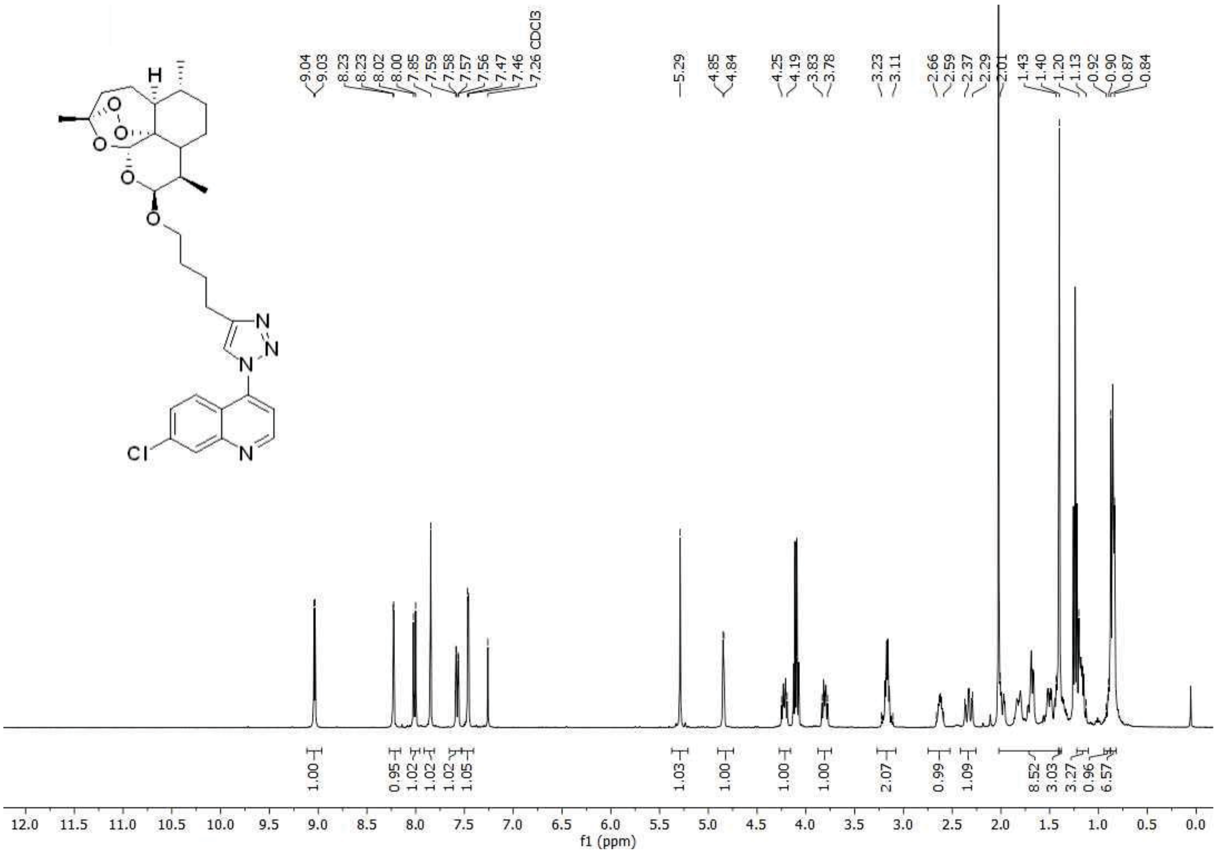

$^{13}C$ NMR spectrum of hybrid **12** recorded on a Bruker Avance spectrometer (75 MHz, $CDCl_3$):

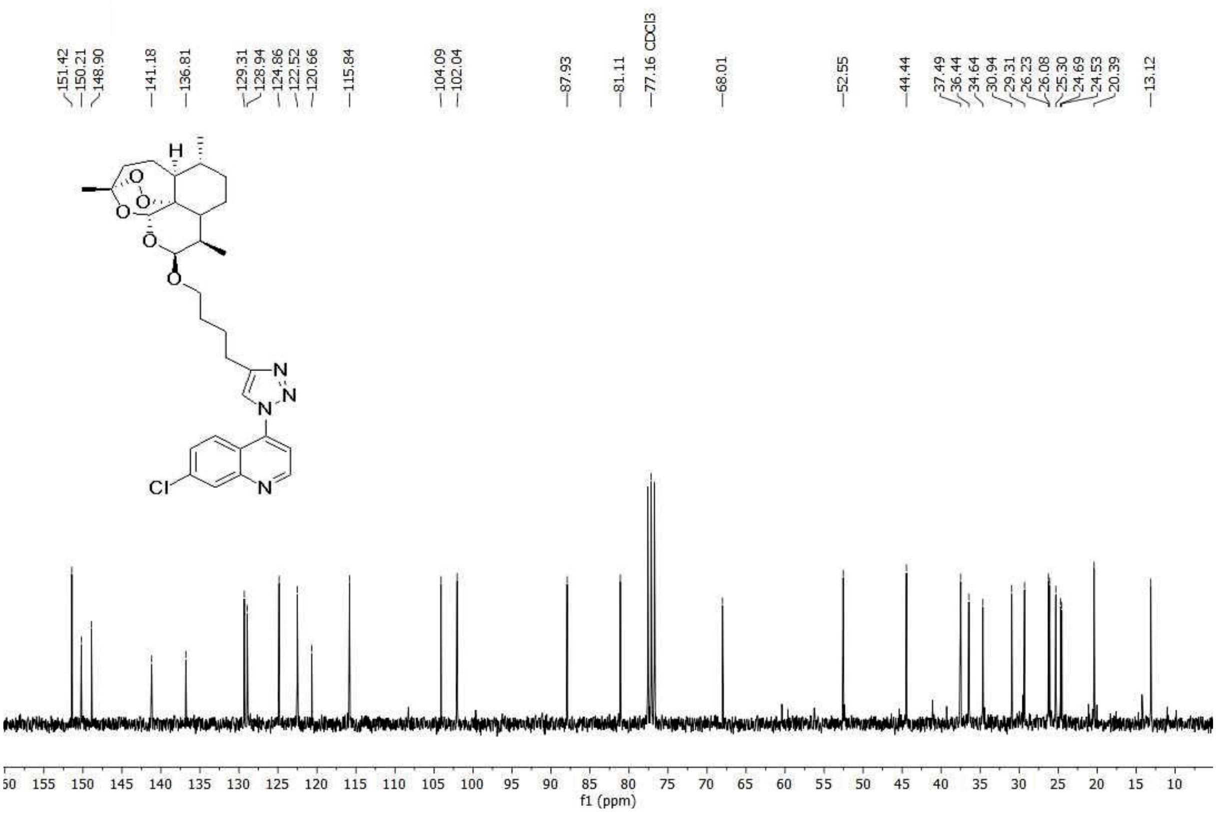


ESI mass spectrum of hybrid **12** recorded on a Bruker micrOTOF II focus TOF MS-spectrometer:

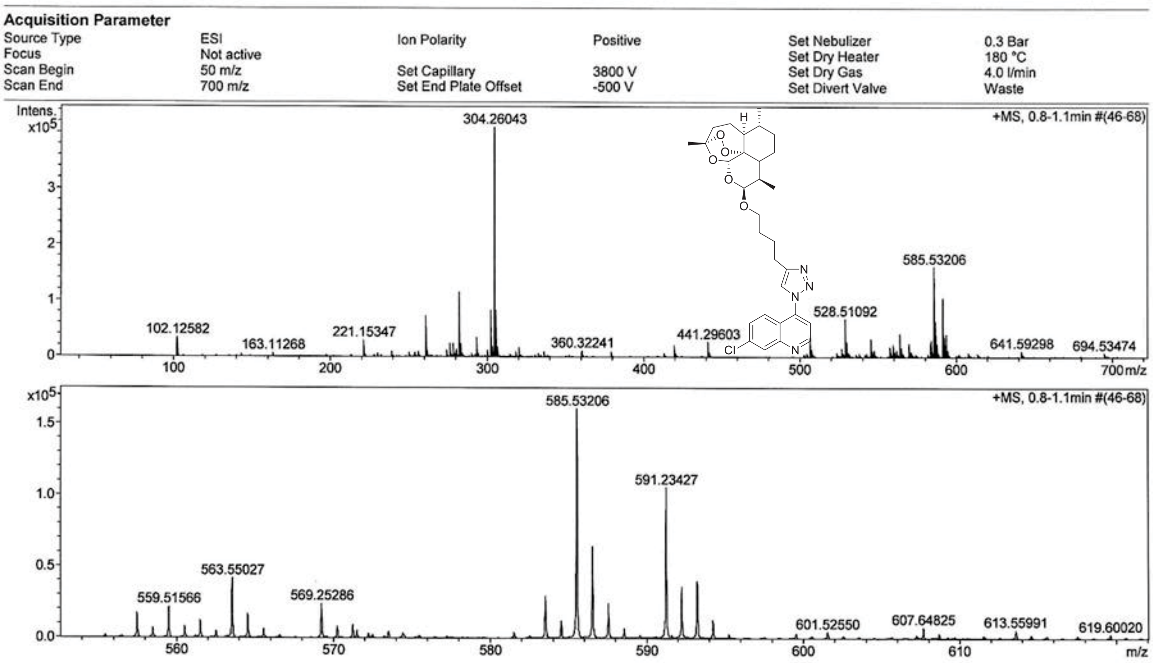


| Meas. m/z | # | Formula | Score | m/z | err [mDa] | err [ppm] | mSigma | rdb | e⁻ Conf | N-Rule |
|---|---|---|---|---|---|---|---|---|---|---|
| 591.23427 | 1 | C 30 H 37 Cl N 4 Na O 5 | 100.00 | 591.23447 | 0.2 | 0.3 | 3.6 | 13.5 | even | ok |

$^{1}$H NMR spectrum of **hybrid 13** recorded on a Bruker Avance spectrometer (400 MHz, $CDCl_3$):

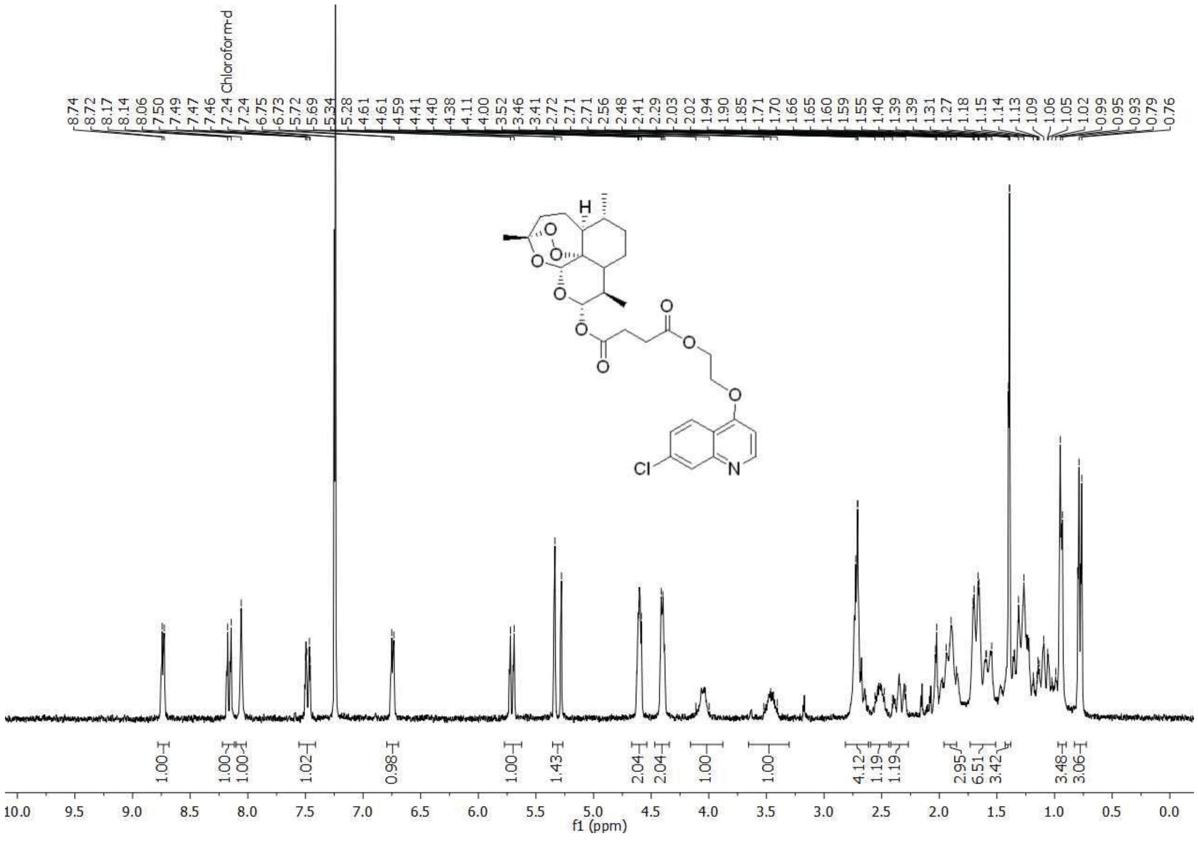


$^{13}$C NMR spectrum of **hybrid 13** recorded on a Bruker Avance spectrometer (100 MHz, $CDCl_3$):

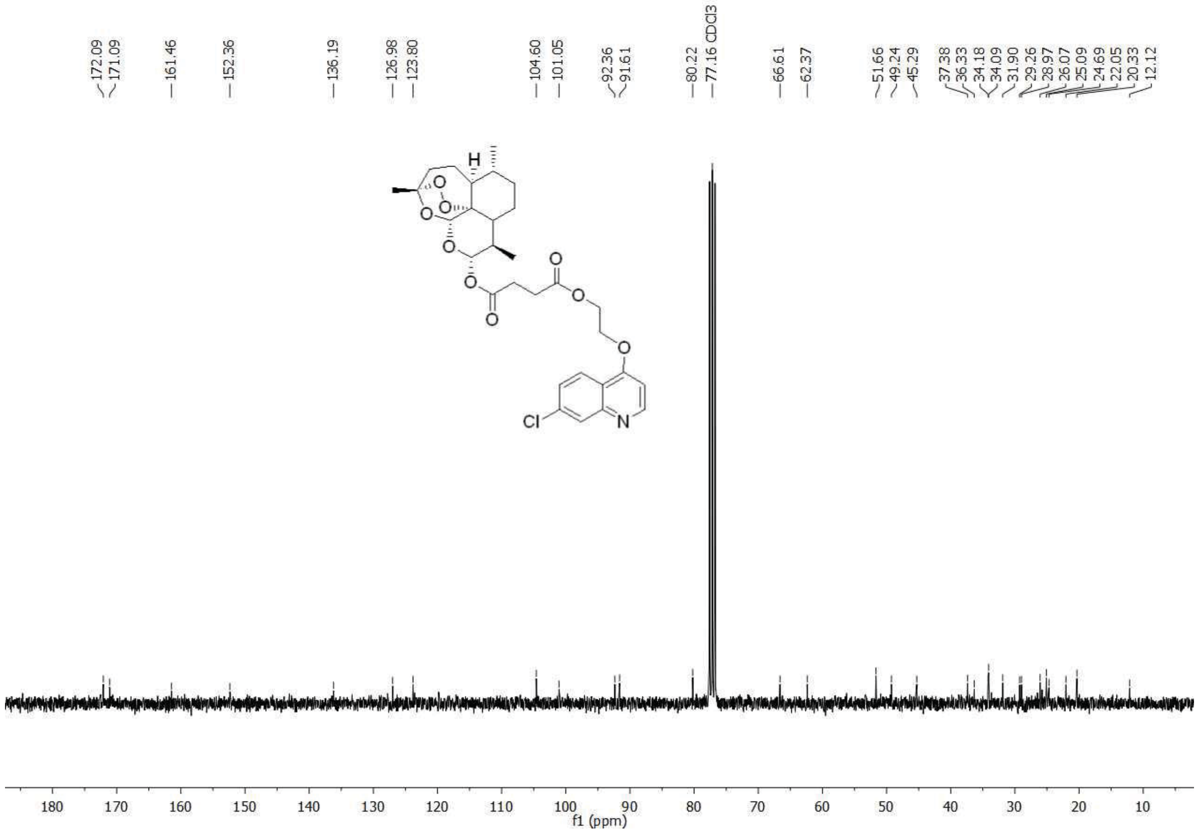

EI mass spectrum of **hybrid 13** recorded on a Bruker micrOTOF II focus TOF MS-spectrometer:

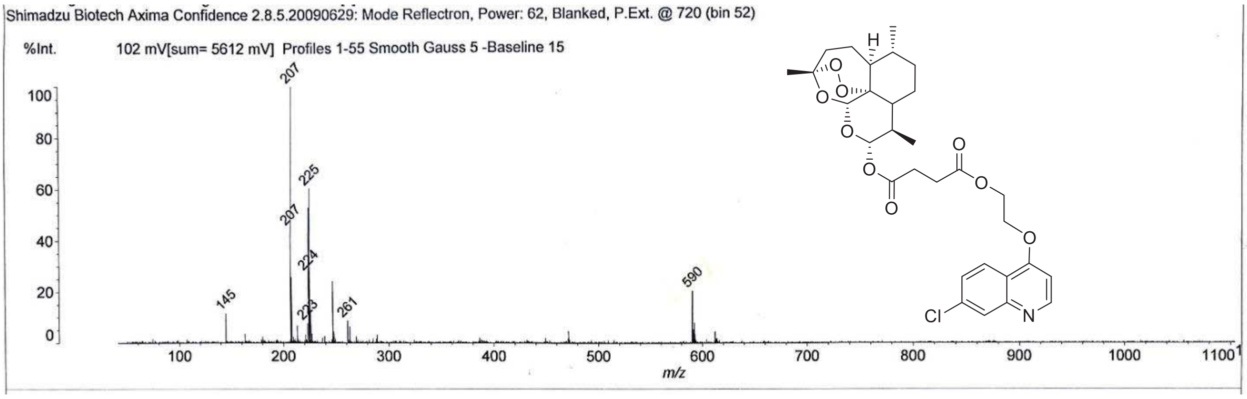


$^1H$ NMR spectrum of **hybrid 14** recorded on a Bruker Avance spectrometer (400 MHz, $CDCl_3$):

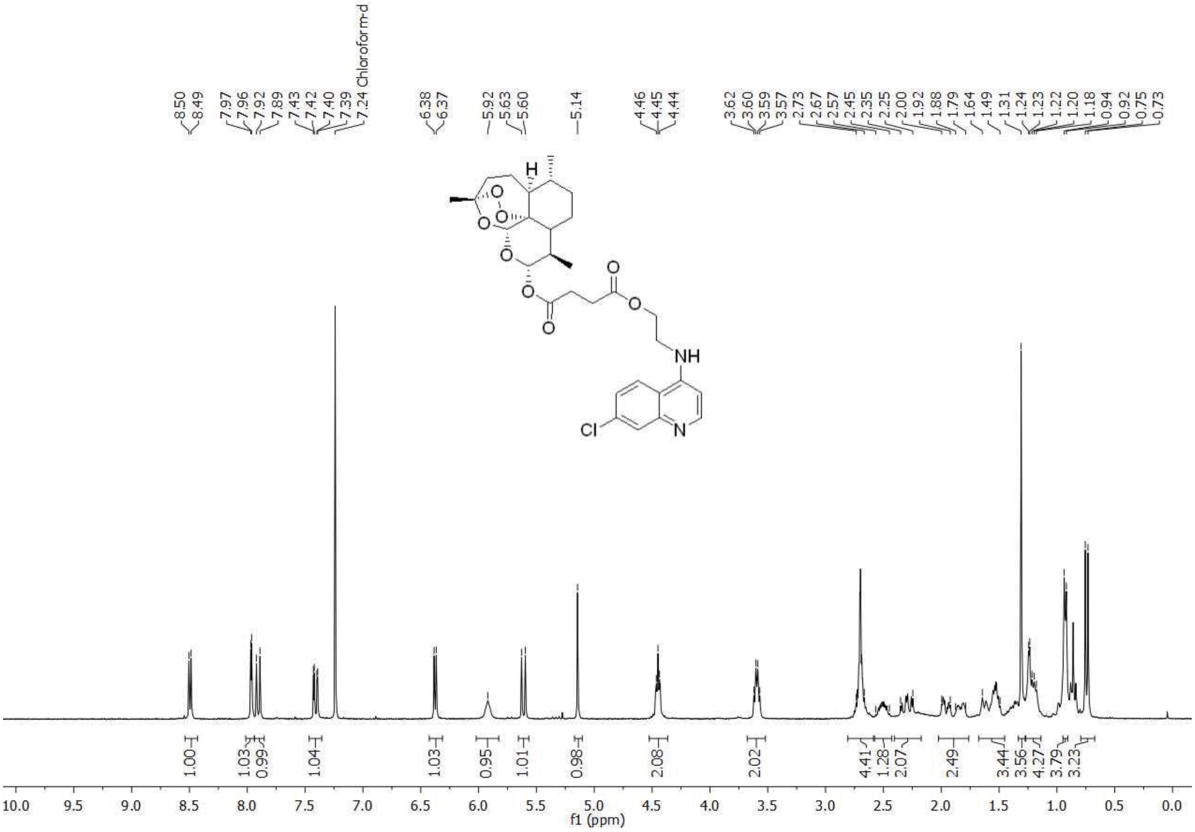

$^{13}$C NMR spectrum of **hybrid 14** recorded on a Bruker Avance spectrometer (100 MHz, $CDCl_3$):

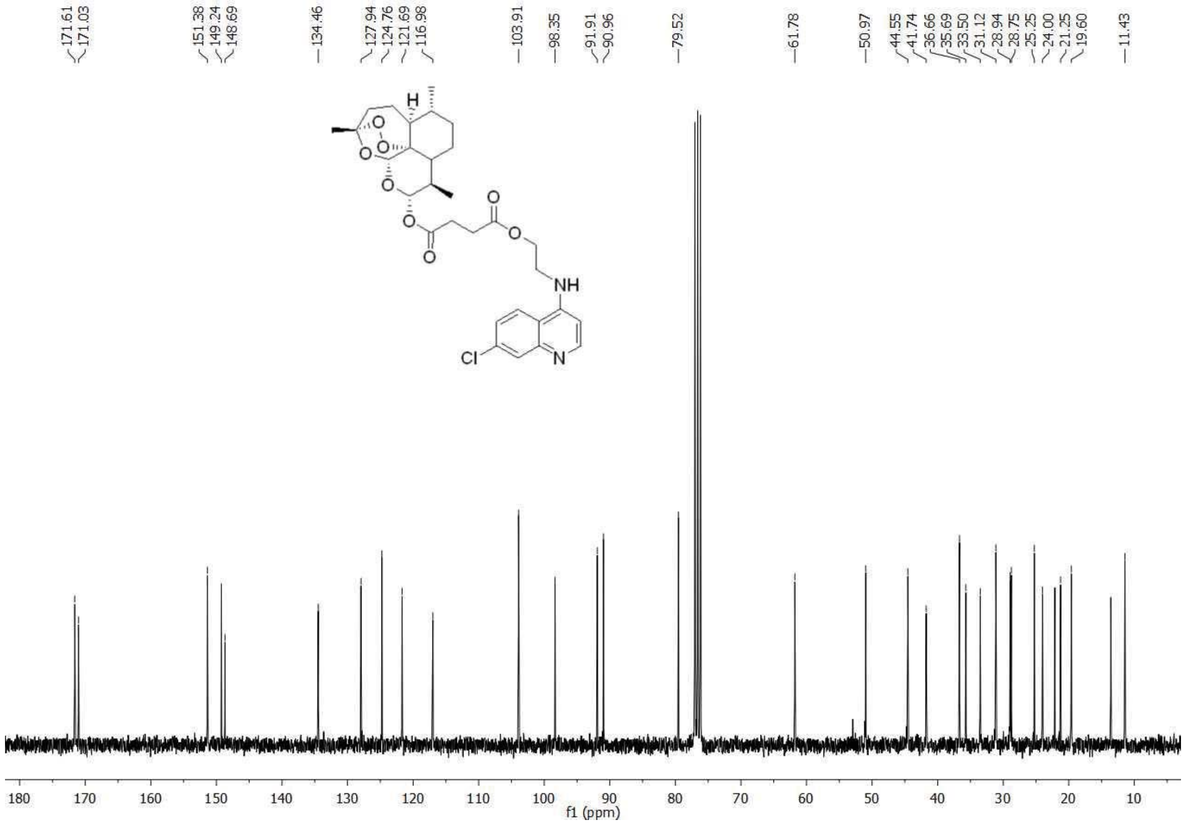


ESI mass spectrum of **hybrid 14** recorded on a Bruker micrOTOF II focus TOF MS-spectrometer:

**Acquisition Parameter**

| | | | | | |
|---|---|---|---|---|---|
| Source Type | ESI | Ion Polarity | Positive | Set Nebulizer | 0.3 Bar |
| Focus | Not active | | | Set Dry Heater | 200 °C |
| Scan Begin | 50 m/z | Set Capillary | 4500 V | Set Dry Gas | 4.0 l/min |
| Scan End | 1250 m/z | Set End Plate Offset | -500 V | Set Divert Valve | Waste |

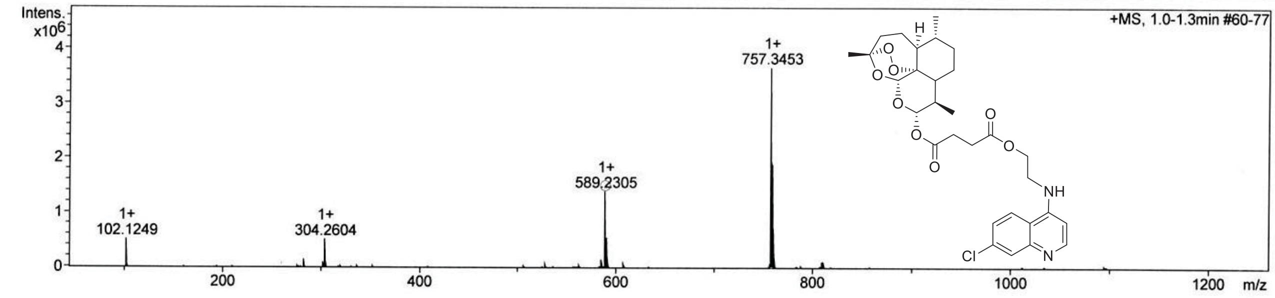


| Meas. m/z | # | Ion Formula | m/z | err [ppm] | Mean err [ppm] | rdb | N-Rule | e⁻ Conf | mSigma | Std I | Std Mean m/z | Std I VarNorm | Std m/z Diff | Std Comb Dev |
|---|---|---|---|---|---|---|---|---|---|---|---|---|---|---|
| 589.230489 | 1 | C30H38ClN2O8 | 589.231120 | 1.1 | 1.1 | 12.5 | ok | even | 6.8 | 8.3 | n.a. | n.a. | n.a. | n.a. |

$^{1}H$ NMR spectrum of **hybrid 15** recorded on a Bruker Avance spectrometer (400 MHz, $CDCl_3$):

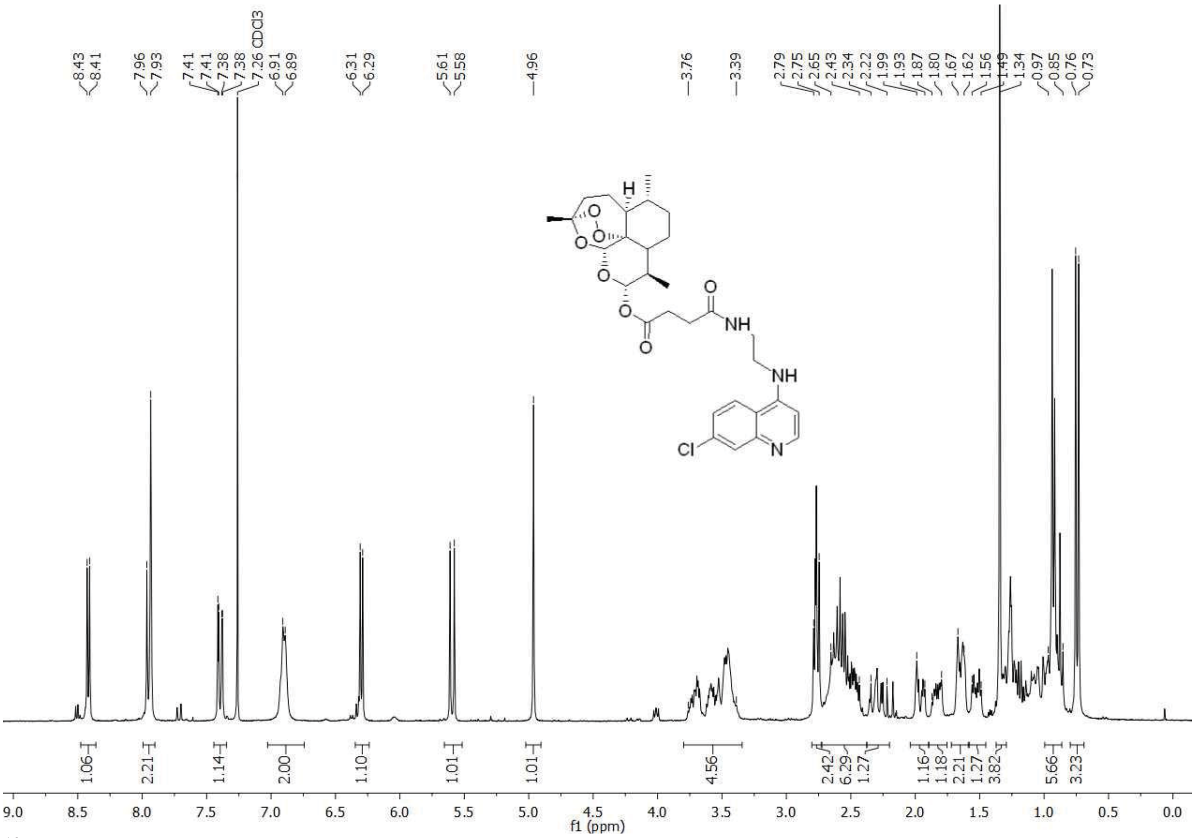


$^{13}C$ NMR spectrum of **hybrid 15** recorded on a Bruker Avance spectrometer (100 MHz, $CDCl_3$):

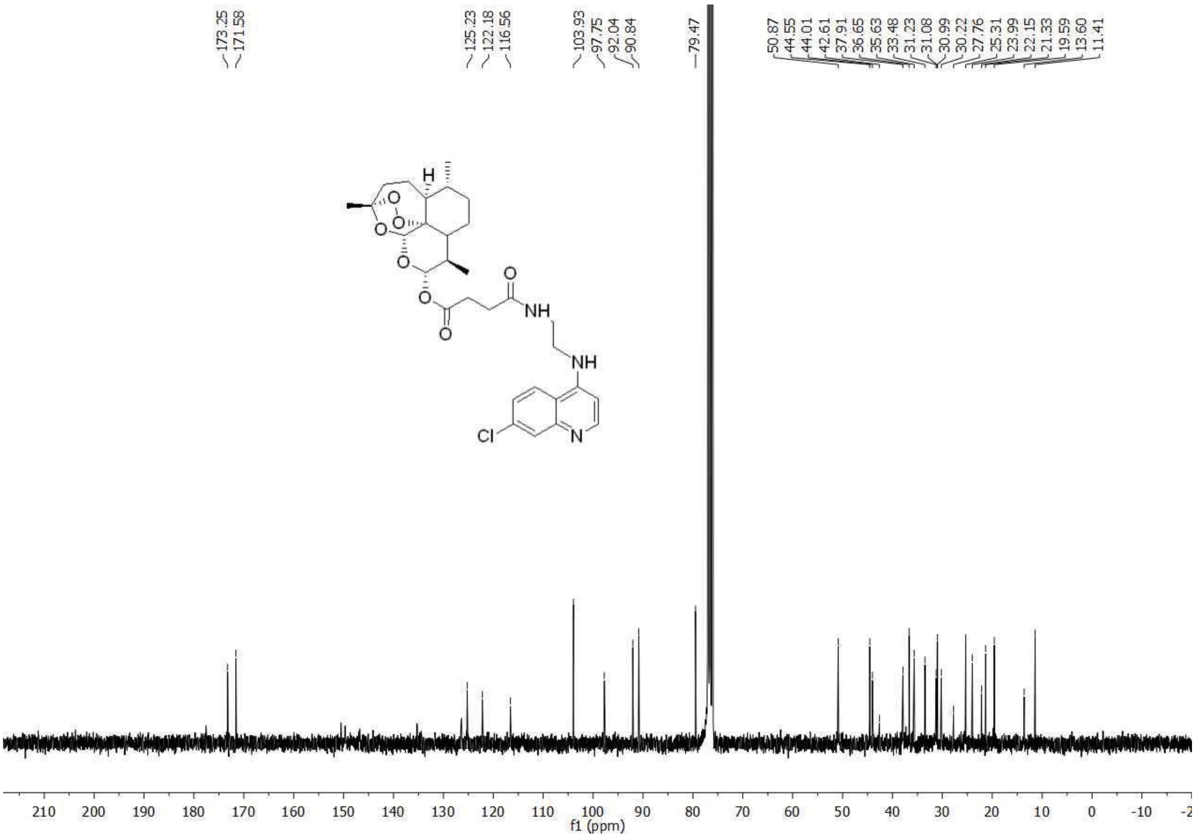

ESI mass spectrum of **hybrid 15** recorded on a Bruker micrOTOF II focus TOF MS-spectrometer:

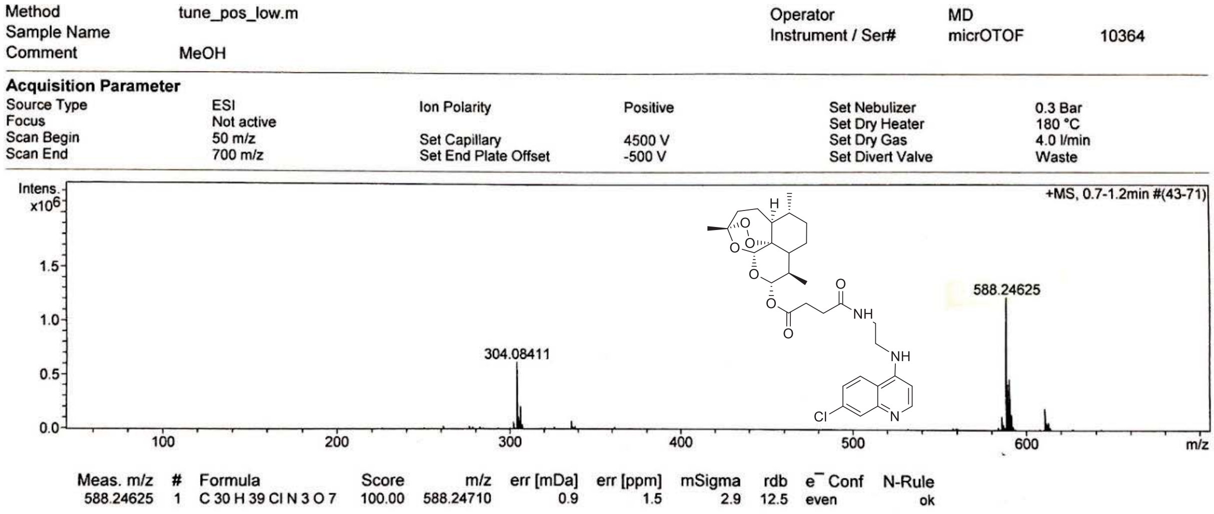


$^1$H NMR spectrum of **hybrid 16** recorded on a Bruker Avance spectrometer (400 MHz, $CDCl_3$):

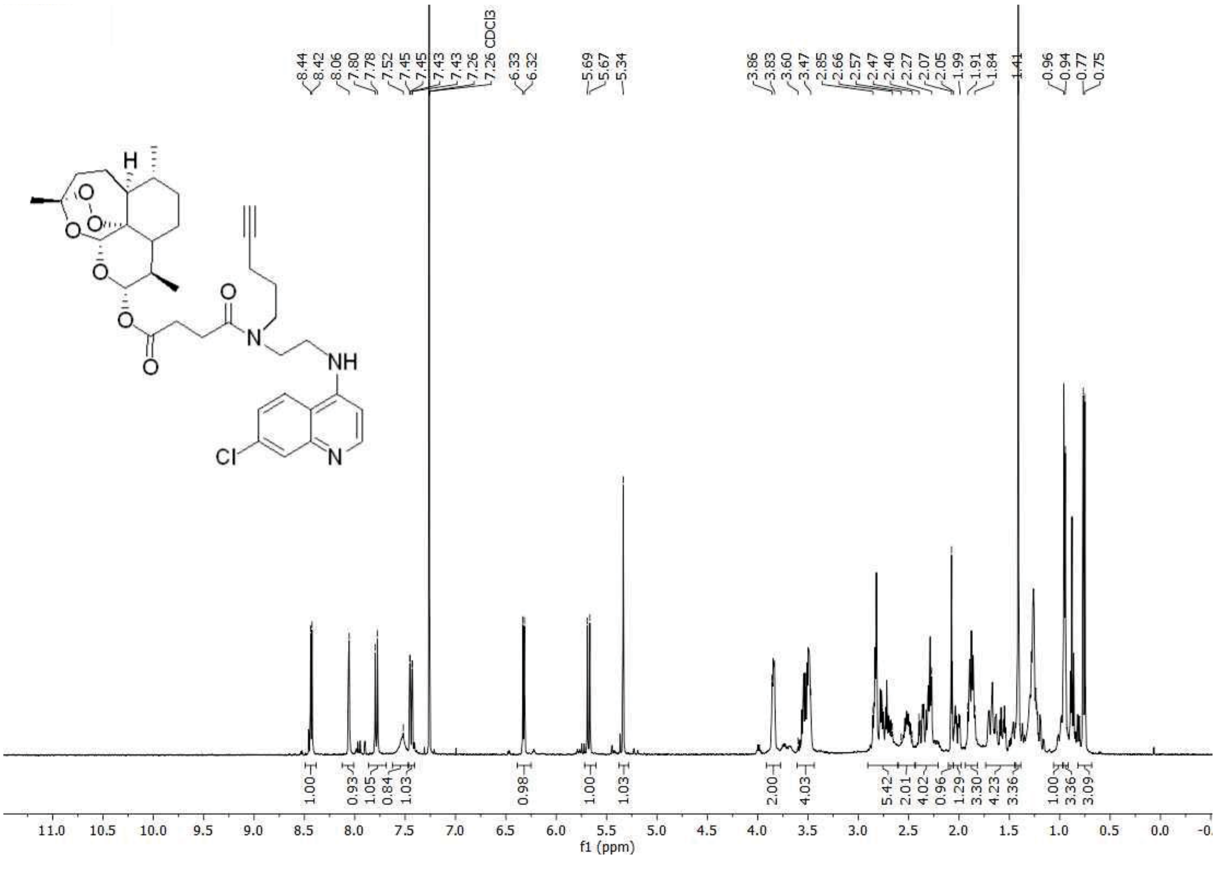

$^{13}C$ NMR spectrum of **hybrid 16** recorded on a Bruker Avance spectrometer (125 MHz, $CDCl_3$):

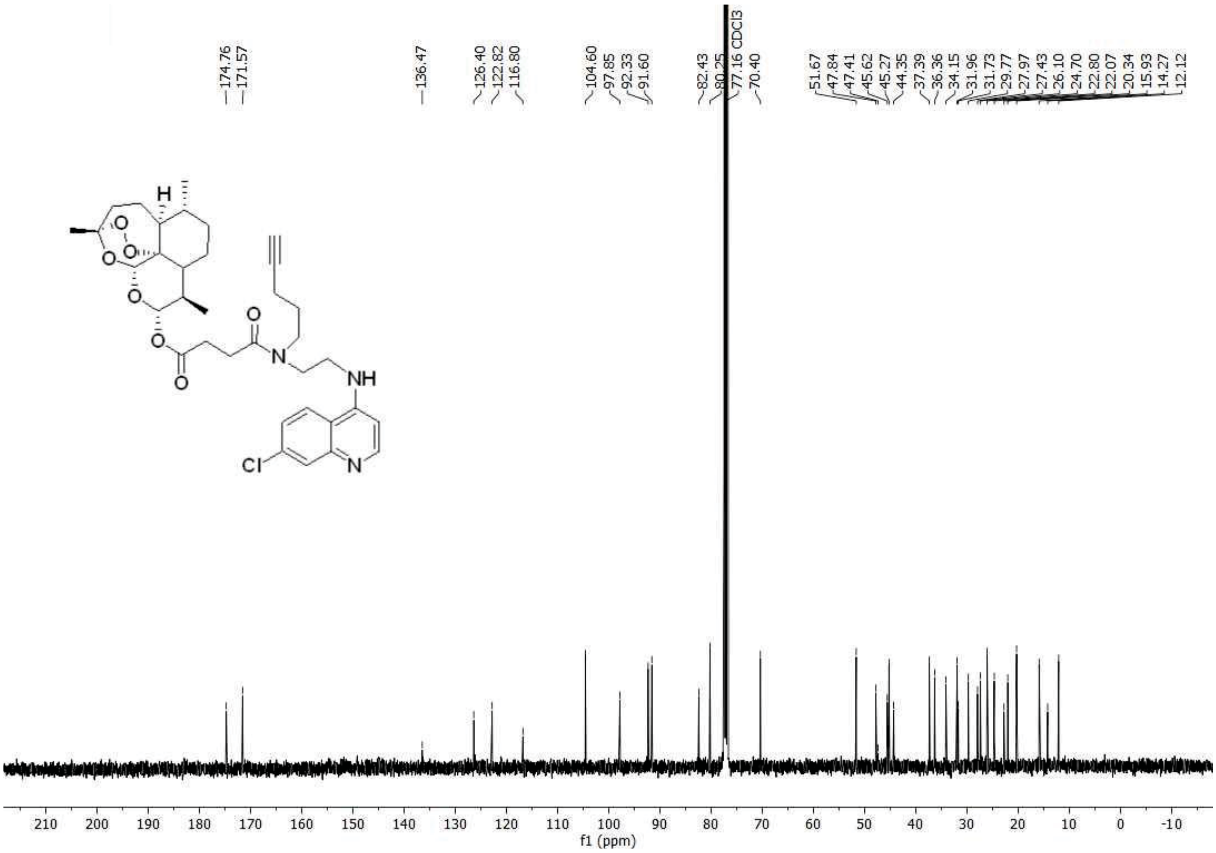


ESI mass spectrum of **hybrid 16** recorded on a Bruker micrOTOF II focus TOF MS-spectrometer:

| | | | | |
|---|---|---|---|---|
| Method | tune_low-APPI.m | Operator | MD | |
| Sample Name | | Instrument | maXis | 288882.20183 |
| Comment | ,ACN,CH2Cl2 | | | |

**Acquisition Parameter**

| | | | | | |
|---|---|---|---|---|---|
| Source Type | APPI | Ion Polarity | Positive | Set Nebulizer | 2.5 Bar |
| Focus | Not active | Set Capillary | 700 V | Set Dry Heater | 220 °C |
| Scan Begin | 50 m/z | Set End Plate Offset | -500 V | Set Dry Gas | 1.5 l/min |
| Scan End | 1550 m/z | Set Charging Voltage | 0 V | Set Divert Valve | Waste |
| | | Set Corona | 0 nA | Set APCI Heater | 250 °C |

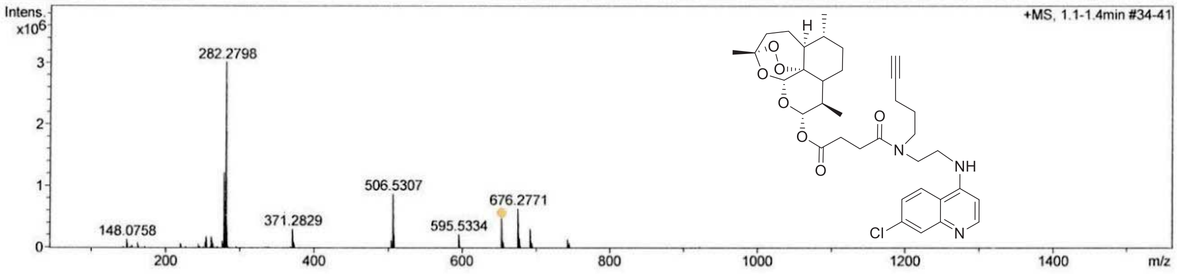


| Meas. m/z | # | Ion Formula | m/z | err [ppm] | mSigma | # mSigma | Score | rdb | e⁻ Conf | N-Rule |
|---|---|---|---|---|---|---|---|---|---|---|
| 654.2950 | 1 | C35H45ClN3O7 | 654.2941 | -1.4 | 119.5 | 1 | 100.00 | 14.5 | even | ok |

$^{1}H$ NMR spectrum of **hybrid 17** recorded on a Bruker Avance spectrometer (600 MHz, CDCl3):

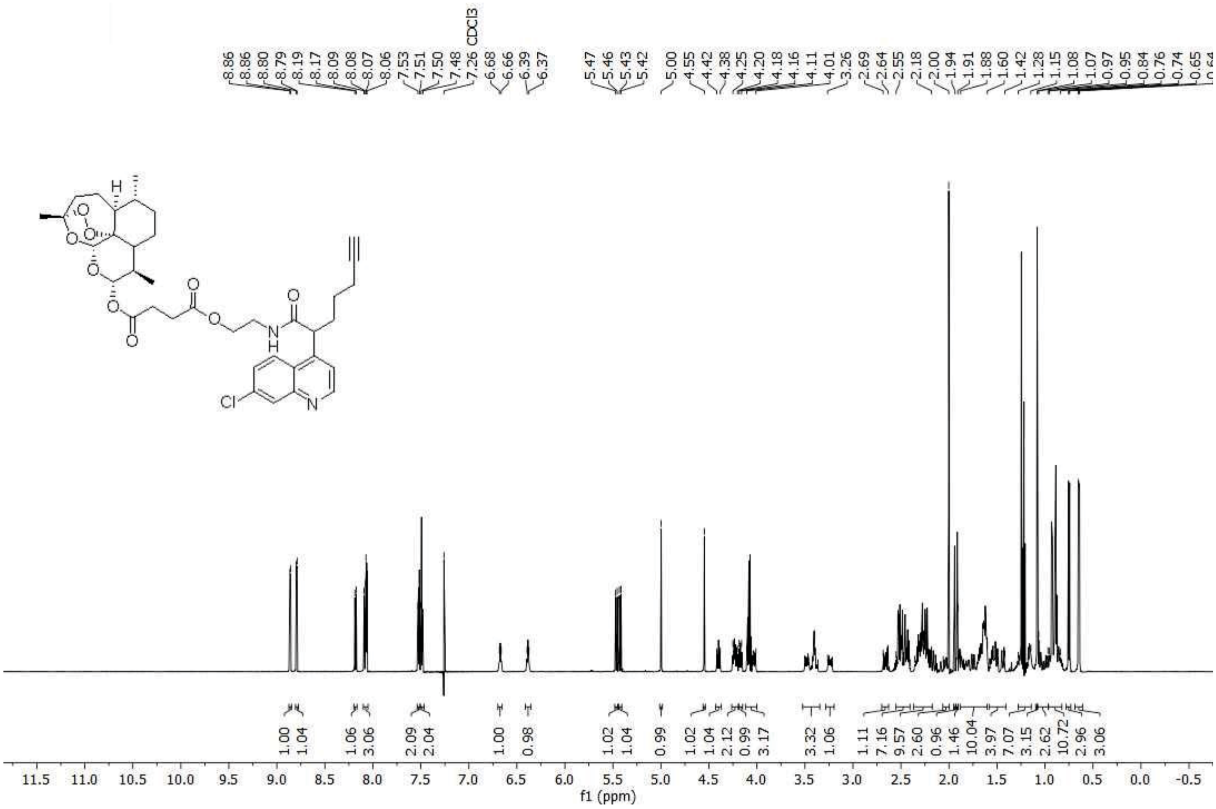


$^{13}C$ NMR spectrum of **hybrid 17** recorded on a Bruker Avance spectrometer (125 MHz, $CDCl_3$):

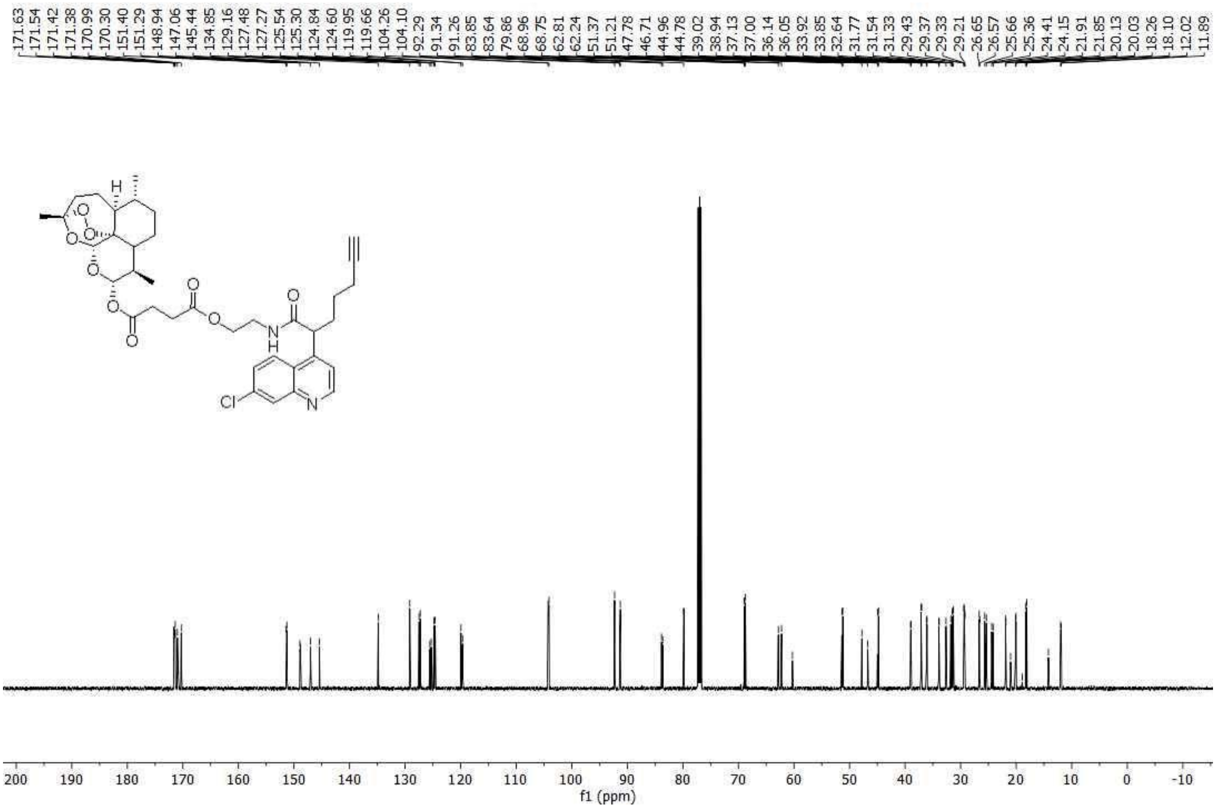

ESI mass spectrum of **hybrid 17** recorded on a Bruker micrOTOF II focus TOF MS-spectrometer:

**Acquisition Parameter**

| | | | | | |
|---|---|---|---|---|---|
| Source Type | APPI | Ion Polarity | Positive | Set Nebulizer | 2.2 Bar |
| Focus | Not active | Set Capillary | 800 V | Set Dry Heater | 200 °C |
| Scan Begin | 200 m/z | Set End Plate Offset | -500 V | Set Dry Gas | 1.5 l/min |
| Scan End | 1600 m/z | Set Charging Voltage | 0 V | Set Divert Valve | Waste |
| | | Set Corona | 0 nA | Set APCI Heater | 400 °C |

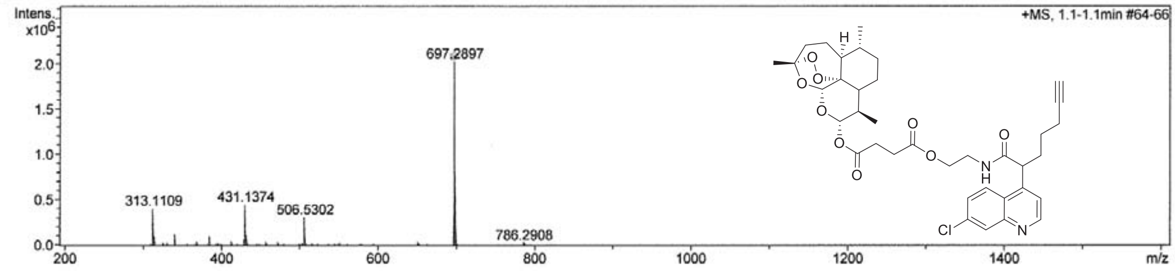


| Meas. m/z | # | Ion Formula | m/z | err [ppm] | mSigma | # mSigma | Score | rdb | e⁻ Conf | N-Rule |
|---|---|---|---|---|---|---|---|---|---|---|
| 697.2897 | 1 | C37H46ClN2O9 | 697.2886 | -1.5 | 69.6 | 1 | 100.00 | 15.5 | even | ok |

## 6. NMR spectra of the compounds after stability analysis:

$^1$H NMR spectrum of hybrid **13** recorded (400 MHz, DMSO-d$_6$) after heating 60 h at 60 °C:

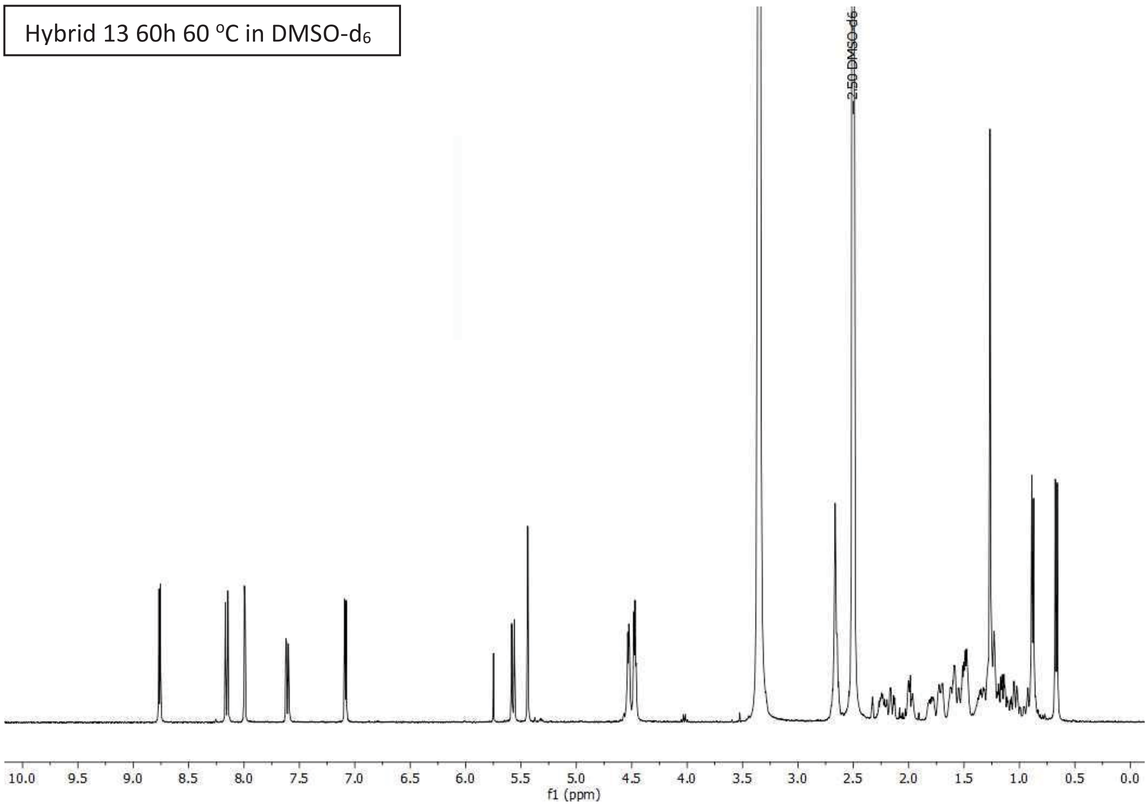

$^{1}H$ NMR spectrum of hybrid **14** recorded (400 MHz, DMSO-$d_6$) before heating 60 h at 60 °C:

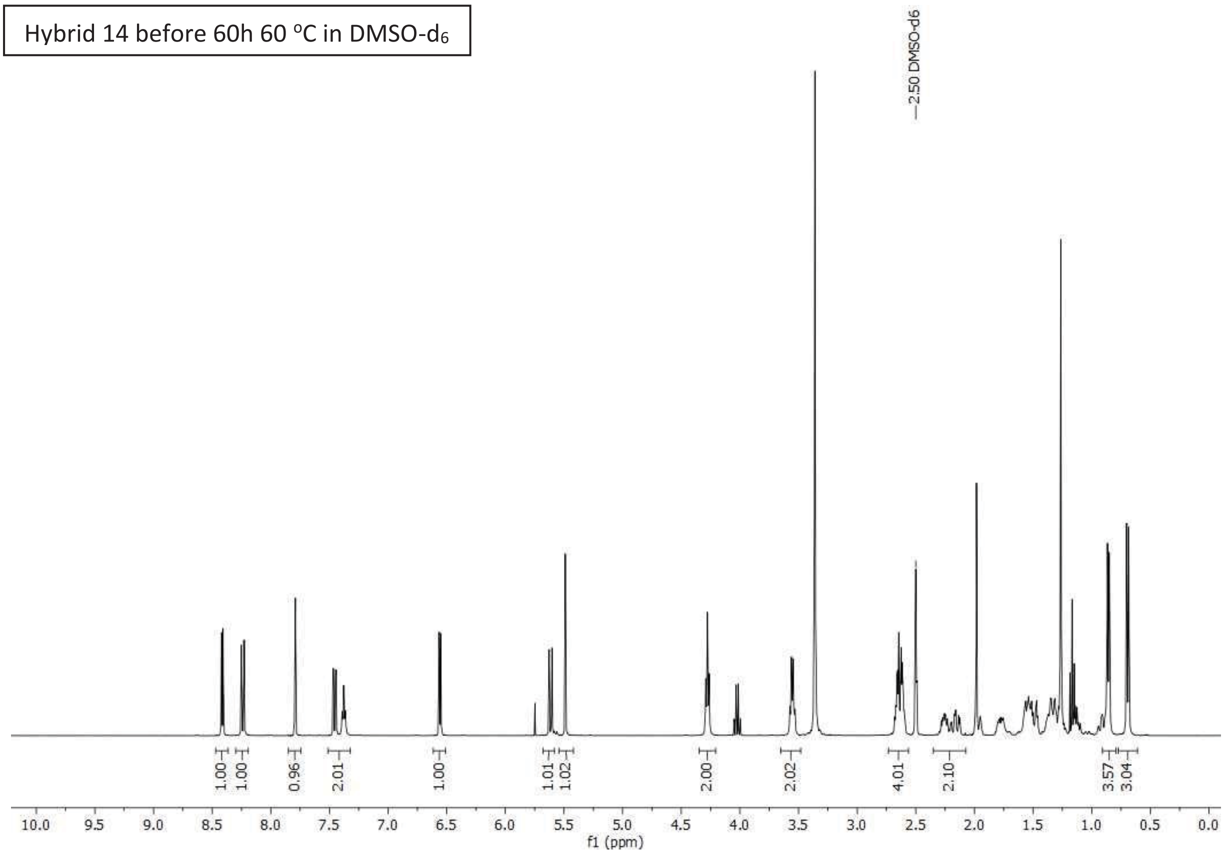


$^{1}H$ NMR spectrum of hybrid **14** recorded (400 MHz, DMSO-$d_6$) after heating 60 h at 60 °C:

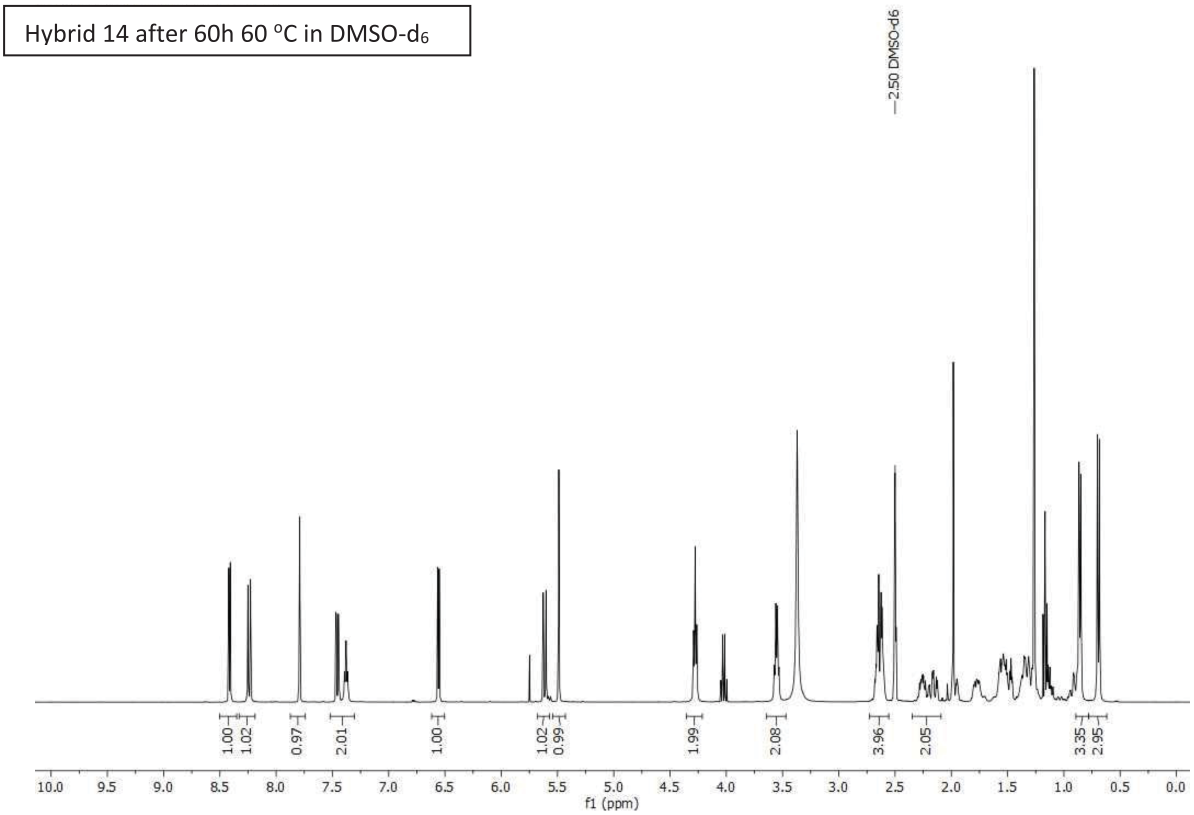

$^{13}$C NMR spectrum of hybrid **14** recorded (400 MHz, DMSO-$d_6$) before and after heating 60 h at 60 ºC:

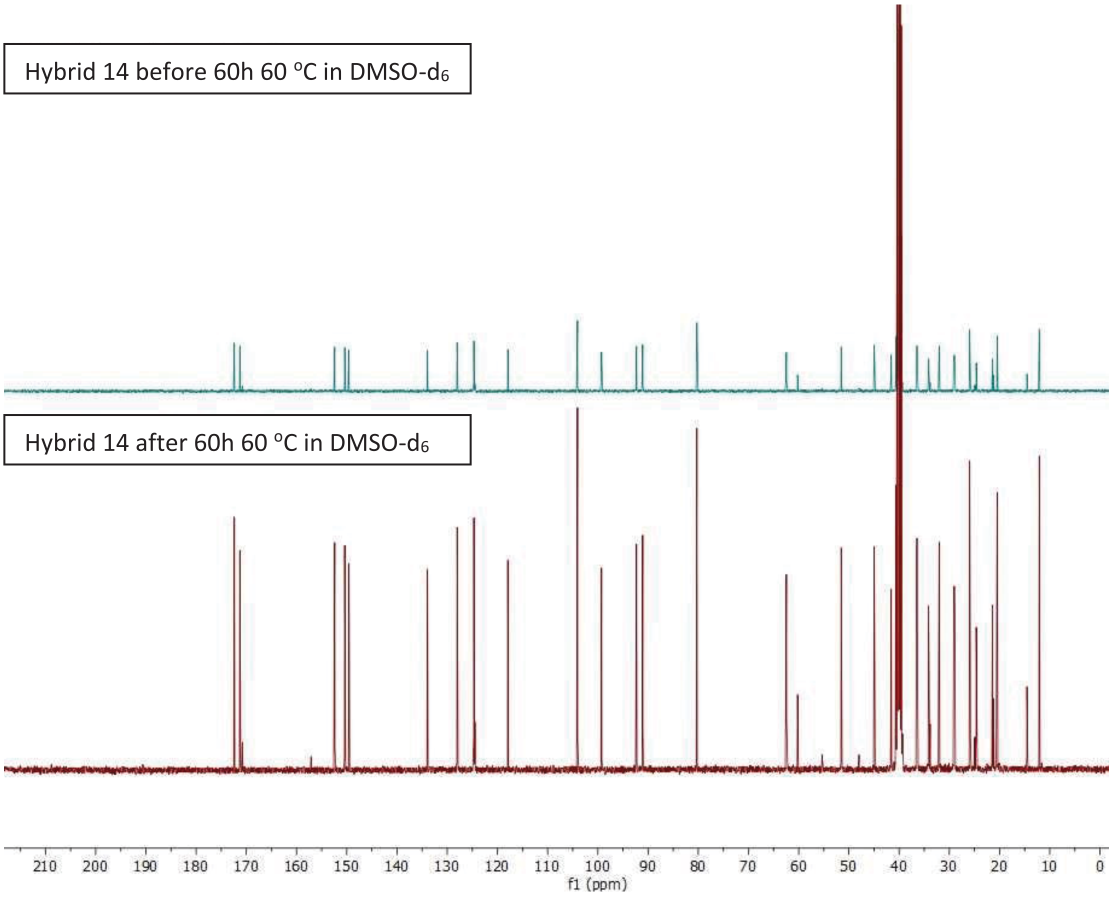


$^{1}$H NMR spectrum of hybrid **14** recorded (400 MHz, DMSO-$d_6$) after stirring in PBS 84 h at rt, in Fetal Bovine Serum 48 h at 37 ºC, and in Human Serum 48 h at 37 ºC:

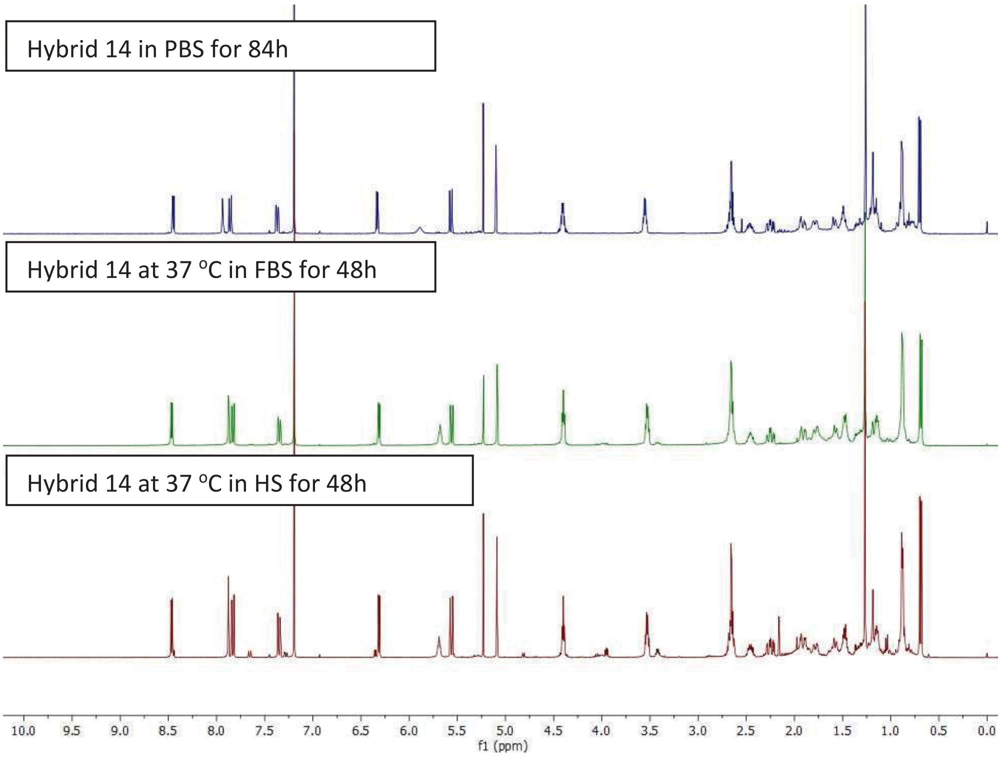

$^1H$ NMR spectrum of hybrid **14** recorded (400 MHz, DMSO-$d_6$) after stirring in Acetate buffer pH 5, 24 h at 37 °C:

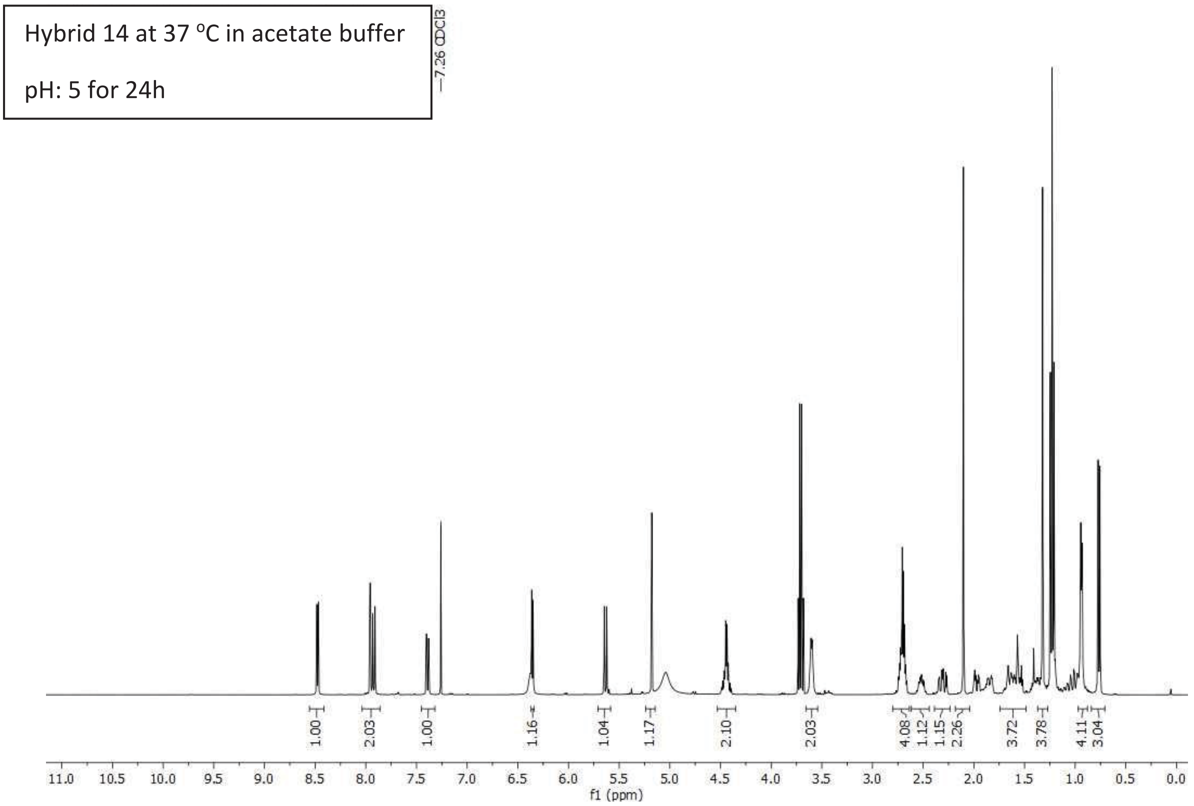


$^1H$ NMR spectrum of hybrid **14** recorded (400 MHz, DMSO-$d_6$) after stirring in the presence of Carboxylesterase:

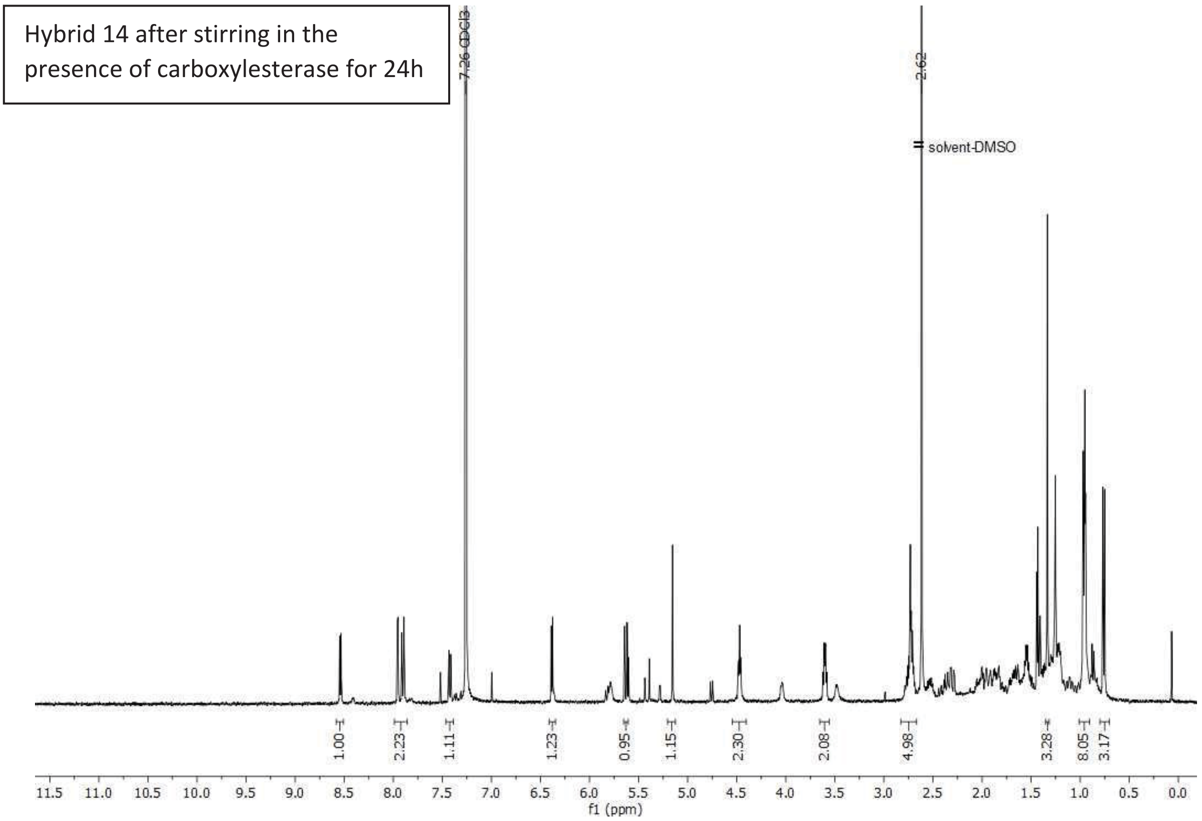

$^{1}$H NMR spectrum of hybrid **4** recorded (400 MHz, DMSO-$d_6$) after heating 60 h at 60 °C:

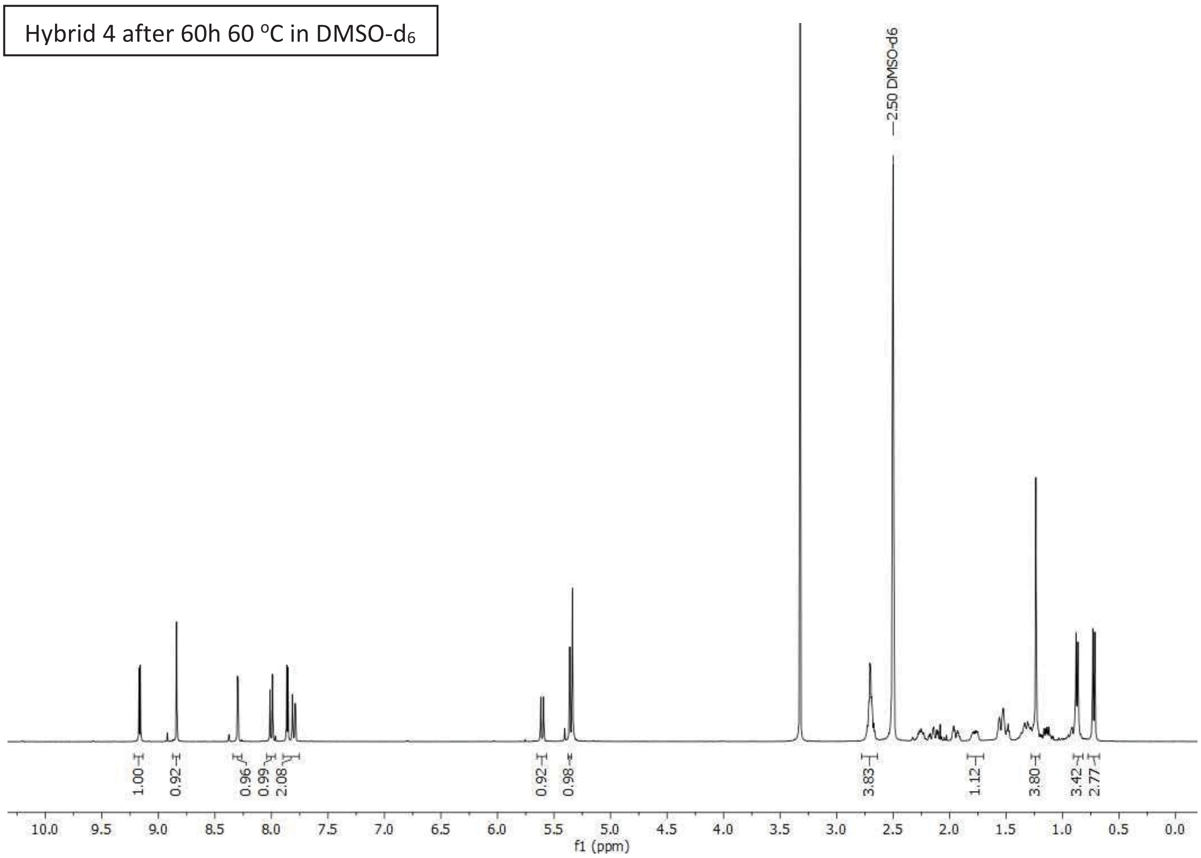


$^{1}$H NMR spectrum of hybrid **7** recorded (400 MHz, DMSO-$d_6$) after heating 60 h at 60 °C:

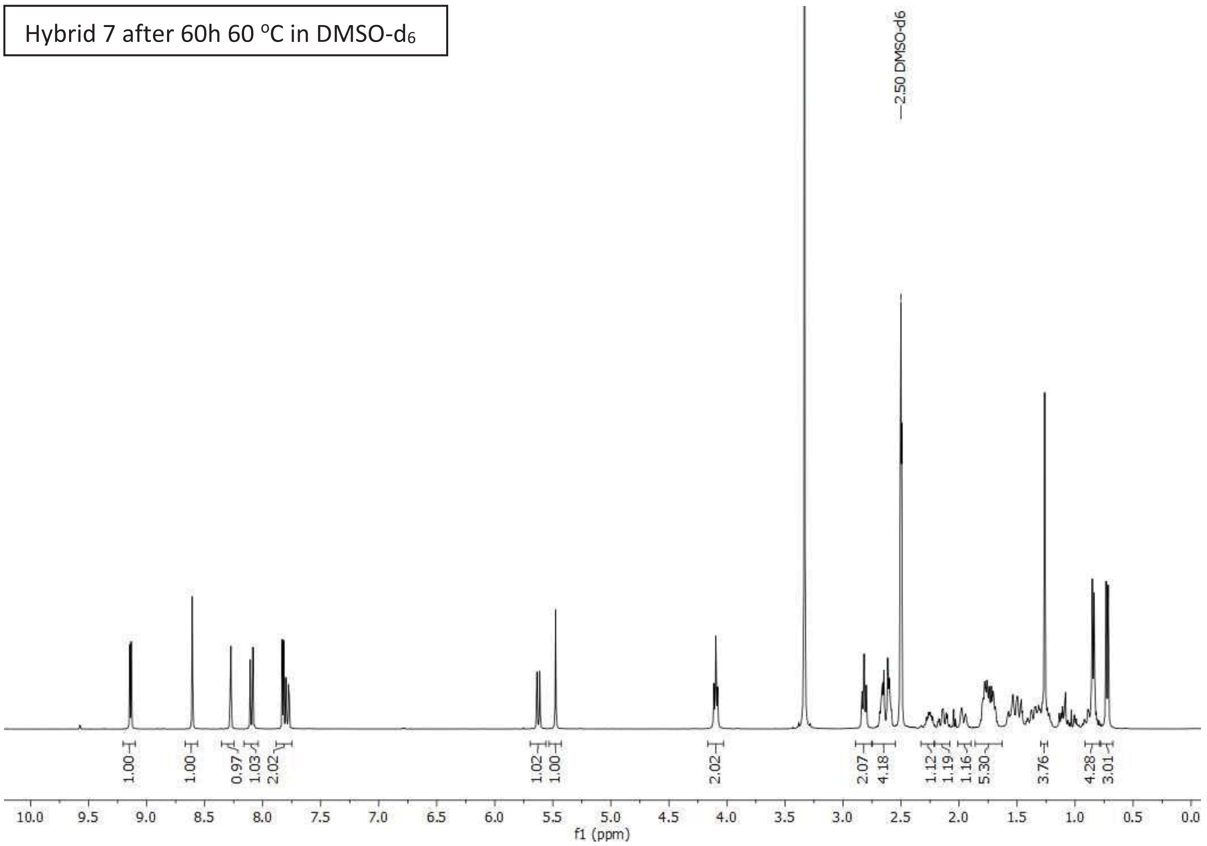

$^{13}C$ NMR spectrum of hybrid **7** recorded (400 MHz, DMSO-$d_6$) after heating 60 h at 60 °C:

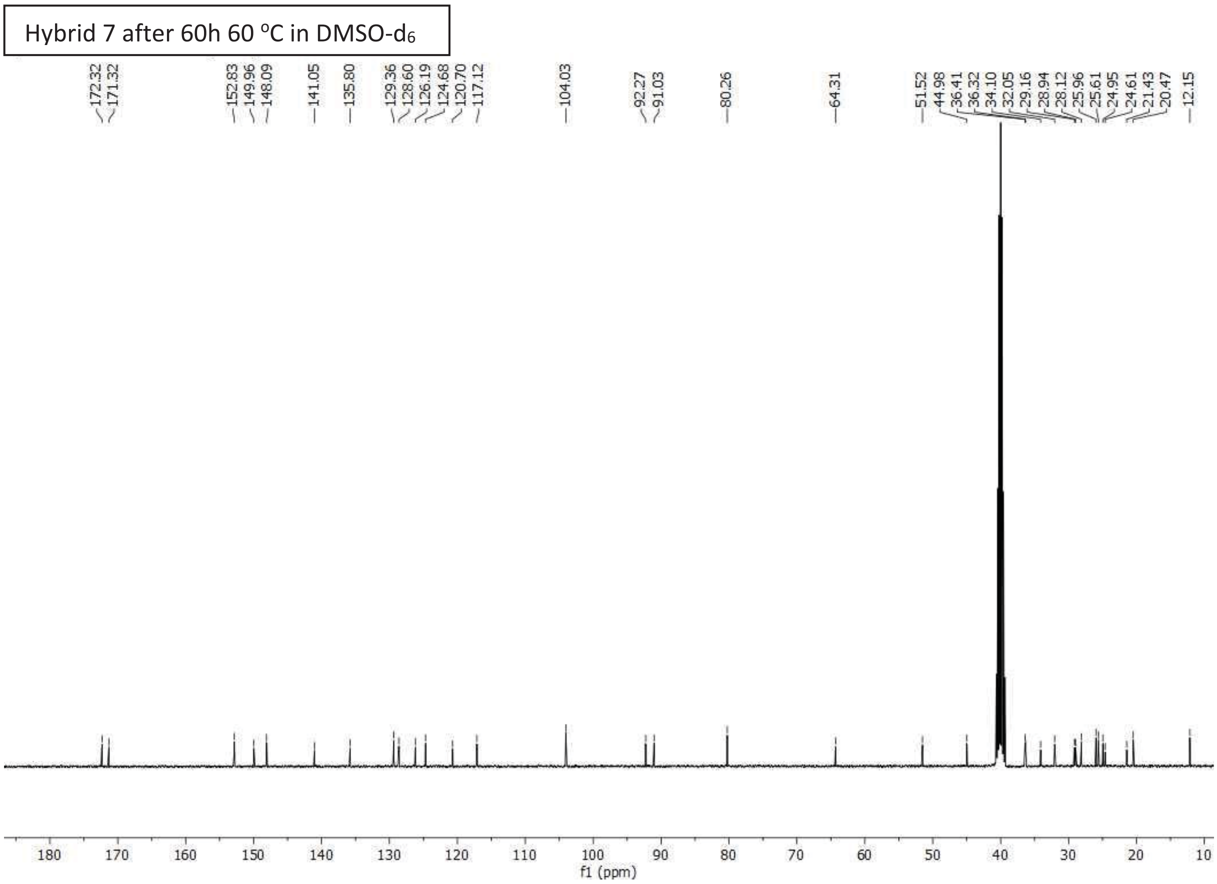


$^{1}H$ NMR spectrum of hybrid **9** recorded (400 MHz, DMSO-$d_6$) after heating 60 h at 60 °C:

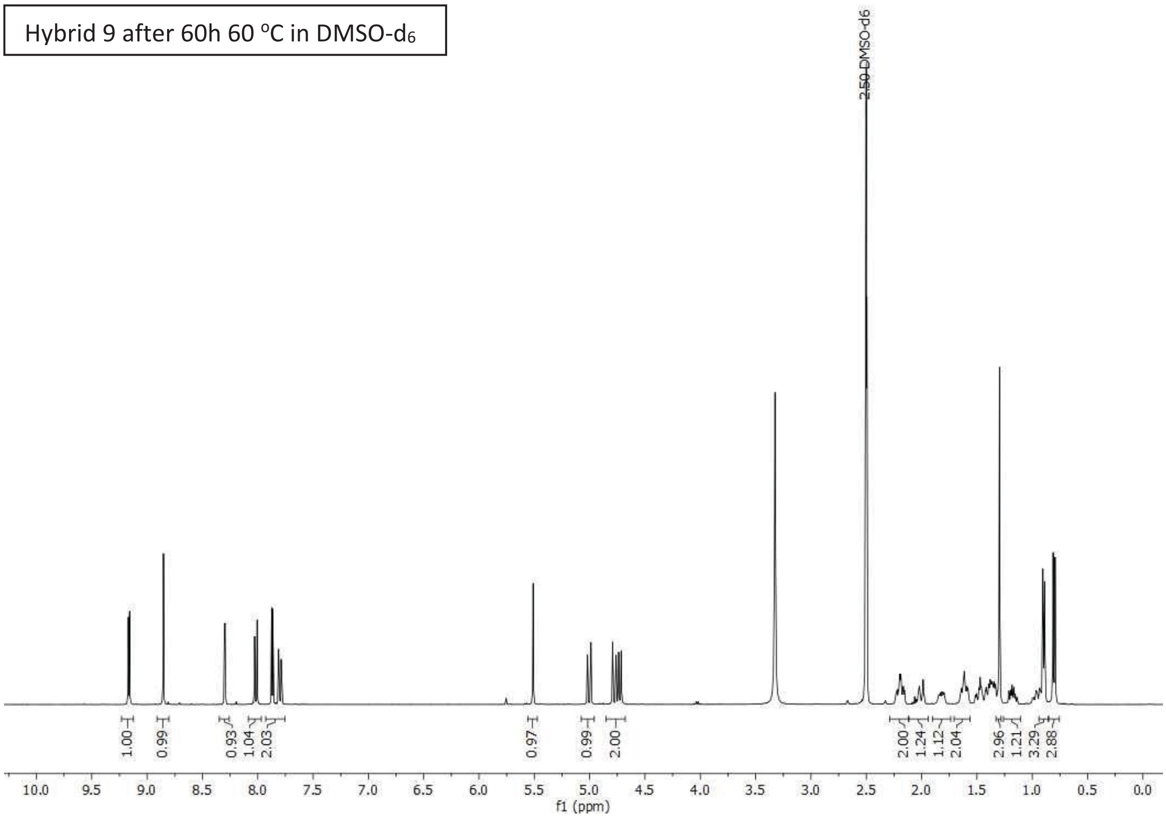

$^{1}$H NMR spectrum of hybrid **10** recorded (400 MHz, DMSO-$d_6$) after heating 60 h at 60 °C:

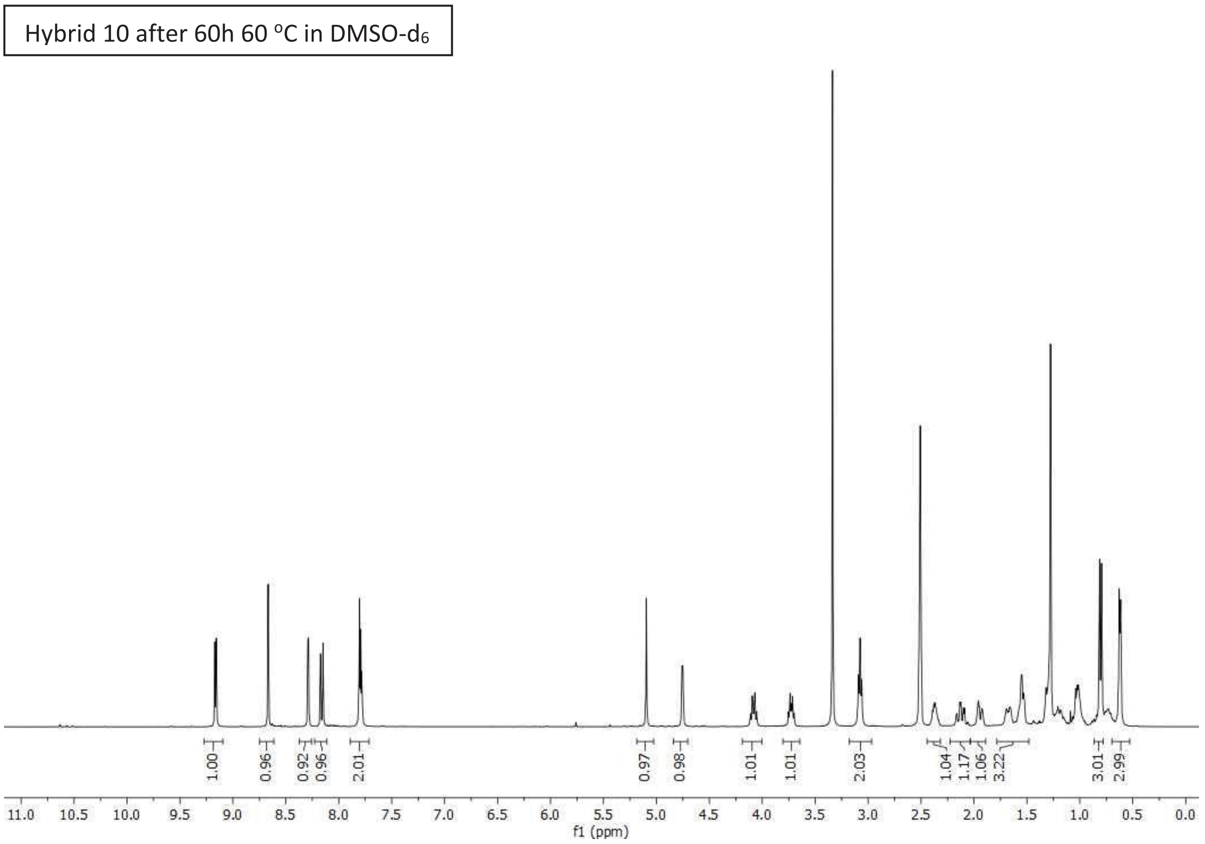


$^{13}$C NMR spectrum of hybrid **10** recorded (400 MHz, DMSO-$d_6$) after heating 60 h at 60 °C:

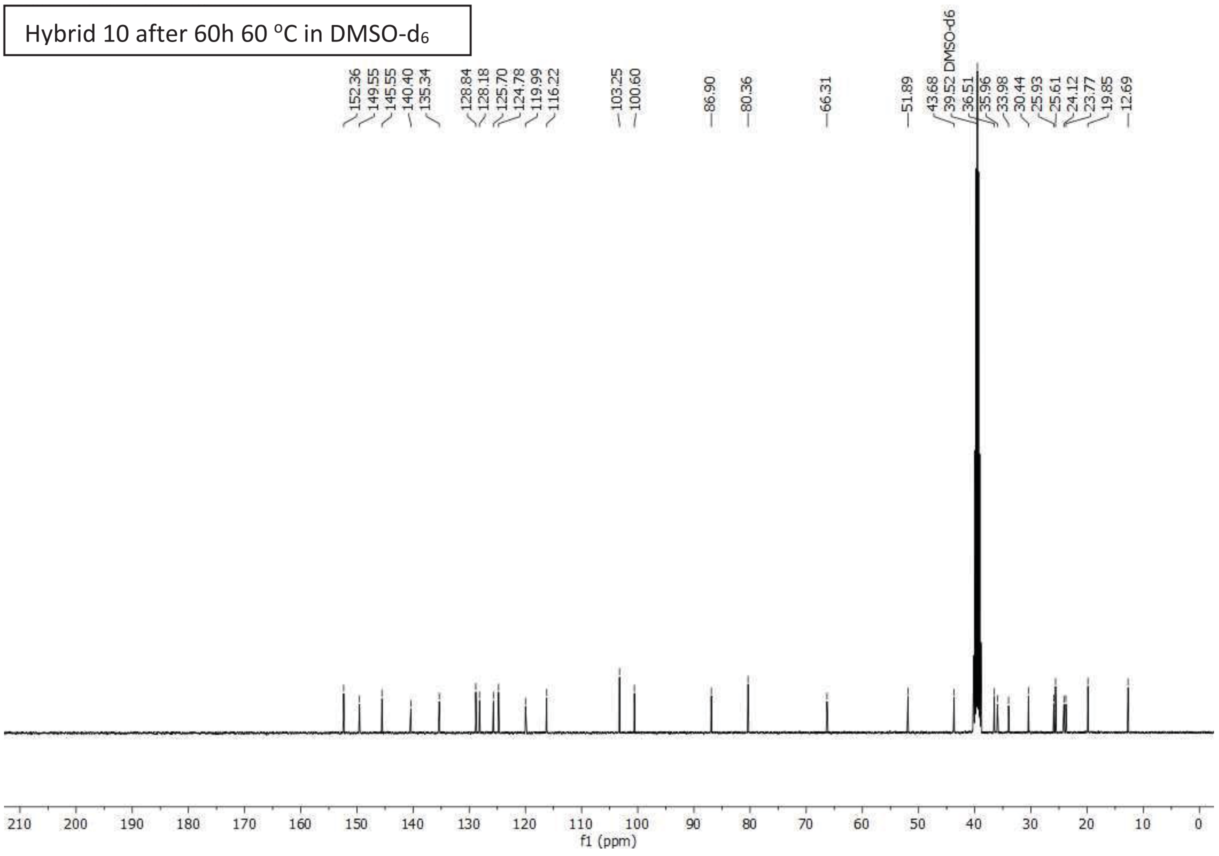

$^{1}H$ NMR spectrum of hybrid **11** recorded (400 MHz, DMSO-$d_6$) before heating 60 h at 60 ºC:

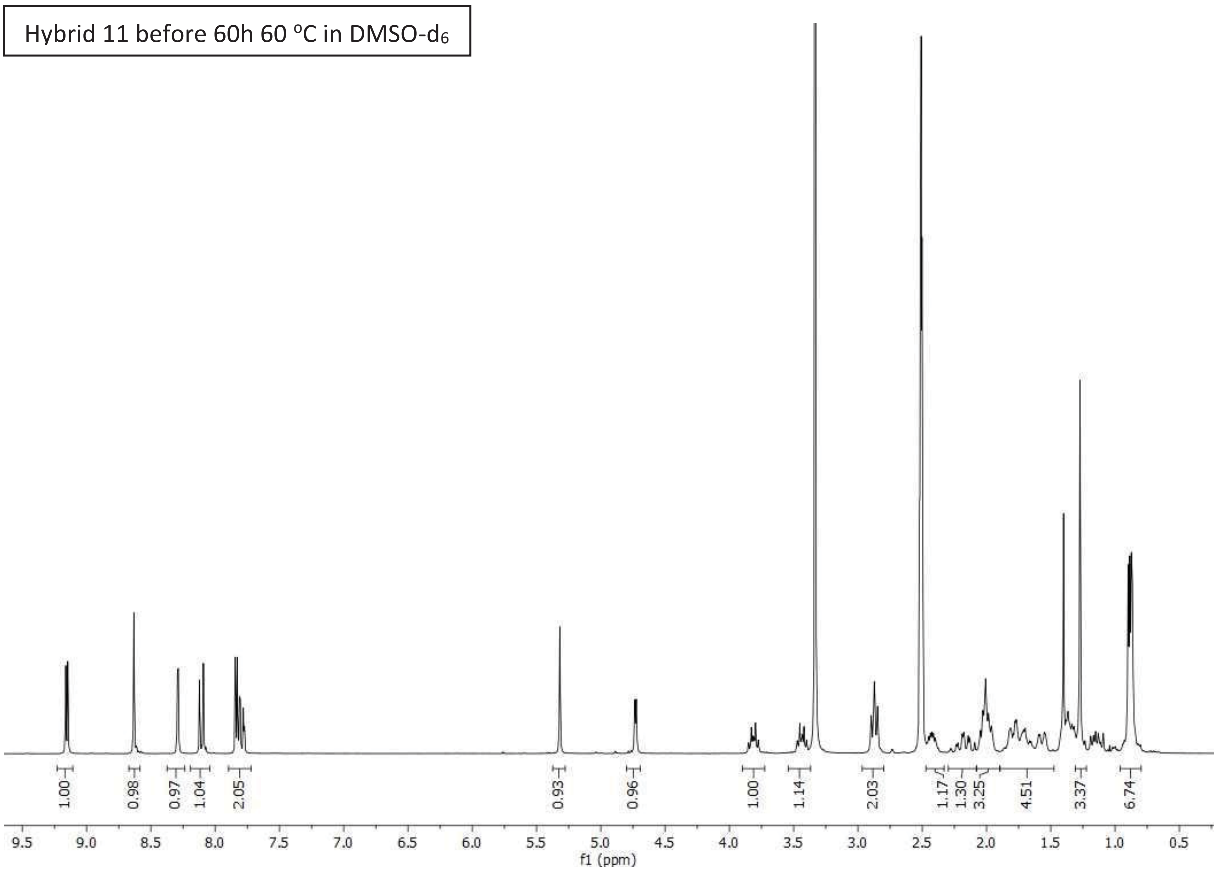


$^{1}H$ NMR spectrum of hybrid **11** recorded (400 MHz, DMSO-$d_6$) after heating 60 h at 60 ºC:

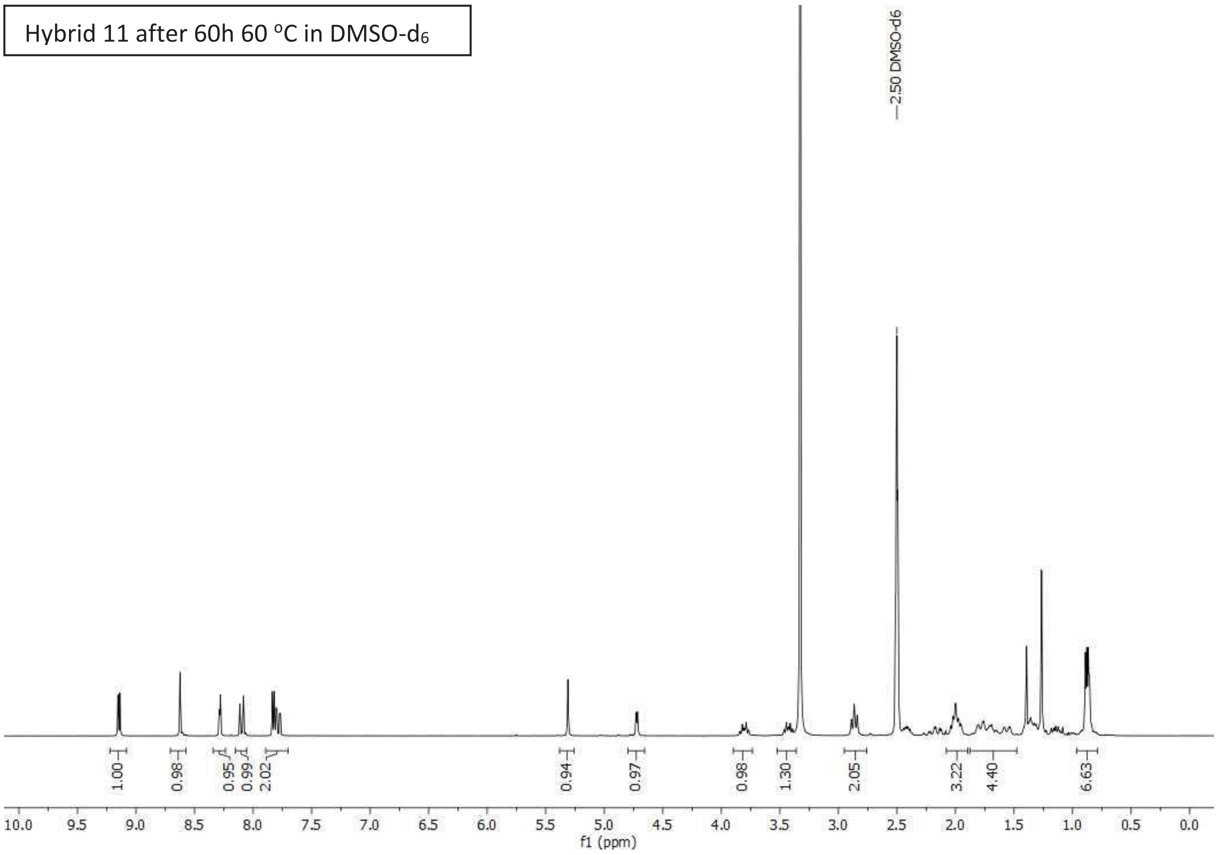

$^{13}C$ NMR spectrum of hybrid **11** recorded (400 MHz, DMSO-$d_6$) before heating 60 h at 60 $^oC$:

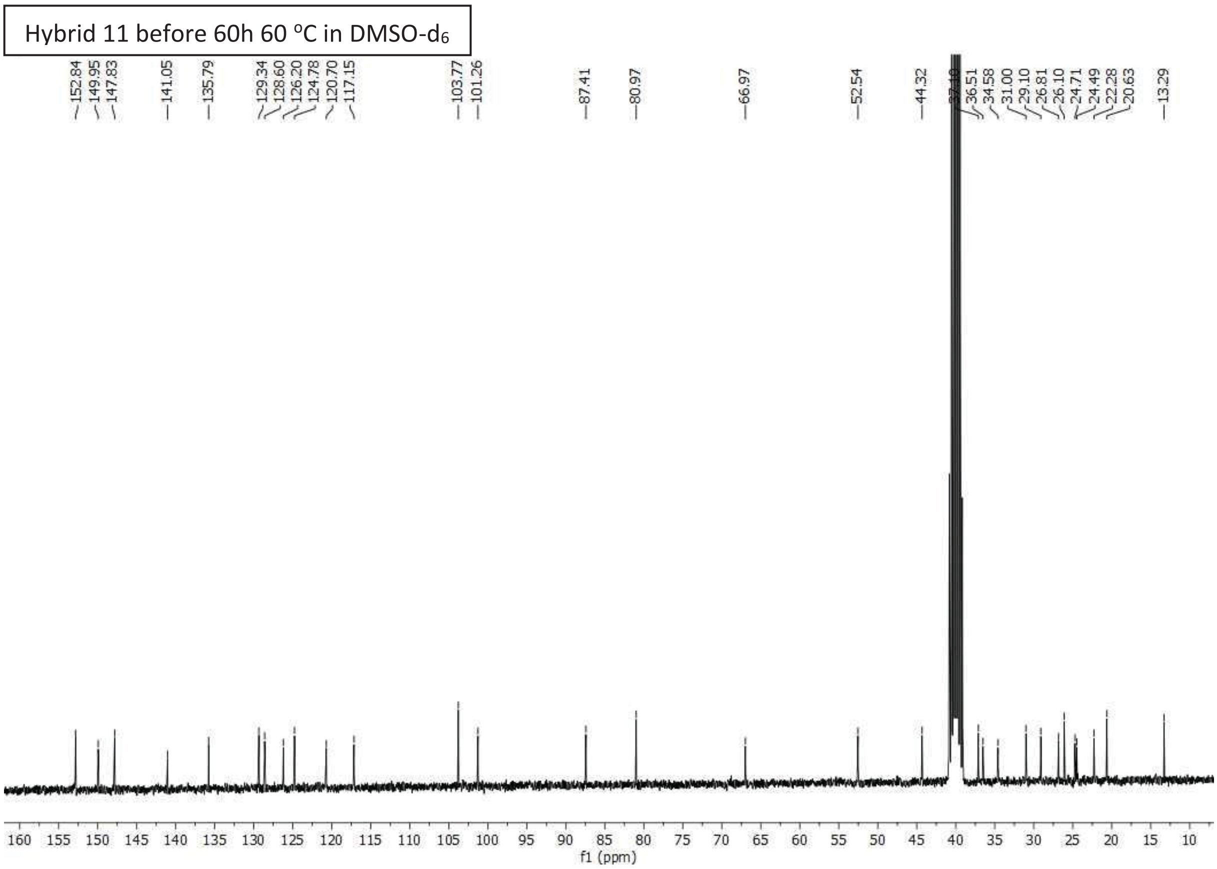


$^{13}C$ NMR spectrum of hybrid **11** recorded (400 MHz, DMSO-$d_6$) after heating 60 h at 60 $^oC$:

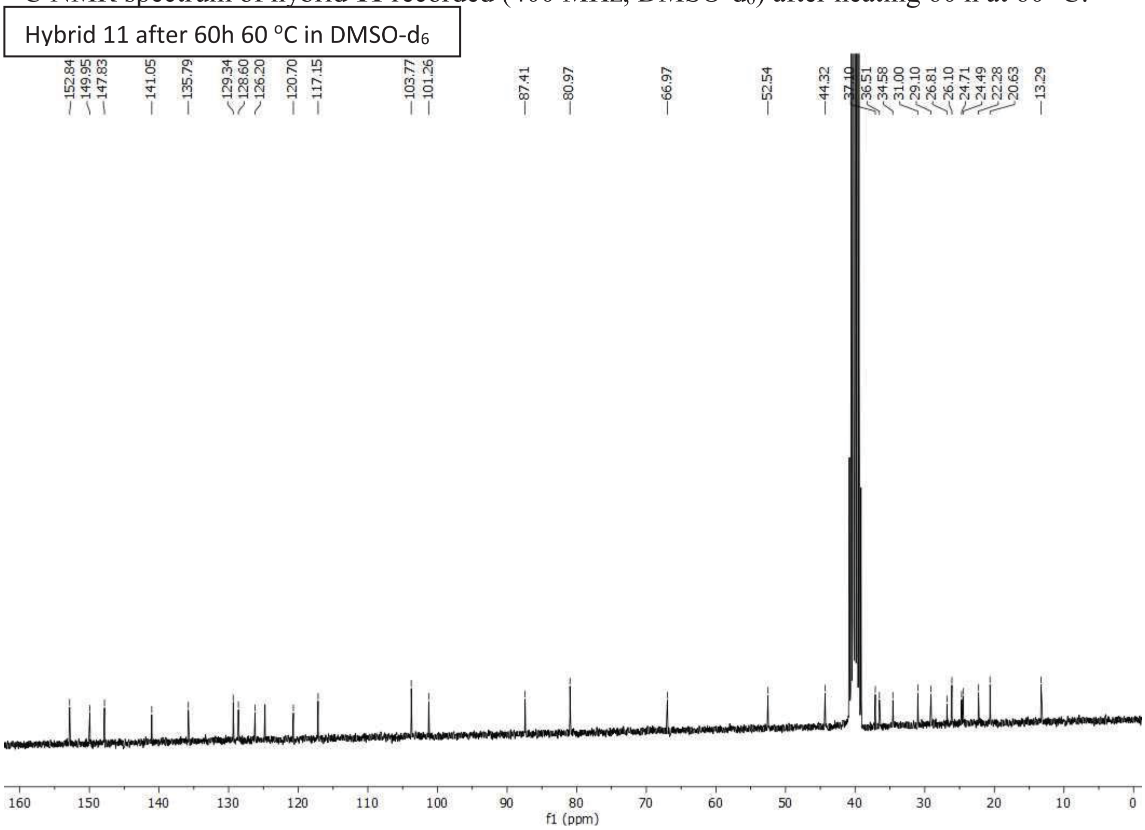

$^1$H NMR spectrum of hybrid **1** recorded (400 MHz, DMSO-d$_6$) after heating 60 h at 60 °C:

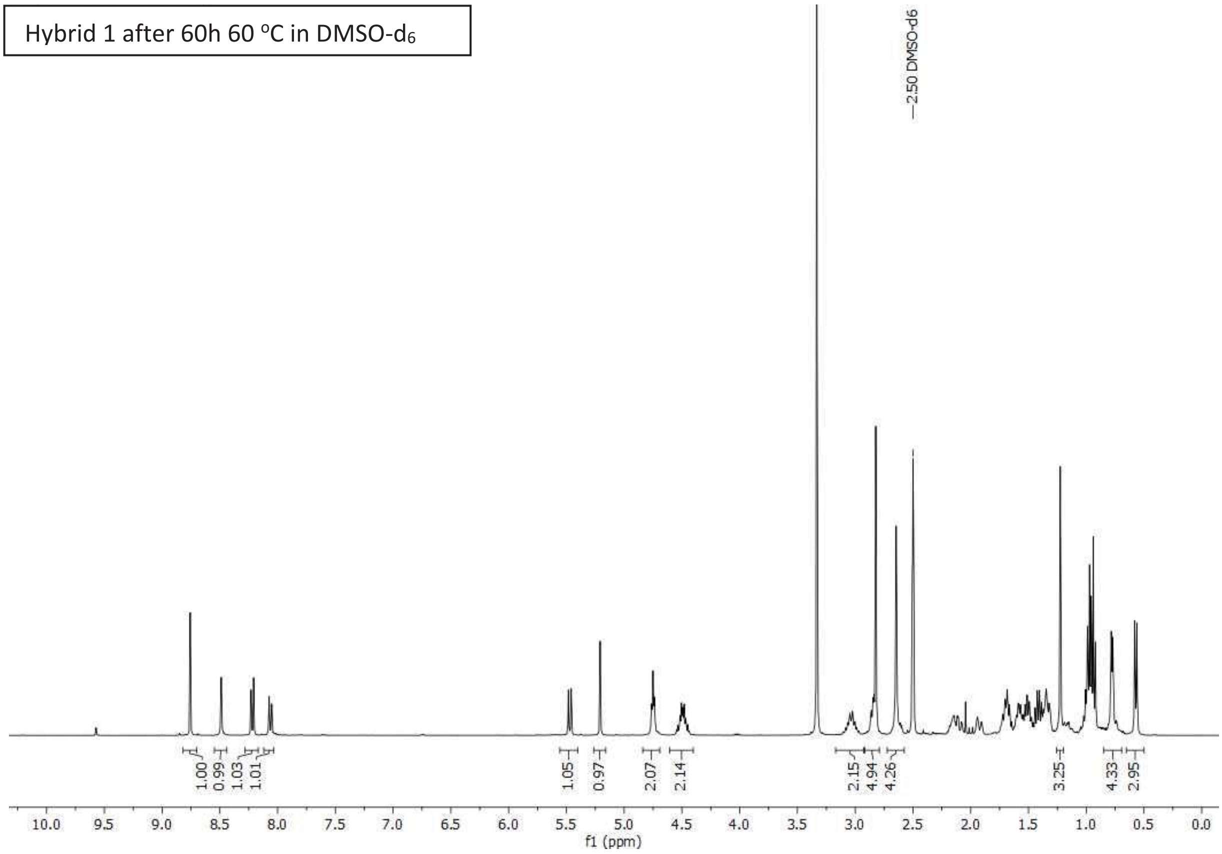

## 7. Filtering PAINS Elements among Hybrid Compounds 1 - 17

**Table S4. Results of screening using all three available PAINS filters**

| Hybrids | Smiles |
|---|---|
| Hybrid 1 | CCCCc1nc(C)c2ccc(cc2c1CCCC)-c1cn(CCOC(=O)CCC(=O)O[C@@H]2O[C@@H]3O[C@@]4(C)CCC5[C@H](C)CCC([C@H]2C)[C@@]35OO4)nn1 |
| Hybrid 2 | C[C@@H]1CCC2[C@@H](C)[C@H](OC(=O)CCC(=O)OCCn3cc(nn3)-c3ccc4c(C)nc(-c5ccccc5)c(-c5ccccc5)c4c3)O[C@@H]3O[C@@]4(C)CCC1[C@@]23OO4 |
| Hybrid 3 | C[C@@H]1CCC2C(C)[C@H](OC(=O)CCC(=O)OCCn3cc(nn3)-c3ccc4c(C)nc(-c5ccccc5)c(-c5ccccc5)c4c3)O[C@@H]3O[C@]4(C)O[C@]23[C@H]1C[C@H]4O |
| Hybrid 4 | C[C@@H]1CCC2[C@@H](C)[C@H](OC(=O)CCC(=O)OCc3cn(nn3)-c3ccnc4cc(Cl)ccc34)O[C@@H]3O[C@@]4(C)CCC1[C@@]23OO4 |
| Hybrid 5 | C[C@@H]1CCC2[C@@H](C)[C@H](OC(=O)CCC(=O)OCCc3cn(nn3)-c3ccnc4cc(Cl)ccc34)O[C@@H]3O[C@@]4(C)CCC1[C@@]23OO4 |
| Hybrid 6 | C[C@@H]1CCC2[C@@H](C)[C@H](OC(=O)CCC(=O)OCCCc3cn(nn3)-c3ccnc4cc(Cl)ccc34)O[C@@H]3O[C@@]4(C)CCC1[C@@]23OO4 |
| Hybrid 7 | C[C@@H]1CCC2[C@@H](C)[C@H](OC(=O)CCC(=O)OCCCCc3cn(nn3)-c3ccnc4cc(Cl)ccc34)O[C@@H]3O[C@@]4(C)CCC1[C@@]23OO4 |
| Hybrid 8 | C[C@@H]1CCC2[C@@H](C)[C@@H](OCc3cn(nn3)-c3ccnc4cc(Cl)ccc34)O[C@@H]3O[C@@]4(C)CCC1[C@@]23OO4 |
| Hybrid 9 | C[C@@H]1CCC2[C@@H](C)[C@H](OCc3cn(nn3)-c3ccnc4cc(Cl)ccc34)O[C@@H]3O[C@@]4(C)CCC1[C@@]23OO4 |
| Hybrid 10 | C[C@@H]1CCC2[C@@H](C)[C@@H](OCCc3cn(nn3)-c3ccnc4cc(Cl)ccc34)O[C@@H]3O[C@@]4(C)CCC1[C@@]23OO4 |
| Hybrid 11 | C[C@@H]1CCC2[C@@H](C)[C@@H](OCCCc3cn(nn3)-c3ccnc4cc(Cl)ccc34)O[C@@H]3O[C@@]4(C)CCC1[C@@]23OO4 |
| Hybrid 12 | C[C@@H]1CCC2[C@@H](C)[C@@H](OCCCCc3cn(nn3)-c3ccnc4cc(Cl)ccc34)O[C@@H]3O[C@@]4(C)CCC1[C@@]23OO4 |
| Hybrid 13 | C[C@@H]1CCC2[C@@H](C)[C@H](OC(=O)CCC(=O)OCCOc3ccnc4cc(Cl)ccc34)O[C@@H]3O[C@@]4(C)CCC1[C@@]23OO4 |
| Hybrid 14 | C[C@@H]1CCC2[C@@H](C)[C@H](OC(=O)CCC(=O)OCCOc3ccnc4cc(Cl)ccc34)O[C@@H]3O[C@@]4(C)CCC1[C@@]23OO5 |
| Hybrid 15 | C[C@@H]1CCC2[C@@H](C)[C@H](OC(=O)CCC(=O)OCCOc3ccnc4cc(Cl)ccc34)O[C@@H]3O[C@@]4(C)CCC1[C@@]23OO6 |
| Hybrid 16 | C[C@@H]1CCC2[C@@H](C)[C@H](OC(=O)CCC(=O)N(CCCC#C)CCNc3ccnc4cc(Cl)ccc34)O[C@@H]3O[C@@]4(C)CCC1[C@@]23OO4 |
| Hybrid 17 | CC1CCC2[C@@H](C)[C@H](OC(=O)CCC(=O)OCCNC(=O)C(CCCC#C)c3ccnc4cc(Cl)ccc34)O[C@@H]3O[C@@]4(C)CCC1[C@@]23OO4 |

| Hybrids | MW | logP | logD | logSw | tPSA | RotatableB | RigidB | Flexibility | HBD | HBA | HBD_HBA | Rings | MaxSizeRing | NumCharges | TotalCharge | HeavyAtoms | CarbonAtoms |
|---|---|---|---|---|---|---|---|---|---|---|---|---|---|---|---|---|---|
| Hybrid 1 | 732.91 | 8.1 | 8.79 | -8.53 | 133.12 | 16 | 37 | 0.3 | 0 | 12 | 12 | 3 | 19 | 0 | 0 | 53 | 41 |
| Hybrid 2 | 772.88 | 7.77 | 8.95 | -8.93 | 133.12 | 12 | 49 | 0.2 | 0 | 12 | 12 | 5 | 19 | 0 | 0 | 57 | 45 |
| Hybrid 3 | 772.88 | 6.75 | 7.68 | -8.28 | 144.12 | 12 | 48 | 0.2 | 1 | 12 | 13 | 5 | 16 | 0 | 0 | 57 | 45 |
| Hybrid 4 | 627.08 | 4.7 | 5.75 | -6.24 | 133.12 | 9 | 37 | 0.2 | 0 | 12 | 12 | 3 | 19 | 0 | 0 | 44 | 31 |
| Hybrid 5 | 641.11 | 5.16 | 5.98 | -6.54 | 133.12 | 10 | 37 | 0.21 | 0 | 12 | 12 | 3 | 19 | 0 | 0 | 45 | 32 |
| Hybrid 6 | 655.14 | 5.52 | 6.27 | -6.78 | 133.12 | 11 | 37 | 0.23 | 0 | 12 | 12 | 3 | 19 | 0 | 0 | 46 | 33 |
| Hybrid 7 | 669.16 | 5.87 | 6.72 | -7.02 | 133.12 | 12 | 37 | 0.24 | 0 | 12 | 12 | 3 | 19 | 0 | 0 | 47 | 34 |
| Hybrid 8 | 527.01 | 4.9 | 5.75 | -6.09 | 89.75 | 4 | 35 | 0.1 | 0 | 9 | 9 | 3 | 19 | 0 | 0 | 37 | 27 |
| Hybrid 9 | 527.01 | 4.9 | 5.75 | -6.09 | 89.75 | 4 | 35 | 0.1 | 0 | 9 | 9 | 3 | 19 | 0 | 0 | 37 | 27 |
| Hybrid 10 | 541.04 | 5.84 | 5.98 | -6.7 | 89.75 | 5 | 35 | 0.13 | 0 | 9 | 9 | 3 | 19 | 0 | 0 | 38 | 28 |
| Hybrid 11 | 555.06 | 6.19 | 6.27 | -6.94 | 89.75 | 6 | 35 | 0.15 | 0 | 9 | 9 | 3 | 19 | 0 | 0 | 39 | 29 |
| Hybrid 12 | 569.09 | 6.55 | 6.71 | -7.18 | 89.75 | 7 | 35 | 0.17 | 0 | 9 | 9 | 3 | 19 | 0 | 0 | 40 | 30 |
| Hybrid 13 | 590.06 | 4.77 | 5.89 | -5.94 | 111.64 | 10 | 32 | 0.24 | 0 | 10 | 10 | 2 | 19 | 0 | 0 | 41 | 30 |
| Hybrid 14 | 589.08 | 4.78 | 5.24 | -5.94 | 114.44 | 10 | 32 | 0.24 | 1 | 10 | 11 | 2 | 19 | 0 | 0 | 41 | 30 |
| Hybrid 15 | 588.09 | 4.21 | 4.32 | -5.64 | 117.24 | 9 | 33 | 0.21 | 2 | 10 | 12 | 2 | 19 | 0 | 0 | 41 | 30 |
| Hybrid 16 | 654.19 | 5.32 | 5.5 | -6.54 | 108.45 | 12 | 34 | 0.26 | 1 | 10 | 11 | 2 | 19 | 0 | 0 | 46 | 35 |
| Hybrid 17 | 697.21 | 6.16 | 6.62 | -7.2 | 131.51 | 14 | 35 | 0.29 | 1 | 11 | 12 | 2 | 19 | 0 | 0 | 49 | 37 |

| Hybrids | HeteroAtoms | ratioH/C | Lipinski_Violation | Solubility(mg/l) | SolubilityForecastIndex | Oral_Bioavailability_VEBER | Oral_Bioavailability_EGAN | TrafficLights | 4_400 |
|---|---|---|---|---|---|---|---|---|---|
| Hybrid 1 | 12 | 0.29 | 3 | 144.3 | Reduced Solubility | Low | Low | 7 | bad |
| Hybrid 2 | 12 | 0.27 | 3 | 102.78 | Reduced Solubility | Low | Low | 7 | bad |
| Hybrid 3 | 12 | 0.27 | 3 | 195.43 | Reduced Solubility | Low | Low | 8 | bad |
| Hybrid 4 | 13 | 0.42 | 2 | 1228.32 | Reduced Solubility | Good | Good | 5 | bad |
| Hybrid 5 | 13 | 0.41 | 3 | 925.1 | Reduced Solubility | Low | Low | 6 | bad |
| Hybrid 6 | 13 | 0.39 | 3 | 741.41 | Reduced Solubility | Low | Low | 7 | bad |
| Hybrid 7 | 13 | 0.38 | 3 | 597.49 | Reduced Solubility | Low | Low | 7 | bad |
| Hybrid 8 | 10 | 0.37 | 1 | 1189.48 | Reduced Solubility | Good | Good | 3 | bad |
| Hybrid 9 | 10 | 0.37 | 1 | 1189.48 | Reduced Solubility | Good | Good | 3 | bad |
| Hybrid 10 | 10 | 0.36 | 2 | 665.99 | Reduced Solubility | Good | Good | 4 | bad |
| Hybrid 11 | 10 | 0.34 | 2 | 540.09 | Reduced Solubility | Good | Good | 4 | bad |
| Hybrid 12 | 10 | 0.33 | 2 | 434.75 | Reduced Solubility | Good | Good | 4 | bad |
| Hybrid 13 | 11 | 0.37 | 1 | 1553.71 | Reduced Solubility | Good | Good | 4 | bad |
| Hybrid 14 | 11 | 0.37 | 1 | 1552.72 | Reduced Solubility | Good | Good | 4 | bad |
| Hybrid 15 | 11 | 0.37 | 1 | 2093.3 | Reduced Solubility | Good | Good | 4 | bad |
| Hybrid 16 | 11 | 0.31 | 2 | 947.04 | Reduced Solubility | Good | Good | 6 | bad |
| Hybrid 17 | 12 | 0.32 | 3 | 521.44 | Reduced Solubility | Low | Low | 7 | bad |

| Hybrids | 3_75 | Phospholipidosis | Fsp3 | StereoCenters | PPI_Friendly | PAINS_Filter_A | PAINS_Filter_B | PAINS_Filter_C | Result | Alert |
|---|---|---|---|---|---|---|---|---|---|---|
| Hybrid 1 | warning | NonInducer | 0.68 | 8 | Not Computed | 0 | 0 | 0 | Accepted | |
| Hybrid 2 | warning | NonInducer | 0.44 | 8 | Not Computed | 0 | 0 | 0 | Accepted | |
| Hybrid 3 | warning | NonInducer | 0.44 | 9 | Not Computed | 0 | 0 | 0 | Accepted | |
| Hybrid 4 | warning | NonInducer | 0.58 | 8 | Not Computed | 0 | 0 | 0 | Accepted | |
| Hybrid 5 | warning | NonInducer | 0.59 | 8 | Not Computed | 0 | 0 | 0 | Accepted | |
| Hybrid 6 | warning | NonInducer | 0.61 | 8 | Not Computed | 0 | 0 | 0 | Accepted | |
| Hybrid 7 | warning | NonInducer | 0.62 | 8 | Not Computed | 0 | 0 | 0 | Accepted | |
| Hybrid 8 | warning | NonInducer | 0.59 | 8 | Not Computed | 0 | 0 | 0 | Accepted | |
| Hybrid 9 | warning | NonInducer | 0.59 | 8 | Not Computed | 0 | 0 | 0 | Accepted | |
| Hybrid 10 | warning | NonInducer | 0.61 | 8 | Not Computed | 0 | 0 | 0 | Accepted | |
| Hybrid 11 | warning | NonInducer | 0.62 | 8 | Not Computed | 0 | 0 | 0 | Accepted | |
| Hybrid 12 | warning | NonInducer | 0.63 | 8 | Not Computed | 0 | 0 | 0 | Accepted | |
| Hybrid 13 | warning | NonInducer | 0.63 | 8 | Not Computed | 0 | 0 | 0 | Accepted | |
| Hybrid 14 | warning | NonInducer | 0.63 | 8 | Not Computed | 0 | 0 | 0 | Accepted | |
| Hybrid 15 | warning | NonInducer | 0.63 | 8 | Not Computed | 0 | 0 | 0 | Accepted | |
| Hybrid 16 | warning | NonInducer | 0.63 | 8 | Not Computed | 0 | 0 | 0 | Accepted | |
| Hybrid 17 | warning | NonInducer | 0.62 | 9 | Not Computed | 0 | 0 | 0 | Accepted | |

## 8. References


1 Wang, H., Koeller, J., Liu, W. & Ackermann, L. Cobalt(III)-catalyzed C-H/N-O functionalizations: isohypsic access to isoquinolines. *Chem. Eur. J.* **21**, 15525-15528 (2015).

2 De Souza, M. V., Pais, K. C., Kaiser, C. R., Peralta, M. A., de, L. F. M. & Lourenco, M. C. Synthesis and in vitro antitubercular activity of a series of quinoline derivatives. *Bioorg Med Chem* **17**, 1474-1480 (2009).

3 Natarajan, J. K., Alumasa, J. N., Yearick, K., Ekoue-Kovi, K. A., Casabianca, L. B., de Dios, A. C., Wolf, C. & Roepe, P. D. 4-N-, 4-S-, and 4-O-chloroquine analogues: influence of side chain length and quinolyl nitrogen p K a on activity vs chloroquine resistant malaria. *J. Med. Chem.* **51**, 3466-3479 (2008).

4 Lavis, L. D. Ester bonds in prodrugs. *ACS Chem. Biol.* **3**, 203-206 (2008).

5 Uludag, M. O., Ergun, B. C., Alkan, D. A., Ercan, N., Ozkan, G. Y. & Banoglu, E. Stable ester and amide conjugates of some NSAIDs as analgesic and antiinflammatory compounds with improved biological activity. *Turk J. Chem.* **35**, 427-439 (2011).

6 Perez, C., Daniel, K. B. & Cohen, S. M. Evaluating prodrug strategies for esterase-triggered release of alcohols. *ChemMedChem* **8**, 1662-1667 (2013).

7 Lambros, C. & Vanderberg, J. P. Synchronization of plasmodium falciparum erythrocytic stages in culture. *J Parasitol* **65**, 418-420 (1979).

8 Smilkstein, M., Sriwilaijaroen, N., Kelly, J. X., Wilairat, P. & Riscoe, M. Simple and inexpensive fluorescence-based technique for high-throughput antimalarial drug screening. *Antimicrob. Agents Chemother.* **48**, 1803-1806 (2004).

9 Beez, D., Sanchez, C. P., Stein, W. D. & Lanzer, M. Genetic predisposition favors the acquisition of stable artemisinin resistance in malaria parasites. *Antimicrob. Agents Chemother.* **55**, 50-55 (2011).

10 Sanchez, B. A., Mota, M. M., Sultan, A. A. & Carvalho, L. H. Plasmodium berghei parasite transformed with green fluorescent protein for screening blood schizontocidal agents. *Int. J. Parasitol.* **34**, 485-490 (2004).

11 Combrinck, J. M., Mabotha, T. E., Ncokazi, K. K., Ambele, M. A., Taylor, D., Smith, P. J., Hoppe, H. C. & Egan, T. J. J. A. c. b. Insights into the role of heme in the mechanism of action of antimalarials. **8**, 133-137 (2012).

12 Wang, J., Zhang, C. J., Chia, W. N., Loh, C. C., Li, Z., Lee, Y. M., He, Y., Yuan, L. X., Lim, T. K., Liu, M., Liew, C. X., Lee, Y. Q., Zhang, J., Lu, N., Lim, C. T., Hua, Z. C., Liu, B., Shen, H. M., Tan, K. S. & Lin, Q. Haem-activated promiscuous targeting of artemisinin in Plasmodium falciparum. *Nat. Commun.* **6**, 10111 (2015).

13 Lagorce, D., Bouslama, L., Becot, J., Miteva, M. A. & Villoutreix, B. O. FAF-Drugs4: free ADME-tox filtering computations for chemical biology and early stages drug discovery. *Bioinformatics* **33**, 3658-3660 (2017).
